# Mechanism of Gold-Catalyzed Arylation-Lactonization: A Density Functional Theory Study on the Role of the (MIC^N)AuCl Complex in Au(I)/Au(III) Catalysis

**DOI:** 10.1021/acs.inorgchem.5c01666

**Published:** 2025-06-17

**Authors:** Roger Monreal-Corona, Xavi Ribas, Anna Pla-Quintana, Albert Poater

**Affiliations:** Institut de Química Computacional i Catàlisi (IQCC) and Departament de Química, 117394 Universitat de Girona (UdG), Facultat de Ciències, C/Maria Aurèlia Capmany, 69, Girona, Catalunya 17003, Spain

## Abstract

Gold-catalyzed redox transformations via Au­(I)/Au­(III) cycles offer efficient oxidative addition and reductive elimination under mild, oxidant-free conditions. Recent studies highlight the role of hemilabile mesoionic carbene (MIC) ligands in stabilizing key intermediates. Using DFT, we investigated the mechanism of the arylation-lactonization of γ-alkenoic acids, revealing two viable pathways, *cis* and *trans*, each with distinct rate-determining steps. While the *trans* pathway avoids decomposition of the catalyst, its lactonization step is hindered by a high barrier. In contrast, the *cis* pathway features competing productive and decomposition routes. By correlating computed activation barriers with experimental yields, we built statistically significant multivariable models (*R* ^2^ = 0.919), enabling the prediction of product yields across various substituted aryl iodides. These models revealed clear electronic and steric trends. Additionally, ligand modifications suggest that *trans*-selective oxidative addition can be improved through steric tuning with the *trans* effect also influencing selectivity. Overall, this study provides valuable design principles for future gold-catalyzed redox processes.

## Graphical abstract

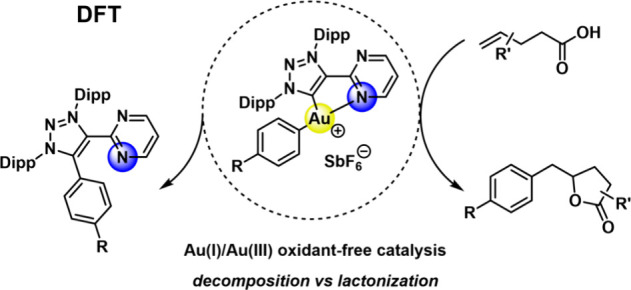

## Introduction

Gold-catalyzed transformations have garnered significant attention due to their unique reactivity and potential to facilitate challenging organic transformations,^1,2^ under mild conditions.^3–6^ Among these, redox Au­(I)/Au­(III) catalysis has emerged as a promising platform for enabling oxidative addition and reductive elimination processes,^7–10^ steps traditionally associated with other transition metals.^11,12^ The ability to achieve these transformations without the need for external oxidants has profound implications for sustainable and efficient catalysis,^13–21^ confirmed by computational mechanistic insights.^22–24^ Despite recent advancements, specially with Au­(III),^25^ Au­(I) species remain reluctant to undergo oxidative addition,^26–29^ posing a significant challenge to the broader application of this catalytic cycle,^30^ with special emphasis on redox Au­(I)/Au­(III) catalysis.^31–33^

Efforts to overcome this limitation have led to the development of innovative ligand designs capable of stabilizing key intermediates and promoting challenging bond activations. The experimental study by Ribas et al.^34^ represents a landmark achievement in this area. By employing bidentate hemilabile mesoionic carbene (MIC^N) ligands, the authors introduced a catalytic system that facilitates oxidant-free Au­(I)/Au­(III) redox cycles (Scheme 1). This approach broadens the scope of hemilabile MIC^N,^35–38^ MeDalphos,^39–42^ and NHC^N ligands,^43,44^ which enable the stabilization of square-planar Au­(III) intermediates crucial for facilitating oxidative addition of aryl iodides and strained C*sp* ^2^–C*sp* ^2^ bonds. Importantly, the system also catalyzes the arylation-lactonization of γ-alkenoic acids to form γ-benzyl-γ-butyrolactones, showcasing the synthetic utility of this strategy.

**Scheme 1:**
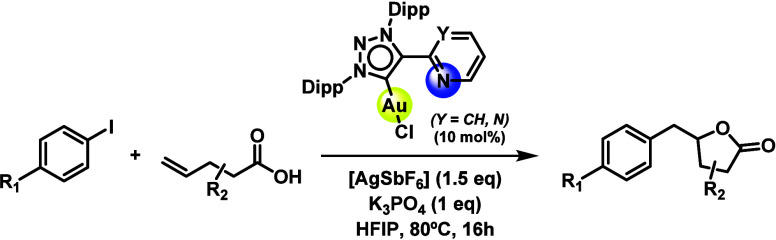
Optimal Experimental Conditions for the Application of the (MIC^N)­Au­(I) Complexes to the Arylation-Lactonization Reaction of γ-Alkenoic Acids^34^

A defining characteristic of MIC^N ligands is their ability to modulate stability and reactivity by adopting a flexible coordination mode at the gold center, thereby enabling the transition between linear Au­(I) and square-planar Au­(III) geometries. This adaptability is critical for promoting oxidative addition while also enabling subsequent catalytic steps, such as π-coordination and reductive elimination. The study highlights how ligand design can overcome inherent challenges in gold catalysis, offering a more sustainable alternative to traditional systems that rely on external oxidants.

Although these experimental findings establish a solid foundation for understanding the potential of MIC^N ligands, they also prompt key mechanistic questions. For instance, what governs the energy barriers associated with the oxidative addition and lactonization steps? How do the electronic and steric properties of the aryl iodides influence the overall catalytic efficiency? Addressing these questions is essential for refining this catalytic system and exploring its broader applicability.

The present work builds on the experimental insights by employing density functional theory (DFT) calculations to dissect the detailed mechanism of the Au­(I)/Au­(III)-mediated arylation-lactonization reaction. Through computational analysis, we aim to elucidate the role of MIC^N ligands in stabilizing reactive intermediates and facilitating key transformations. By characterizing intermediates and transition states, this study seeks to quantify the energy landscape of the catalytic cycle and establish correlations between calculated activation barriers and experimental yields.^45^ These insights will not only enhance our understanding of the current system but also inform the rational design of next-generation gold catalysts.

By focusing on the fundamental principles of ligand design and redox behavior, this work contributes to the broader field of homogeneous catalysis, with implications for a wide range of transition metal-catalyzed processes. The integration of theoretical and experimental approaches underscores the importance of multidisciplinary efforts in advancing catalytic science and developing innovative solutions for sustainable chemical transformations.

## Results and Discussion

To gain detailed insights into the mechanism of the oxidant-free Au­(I)/Au­(III)-mediated arylation-lactonization reaction, we performed DFT calculations (Figure 1). The primary objective was to characterize the key intermediates and transition states of the catalytic cycle, thereby constructing a comprehensive energy profile for the reaction. This computational investigation builds on the experimental findings of Ribas et al.,^34^ who demonstrated the critical role of hemilabile MIC^N ligands in enabling this transformation.

**Figure 1:**
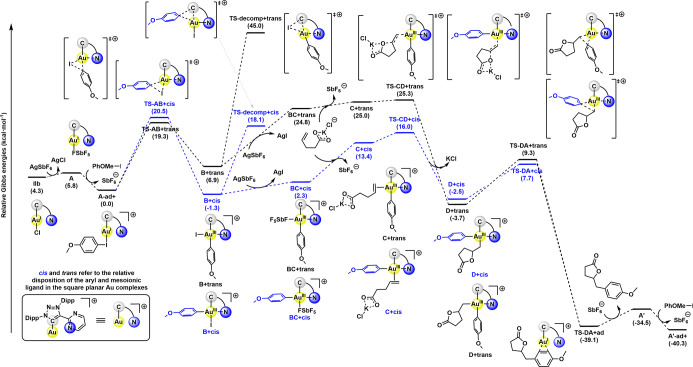
Gibbs energy profile (in kcal·mol^‑^^1^) for the Au­(I)/Au­(III)-mediated arylation-lactonization of γ-alkenoic acid with *p*MeO-phenyl iodide. The *cis* mechanism is depicted in blue, whereas the *trans* mechanism is depicted in black.

The reaction profile begins with the *in situ* generation of the cationic Au­(I) active species **A+**, which is 1.5 kcal·mol^‑^^1^ higher in energy than the precatalytic species **IIb**, and has a Mayer Bond Order (MBO) value^46^ of 0.129 for the Au–N interaction, in line with the assignation of the Au­(I) complex.^47,48^ Upon the addition of *p*MeO-phenyl iodide in the reaction mixture, reactant complex **A-ad+** is formed. From this minimum, we evaluated the two possibilities for the oxidative addition of aryl iodides to the Au­(I) complex, a key step traditionally considered challenging for gold catalysis. The reaction can proceed via *cis* or *trans* oxidative addition, with kinetic barriers of 20.5 and 19.3 kcal·mol^‑^^1^, respectively. These activation barriers for the oxidative addition process are in agreement with those reported by Bower et al.,^43^ who showed that the removal of the NMe_2_ substituent in hemilabile NHC-Au complexes increases the kinetic cost from 18.9 to 22.6 kcal·mol^‑^^1^, or the activation energy of 21.3 kcal·mol^‑^^1^ reported by Xing and Chen for a hemilabile (P, N) MeDalphos complex.^49^ Moreover, to gain more insights into the role of the MIC^N ligand, we performed distortion-interaction analysis on the oxidative addition transition state, showing that the hemilabile ligand promotes the oxidative addition process by minimizing the distortion of the substrate and catalyst (see Figure S1).

The oxidative addition step results in two possible Au­(III) intermediates, **B+trans** and **B+cis**, with the *cis* intermediate being 8.2 kcal·mol^‑^^1^ lower in energy than the *trans*. These intermediates display MBO values of 0.383 and 0.306, respectively, consistent with Au­(III) complexes that will persist until recovery of the **A+** species. As reported by Ribas et al.,^34^ two decomposition products were identified experimentally, arising from the reductive elimination of the preceding intermediates. For the decomposition pathway originating from **B+trans**, the activation energy is 45.0 kcal·mol^‑^^1^, leading to the formation of a ligand-iodide side product, which is obtained in negligible amounts. In contrast, for the decomposition pathway from **B+cis**, the activation barrier is significantly lower, at 19.4 kcal·mol^‑^^1^, resulting in the preferential formation and isolation of the ligand-aryl side product in higher yields.

Conversely, the *cis* and *trans* intermediates formed via oxidative addition can also undergo ligand dissociation at the gold center upon treatment with AgSbF_6_,^50^ facilitating the incorporation of deprotonated γ-alkenoic acid to yield the intermediate species **C+trans** and **C+cis**. Subsequently, intramolecular γ-lactonization is facilitated and the π-activation of the alkene induced by the gold center occurs, yielding alkylaryl Au­(III) intermediates **D+cis** and **D+trans**, which are nearly isoenergetic. The kinetic barrier for the lactonization step notably varies between the two conformations, requiring 25.3 kcal·mol^‑^^1^ to overcome **TS-CD+trans** and 17.3 kcal·mol^‑^^1^ to overcome **TS-CD+cis**. It is to be noted that the activation barriers for the lactonization step increase to 34.9 and 37.8 kcal·mol^‑^^1^ for **TS-CD+trans** and **TS-CD+cis**, respectively, underlying the importance of the K^+^ cation and the deprotonated alkenoic acid intermediate.

The last step of the transformation is the reductive elimination step that affords the γ-benzyl-γ-butyrolactone product and closes the catalytic cycle by regenerating the initial catalytically active hemilabile Au­(I) species through **TS-DA+trans** and **TS-DA+cis** with activation energies of 13.0 and 10.2 kcal·mol^‑1^, respectively. The complexity of the investigated transformation involves two distinct rate-determining steps (rds), depending on whether the reaction proceeds via the *cis* or *trans* mechanism (see Figure 2). For the *trans* mechanism, the rds corresponds to the intramolecular γ-lactonization, with a calculated activation energy of 25.3 kcal·mol^‑^^1^, while the oxidative addition of the aryl iodide to the Au­(I) complex occurs with a lower barrier of 19.3 kcal·mol^‑^^1^. In contrast, for the *cis* mechanism, the rds is the oxidative addition of the aryl iodide to the Au­(I) complex, featuring an activation energy of 20.5 kcal·mol^‑^^1^, while the intramolecular γ-lactonization step is slightly less demanding (17.3 kcal·mol^‑^^1^). Notably, in the *trans* energy profile, the decomposition pathway is nearly inaccessible, and not accessible either with a dual gold approach.^51–53^ However, for the *cis* pathway, decomposition competes with the formation of the desired product, exhibiting a comparable activation barrier of 19.4 kcal·mol^‑^^1^. These results indicate that for the substrate under investigation, the reaction proceeds predominantly via the *cis* pathway, as the *trans* oxidative addition of the aryl iodide is reversible. However, for other substrates, a reduction in the kinetic barrier associated with transition state **TS-CD+trans** to levels comparable to those of the *cis* pathway could render the *trans* mechanism competitive and thus operative.

**Figure 2:**
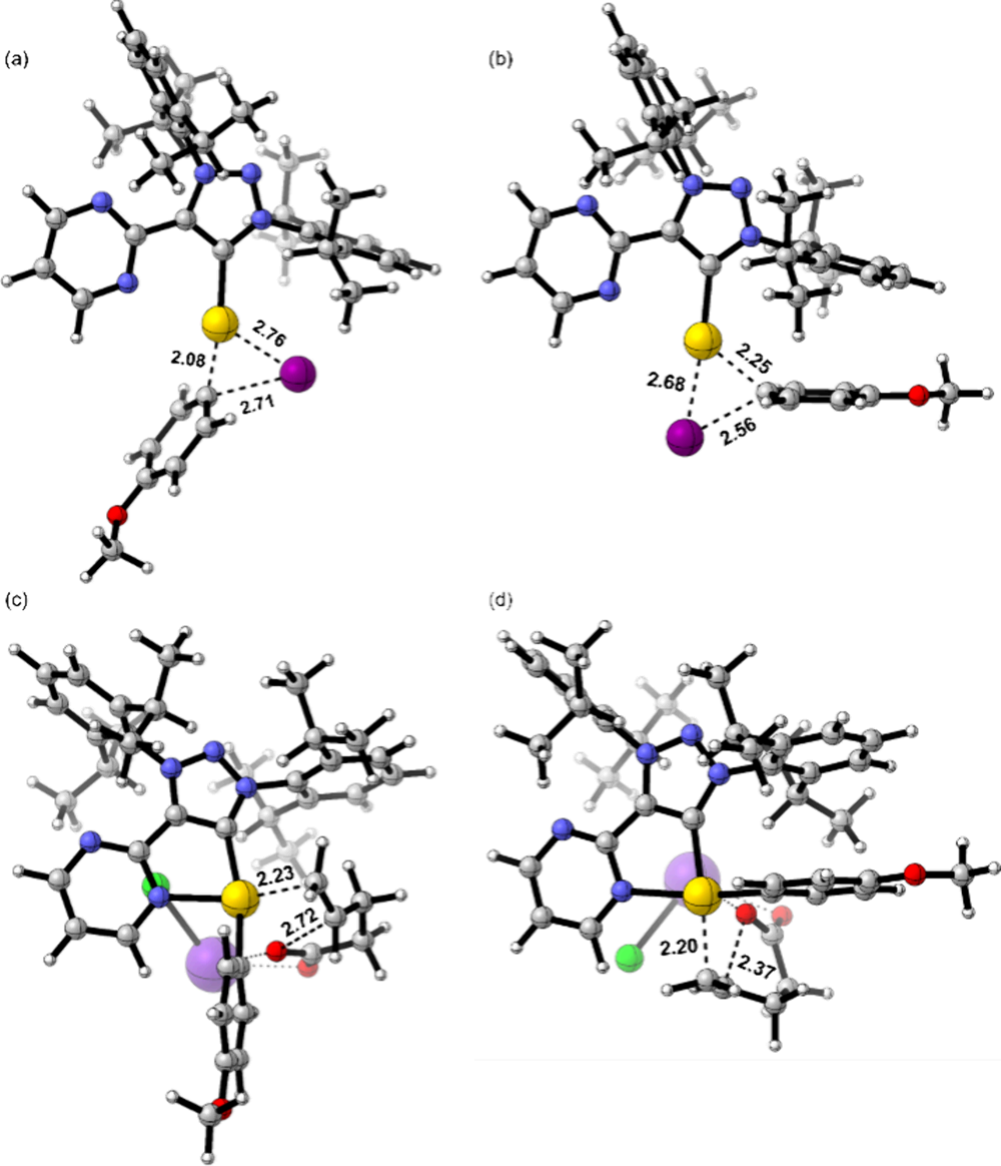
Transition states (a) **TS-AB+trans**, (b) **TS-AB+cis**, (c) **TS-CD+trans**, and (d) **TS-CD+cis**; selected distances in Å. Potassium (K^+^) and chloride (Cl^–^) ions are colored pink and green, respectively.

With this information in hand, we next focused on the experimental reaction scope to explore potential correlations between the observed yields and the computed activation barriers (Figure 3a). The computed activation free energies for the key reaction steps are summarized in Table S1 for substrates 10 through 16. Distinct correlations are observed depending on the nature of the catalyst: the N catalyst featuring a pyrimidine-based ligand and the CH catalyst incorporating a pyridine-based ligand. For clarity, each entry is labeled using the substrate number, followed by the catalyst code (e.g., 10-N refers to substrate 10 with the pyrimidine-ligated N catalyst). Given the complexity of the system under investigation, no clear correlation was observed when considering only a single transition state. However, a good multivariable model is obtained when considering the activation barriers of both rate-determining steps (**TS-AB+cis** and **TS-CD+trans**) alongside the competing decomposition pathway (**TS-decomp+cis**), leading to a good correlation with the experimental yields (*R* ^2^ = 0.919, *p* = 0.039; see Figure 3b). Moreover, in an effort to simplify the model, we evaluated the difference in activation barriers between the *cis* oxidative addition of aryl iodides to the Au­(I) complex and the competing *cis* decomposition pathway. This simplified descriptor together with the activation energy of the rds of the *trans* mechanism also yielded a strong correlation with the experimental yields (*R* ^2^ = 0.893, *p* = 0.011; see Figure S2), underscoring the critical role of the energetic balance between the rate-determining step of the productive *cis* pathway and its associated decomposition process.

**Figure 3:**
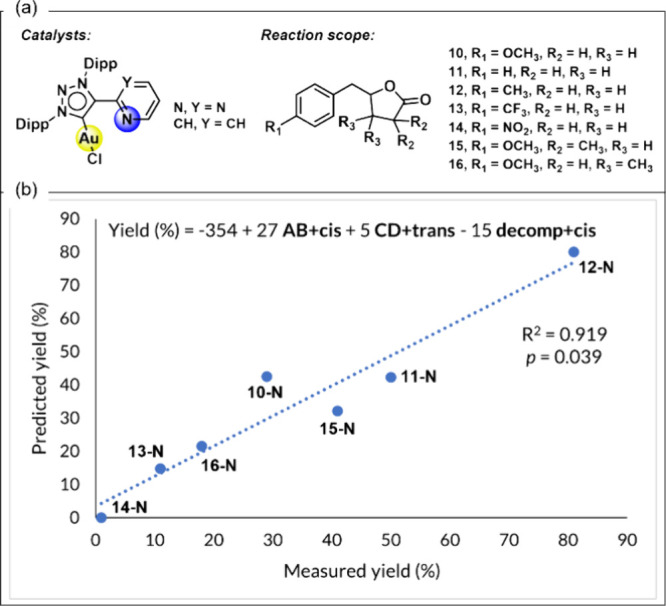
(a) Investigated experimental scope for the Au­(I)/Au­(III)-mediated arylation-lactonization reaction. (b) Three-variable model for the prediction of the experimental yield with catalyst **N** considering the kinetic cost of **TS-CD+trans**, **TS-AB+cis**, and **TS-decomp+cis**.

The term **AB+cis** corresponds to the oxidative addition of the aryl iodide to the Au­(I) complex via the *cis* pathway. The positive coefficient in the regression suggests that within the studied range, slightly higher activation barriers at this step are associated with improved yields. This counterintuitive trend may reflect a kinetic selectivity filter: substrates undergoing slower oxidative addition could avoid premature or unproductive binding events, thereby favoring a more controlled and productive catalytic turnover. The **CD+trans** term, associated with the intramolecular γ-lactonization step along the *trans* pathway, also exhibits a positive, albeit smaller, contribution to the yield. Although this step is rate-determining for the *trans* mechanism, it is generally less favorable due to its higher barrier and the reversibility of the preceding oxidative addition. Nevertheless, for certain substrates where this barrier is lowered, partial engagement of the *trans* pathway may still contribute to product formation. In contrast, the **decomp+cis** term represents the decomposition pathway via reductive elimination from the *cis*-Au­(III) intermediate. Its negative coefficient aligns with mechanistic expectations: lower decomposition barriers facilitate undesired side reactions, ultimately reducing the efficiency of the catalytic cycle. Overall, these results highlight that high-yielding substrates strike an optimal balance between effective oxidative addition and lactonization steps while simultaneously suppressing deleterious decomposition pathways, a conclusion that both supports and quantifies the mechanistic scenario derived from DFT calculations.

It is noted that the yields obtained with both catalysts bearing pyridine (CH) and pyrimidine (N) ligands were considered. The experimental yields obtained are quite similar, and this is consistent with the slightly larger negative charge on the pyrimidine nitrogen (−0.492 e) compared to pyridine (−0.474 e), indicating a marginally stronger electron-donating character for the pyrimidine ligand, and very similar %*V*_Bur_ values of 48.5 and 50.4%, respectively,^54,55^ since this modification does not alter significantly the first sphere around the gold center (see Figure S3). %*V*_Bur_ is an index that unveils how a particular ligand/s affects the metal environment toward the potential interaction with entering substrates.^56^ Multivariable models incorporating two and three variables were also built for the CH catalyst (Figures S4 and S5), yielding similarly strong correlations with the experimental yield.

As part of a predictive catalysis approach,^57^ we designed a series of aryl iodide substrates featuring both electron-donating groups (EDG) and electron-withdrawing groups (EWG) of varying steric demands at the *ortho* and *meta* positions, with the aim of predicting experimental yields using the developed model (see Figure S6). As shown in Table S2, *ortho*-substitution consistently results in predicted yields of 0% for all substituents, with the sole exception of the methoxy group, which affords a modest yield of 12%. In contrast, *meta*-substitution reveals a clear electronic effect: electron-donating groups (e.g., OCH_3_ and CH_3_) lead to low predicted yields of 1 and 3%, respectively, whereas electron-withdrawing groups significantly enhance the predicted yield, reaching 20% for CF_3_ and 55% for NO_2_.

We also considered modifying the hemilabile mesoionic carbene (MIC^N) ligands. Analysis of the reaction mechanism reveals that one of the most critical steps is the oxidative addition of aryl iodides to the Au­(I) complex. This step is pivotal because, in the *trans* mechanism, the decomposition pathway is energetically inaccessible, whereas in the *cis* mechanism, the decomposition pathway is isoenergetic with the formation of the desired γ-lactonization product. We hypothesized that modifying the catalyst to stabilize **TS-AB+trans** relative to **TS-AB+cis** while also increasing the energy gap between these two transition states could enhance the yield of the desired product. This approach is based on the premise that decomposition pathways can be effectively ruled out due to their significantly higher activation energies.

To test this hypothesis, we explored the impact of steric effects on promoting the *trans* addition over the *cis* addition by substituting the diisopropyl (dipp) ligands. Considering the transition state structures depicted in Figure 2 and the size disparity between the aryl and iodide substituents, we replaced dipp with hydrogen, mesityl, and adamantyl substituents. We initially envisioned that smaller substituents (e.g., H) would favor the *cis* addition, while bulkier substituents (e.g., mesityl and adamantyl) would favor the targeted *trans* addition. However, the computed activation energies for **TS-AB+trans** and **TS-AB+cis** reveal that steric effects alone are not solely responsible for pathway selection. The differences in kinetic barriers between the two possible transition states for the oxidative addition step are 1.4, 0.5, and 1.4 kcal·mol^‑1^ for hydrogen, mesityl, and adamantyl, respectively, with the *trans* transition state consistently being lower in energy than the *cis* (see Figure S7 and Table S3). These findings suggest that in addition to steric effects, the well-known *trans* effect likely plays a significant role in determining the observed reactivity.

## Conclusions

This study provides a detailed mechanistic investigation into the gold-catalyzed arylation-lactonization of γ-alkenoic acids by using DFT calculations. We identified two possible mechanistic pathways, *cis* and *trans*, each with distinct rate-determining steps. While the *trans* pathway features an energetically inaccessible decomposition step, the *cis* pathway faces competition between product formation and decomposition, with activation barriers that are nearly identical. Through quantitative analysis of computed transition state energies, we established multivariable models that reliably correlate with experimental yields. These models successfully rationalize the substrate scope and predict reactivity trends based on electronic and steric parameters. Furthermore, ligand modification studies underscore the nuanced role of steric and *trans* effects in directing the oxidative addition selectivity. Collectively, these insights lay the groundwork for rational design of next-generation gold catalysts and advance the broader application of redox-active gold systems in homogeneous catalysis.

## Computational Details

All geometry optimizations and analytical vibrational frequency calculations were carried out using the Gaussian16 software package (revision A.03).^58^ The B3LYP hybrid exchange–correlation,^59–61^ along with the Grimme D3 correction, was employed for electronic energy calculations.^62,63^ The molecular electronic configuration was described using the SDD basis set and pseudopotentials for Au and I,^64–66^ while the Def2-SVP basis set, including the polarization of Ahlrichs and co-workers, was used for all other atoms.^67^ The Intrinsic Reaction Coordinate (IRC) procedure was used to confirm the two minima connected by each transition state.^68^ To improve accuracy, single-point energy calculations were carried out again with the B3LYP hybrid exchange–correlation functional^59^ and the Def2-TZVP for nonmetal atoms.^69^ Moreover, solvent effect corrections were included to simulate a 2,2,2-trifluoroethanol solution by means of the universal solvation model SMD of Cramer et al.^70^ As a summary, the reported Gibbs energies are obtained at the B3LYP-D3/Def2-TZVP∼SDD-SMD­(2,2,2-trifluoroethanol)//B3LYP-D3/Def2-SVP∼SDD level of theory together with gas-phase thermal and entropic contributions computed at 298 K and 1 atm at the B3LYP-D3/Def2-SVP∼SDD level of theory.

## Supplementary Material



## Notes

Additional information about the mechanistic modeling, steric analysis, and statistical modeling to rationalize and predict catalytic performance; all *xyz* coordinates and absolute energies of all computed species (PDF)
