# Supplementary material for: Mechanism of Gold-Catalyzed Arylation-Lactonization: A Density Functional Theory Study on the Role of the (MIC^N)AuCl Complex in Au(I)/Au(III) Catalysis
Source: Inorg Chem. 2025 Jun 17;64(25):12755–61. doi: 10.1021/acs.inorgchem.5c01666 (PMC12216232; doi:10.1021/acs.inorgchem.5c01666)
Supplement: Supplementary file 1 [file ic5c01666_si_001.pdf]

Mechanism of Gold-Catalyzed Arylation-Lactonization: A DFT Study on the Role of (MIC^N)AuCl Complex in Au(I)/Au(III) Catalysis

Roger Monreal-Corona, Xavi Ribas,* Anna Pla-Quintana,* Albert Poater*

Institut de Química Computacional i Catàlisi (IQCC) and Departament de Química, Universitat de Girona (UdG), Facultat de Ciències, C/ Maria Aurèlia Capmany, 69, 17003-Girona, Catalunya, Spain.

Corresponding authors: xavi.ribas@udg.edu; anna.plaq@udg.edu; albert.poater@udg.edu

Electronic Supplementary Information

Contents:

Table S1. Summary of experimental yields and activation energies (in kcal·mol^{-1}) of **TS-AB+trans**, **TS-AB+cis**, **TS-CD+trans**, **TS-CD+cis**, and **TS-decomp+cis** for the experimental scope. The effect of the catalysts bearing pyrimidine (N) and pyridine (CH) ligands was also considered.

Figure S1. Structures and distortion-interaction analysis of the oxidative addition transition states mediated by MIC^N and Dalphos ligands. Energies are in kcal/mol.

Figure S2. 2-variable model for the prediction of the experimental yield with catalyst **N** considering the kinetic cost of **TS-CD+trans**, and the difference between **TS-decomp+cis** and **TS-AB+cis**.

Figure S3. Buried volumes (%V_{Bur}) and steric maps of the xy plane of the studied catalysts pyridine (CH) and pyrimidine (N) ligands, featuring the external ring, with a radius of 7.0 Å from the center of the ring, the length of the corresponding CNT the z axis, and any of the carbon atoms providing the xz plane; curves are indicated in Å.

Figure S4. 3-variable model for the prediction of the experimental yield with catalyst **CH** considering the kinetic cost of **TS-CD+trans**, **TS-AB+cis** and **TS-decomp+cis**.

Figure S5. 2-variable model for the prediction of the experimental yield with catalyst **CH** considering the kinetic cost of **TS-CD+trans**, and the difference between **TS-decomp+cis** and **TS-AB+cis**.

Figure S6. Designed structures to be evaluated with the 2-variable model for the prediction of the experimental yield.

Table S2. Summary of calculated activation energies (in kcal·mol^{-1}) of **TS-AB+cis**, **TS-CD+trans**, and **TS-decomp+cis** together with the predicted experimental yield (in %) obtained with the 2- and 3-variable model.

Figure S7. Model systems studied to explore the impact of steric effects on promoting the *trans* addition over the *cis* addition by substituting the diisopropyl (dipp) ligands.

Table S3. Activation energies (in kcal·mol^{-1}) of **TS-AB+trans** and **TS-AB+cis** for the modified catalysts.

Cartesian coordinates of the optimized geometries at the B3LYP-D3/Def2-SVP~SDD level of theory.

Table S1. Summary of experimental yields and activation energies (in kcal·mol^{-1}) of **TS-AB+trans**, **TS-AB+cis**, **TS-CD+trans**, **TS-CD+cis**, and **TS-decomp+cis** (see Figure 1, main text) for the experimental scope. The effect of the catalysts bearing pyrimidine (N) and pyridine (CH) ligands was also considered.

Species	Yield (%)	TS-AB+trans	TS-AB+cis	TS-CD+trans	TS-CD+cis	TS-decomp+cis
**10-N**	29	19.3	20.5	25.3	17.3	19.4
**11-N**	50	23.6	20.2	25.6	17.3	19.0
**12-N**	81	21.8	21.4	26.2	17.1	18.8
**13-N**	11	22.7	20.5	23.2	16.9	20.6
**14-N**	1	23.3	20.7	24.7	17.4	22.6
**15-N**	41	19.3	20.5	23.2	13.9	19.4
**16-N**	18	19.3	20.5	22.7	15.5	19.4
**10-CH**	35	18.9	20.6	22.3	17.4	21.1
**11-CH**	31	20.4	21.5	22.2	17.3	22.1
**12-CH**	60	19.3	21.5	22.3	17.1	21.0
**13-CH**	13	20.1	20.9	19.8	16.9	23.1
**14-CH**	1	19.5	19.1	20.2	17.3	25.4
**15-CH**	37	18.9	20.6	20.7	14.1	21.1
**16-CH**	32	18.9	20.6	20.4	15.8	21.1

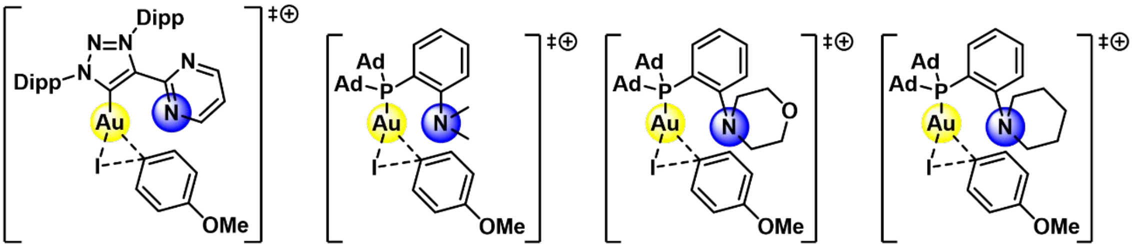

$\Delta E^{‡}$ = 18.6	$\Delta E^{‡}$ = 17.8	$\Delta E^{‡}$ = 24.6	$\Delta E^{‡}$ = 23.1
$\Delta E_{\text{dist-cat}}$ = 4.7	$\Delta E_{\text{dist-cat}}$ = 1.1	$\Delta E_{\text{dist-cat}}$ = 6.8	$\Delta E_{\text{dist-cat}}$ = 2.0
$\Delta E_{\text{dist-sub}}$ = 21.7	$\Delta E_{\text{dist-sub}}$ = 17.3	$\Delta E_{\text{dist-sub}}$ = 22.8	$\Delta E_{\text{dist-sub}}$ = 25.1
ΔE_{int} = -6.2	ΔE_{int} = -0.6	ΔE_{int} = -5.0	ΔE_{int} = -4.0

Figure S1. Structures and distortion-interaction analysis of the oxidative addition transition states mediated by MIC^N and Dalphos ligands.[48] Energies are in kcal/mol.

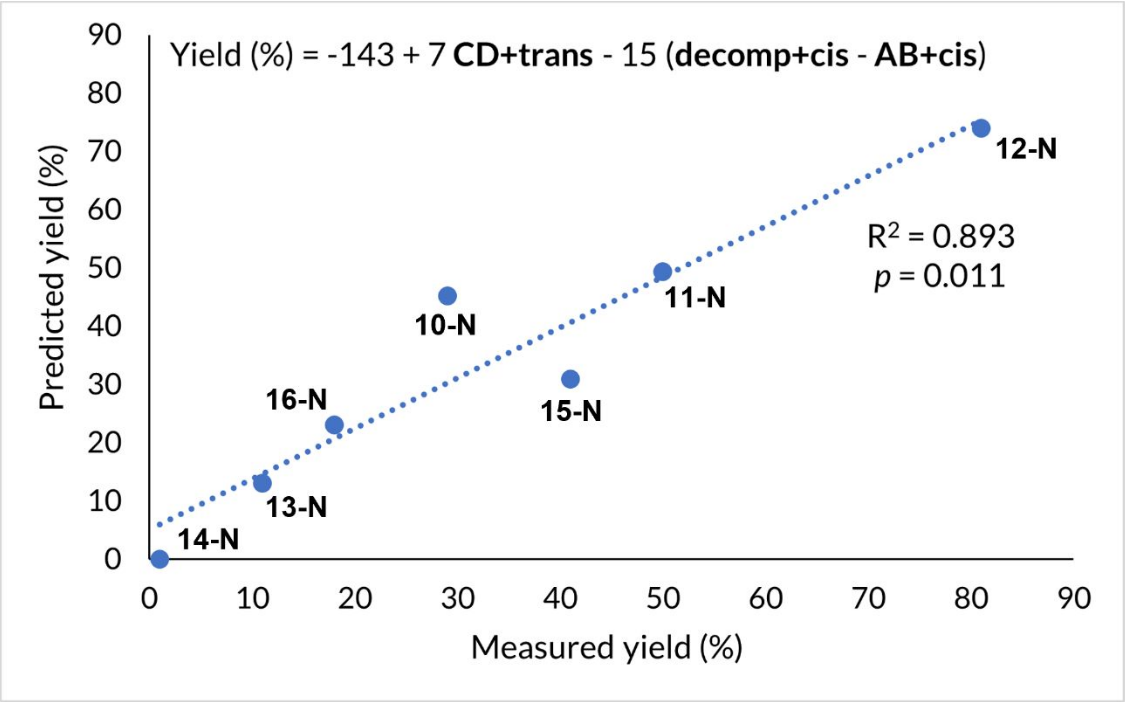

Figure S2. 2-variable model for the prediction of the experimental yield with catalyst **N** considering the kinetic cost of **TS-CD+trans**, and the difference between **TS-decomp+cis** and **TS-AB+cis** (see Figure 1, main text).

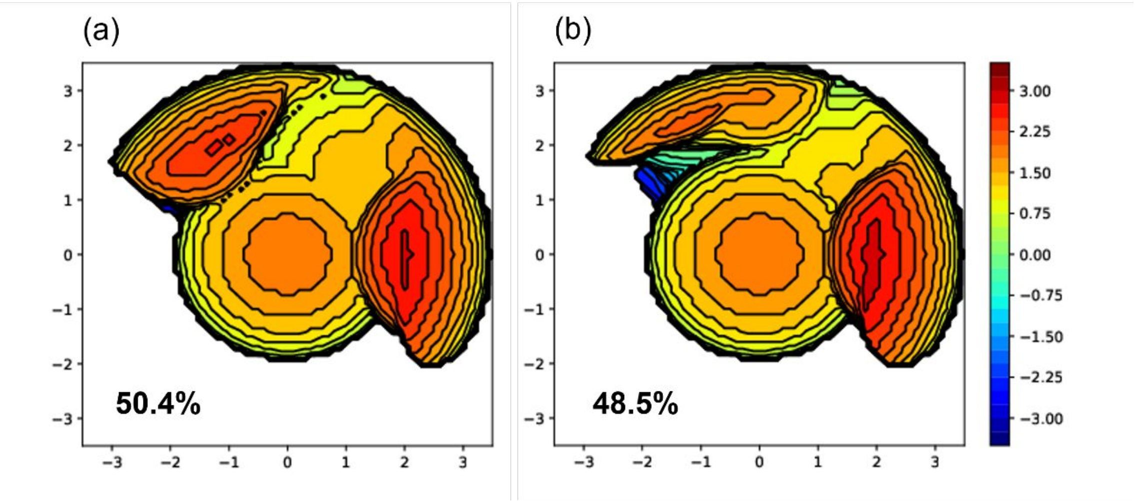

Figure S3. Buried volume (%V_{Bur}) and steric maps of the xy plane of the studied catalysts bearing (a) pyridine (CH) and (b) pyrimidine (N) ligands, featuring the external ring, with a radius of 3.5 Å from the center of the ring, the length of the corresponding CNT the z axis, and any of the carbon atoms providing the xz plane; curves are indicated in Å.

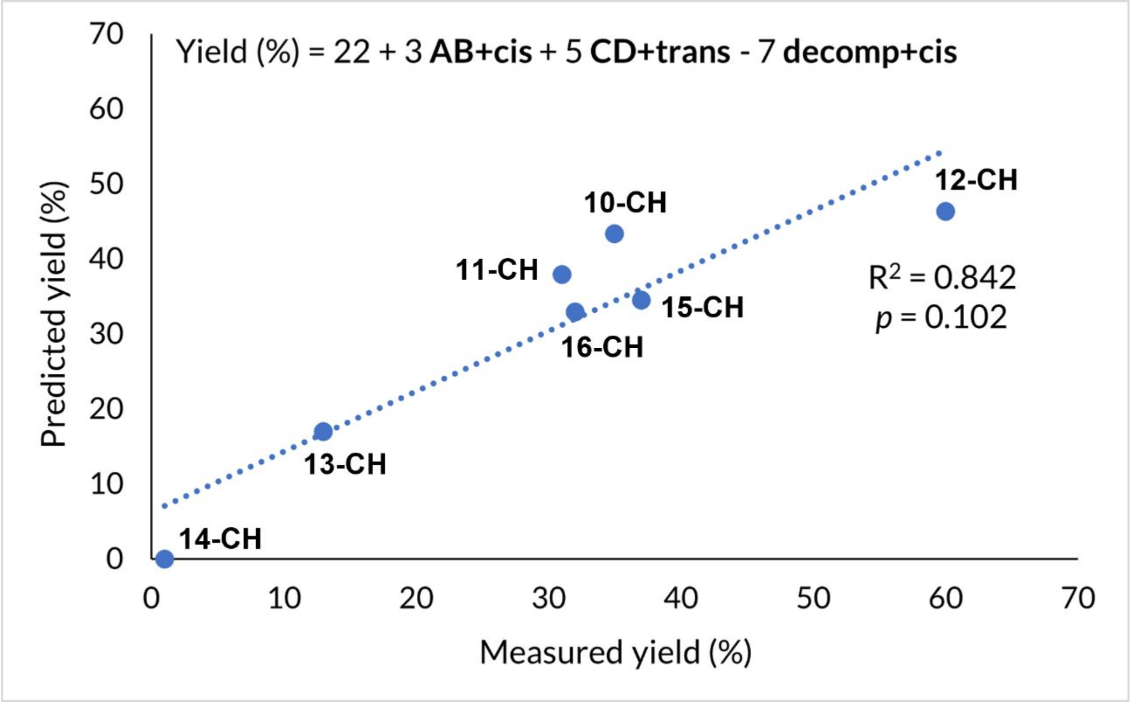

Figure S4. 3-variable model for the prediction of the experimental yield with catalyst **CH** considering the kinetic cost of **TS-CD+trans**, **TS-AB+cis** and **TS-decomp+cis**.

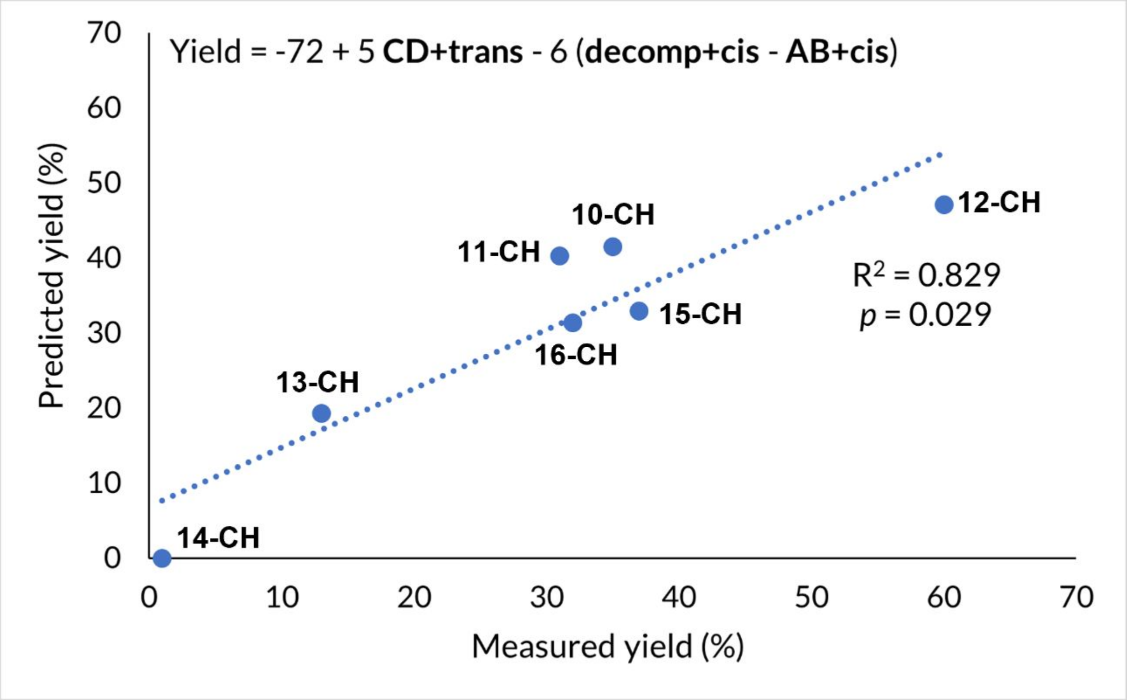

Figure S5. 2-variable model for the prediction of the experimental yield with catalyst **CH** considering the kinetic cost of **TS-CD+trans**, and the difference between **TS-decomp+cis** and **TS-AB+cis**.

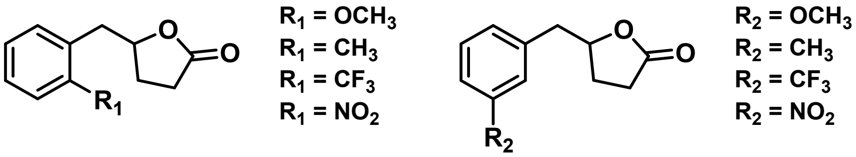

Figure S6. Designed structures to be evaluated with the 2-variable model for the prediction of the experimental yield.

Table S2. Summary of calculated activation energies (in kcal·mol^{-1}) of **TS-AB+cis**, **TS-CD+trans**, and **TS-decomp+cis** for the **N** catalyst, together with the predicted experimental yield (in %) obtained with the 2- (Figure 3b, main text) and 3-variable (Figure S1) model.

Products expected	TS-AB+cis	TS-CD+trans	TS-decomp+cis	Yield 2-var[a]	Yield 3-var[a]
**o-$OCH_{3\text{-}N}$**	20.0	19.8	23.0	16	12
**o-CH_3-N**	17.4	22.7	24.7	0	0
**o-CF_3-N**	21.6	29.3	21.2	0	0
**o-NO_2-N**	16.5	29.3	19.2	0	0
**m-OCH_3-N**	19.4	20.2	25.0	15	1
**m-CH_3-N**	19.3	19.4	23.9	18	3
**m-CF_3-N**	20.5	20.2	23.3	19	20
**m-NO_2-N**	22.4	21.9	24.7	32	55

[a]Note that the predicted yields were set to 0% whenever the obtained values were negative.

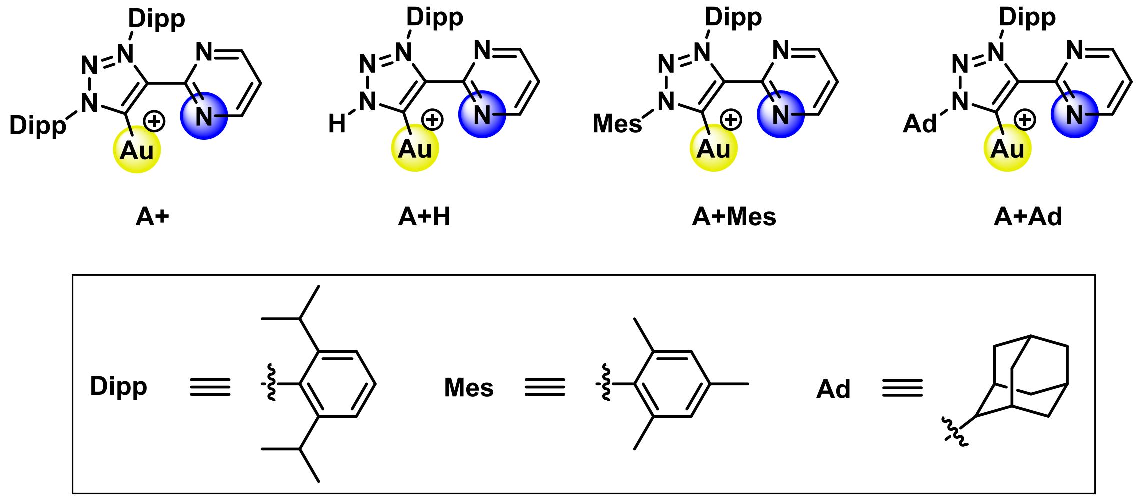

Figure S7. Model systems studied to explore the impact of steric effects on promoting the *trans* addition over the *cis* addition by substituting the diisopropyl (dipp) ligands.

Table S3. Activation energies (in kcal·mol^{-1}) of **TS-AB+trans** and **TS-AB+cis** for the modified catalysts.

Species	$\Delta G^{\ddagger}_{TS\text{-}AB+trans}$	$\Delta G^{\ddagger}_{TS\text{-}AB+cis}$	$\Delta\Delta G^{\ddagger}$
A+	19.3	20.5	1.2
A+H	19.4	20.8	1.4
A+Mes	18.8	19.3	0.5
A+Ada	18.6	20.0	1.4

Cartesian coordinates and absolute energies (in kcal·mol^{-1}) of the optimized geometries at the B3LYP-D3/Def2-SVP~SDD level of theory.

74

IIb SCF Done: -2034.20798119 A.U.

C	-0.911493	0.712532	-0.028670
C	0.447404	0.374159	-0.018365
N	-0.822751	-1.513181	0.019148
N	0.396739	-1.001106	0.016075
N	-1.614506	-0.468278	0.000755
C	1.529246	-1.893163	0.064871
C	2.098421	-2.310556	-1.151020
C	2.025189	-2.259931	1.328490
C	3.216887	-3.151054	-1.070774
C	3.144705	-3.102346	1.349456
C	3.732748	-3.543930	0.164175
H	3.697574	-3.493183	-1.989569
H	3.569418	-3.406299	2.308440
H	4.609561	-4.195097	0.203390
C	-3.041241	-0.687219	-0.072806
C	-3.620340	-0.794971	-1.348447
C	-3.757675	-0.785375	1.131775
C	-5.002178	-1.030599	-1.394081
C	-5.132856	-1.025797	1.028396
C	-5.746430	-1.147061	-0.220217
H	-5.501352	-1.128611	-2.359421
H	-5.735166	-1.114776	1.933851
H	-6.821879	-1.333270	-0.278346
C	-2.778453	-0.723854	-2.617174
H	-1.857490	-0.166454	-2.382956
C	-3.052873	-0.671581	2.475610
H	-2.141340	-0.075459	2.316579
C	1.423553	-1.735375	2.625878
H	0.510095	-1.173735	2.379311
C	1.573446	-1.843319	-2.502320
H	0.640519	-1.284814	-2.333239
C	-2.622465	-2.063160	2.974952
H	-2.059470	-1.980159	3.918198
H	-3.502699	-2.701508	3.156065
H	-1.981779	-2.570081	2.238118
C	-3.882484	0.075982	3.526178
H	-4.777324	-0.491629	3.828403
H	-3.280985	0.239839	4.434060
H	-4.204976	1.056775	3.145213
C	-3.470337	0.032053	-3.759546
H	-2.773216	0.160091	-4.602288
H	-4.346521	-0.514454	-4.143592
H	-3.805636	1.030300	-3.437649
C	-2.357174	-2.139671	-3.053430

H	-3.241826	-2.746038	-3.307648
H	-1.704966	-2.095102	-3.939743
H	-1.809767	-2.655742	-2.251064
C	1.228608	-3.023597	-3.424141
H	0.791314	-2.656501	-4.366478
H	2.124306	-3.609703	-3.684791
H	0.503535	-3.704636	-2.952389
C	2.570027	-0.876019	-3.165279
H	3.521417	-1.384343	-3.391517
H	2.159132	-0.489291	-4.111970
H	2.792127	-0.020250	-2.509481
C	1.006241	-2.875495	3.568046
H	1.876112	-3.461295	3.905743
H	0.515197	-2.467828	4.466234
H	0.302883	-3.565651	3.077006
C	2.389914	-0.752829	3.310730
H	1.926634	-0.324178	4.214241
H	3.319037	-1.261065	3.615795
H	2.662531	0.072997	2.635641
C	-1.530836	2.047798	-0.020852
N	-0.857892	3.018104	-0.650118
N	-2.692542	2.190839	0.631456
C	-1.384804	4.240171	-0.606986
C	-3.222385	3.414658	0.651610
C	-2.595501	4.503603	0.042659
H	-0.823629	5.034645	-1.111452
H	-4.175563	3.529628	1.180161
H	-3.026123	5.505911	0.072258
Au	2.126601	1.467398	0.004947
Cl	4.089666	2.714560	0.051797

1
Ag+ SCF Done: -146.700744720 A.U.

Ag	0.000000	0.000000	0.000000

2
AgCl SCF Done: -607.110420970 A.U.

Cl	0.000000	0.000000	-1.704143
Ag	0.000000	0.000000	0.616392

73
A+ SCF Done: -1573.85454278 A.U.

C	0.843568	-0.728815	-0.031228
C	-0.543533	-0.615385	-0.034967
N	0.335756	1.440994	0.036068
N	-0.772284	0.731153	0.003595
N	1.311008	0.564704	0.013129
C	-2.056128	1.397541	-0.001625
C	-2.615982	1.727712	-1.249395

C	-2.675002	1.642958	1.237552
C	-3.882320	2.327655	-1.227054
C	-3.940103	2.244812	1.195680
C	-4.536542	2.579992	-0.020502
H	-4.363690	2.600937	-2.167992
H	-4.465918	2.454725	2.129090
H	-5.523694	3.048071	-0.028016
C	2.671284	1.066296	0.082541
C	3.324962	1.352609	-1.127108
C	3.230677	1.248809	1.357425
C	4.619671	1.874752	-1.026384
C	4.530973	1.770980	1.397010
C	5.213563	2.081212	0.220597
H	5.174656	2.122326	-1.932336
H	5.015089	1.941560	2.359695
H	6.225205	2.490231	0.275420
C	2.641421	1.139820	-2.471062
H	1.863000	0.371104	-2.331673
C	2.449399	0.951019	2.631703
H	1.650599	0.232251	2.382175
C	-2.036260	1.262260	2.567718
H	-1.008839	0.917114	2.373424
C	-1.915977	1.431357	-2.570302
H	-0.894500	1.083161	-2.352390
C	1.767278	2.231398	3.149289
H	1.162774	2.015464	4.044122
H	2.520368	2.987978	3.421012
H	1.108843	2.672357	2.385789
C	3.306471	0.291162	3.720917
H	4.067054	0.981299	4.117747
H	2.672680	-0.012254	4.568559
H	3.824485	-0.602654	3.340093
C	3.592542	0.605410	-3.549843
H	3.027130	0.350433	-4.459560
H	4.347663	1.352973	-3.838683
H	4.118496	-0.297958	-3.205040
C	1.938659	2.432632	-2.926559
H	2.674243	3.236422	-3.088718
H	1.398716	2.269082	-3.872536
H	1.217223	2.786171	-2.174432
C	-1.776285	2.690667	-3.440512
H	-1.201580	2.461404	-4.351271
H	-2.757463	3.075839	-3.758800
H	-1.254676	3.494997	-2.900128
C	-2.633433	0.298588	-3.325238
H	-3.663354	0.587135	-3.588517
H	-2.101313	0.056883	-4.258774
H	-2.687343	-0.617631	-2.714578
C	-1.927264	2.468408	3.514636

H	-2.919534	2.845125	3.807600
H	-1.398806	2.181573	4.436991
H	-1.374390	3.296720	3.046590
C	-2.794289	0.096213	3.225907
H	-2.302898	-0.205159	4.164356
H	-3.831496	0.380447	3.463973
H	-2.832015	-0.783247	2.562154
C	1.679977	-1.944967	-0.074047
N	1.047606	-3.103707	0.151849
N	2.977720	-1.796604	-0.342234
C	1.792549	-4.209668	0.114447
C	3.719713	-2.905777	-0.372602
C	3.166551	-4.167526	-0.142745
H	1.278185	-5.159022	0.297010
H	4.785446	-2.780165	-0.591487
H	3.772577	-5.074586	-0.167123
Au	-1.956435	-2.018603	-0.068052

16
PhOMeI SCF Done: -357.343005094 A.U.

C	2.605215	0.271978	0.000145
C	1.807857	1.430227	0.000058
C	0.419340	1.335707	-0.000028
C	-0.188655	0.074244	0.000022
C	0.588764	-1.081822	0.000166
C	1.986465	-0.987631	0.000249
H	2.304960	2.402271	0.000018
H	-0.183498	2.245678	-0.000114
H	0.121202	-2.068237	0.000254
H	2.573446	-1.906004	0.000423
O	3.945189	0.472078	0.000224
C	4.811270	-0.641806	-0.000398
H	4.672757	-1.270431	-0.899215
H	5.834570	-0.243011	-0.000201
H	4.672722	-1.271322	0.897795
I	-2.334702	-0.076810	-0.000038

89
A-ad+cis SCF Done: -1931.25955007 A.U.

Au	0.885051	-1.454042	0.276512
N	-1.998177	-2.964381	-0.227586
N	-0.730195	1.068594	0.073297
N	-1.919375	1.621838	-0.069140
N	-2.760171	0.616934	-0.078461
C	-2.586770	-4.160765	-0.241367
H	-1.949075	-5.021855	-0.468518
C	-3.952200	-4.319079	0.015853
H	-4.430405	-5.299569	-0.005911
C	-4.667707	-3.156736	0.311486

H	-5.738220	-3.187791	0.541089
C	-2.778780	-1.912749	0.053394
C	-2.118207	-0.594008	0.048773
C	-0.759636	-0.299714	0.147400
C	0.439656	1.916014	0.138428
C	0.784793	2.462524	1.386234
C	1.907907	3.301440	1.417433
H	2.212173	3.759310	2.360175
C	2.646884	3.551216	0.262277
H	3.534941	4.184963	0.309487
C	2.287046	2.965507	-0.952367
H	2.892450	3.160110	-1.838204
C	1.168220	2.129045	-1.047641
C	-0.037660	2.189612	2.638506
H	-0.648640	1.291627	2.452853
C	-1.003316	3.357048	2.909440
H	-1.631408	3.145979	3.788941
H	-1.665075	3.532151	2.048183
H	-0.445687	4.287251	3.103701
C	0.836190	1.879025	3.862339
H	1.418168	2.755943	4.186414
H	1.541889	1.059514	3.652554
H	0.204422	1.577586	4.712233
C	0.729555	1.518208	-2.372754
H	0.197329	0.580317	-2.147529
C	1.906807	1.141188	-3.281027
H	2.639612	0.518079	-2.745844
H	2.431798	2.030186	-3.665207
H	1.543560	0.578692	-4.154912
C	-0.261863	2.453222	-3.091096
H	-1.131732	2.681980	-2.457230
H	-0.627529	1.991009	-4.021498
H	0.223996	3.407317	-3.351001
C	-4.163674	0.897716	-0.304785
C	-4.962838	1.233709	0.800236
C	-6.317276	1.481067	0.541881
H	-6.981487	1.742759	1.367982
C	-6.830804	1.392432	-0.752286
H	-7.891582	1.585705	-0.928935
C	-5.999146	1.066816	-1.823868
H	-6.415822	1.010766	-2.831463
C	-4.634705	0.815332	-1.626957
C	-4.418682	1.305432	2.220960
H	-3.329966	1.144347	2.183010
C	-4.645457	2.692459	2.843615
H	-4.186513	2.742749	3.843411
H	-5.718562	2.910052	2.962172
H	-4.205835	3.488280	2.223623
C	-5.014223	0.187541	3.094241

H	-4.830917	-0.799505	2.645400
H	-6.103038	0.311920	3.208380
H	-4.568325	0.206328	4.101491
C	-3.734914	0.450740	-2.802697
H	-2.691783	0.430468	-2.450513
C	-4.066380	-0.955208	-3.334135
H	-3.985933	-1.716311	-2.543190
H	-3.380363	-1.233039	-4.149946
H	-5.093946	-0.994227	-3.729379
C	-3.798781	1.503301	-3.921063
H	-3.554093	2.506548	-3.540653
H	-4.798832	1.549289	-4.379567
H	-3.082005	1.254859	-4.719521
N	-4.083262	-1.957406	0.335509
I	3.286716	-2.748022	0.376004
C	4.301108	-0.849971	0.114732
C	4.354838	0.034041	1.196497
C	4.817683	-0.521360	-1.132788
H	3.944097	-0.232467	2.171515
C	4.929325	1.283888	1.005198
C	5.393015	0.742141	-1.318422
H	4.771712	-1.219257	-1.970594
H	4.978332	2.005945	1.821093
C	5.435401	1.657532	-0.254544
H	5.787050	0.997327	-2.301541
O	5.912084	2.914518	-0.344870
C	6.469752	3.369805	-1.566604
H	6.801050	4.400901	-1.390677
H	5.722097	3.364313	-2.379874
H	7.337718	2.758305	-1.868134

89

A-ad+trans SCF Done: -1931.25556673 A.U.

Au	-1.125868	-1.102535	0.188595
N	-0.832699	2.133206	0.141314
N	1.881449	-1.112384	-0.119695
N	2.985526	-0.393407	-0.175486
N	2.594095	0.842611	0.015458
C	-1.528229	3.252021	0.347507
H	-2.599240	3.210790	0.122717
C	-0.924493	4.422200	0.818250
H	-1.495311	5.338682	0.975415
C	0.445139	4.353300	1.083014
H	0.997220	5.218546	1.465564
C	0.477213	2.175959	0.420168
C	1.231810	0.930081	0.188719
C	0.748826	-0.373787	0.094893
C	1.946046	-2.544288	-0.306340
C	2.075031	-3.352242	0.837966

C	2.116198	-4.736528	0.624568
H	2.221104	-5.411852	1.474673
C	2.025632	-5.265564	-0.664133
H	2.062539	-6.348240	-0.806799
C	1.885902	-4.428584	-1.772140
H	1.813653	-4.866041	-2.768872
C	1.837248	-3.036257	-1.619523
C	2.217403	-2.743740	2.227654
H	1.703048	-1.768689	2.218096
C	3.702606	-2.482449	2.543371
H	3.810513	-1.988848	3.522127
H	4.170130	-1.840930	1.782075
H	4.263555	-3.430141	2.575356
C	1.552051	-3.580039	3.328237
H	2.075521	-4.534856	3.492995
H	0.501451	-3.804289	3.085949
H	1.574883	-3.031746	4.282684
C	1.724125	-2.099267	-2.815944
H	1.285353	-1.152705	-2.459644
C	0.789075	-2.629008	-3.911289
H	-0.199135	-2.894773	-3.504182
H	1.202251	-3.519223	-4.410721
H	0.646581	-1.861638	-4.688014
C	3.122846	-1.779719	-3.376986
H	3.773523	-1.340033	-2.606212
H	3.051457	-1.068188	-4.214357
H	3.609473	-2.695943	-3.747761
C	3.581860	1.899198	-0.067778
C	4.316055	2.215544	1.086805
C	5.251677	3.251656	0.970594
H	5.847770	3.535652	1.840212
C	5.428997	3.928353	-0.236439
H	6.161326	4.736551	-0.302285
C	4.685014	3.575818	-1.362749
H	4.843582	4.110141	-2.301479
C	3.741809	2.540812	-1.308785
C	4.113298	1.498313	2.414704
H	3.356481	0.711451	2.271481
C	5.403235	0.801609	2.877643
H	5.221796	0.234797	3.804202
H	6.201118	1.531821	3.085818
H	5.777225	0.101715	2.114989
C	3.569673	2.459628	3.485351
H	2.640290	2.938580	3.144029
H	4.298104	3.253648	3.715150
H	3.363561	1.916013	4.421131
C	2.926582	2.165953	-2.541626
H	2.381150	1.233656	-2.326910
C	1.879765	3.249297	-2.858015

H	1.210883	3.429699	-2.002853
H	1.262736	2.952132	-3.720860
H	2.367871	4.205576	-3.105018
C	3.820584	1.880001	-3.758689
H	4.568781	1.105853	-3.532256
H	4.355423	2.782742	-4.092400
H	3.209549	1.529510	-4.605379
N	1.143863	3.232704	0.891776
I	-3.709955	-1.957834	0.258530
C	-4.540331	0.025187	-0.004225
C	-4.098507	0.809006	-1.073990
C	-5.495395	0.482940	0.896919
H	-3.342872	0.445351	-1.771764
C	-4.633859	2.082659	-1.233214
C	-6.032261	1.765492	0.730155
H	-5.832269	-0.132866	1.733019
H	-4.318984	2.722452	-2.059803
C	-5.602890	2.575567	-0.335552
H	-6.781078	2.115166	1.440164
O	-6.042870	3.823629	-0.574510
C	-7.056833	4.384309	0.244024
H	-7.262835	5.383370	-0.160168
H	-6.724887	4.480520	1.292977
H	-7.982189	3.783321	0.209611

89

TS-AB+cis SCF Done: -1931.23370654 A.U.

Au	-1.133739	1.374573	-0.015297
N	1.348325	2.870744	-0.185590
N	0.624545	-1.218719	0.113776
N	1.872334	-1.658135	0.105321
N	2.613386	-0.582271	0.006809
C	1.717339	4.146605	-0.278608
H	0.915299	4.892214	-0.303761
C	3.065452	4.515047	-0.340850
H	3.371064	5.559588	-0.417036
C	3.997876	3.475841	-0.300040
H	5.073914	3.674719	-0.343303
C	2.320919	1.946566	-0.153376
C	1.847615	0.556403	-0.048128
C	0.519634	0.140359	0.021190
C	-0.445152	-2.189350	0.210210
C	-0.912440	-2.532860	1.492685
C	-1.907530	-3.516722	1.555816
H	-2.304699	-3.822537	2.524283
C	-2.400074	-4.115021	0.395891
H	-3.176157	-4.879099	0.472762
C	-1.922280	-3.734669	-0.857720
H	-2.321886	-4.212683	-1.753419

C	-0.929440	-2.753177	-0.983624
C	-0.316172	-1.912408	2.750218
H	0.056385	-0.909688	2.482631
C	0.886677	-2.745120	3.234833
H	1.359830	-2.270983	4.108815
H	1.647758	-2.854726	2.448739
H	0.561030	-3.755465	3.529436
C	-1.338771	-1.717982	3.877524
H	-1.673747	-2.678731	4.298605
H	-2.227385	-1.172186	3.527288
H	-0.886292	-1.145779	4.701936
C	-0.353950	-2.367720	-2.340728
H	0.080544	-1.358840	-2.246509
C	-1.414937	-2.287711	-3.446731
H	-2.247989	-1.631027	-3.155440
H	-1.829447	-3.278283	-3.691413
H	-0.967895	-1.889150	-4.370467
C	0.783865	-3.331085	-2.728690
H	1.573875	-3.349386	-1.963647
H	1.237653	-3.030453	-3.685736
H	0.399236	-4.357364	-2.840776
C	4.054303	-0.734768	-0.030665
C	4.749028	-0.708814	1.190327
C	6.142991	-0.831404	1.120800
H	6.729938	-0.813071	2.041211
C	6.791215	-0.975302	-0.105981
H	7.879503	-1.067274	-0.136292
C	6.060718	-1.010547	-1.294050
H	6.584419	-1.130310	-2.244393
C	4.664650	-0.893281	-1.286607
C	4.051441	-0.526604	2.532707
H	2.963432	-0.568209	2.367148
C	4.393741	-1.659665	3.513117
H	3.822504	-1.541840	4.447193
H	5.462318	-1.656028	3.778872
H	4.154425	-2.645507	3.086684
C	4.370756	0.854173	3.132603
H	4.104914	1.662958	2.435722
H	5.445376	0.947261	3.356898
H	3.816981	1.005368	4.072815
C	3.876089	-0.901558	-2.590705
H	2.803299	-0.952923	-2.346694
C	4.107133	0.402546	-3.374950
H	3.856677	1.284577	-2.766976
H	3.491600	0.420121	-4.288414
H	5.162312	0.496323	-3.677736
C	4.193496	-2.136523	-3.448342
H	4.020979	-3.068658	-2.889644
H	5.239844	-2.134950	-3.791163

H	3.555576	-2.150941	-4.345893
N	3.629093	2.195106	-0.206525
I	-3.135417	3.158836	-0.040634
C	-3.250954	0.603662	0.010196
C	-3.635240	0.083816	1.250154
C	-3.660837	0.044119	-1.196234
H	-3.353954	0.574963	2.181861
C	-4.363819	-1.098911	1.266368
C	-4.396500	-1.145124	-1.165064
H	-3.401707	0.502948	-2.150781
H	-4.651822	-1.563536	2.210837
C	-4.735258	-1.734064	0.064982
H	-4.685866	-1.603036	-2.110239
O	-5.399895	-2.896743	0.197099
C	-5.845429	-3.586151	-0.957771
H	-6.361749	-4.486500	-0.601665
H	-4.998118	-3.884490	-1.600462
H	-6.551006	-2.975707	-1.547694

89

TS-AB+trans SCF Done: -1931.23226038 A.U.

Au	1.462315	-0.187178	-0.072328
N	-0.009883	-2.685841	-0.206887
N	-1.324384	1.227451	0.102490
N	-2.631083	1.033011	0.128862
N	-2.781115	-0.267487	0.044883
C	0.289513	-3.979026	-0.304920
H	1.352594	-4.236339	-0.361950
C	-0.706962	-4.960561	-0.334561
H	-0.464570	-6.021158	-0.415824
C	-2.026873	-4.510500	-0.254526
H	-2.868423	-5.211182	-0.269546
C	-1.311445	-2.353918	-0.137736
C	-1.569624	-0.910914	-0.034174
C	-0.593748	0.078944	0.003293
C	-0.789543	2.568399	0.169054
C	-0.434224	3.071371	1.433329
C	0.102028	4.365053	1.464597
H	0.389748	4.808509	2.418568
C	0.278895	5.095279	0.288305
H	0.696920	6.103667	0.336609
C	-0.062289	4.548739	-0.949297
H	0.098192	5.133073	-1.856291
C	-0.602244	3.258936	-1.040831
C	-0.658027	2.259661	2.703310
H	-0.610700	1.193038	2.428233
C	-2.064450	2.528344	3.271508
H	-2.251890	1.902653	4.158476
H	-2.846974	2.313843	2.528255

H	-2.165476	3.583770	3.570809
C	0.429247	2.484930	3.762105
H	0.382583	3.498033	4.191181
H	1.436089	2.339570	3.339960
H	0.296540	1.776917	4.594946
C	-0.999539	2.647204	-2.378375
H	-0.974940	1.550684	-2.264437
C	-0.020536	2.990859	-3.509208
H	1.017023	2.752012	-3.227852
H	-0.063390	4.057329	-3.780252
H	-0.274767	2.418498	-4.414902
C	-2.443553	3.041586	-2.741021
H	-3.151666	2.738471	-1.955193
H	-2.751341	2.564418	-3.684669
H	-2.526363	4.132864	-2.867555
C	-4.122196	-0.813207	0.047241
C	-4.692058	-1.146824	1.287308
C	-5.982585	-1.691431	1.258089
H	-6.469068	-1.970178	2.194993
C	-6.654060	-1.882153	0.050096
H	-7.659663	-2.309422	0.050937
C	-6.055532	-1.524783	-1.158527
H	-6.598586	-1.675169	-2.093659
C	-4.767186	-0.975018	-1.190548
C	-3.954869	-0.960788	2.607766
H	-3.008499	-0.435009	2.406074
C	-4.748784	-0.080747	3.586200
H	-4.164312	0.094363	4.503051
H	-5.695174	-0.558597	3.884132
H	-4.988414	0.896731	3.140966
C	-3.597208	-2.321699	3.230511
H	-3.013797	-2.938335	2.530061
H	-4.505645	-2.886215	3.494847
H	-3.008181	-2.183681	4.151208
C	-4.107857	-0.610050	-2.515037
H	-3.164874	-0.083510	-2.299478
C	-3.753042	-1.876811	-3.313730
H	-3.116855	-2.554449	-2.724552
H	-3.220521	-1.613819	-4.241578
H	-4.661993	-2.433049	-3.593573
C	-4.972883	0.354756	-3.341146
H	-5.213860	1.264604	-2.771141
H	-5.920416	-0.113262	-3.650616
H	-4.440210	0.655108	-4.257063
N	-2.330110	-3.213068	-0.156460
I	3.188532	1.961348	-0.133575
C	3.465091	-0.739417	-0.144241
C	3.972063	-1.182724	-1.386332
C	4.065361	-1.158420	1.057519

H	3.577592	-0.781363	-2.321865
C	4.955622	-2.157536	-1.409376
C	5.054976	-2.138935	1.034425
H	3.747524	-0.733822	2.011850
H	5.340762	-2.554010	-2.350768
C	5.506663	-2.648919	-0.202072
H	5.480865	-2.485923	1.975555
O	6.461681	-3.574784	-0.335288
C	7.131678	-4.089329	0.807687
H	7.865806	-4.812772	0.432445
H	6.429909	-4.603212	1.487102
H	7.656973	-3.290319	1.357887

89

B+cis SCF Done: -1931.26397035 A.U.

Au	1.062218	-1.433863	-0.113599
O	5.777278	2.528633	0.325881
N	-0.902400	-2.636538	-0.210850
N	-0.531967	1.385854	0.152080
N	-1.805111	1.759564	0.196111
N	-2.504557	0.657984	0.083705
C	-1.070592	-3.957904	-0.319149
H	-0.159819	-4.562319	-0.372568
C	-2.353755	-4.508449	-0.359087
H	-2.501186	-5.585534	-0.447703
C	-3.429187	-3.621078	-0.279802
H	-4.463612	-3.978737	-0.303225
C	-2.009034	-1.854179	-0.142318
C	-1.688989	-0.432144	-0.024903
C	-0.382412	0.037971	0.016188
C	0.483730	2.419687	0.235455
C	1.128555	2.618234	1.472887
C	2.090650	3.634275	1.515924
H	2.634874	3.824401	2.440551
C	2.364358	4.414872	0.391582
H	3.114388	5.206639	0.456609
C	1.697740	4.190372	-0.810853
H	1.930458	4.809287	-1.678482
C	0.742559	3.170985	-0.924252
C	0.748286	1.811941	2.710754
H	0.571848	0.772762	2.383627
C	-0.561725	2.350695	3.322509
H	-0.883151	1.718583	4.165197
H	-1.382805	2.393023	2.593672
H	-0.410930	3.372883	3.703936
C	1.845417	1.761922	3.780781
H	1.985508	2.741856	4.263511
H	2.809695	1.445176	3.361034
H	1.562485	1.051130	4.572204

C	0.008539	2.921275	-2.236656
H	-0.380362	1.888708	-2.214957
C	0.924971	3.020816	-3.465559
H	1.820985	2.390547	-3.360726
H	1.258661	4.054709	-3.643651
H	0.382816	2.698014	-4.367675
C	-1.195064	3.873012	-2.372154
H	-1.893974	3.762716	-1.530426
H	-1.742666	3.674433	-3.306215
H	-0.855011	4.920535	-2.393568
C	-3.952520	0.721801	0.091724
C	-4.606406	0.567212	1.325717
C	-6.006734	0.613232	1.300394
H	-6.563935	0.501582	2.232648
C	-6.697007	0.800658	0.103172
H	-7.789097	0.831613	0.107086
C	-6.006256	0.954844	-1.099853
H	-6.565821	1.103183	-2.024883
C	-4.606400	0.922777	-1.137708
C	-3.860136	0.350108	2.635904
H	-2.778552	0.376500	2.430525
C	-4.146881	1.478454	3.640084
H	-3.549644	1.338038	4.554459
H	-5.207917	1.494164	3.934418
H	-3.899264	2.463843	3.216864
C	-4.174324	-1.033837	3.229907
H	-3.957100	-1.835441	2.507469
H	-5.236272	-1.115913	3.510386
H	-3.575539	-1.208634	4.137662
C	-3.851457	1.046007	-2.456130
H	-2.796030	1.263122	-2.228664
C	-3.895158	-0.286663	-3.226390
H	-3.504196	-1.116932	-2.619101
H	-3.301761	-0.220548	-4.152042
H	-4.930348	-0.541526	-3.504128
C	-4.360927	2.207893	-3.321641
H	-4.343980	3.158882	-2.768772
H	-5.389755	2.035308	-3.673424
H	-3.727763	2.318686	-4.215607
C	2.676929	-0.187654	-0.019045
C	3.453116	-0.133525	1.141646
H	3.258854	-0.797749	1.985025
C	4.498165	0.784115	1.214779
H	5.120633	0.856430	2.108682
C	4.776677	1.642965	0.135927
C	4.014795	1.542853	-1.038648
H	4.211136	2.184587	-1.896712
C	2.960694	0.624551	-1.110731
H	2.366211	0.571511	-2.024295

C	6.157848	3.389052	-0.730471
H	6.990828	3.996159	-0.353227
H	5.330922	4.058709	-1.028622
H	6.498128	2.822461	-1.615517
I	2.688680	-3.534239	-0.303051
N	-3.255943	-2.298136	-0.171378

89

B+trans SCF Done: -1931.24732170 A.U.

Au	1.496201	-0.131219	0.051239
N	0.639215	-2.189032	0.067799
N	-1.531328	1.205268	-0.046266
N	-2.776533	0.746252	-0.060924
N	-2.666126	-0.559346	-0.032752
C	1.335220	-3.329647	0.102151
H	2.424603	-3.234490	0.127873
C	0.672665	-4.558405	0.107484
H	1.228259	-5.496468	0.136491
C	-0.723622	-4.527403	0.074818
H	-1.311074	-5.451267	0.076808
C	-0.722023	-2.252446	0.037148
C	-1.351028	-0.936734	-0.000363
C	-0.591920	0.224599	-0.008568
C	-1.295806	2.632232	-0.064796
C	-1.204152	3.293575	1.171653
C	-0.961271	4.672299	1.124567
H	-0.884117	5.239800	2.052802
C	-0.807198	5.329970	-0.096487
H	-0.617024	6.405792	-0.109435
C	-0.882507	4.629240	-1.301278
H	-0.744230	5.164137	-2.241580
C	-1.124951	3.250028	-1.315739
C	-1.390205	2.553053	2.490315
H	-1.128279	1.495291	2.319408
C	-2.866250	2.597904	2.928431
H	-3.014293	2.023038	3.856288
H	-3.529459	2.181118	2.155131
H	-3.182219	3.636215	3.117092
C	-0.455879	3.057208	3.598360
H	-0.715516	4.077166	3.922010
H	0.593590	3.059493	3.265384
H	-0.537315	2.408272	4.484274
C	-1.230496	2.461826	-2.615631
H	-0.947342	1.418566	-2.395996
C	-0.262701	2.954768	-3.699319
H	0.770282	3.001978	-3.321332
H	-0.538007	3.953645	-4.072164
H	-0.285036	2.272091	-4.563018
C	-2.685914	2.449768	-3.120858

H	-3.374248	2.045169	-2.363335
H	-2.776566	1.836765	-4.031607
H	-3.018790	3.471444	-3.363530
C	-3.855596	-1.383432	-0.035815
C	-4.412972	-1.731758	1.206830
C	-5.546888	-2.554026	1.173332
H	-6.018388	-2.857007	2.110207
C	-6.082399	-2.989544	-0.039457
H	-6.967655	-3.629869	-0.040823
C	-5.502642	-2.609928	-1.250590
H	-5.940048	-2.955694	-2.189236
C	-4.367185	-1.789582	-1.280381
C	-3.813630	-1.277682	2.532354
H	-3.023650	-0.539665	2.321801
C	-4.848699	-0.570234	3.420842
H	-4.368466	-0.192153	4.336757
H	-5.654376	-1.254230	3.729896
H	-5.308101	0.282464	2.898599
C	-3.153389	-2.460809	3.262504
H	-2.399114	-2.951458	2.627952
H	-3.901505	-3.221839	3.536081
H	-2.664470	-2.120881	4.189189
C	-3.721628	-1.394478	-2.602947
H	-2.925461	-0.662080	-2.396036
C	-3.060578	-2.613166	-3.271021
H	-2.334256	-3.092941	-2.596792
H	-2.538264	-2.313800	-4.193475
H	-3.812736	-3.371255	-3.541559
C	-4.719675	-0.703164	-3.545106
H	-5.177811	0.175853	-3.067179
H	-5.528996	-1.384845	-3.849832
H	-4.208714	-0.367347	-4.460902
N	-1.413562	-3.381474	0.039709
C	3.432523	-0.824585	0.118435
C	3.992182	-1.197271	1.351713
C	4.148121	-1.049783	-1.059949
H	3.470928	-0.988662	2.289910
C	5.227306	-1.839615	1.393036
C	5.392229	-1.695165	-1.026165
H	3.751833	-0.723360	-2.024917
H	5.677676	-2.141672	2.340747
C	5.939540	-2.096483	0.204823
H	5.926251	-1.863323	-1.961345
O	7.126854	-2.717816	0.351330
C	7.936590	-2.962988	-0.783577
H	8.847124	-3.452844	-0.415408
H	8.214423	-2.024663	-1.295598
H	7.435466	-3.632616	-1.505695
I	2.480145	2.312913	0.045096

89
TS-decomp+cis SCF Done: -1931.23305988 A.U.

Au	-1.653452	-1.061840	-0.365791
O	-3.078868	4.343733	-2.828705
N	0.586878	-1.385986	-2.577546
N	0.405820	0.762183	1.130811
N	1.711641	0.829915	1.295644
N	2.232049	0.163921	0.297590
C	0.793724	-2.253950	-3.570129
H	0.000798	-2.345274	-4.319447
C	1.966060	-3.010765	-3.649846
H	2.136417	-3.718110	-4.462868
C	2.904137	-2.820628	-2.631504
H	3.847267	-3.376912	-2.613676
C	1.546951	-1.284233	-1.651854
C	1.273475	-0.364959	-0.529010
C	0.047852	0.041414	0.014688
C	-0.451329	1.454495	2.068667
C	-0.542607	2.860033	1.956359
C	-1.416916	3.499981	2.842645
H	-1.528928	4.583772	2.799610
C	-2.153029	2.772795	3.780351
H	-2.833395	3.295544	4.456827
C	-2.015897	1.388886	3.873495
H	-2.578522	0.842596	4.631876
C	-1.147848	0.688480	3.021753
C	0.289602	3.656264	0.955943
H	0.484729	3.001016	0.089767
C	1.643049	4.057068	1.577743
H	2.247578	4.613330	0.845192
H	2.219910	3.184676	1.912234
H	1.480496	4.711056	2.449075
C	-0.423119	4.904739	0.415867
H	-0.513615	5.687122	1.185373
H	-1.427425	4.670683	0.039379
H	0.159448	5.335108	-0.412965
C	-0.914978	-0.804708	3.219242
H	-0.414346	-1.204789	2.324466
C	-2.218509	-1.596053	3.393738
H	-2.921735	-1.406717	2.569287
H	-2.721921	-1.350468	4.341527
H	-2.005737	-2.675391	3.404300
C	0.044215	-1.025427	4.404661
H	0.999082	-0.499357	4.255066
H	0.258124	-2.098691	4.529209
H	-0.401033	-0.656615	5.342317
C	3.676491	0.141698	0.158490
C	4.264665	1.138267	-0.640135

C	5.660049	1.089013	-0.765937
H	6.172997	1.833979	-1.376086
C	6.404291	0.105691	-0.113416
H	7.491426	0.091385	-0.221400
C	5.776887	-0.856198	0.680634
H	6.379578	-1.611783	1.186926
C	4.385343	-0.866897	0.830871
C	3.439475	2.199776	-1.359843
H	2.465254	2.273524	-0.851538
C	4.077143	3.595253	-1.287600
H	3.379154	4.349876	-1.682526
H	4.995720	3.656967	-1.891414
H	4.331673	3.868374	-0.252872
C	3.169453	1.792613	-2.820288
H	2.657721	0.820998	-2.888931
H	4.113723	1.709820	-3.381346
H	2.539294	2.544473	-3.321346
C	3.679456	-1.892163	1.705900
H	2.619594	-1.919131	1.404532
C	4.223648	-3.313805	1.512884
H	4.204593	-3.602008	0.451122
H	3.610084	-4.032964	2.077319
H	5.257315	-3.412043	1.879485
C	3.732560	-1.456680	3.181904
H	3.303820	-0.451862	3.317862
H	4.773529	-1.428367	3.541501
H	3.171692	-2.161537	3.815496
C	-1.634492	0.954065	-0.988683
C	-1.196953	1.306031	-2.273177
H	-0.488431	0.669310	-2.802666
C	-1.708897	2.455460	-2.859246
H	-1.401303	2.760137	-3.861251
C	-2.653339	3.253205	-2.177541
C	-3.070202	2.883384	-0.884244
H	-3.800594	3.475527	-0.334310
C	-2.537061	1.746667	-0.272688
H	-2.857374	1.484068	0.735536
C	-4.046289	5.197514	-2.235153
H	-4.235585	5.998536	-2.960258
H	-3.670332	5.640646	-1.296589
H	-4.988773	4.659585	-2.034734
I	-2.903098	-3.345818	-0.264673
N	2.692689	-1.964923	-1.628996

87

decomp+cis SCF Done: -1784.03099909 A.U.

O	5.423140	-3.153111	-1.125616
N	-1.060025	-3.166279	-0.135850
N	0.525339	0.895731	0.235424

N	-0.712610	1.293497	0.439705
N	-1.461979	0.281381	0.085634
C	-1.687883	-4.283594	-0.517907
H	-1.424548	-5.204433	0.012775
C	-2.640123	-4.279853	-1.539151
H	-3.156453	-5.191714	-1.843816
C	-2.898224	-3.050506	-2.152041
H	-3.628513	-2.958942	-2.963006
C	-1.381865	-2.048379	-0.789685
C	-0.727848	-0.792129	-0.344437
C	0.604145	-0.397173	-0.244645
C	1.611648	1.791845	0.572012
C	2.287416	1.581411	1.787238
C	3.336691	2.464552	2.080422
H	3.893381	2.343137	3.011639
C	3.674883	3.496586	1.206760
H	4.493159	4.174658	1.460301
C	2.976141	3.673205	0.011020
H	3.254057	4.489298	-0.657701
C	1.925395	2.817607	-0.341168
C	1.927968	0.466637	2.761334
H	1.070417	-0.091133	2.354146
C	1.483718	1.036056	4.119546
H	1.166393	0.221381	4.789264
H	0.641438	1.735266	4.006827
H	2.305222	1.573984	4.617986
C	3.089386	-0.530561	2.917707
H	3.965910	-0.049956	3.380289
H	3.399779	-0.942915	1.946565
H	2.788992	-1.368768	3.565862
C	1.142331	3.018199	-1.633007
H	0.612019	2.077878	-1.859961
C	2.043041	3.320284	-2.839875
H	2.829506	2.559341	-2.965407
H	2.534944	4.300930	-2.749056
H	1.442095	3.343622	-3.761922
C	0.077528	4.113235	-1.441813
H	-0.605948	3.864851	-0.617078
H	-0.517897	4.239513	-2.358921
H	0.554228	5.078691	-1.209108
C	-2.900253	0.334299	0.254397
C	-3.434754	-0.329937	1.379654
C	-4.828270	-0.310277	1.510139
H	-5.298288	-0.802426	2.361998
C	-5.629063	0.340273	0.568237
H	-6.714645	0.341554	0.691781
C	-5.057530	0.991608	-0.522402
H	-5.700531	1.496759	-1.245614
C	-3.667313	1.005935	-0.711527

C	-2.539811	-0.963376	2.441819
H	-1.634402	-1.349160	1.947157
C	-2.091131	0.106223	3.457319
H	-1.394410	-0.325533	4.192730
H	-2.959282	0.508349	4.003061
H	-1.587307	0.950559	2.963229
C	-3.181082	-2.164405	3.147443
H	-3.546177	-2.910564	2.424775
H	-4.026089	-1.865030	3.786888
H	-2.441202	-2.653410	3.799270
C	-3.064103	1.721228	-1.913504
H	-1.967920	1.639809	-1.853100
C	-3.495240	1.060809	-3.233986
H	-3.202132	0.001755	-3.249458
H	-3.019554	1.569968	-4.087183
H	-4.585902	1.124987	-3.375665
C	-3.410836	3.219676	-1.890845
H	-3.084404	3.696104	-0.954288
H	-4.495461	3.381165	-1.992109
H	-2.921128	3.737857	-2.729636
C	1.859774	-1.095370	-0.521551
C	2.038067	-2.428129	-0.088365
H	1.225472	-2.936031	0.431218
C	3.238852	-3.084210	-0.305307
H	3.393950	-4.109182	0.035679
C	4.302111	-2.435651	-0.966860
C	4.129352	-1.110250	-1.410948
H	4.926571	-0.584600	-1.935038
C	2.921188	-0.455277	-1.185020
H	2.807983	0.566878	-1.544042
C	6.549016	-2.582352	-1.774039
H	7.328323	-3.354572	-1.774658
H	6.919634	-1.694753	-1.232284
H	6.316188	-2.303001	-2.816302
N	-2.268229	-1.933861	-1.779905

89

TS-decomp+trans SCF Done: -1931.19727192 A.U.

Au	-1.436961	-1.115241	-1.015757
N	-0.243074	1.278007	-2.659850
N	1.240298	-0.811165	0.743732
N	1.638354	0.269679	1.388800
N	1.473888	1.265528	0.555073
C	-0.579579	2.080568	-3.671034
H	-1.306028	1.688735	-4.390587
C	-0.034693	3.361185	-3.804366
H	-0.315329	4.020176	-4.627471
C	0.896698	3.749567	-2.836320
H	1.385023	4.728950	-2.875156

C	0.641508	1.755273	-1.777000
C	0.965300	0.865377	-0.651734
C	0.754209	-0.518264	-0.514372
C	1.400593	-2.109907	1.364831
C	0.371321	-2.591630	2.195933
C	0.554572	-3.873518	2.733668
H	-0.212613	-4.299836	3.380586
C	1.709426	-4.608994	2.467191
H	1.825425	-5.607090	2.896179
C	2.729038	-4.073200	1.680188
H	3.639752	-4.650467	1.514788
C	2.604269	-2.798598	1.113198
C	-0.820901	-1.730830	2.590918
H	-1.020636	-1.020743	1.773183
C	-0.475543	-0.908714	3.848703
H	-1.324479	-0.265046	4.126965
H	0.405352	-0.269608	3.687716
H	-0.258695	-1.574669	4.699183
C	-2.106106	-2.538708	2.803216
H	-2.050427	-3.166698	3.706073
H	-2.316183	-3.193125	1.943768
H	-2.958454	-1.855999	2.931253
C	3.766631	-2.157354	0.363824
H	3.371783	-1.339022	-0.259806
C	4.497179	-3.124714	-0.576935
H	3.800556	-3.625271	-1.266702
H	5.039585	-3.904303	-0.020309
H	5.242631	-2.578587	-1.175340
C	4.738400	-1.520699	1.375832
H	4.228755	-0.780192	2.010041
H	5.565303	-1.016329	0.852571
H	5.170167	-2.289921	2.035167
C	1.761722	2.606304	1.030534
C	0.675058	3.388218	1.461305
C	0.982707	4.664084	1.952830
H	0.177545	5.307912	2.311929
C	2.299096	5.122408	1.993369
H	2.513400	6.121356	2.380440
C	3.344474	4.316077	1.542866
H	4.367518	4.695283	1.572952
C	3.102764	3.027955	1.048498
C	-0.773947	2.915870	1.410065
H	-0.815649	1.922625	0.935629
C	-1.361241	2.752341	2.822079
H	-2.381736	2.342159	2.761866
H	-1.414549	3.718893	3.347595
H	-0.750081	2.070099	3.431313
C	-1.633386	3.857820	0.549811
H	-1.248056	3.924831	-0.479935

H	-1.654020	4.876879	0.965682
H	-2.669612	3.493845	0.510176
C	4.247670	2.178962	0.510655
H	3.868829	1.163325	0.318684
C	4.745312	2.742056	-0.832914
H	3.920725	2.820474	-1.556720
H	5.529139	2.094192	-1.256719
H	5.175047	3.748038	-0.701486
C	5.392267	2.042003	1.526140
H	5.035537	1.629730	2.482220
H	5.869602	3.012389	1.732663
H	6.172473	1.372461	1.133023
N	1.238675	2.948149	-1.825843
C	-3.182158	-0.345666	-0.332570
C	-3.485999	1.008554	-0.592388
C	-4.132934	-1.116593	0.357058
H	-2.771261	1.638351	-1.126181
C	-4.692826	1.563257	-0.183898
C	-5.355198	-0.571803	0.765188
H	-3.938058	-2.167091	0.574282
H	-4.939373	2.607547	-0.387529
C	-5.642791	0.779001	0.501599
H	-6.072676	-1.208526	1.282907
O	-6.780082	1.406530	0.857428
C	-7.798193	0.690362	1.532811
H	-8.615908	1.401830	1.705185
H	-7.446538	0.303102	2.505654
H	-8.174748	-0.151059	0.924679
I	0.982003	-2.052972	-2.168888

73

decomp+trans SCF Done: -1449.47612688 A.U.

N	-1.465774	2.839316	-1.073203
N	1.081947	-0.359940	0.219951
N	0.077040	-1.086399	0.655013
N	-1.004842	-0.490222	0.216529
C	-2.401859	3.655563	-1.565187
H	-2.175845	4.727019	-1.561419
C	-3.617103	3.170112	-2.057479
H	-4.384013	3.839879	-2.450114
C	-3.797841	1.784107	-2.031806
H	-4.713704	1.320074	-2.412466
C	-1.751222	1.536940	-1.084680
C	-0.724699	0.642950	-0.506013
C	0.660425	0.727658	-0.497502
C	2.443273	-0.734479	0.541961
C	3.132703	-1.547096	-0.375801
C	4.448425	-1.886777	-0.035762
H	5.033250	-2.518833	-0.705131

C	5.022595	-1.426926	1.150718
H	6.050392	-1.705515	1.394921
C	4.303225	-0.613838	2.027987
H	4.776242	-0.265914	2.947160
C	2.983651	-0.239321	1.742613
C	2.462467	-2.074894	-1.638095
H	1.657448	-1.372200	-1.912508
C	1.804848	-3.438887	-1.354884
H	1.269114	-3.802987	-2.245472
H	1.087681	-3.377338	-0.523008
H	2.567291	-4.186822	-1.085673
C	3.410474	-2.147331	-2.842234
H	4.185089	-2.918154	-2.707756
H	3.913399	-1.184205	-3.019275
H	2.846273	-2.412646	-3.749390
C	2.161607	0.610533	2.704059
H	1.367743	1.109242	2.122076
C	2.977985	1.723588	3.374666
H	3.507192	2.341098	2.632772
H	3.723343	1.318311	4.076424
H	2.312045	2.379739	3.955788
C	1.471488	-0.287677	3.748797
H	0.835645	-1.048456	3.271024
H	0.842245	0.315756	4.421126
H	2.221214	-0.812364	4.361880
C	-2.302165	-1.019210	0.592691
C	-2.823357	-2.088340	-0.156099
C	-4.083127	-2.563077	0.230877
H	-4.533878	-3.392647	-0.317243
C	-4.772089	-1.989155	1.299714
H	-5.755172	-2.374791	1.579822
C	-4.214703	-0.931061	2.017839
H	-4.765070	-0.499692	2.856194
C	-2.954314	-0.417327	1.684373
C	-2.092543	-2.709209	-1.339077
H	-1.122551	-2.200927	-1.455940
C	-1.793922	-4.196929	-1.090016
H	-1.209227	-4.615043	-1.923879
H	-2.722530	-4.783365	-1.009087
H	-1.221492	-4.343034	-0.161257
C	-2.873078	-2.494832	-2.647092
H	-3.059417	-1.424629	-2.817662
H	-3.844403	-3.013748	-2.621880
H	-2.304895	-2.894979	-3.501679
C	-2.357409	0.739364	2.478322
H	-1.334529	0.925823	2.113912
C	-3.163133	2.032798	2.261750
H	-3.231895	2.296353	1.195458
H	-2.691187	2.875101	2.791204

H	-4.189859	1.926433	2.645751
C	-2.235902	0.397397	3.972755
H	-1.656918	-0.525253	4.126625
H	-3.224553	0.258553	4.436954
H	-1.730674	1.214835	4.510788
N	-2.862172	0.963278	-1.548990
I	1.980087	2.084438	-1.362047

8

AgSbF6 SCF Done: -751.118958803 A.U.

Sb	-1.034831	-0.000139	-0.000323
F	0.254079	0.281551	-1.454712
F	0.254728	1.120151	0.969656
F	-2.003753	1.521729	-0.525518
F	-2.004437	-1.217368	-1.053467
F	0.252889	-1.400132	0.485602
F	-2.005848	-0.303535	1.579524
Ag	2.128670	-0.000308	0.000143

2

AgI SCF Done: -158.473946668 A.U.

Ag	0.000000	0.000000	-1.395453
I	0.000000	0.000000	1.237477

95

BC+cis SCF Done: -2523.91060341 A.U.

Au	0.943978	-0.295226	-0.843998
O	3.906488	5.067548	-0.297983
N	-0.287555	-2.150011	-1.323750
N	-1.451099	1.274703	0.469234
N	-2.716341	0.999458	0.751055
N	-2.959541	-0.177944	0.227742
C	0.084453	-3.293358	-1.909504
H	1.144145	-3.412649	-2.136833
C	-0.864767	-4.284657	-2.173693
H	-0.574538	-5.224729	-2.644425
C	-2.184973	-4.023332	-1.803981
H	-2.978479	-4.757455	-1.975576
C	-1.596971	-1.983114	-1.011207
C	-1.853296	-0.682907	-0.386905
C	-0.858960	0.273906	-0.239410
C	-0.893177	2.528642	0.940163
C	-0.029181	2.486741	2.053089
C	0.490634	3.716525	2.474352
H	1.174070	3.749736	3.322532
C	0.144218	4.905079	1.828699
H	0.560763	5.850650	2.183561
C	-0.720928	4.898884	0.735934
H	-0.973534	5.839615	0.245023

C	-1.257654	3.698904	0.251255
C	0.272868	1.186186	2.791664
H	0.373962	0.385557	2.041820
C	-0.900463	0.814318	3.721096
H	-0.721356	-0.164704	4.192233
H	-1.861119	0.767414	3.188095
H	-1.007539	1.562931	4.521751
C	1.591232	1.204544	3.573743
H	1.530994	1.855939	4.459831
H	2.428654	1.546324	2.950439
H	1.826102	0.188889	3.924362
C	-2.198935	3.678646	-0.948409
H	-2.170265	2.663766	-1.382459
C	-1.772489	4.644492	-2.064287
H	-0.722917	4.493724	-2.358059
H	-1.890415	5.696096	-1.761035
H	-2.402697	4.493744	-2.954154
C	-3.648865	3.957791	-0.508554
H	-3.989216	3.235488	0.247176
H	-4.331341	3.904838	-1.371001
H	-3.729335	4.966047	-0.072418
C	-4.270237	-0.778355	0.377361
C	-4.463409	-1.656765	1.456895
C	-5.734998	-2.236100	1.565212
H	-5.942377	-2.923730	2.387459
C	-6.738014	-1.943643	0.641386
H	-7.721403	-2.407933	0.746114
C	-6.501716	-1.059392	-0.413124
H	-7.302387	-0.843385	-1.122177
C	-5.252834	-0.446673	-0.573581
C	-3.371895	-1.979140	2.470010
H	-2.465715	-1.412653	2.202871
C	-3.773180	-1.528234	3.884409
H	-2.948590	-1.708296	4.591331
H	-4.651905	-2.083883	4.247332
H	-4.018388	-0.455473	3.909102
C	-2.996944	-3.470671	2.434366
H	-2.702684	-3.785091	1.421478
H	-3.842114	-4.103121	2.748316
H	-2.157520	-3.671935	3.117996
C	-4.966673	0.477622	-1.751339
H	-4.068189	1.068375	-1.512836
C	-4.656516	-0.348863	-3.013364
H	-3.829213	-1.054226	-2.840951
H	-4.386704	0.310125	-3.853770
H	-5.534943	-0.941622	-3.314324
C	-6.095233	1.487238	-2.004721
H	-6.331409	2.062638	-1.097087
H	-7.017962	0.993610	-2.346633

H	-5.796867	2.195770	-2.792976
C	1.855759	1.505418	-0.623703
C	2.948947	1.615382	0.240473
H	3.295269	0.775042	0.838442
C	3.622992	2.829984	0.307033
H	4.491087	2.948725	0.957944
C	3.204264	3.932582	-0.463296
C	2.104872	3.795818	-1.328860
H	1.761247	4.628435	-1.941640
C	1.420381	2.578056	-1.397223
H	0.553828	2.490773	-2.054710
C	3.568784	6.221087	-1.046719
H	4.277852	7.003486	-0.748096
H	2.541374	6.561848	-0.825476
H	3.665456	6.043200	-2.132352
N	-2.551053	-2.870224	-1.225862
Sb	3.683692	-2.356163	0.169062
F	2.235022	-3.555555	-0.173978
F	2.780242	-1.241926	-1.317846
F	4.382313	-3.367677	1.582046
F	4.869744	-0.897554	0.352047
F	4.663735	-3.130328	-1.234063
F	2.432097	-1.390610	1.284113

95
BC+trans SCF Done: -2523.87311318 A.U.

Au	1.193585	0.765867	0.068339
N	0.181029	2.470736	-0.546165
N	-1.639622	-0.905549	0.681533
N	-2.925228	-0.594520	0.560038
N	-2.960558	0.603542	0.037146
C	0.755639	3.625313	-0.926996
H	1.844898	3.670585	-0.880382
C	-0.033112	4.690642	-1.354353
H	0.426534	5.629846	-1.664207
C	-1.416868	4.499900	-1.367381
H	-2.097437	5.294405	-1.690591
C	-1.191721	2.364603	-0.595898
C	-1.689555	1.076127	-0.155447
C	-0.815621	0.088368	0.258662
C	-1.277989	-2.171601	1.283007
C	-1.528046	-3.351561	0.553781
C	-1.191730	-4.554000	1.184689
H	-1.358737	-5.497444	0.663762
C	-0.622429	-4.567513	2.457750
H	-0.356404	-5.520276	2.920949
C	-0.377572	-3.377029	3.137902
H	0.072118	-3.407329	4.132227
C	-0.705585	-2.139652	2.568448

C	-2.137055	-3.339980	-0.843652
H	-1.952476	-2.344371	-1.276462
C	-3.659121	-3.566758	-0.782220
H	-4.095139	-3.521385	-1.792969
H	-4.158835	-2.813011	-0.156241
H	-3.886050	-4.559325	-0.361294
C	-1.469885	-4.349538	-1.790284
H	-1.695680	-5.389358	-1.507031
H	-0.379689	-4.224741	-1.812929
H	-1.849050	-4.205636	-2.814110
C	-0.469417	-0.854638	3.355773
H	-0.754468	0.002880	2.726541
C	1.016392	-0.671073	3.710349
H	1.654495	-0.754779	2.818737
H	1.355281	-1.436311	4.425754
H	1.181671	0.312633	4.179358
C	-1.365473	-0.797658	4.605121
H	-2.430204	-0.882996	4.337944
H	-1.219756	0.153534	5.142247
H	-1.128491	-1.615261	5.303601
C	-4.232912	1.223456	-0.268473
C	-4.832764	0.903537	-1.499165
C	-6.059371	1.525805	-1.769809
H	-6.573286	1.318134	-2.709382
C	-6.634161	2.405858	-0.852518
H	-7.592765	2.875994	-1.084415
C	-6.003821	2.690090	0.361027
H	-6.479767	3.375151	1.063539
C	-4.775789	2.101677	0.686798
C	-4.159170	-0.018695	-2.509093
H	-3.462350	-0.672043	-1.962570
C	-5.145571	-0.946884	-3.230552
H	-4.593403	-1.671352	-3.848643
H	-5.817251	-0.391841	-3.903863
H	-5.763751	-1.509368	-2.514953
C	-3.329789	0.805719	-3.511764
H	-2.594659	1.446199	-2.999695
H	-3.981746	1.464612	-4.107248
H	-2.786656	0.143115	-4.203590
C	-4.089488	2.340825	2.027030
H	-3.002319	2.228095	1.872702
C	-4.309249	3.754916	2.579069
H	-4.048252	4.524598	1.836029
H	-3.686916	3.911577	3.473537
H	-5.354695	3.917060	2.883910
C	-4.519222	1.268703	3.048188
H	-4.316789	0.251672	2.679783
H	-5.599368	1.342274	3.250701
H	-3.982387	1.403508	4.000484

N	-1.979441	3.347618	-0.991634
C	3.030685	1.691056	-0.060756
C	3.774577	1.683158	-1.246740
C	3.497523	2.344463	1.078767
H	3.430505	1.134499	-2.124849
C	4.997051	2.349857	-1.281704
C	4.732129	3.009677	1.044293
H	2.927549	2.343271	2.012865
H	5.610067	2.351425	-2.185191
C	5.488835	3.016565	-0.140961
H	5.090143	3.505332	1.946416
O	6.681022	3.623227	-0.283243
C	7.281642	4.269911	0.824959
H	8.244567	4.659619	0.471394
H	7.461047	3.565886	1.656570
H	6.664926	5.111279	1.188651
Sb	2.305835	-2.494827	-0.849853
F	2.180513	-0.959634	0.571268
F	2.781696	-1.058362	-2.014553
F	1.675389	-3.563356	0.569501
F	4.090228	-2.656680	-0.285528
F	2.301962	-3.843857	-2.153790
F	0.500300	-1.959015	-1.223669

15
ACID SCF Done: -345.549453320 A.U.

C	-1.599944	0.062713	-0.026360
O	-1.838146	1.244208	0.013678
O	-2.561194	-0.871452	-0.184851
H	-3.408132	-0.397516	-0.249232
C	-0.229798	-0.566178	0.095169
H	-0.250381	-1.229754	0.978073
H	-0.085140	-1.238677	-0.767230
C	0.896492	0.466551	0.195666
H	0.896607	1.098833	-0.706662
H	0.669984	1.141891	1.039453
C	2.242551	-0.171012	0.394506
H	2.350319	-0.785524	1.299137
C	3.284157	-0.053924	-0.433611
H	3.221730	0.545893	-1.348264
H	4.238992	-0.546093	-0.228105

8
K3PO4 SCF Done: -2441.79629028 A.U.

P	-0.000213	-0.000864	0.377020
O	-0.186973	-1.501863	0.822848
O	1.389753	0.585330	0.835915
O	-1.206061	0.907292	0.832639
O	0.002579	0.007331	-1.225174

K	-2.523909	-1.065324	-0.276994
K	0.337381	2.715113	-0.276765
K	2.186992	-1.648303	-0.277037

K2HPO4 SCF Done: -1842.48541592 A.U.

P	0.011424	0.646009	-0.159826
O	0.412202	1.030000	1.456332
O	-1.352970	1.282229	-0.449423
O	-0.090688	-0.922581	-0.022105
O	1.238777	1.073699	-0.971693
K	2.491759	-0.866617	0.012885
K	-2.616032	-0.785300	0.028826
H	0.531260	1.989498	1.499990

15

ACETAT SCF Done: -944.872038727 A.U.

C	-0.520276	0.352813	-0.079638
O	-0.843306	-0.853678	-0.280501
O	-1.331497	1.285738	0.181088
C	0.964287	0.730901	-0.184447
H	1.086145	1.256506	-1.149980
H	1.179108	1.485209	0.589270
C	1.928746	-0.456851	-0.099166
H	1.830295	-0.945302	0.884940
H	1.599702	-1.204395	-0.841561
C	3.357951	-0.072597	-0.349286
H	3.560104	0.386422	-1.327859
C	4.373188	-0.225558	0.506827
H	4.221057	-0.670822	1.496522
H	5.391839	0.083949	0.254592
K	-3.268063	-0.306331	0.111771

104

C+cis SCF Done: -3324.81060709 A.U.

Au	-0.888164	-0.986966	-1.257766
O	-6.273252	1.930100	-0.401688
N	1.213206	-1.642771	-1.761177
N	0.100862	1.540758	0.477618
N	1.279260	2.038789	0.828223
N	2.176928	1.285365	0.236898
C	1.625958	-2.735842	-2.416782
H	0.871963	-3.376032	-2.867228
C	2.999811	-2.952265	-2.633967
H	3.336955	-3.826943	-3.189516
C	3.884583	-2.049379	-2.071343
H	4.966598	-2.165951	-2.182417
C	2.164844	-0.824319	-1.234678
C	1.585686	0.295056	-0.491757
C	0.216207	0.468655	-0.355287

C	-1.086436	2.159469	1.042960
C	-1.659067	3.236903	0.343613
C	-2.779195	3.838813	0.932467
H	-3.270719	4.672476	0.430265
C	-3.270315	3.390335	2.158576
H	-4.137610	3.883792	2.604242
C	-2.672847	2.316102	2.817799
H	-3.083240	1.973360	3.768219
C	-1.564622	1.658148	2.269084
C	-1.050492	3.767362	-0.949204
H	-0.555627	2.920918	-1.456291
C	0.025505	4.826240	-0.635060
H	0.504221	5.177633	-1.562135
H	0.807870	4.434266	0.030598
H	-0.430850	5.696447	-0.137248
C	-2.088296	4.329472	-1.929452
H	-2.529633	5.268444	-1.560444
H	-2.904001	3.615111	-2.106843
H	-1.608393	4.558167	-2.893587
C	-0.914436	0.466812	2.962624
H	-0.526159	-0.202650	2.179271
C	-1.898773	-0.381763	3.780181
H	-2.813905	-0.605055	3.211932
H	-2.195791	0.117800	4.716226
H	-1.437835	-1.347621	4.033228
C	0.262248	0.919754	3.847594
H	1.005343	1.503369	3.285981
H	0.767630	0.045414	4.288703
H	-0.095176	1.552240	4.675885
C	3.582638	1.614782	0.355201
C	4.119816	2.500921	-0.597046
C	5.485039	2.792040	-0.480027
H	5.952785	3.469804	-1.196120
C	6.256327	2.228247	0.536632
H	7.318857	2.471803	0.608837
C	5.681725	1.358883	1.463335
H	6.300755	0.930934	2.254379
C	4.322365	1.026475	1.394047
C	3.291834	3.080522	-1.738192
H	2.226586	2.920716	-1.510016
C	3.482597	4.596713	-1.892054
H	2.805952	4.985507	-2.668713
H	4.508840	4.850850	-2.199142
H	3.266003	5.126760	-0.952476
C	3.599280	2.337711	-3.051668
H	3.442389	1.253563	-2.947102
H	4.648585	2.491614	-3.349992
H	2.957535	2.709162	-3.866302
C	3.713308	0.059461	2.401544

H	2.632513	-0.016350	2.199382
C	4.319188	-1.346603	2.240208
H	4.159993	-1.739655	1.223640
H	3.888352	-2.041793	2.981855
H	5.405025	-1.336981	2.420120
C	3.862879	0.574242	3.842280
H	3.427447	1.578017	3.954797
H	4.921165	0.628602	4.141130
H	3.352083	-0.099274	4.548224
C	-2.675482	-0.048349	-0.941102
C	-3.224238	0.738592	-1.960879
H	-2.713419	0.867197	-2.917747
C	-4.448433	1.369923	-1.751286
H	-4.907357	1.983516	-2.528919
C	-5.117640	1.244610	-0.518869
C	-4.554536	0.449880	0.492347
H	-5.040971	0.337990	1.460660
C	-3.329115	-0.191693	0.276966
H	-2.877184	-0.789599	1.067222
C	-7.005148	1.862802	0.807763
H	-7.892561	2.493784	0.670150
H	-6.417133	2.247766	1.660104
H	-7.329316	0.830830	1.031656
N	3.466203	-0.986769	-1.350229
O	-0.895660	-2.226071	1.042800
O	-1.058371	-3.603198	2.782662
C	-1.410533	-3.225633	1.649883
C	-2.449923	-4.086075	0.919056
H	-3.298642	-4.249784	1.599976
H	-1.968902	-5.070538	0.795851
C	-2.940667	-3.577800	-0.437279
C	-1.865976	-3.335504	-1.441387
H	-0.935457	-3.914046	-1.300666
C	-1.995917	-2.527216	-2.558734
H	-1.292934	-2.631699	-3.391910
H	-2.960908	-2.068473	-2.795267
H	-3.575491	-4.361256	-0.899494
H	-3.591216	-2.693428	-0.352202
K	1.424874	-3.402862	2.040574
Cl	1.252463	-4.720542	-0.559786

104
C+trans SCF Done: -3324.82047234 A.U.

Au	1.248725	-0.197131	0.389925
O	6.989119	-2.625600	0.550351
N	0.312413	-1.960116	1.109959
N	-1.627107	1.260270	-0.287626
N	-2.873897	0.815021	-0.435507
N	-2.846528	-0.432926	-0.034779

C	0.927898	-3.037643	1.613356
H	2.000212	-2.951841	1.792968
C	0.210430	-4.205163	1.868938
H	0.707169	-5.086026	2.276787
C	-1.154209	-4.193497	1.574875
H	-1.778089	-5.078428	1.736458
C	-1.035930	-2.031775	0.870207
C	-1.592157	-0.786121	0.372230
C	-0.778667	0.318269	0.191234
C	-1.315380	2.647499	-0.560529
C	-1.597268	3.587118	0.448965
C	-1.243545	4.918072	0.185679
H	-1.442084	5.687594	0.932534
C	-0.636286	5.271093	-1.018636
H	-0.366446	6.313882	-1.202558
C	-0.373149	4.307698	-1.994192
H	0.097791	4.613195	-2.928787
C	-0.709467	2.962931	-1.791363
C	-2.295532	3.196422	1.746716
H	-2.084162	2.129723	1.934663
C	-3.822183	3.346819	1.598161
H	-4.333608	3.029730	2.520175
H	-4.208704	2.743591	0.764112
H	-4.086387	4.398968	1.406750
C	-1.785131	3.972860	2.969071
H	-2.081594	5.032541	2.931552
H	-0.689574	3.926233	3.056410
H	-2.218576	3.550825	3.888994
C	-0.487117	1.896673	-2.853038
H	-0.208748	0.960265	-2.347409
C	0.656264	2.230436	-3.817331
H	1.579859	2.484784	-3.271827
H	0.415303	3.078607	-4.477085
H	0.840253	1.367191	-4.478572
C	-1.786514	1.619802	-3.633385
H	-2.616941	1.357706	-2.962292
H	-1.637985	0.777109	-4.325996
H	-2.093151	2.503540	-4.216025
C	-4.041663	-1.245321	-0.119222
C	-4.948542	-1.199077	0.955059
C	-6.087869	-2.005779	0.843570
H	-6.823674	-2.013211	1.649601
C	-6.294854	-2.802804	-0.284177
H	-7.192999	-3.421582	-0.350329
C	-5.368043	-2.816664	-1.326072
H	-5.546547	-3.445623	-2.200510
C	-4.205540	-2.034731	-1.270686
C	-4.683961	-0.372555	2.207915
H	-3.894418	0.358846	1.976166

C	-5.910124	0.434805	2.657154
H	-5.648806	1.075768	3.513674
H	-6.734675	-0.218912	2.981290
H	-6.284125	1.079686	1.847773
C	-4.158332	-1.275665	3.339041
H	-3.267255	-1.838357	3.020703
H	-4.922623	-2.011111	3.637381
H	-3.897833	-0.677300	4.226799
C	-3.186608	-2.069237	-2.402234
H	-2.345077	-1.405275	-2.156865
C	-2.584961	-3.474971	-2.566244
H	-2.188396	-3.846770	-1.609737
H	-1.749868	-3.439300	-3.280788
H	-3.335052	-4.195921	-2.930105
C	-3.792497	-1.557099	-3.718793
H	-4.195550	-0.538711	-3.607838
H	-4.610209	-2.207698	-4.068307
H	-3.020944	-1.536751	-4.504120
C	3.157385	-0.978945	0.524531
C	3.489574	-2.105094	-0.254234
H	2.731656	-2.566521	-0.896376
C	4.780639	-2.631512	-0.222378
H	5.051590	-3.505031	-0.819832
C	5.770884	-2.048590	0.593313
C	5.437498	-0.935330	1.382578
H	6.176258	-0.464600	2.031278
C	4.138889	-0.408898	1.338715
H	3.906143	0.458955	1.957603
C	8.042968	-2.098483	1.335142
H	8.923634	-2.720937	1.131189
H	7.811163	-2.146754	2.414191
H	8.270280	-1.052475	1.062247
N	-1.767370	-3.109559	1.090719
O	1.910359	1.493623	-0.550988
O	3.976918	1.898914	-1.255294
C	3.077825	2.111621	-0.455262
C	3.206568	3.208997	0.591473
H	3.281641	4.141077	0.005655
H	4.192452	3.092907	1.071631
C	2.074053	3.337826	1.621097
C	2.139077	2.353435	2.756215
H	3.099242	2.305916	3.287674
C	1.131822	1.582370	3.184150
H	1.245845	0.911554	4.040005
H	0.150114	1.610659	2.704068
H	2.138896	4.351746	2.055338
H	1.102310	3.276118	1.109777
K	3.060092	-0.077747	-2.927676
Cl	0.552944	-1.604830	-2.261759

104
TS-CD+cis SCF Done: -3324.80797832 A.U.

Au	-0.914508	-0.823527	-1.340801
O	-6.027548	2.519253	-0.397693
N	1.140871	-1.647765	-1.804340
N	0.265373	1.546859	0.511626
N	1.473602	1.929458	0.907026
N	2.316020	1.124595	0.301120
C	1.472192	-2.757562	-2.475643
H	0.670935	-3.339416	-2.924057
C	2.824736	-3.077965	-2.692024
H	3.094952	-3.969003	-3.257977
C	3.775256	-2.251602	-2.118160
H	4.845543	-2.446325	-2.233436
C	2.151600	-0.918446	-1.255608
C	1.657704	0.218129	-0.477639
C	0.304482	0.499481	-0.357115
C	-0.881570	2.256392	1.049101
C	-1.237152	3.469727	0.434765
C	-2.312675	4.168485	1.000104
H	-2.632455	5.114182	0.560991
C	-2.978262	3.670186	2.118326
H	-3.809719	4.234499	2.547480
C	-2.603610	2.452343	2.688076
H	-3.152861	2.075782	3.550870
C	-1.545306	1.701377	2.162034
C	-0.465075	4.028779	-0.754506
H	0.033882	3.183567	-1.258797
C	0.629405	5.001111	-0.274025
H	1.214418	5.377860	-1.127272
H	1.319933	4.518115	0.432654
H	0.176319	5.866073	0.235916
C	-1.369873	4.694635	-1.800901
H	-1.810765	5.630408	-1.423645
H	-2.191633	4.029865	-2.103107
H	-0.782925	4.953545	-2.695762
C	-1.137628	0.356742	2.758821
H	-0.882807	-0.311547	1.919064
C	-2.261257	-0.343382	3.535188
H	-3.200229	-0.365434	2.963235
H	-2.464008	0.154638	4.496780
H	-1.978438	-1.385892	3.740950
C	0.105218	0.499923	3.659176
H	0.949518	0.978518	3.145056
H	0.433111	-0.488010	4.022999
H	-0.127665	1.115028	4.542716
C	3.741010	1.299777	0.490102
C	4.402234	2.207316	-0.357864

C	5.783090	2.347037	-0.167910
H	6.345883	3.034465	-0.801389
C	6.451133	1.618878	0.817235
H	7.528236	1.746582	0.947824
C	5.755829	0.731260	1.637980
H	6.295561	0.173785	2.406206
C	4.374844	0.546222	1.491801
C	3.680847	2.954007	-1.473764
H	2.600412	2.926382	-1.263911
C	4.073083	4.436482	-1.548431
H	3.460027	4.950472	-2.304850
H	5.125901	4.568009	-1.842417
H	3.921354	4.942316	-0.583127
C	3.909994	2.242696	-2.820607
H	3.611240	1.184654	-2.773752
H	4.975800	2.271195	-3.098241
H	3.336779	2.735272	-3.622018
C	3.627740	-0.431303	2.390891
H	2.561687	-0.407984	2.112395
C	4.129650	-1.871186	2.181847
H	3.997558	-2.197982	1.138990
H	3.600747	-2.569618	2.853759
H	5.199123	-1.963033	2.424730
C	3.710588	-0.009734	3.867212
H	3.340508	1.016397	4.010442
H	4.746967	-0.049856	4.236857
H	3.105429	-0.681004	4.497170
C	-2.641079	0.211049	-1.020043
C	-3.000418	1.224811	-1.917683
H	-2.386641	1.451602	-2.792331
C	-4.156798	1.965884	-1.687152
H	-4.460873	2.764120	-2.366994
C	-4.955244	1.714959	-0.555084
C	-4.593772	0.683610	0.325689
H	-5.188003	0.463741	1.212320
C	-3.432793	-0.063453	0.088843
H	-3.134633	-0.843148	0.787970
C	-6.873776	2.341475	0.721647
H	-7.661211	3.101897	0.641261
H	-6.327876	2.486750	1.671409
H	-7.341115	1.340450	0.726067
N	3.437162	-1.175639	-1.376612
O	-1.393947	-2.512599	0.789394
O	-1.670515	-3.633039	2.699029
C	-1.991527	-3.372195	1.530089
C	-3.107111	-4.164288	0.840834
H	-4.001727	-4.183203	1.480108
H	-2.749788	-5.205386	0.775675
C	-3.391474	-3.593870	-0.544703

C	-2.151699	-3.312489	-1.308590
H	-1.292298	-3.984654	-1.154181
C	-2.082348	-2.382187	-2.371070
H	-1.396964	-2.639043	-3.188830
H	-3.023812	-1.925806	-2.695450
H	-3.948367	-4.326684	-1.165664
H	-4.025118	-2.692627	-0.512979
K	0.882446	-3.477808	2.000598
Cl	0.995500	-4.745159	-0.608519

104

TS-CD+trans SCF Done: -3324.80078555 A.U.

Au	1.473251	-0.128064	0.434874
O	7.120451	-2.748852	0.377055
N	0.757696	-1.946319	-0.501951
N	-1.622880	1.090319	0.641491
N	-2.837960	0.567159	0.497409
N	-2.635881	-0.674927	0.127388
C	1.505797	-2.888482	-1.082996
H	2.585664	-2.730836	-1.084092
C	0.905152	-4.013415	-1.645667
H	1.504848	-4.779834	-2.137355
C	-0.481494	-4.124529	-1.520371
H	-1.015607	-5.004301	-1.893703
C	-0.598623	-2.097726	-0.503811
C	-1.303255	-0.941643	0.028049
C	-0.620396	0.210917	0.386371
C	-1.486461	2.488568	0.980527
C	-1.414963	2.833537	2.344248
C	-1.216197	4.189642	2.639582
H	-1.150726	4.515079	3.678405
C	-1.111990	5.135007	1.617455
H	-0.957186	6.186850	1.869832
C	-1.214821	4.755622	0.278145
H	-1.141067	5.515288	-0.500297
C	-1.409408	3.413530	-0.081810
C	-1.625125	1.792187	3.439574
H	-1.249466	0.825418	3.060822
C	-3.131639	1.617815	3.720943
H	-3.294619	0.832385	4.475089
H	-3.684583	1.342028	2.811880
H	-3.559454	2.556906	4.106026
C	-0.867542	2.097944	4.738830
H	-1.286685	2.973890	5.257518
H	0.202078	2.295348	4.564212
H	-0.948742	1.246108	5.431218
C	-1.593319	2.979409	-1.530922
H	-1.152983	1.976030	-1.653816
C	-0.905246	3.899617	-2.546454

H	0.166747	4.036427	-2.346587
H	-1.379506	4.893277	-2.589711
H	-1.008405	3.463387	-3.552976
C	-3.095333	2.863660	-1.863181
H	-3.625027	2.200069	-1.166658
H	-3.220785	2.456149	-2.877528
H	-3.580164	3.852611	-1.822505
C	-3.760879	-1.584665	0.026915
C	-4.116241	-2.282350	1.198931
C	-5.191732	-3.172332	1.096628
H	-5.505260	-3.742648	1.972706
C	-5.872287	-3.338069	-0.111568
H	-6.711389	-4.035742	-0.167810
C	-5.493886	-2.618210	-1.243036
H	-6.039750	-2.755485	-2.178490
C	-4.418740	-1.717504	-1.205651
C	-3.348698	-2.126005	2.508052
H	-2.755087	-1.200237	2.451901
C	-4.273712	-1.972761	3.724347
H	-3.679781	-1.763943	4.628223
H	-4.849624	-2.890362	3.920256
H	-4.987028	-1.146900	3.583414
C	-2.363801	-3.294425	2.697133
H	-1.679215	-3.387489	1.840212
H	-2.906313	-4.248932	2.788083
H	-1.764000	-3.156156	3.611310
C	-4.008517	-0.944981	-2.448987
H	-3.121987	-0.339006	-2.215263
C	-3.585893	-1.895311	-3.580441
H	-2.794160	-2.576937	-3.239448
H	-3.174686	-1.313486	-4.418064
H	-4.435831	-2.493047	-3.948987
C	-5.125844	0.010075	-2.899632
H	-5.408524	0.709361	-2.097825
H	-6.031575	-0.540562	-3.201315
H	-4.791002	0.601068	-3.766402
C	3.395953	-0.908478	0.499671
C	4.341566	-0.580412	-0.483856
H	4.103920	0.183725	-1.226663
C	5.578512	-1.222125	-0.495012
H	6.330977	-0.984182	-1.250011
C	5.898052	-2.189719	0.478135
C	4.953119	-2.504919	1.469631
H	5.169135	-3.245309	2.239852
C	3.707857	-1.858539	1.474864
H	2.985052	-2.127514	2.250942
C	7.533184	-3.713622	1.326898
H	8.551584	-4.010791	1.045709
H	6.881668	-4.605866	1.311802

H	7.549707	-3.297132	2.349916
N	-1.222089	-3.176097	-0.943595
O	2.423683	1.648967	-1.357533
O	2.512468	3.403177	-2.743097
C	2.551148	2.894738	-1.610576
C	2.801434	3.817596	-0.410267
H	2.426957	4.823009	-0.645890
H	3.896326	3.909500	-0.297254
C	2.178081	3.294621	0.877548
C	2.670115	1.974723	1.335607
H	3.707372	1.713625	1.098407
C	1.971536	1.139148	2.204134
H	2.529309	0.414040	2.804083
H	1.014806	1.482013	2.603217
H	2.443409	3.972088	1.717505
H	1.079718	3.301562	0.837747
K	1.604290	1.284919	-3.994148
Cl	-0.515602	-0.361941	-2.966020

2

KCl SCF Done: -1059.99937751 A.U.

Cl	0.000000	0.000000	-1.410783
K	0.000000	0.000000	1.262280

102

D+cis SCF Done: -2264.77263227 A.U.

Au	1.199419	1.245218	-0.460625
O	5.822332	-2.836817	-0.120354
N	-0.838259	2.431375	-0.676383
N	-0.476842	-1.557598	0.044184
N	-1.749874	-1.914876	0.176968
N	-2.439309	-0.811066	0.034655
C	-1.060022	3.721475	-0.950199
H	-0.189793	4.344154	-1.138364
C	-2.351686	4.248993	-0.987999
H	-2.521146	5.303771	-1.206646
C	-3.401572	3.369970	-0.725142
H	-4.443260	3.706977	-0.724056
C	-1.931441	1.654318	-0.454011
C	-1.612330	0.253445	-0.200626
C	-0.310856	-0.224514	-0.195009
C	0.533178	-2.589202	0.179153
C	1.133671	-3.090338	-0.993923
C	2.092787	-4.095756	-0.823586
H	2.601689	-4.510968	-1.692995
C	2.407680	-4.582724	0.446119
H	3.152443	-5.375610	0.548523
C	1.788538	-4.063526	1.580585
H	2.053753	-4.453733	2.564266

C	0.839756	-3.036811	1.476282
C	0.710925	-2.602356	-2.376266
H	0.563433	-1.511330	-2.305212
C	-0.631714	-3.241709	-2.786905
H	-0.982281	-2.824569	-3.744241
H	-1.419717	-3.086448	-2.037412
H	-0.512266	-4.329258	-2.914540
C	1.761770	-2.840228	-3.467000
H	1.872145	-3.911445	-3.698166
H	2.746048	-2.449040	-3.175457
H	1.452700	-2.340793	-4.398143
C	0.163810	-2.462640	2.716148
H	-0.229938	-1.466008	2.454133
C	1.135633	-2.257402	3.888199
H	2.022258	-1.678290	3.588577
H	1.483190	-3.215643	4.304370
H	0.633122	-1.715410	4.704139
C	-1.027285	-3.342100	3.140513
H	-1.765243	-3.437980	2.331165
H	-1.530944	-2.913262	4.020398
H	-0.681762	-4.353967	3.405884
C	-3.883876	-0.852391	0.137150
C	-4.619534	-1.022444	-1.047356
C	-6.015019	-1.040216	-0.921430
H	-6.632477	-1.174194	-1.811954
C	-6.624553	-0.890618	0.323969
H	-7.714456	-0.905417	0.398947
C	-5.855028	-0.728044	1.476978
H	-6.351556	-0.615288	2.442140
C	-4.455665	-0.708335	1.414442
C	-3.961010	-1.177462	-2.412106
H	-2.868973	-1.134443	-2.279008
C	-4.278404	-2.547230	-3.034124
H	-3.744303	-2.668248	-3.989587
H	-5.355498	-2.655430	-3.237000
H	-3.976871	-3.368715	-2.366434
C	-4.345877	-0.021862	-3.351425
H	-4.104723	0.952836	-2.900279
H	-5.424287	-0.029606	-3.575385
H	-3.807116	-0.107630	-4.308392
C	-3.614296	-0.487280	2.666108
H	-2.579497	-0.787771	2.439940
C	-3.591877	1.005447	3.043429
H	-3.230778	1.627783	2.211298
H	-2.939487	1.176920	3.914162
H	-4.603805	1.355975	3.302359
C	-4.074052	-1.353895	3.847701
H	-4.110459	-2.419263	3.575435
H	-5.071712	-1.058770	4.207975

H	-3.378174	-1.238541	4.693235
C	2.787824	-0.014485	-0.314368
C	3.525592	-0.363092	-1.450897
H	3.305301	0.079849	-2.424252
C	4.548069	-1.304019	-1.346269
H	5.127517	-1.606648	-2.220698
C	4.848868	-1.899921	-0.108740
C	4.133496	-1.508599	1.033549
H	4.345514	-1.944113	2.009290
C	3.104687	-0.566006	0.924283
H	2.545792	-0.289146	1.820046
C	6.209007	-3.454054	1.091702
H	7.009426	-4.161817	0.839996
H	5.371160	-4.007650	1.552671
H	6.596085	-2.718651	1.819670
N	-3.188498	2.076350	-0.467315
O	1.332004	4.238880	0.681522
O	0.672511	5.749774	2.202591
C	1.433264	5.437742	1.335671
C	2.600020	6.215749	0.741622
H	3.159763	6.727245	1.536209
H	2.178571	6.996139	0.084364
C	3.378810	5.155554	-0.038769
C	2.278040	4.144220	-0.411827
H	1.760836	4.497092	-1.326651
C	2.723386	2.710222	-0.587756
H	3.238435	2.595426	-1.554733
H	3.450462	2.454762	0.196977
H	3.907122	5.539131	-0.922439
H	4.118040	4.663523	0.613190

102

D+trans SCF Done: -2264.77966934 A.U.

Au	-1.437948	-0.390277	0.381571
O	-6.888252	-3.367058	0.223811
N	-0.398022	-2.331239	-0.260918
N	1.512108	1.175481	0.441879
N	2.781822	0.828001	0.263357
N	2.748578	-0.445897	-0.035004
C	-0.983086	-3.498433	-0.544729
H	-2.073258	-3.529191	-0.460363
C	-0.214808	-4.603371	-0.918866
H	-0.681064	-5.560903	-1.153935
C	1.169317	-4.424047	-0.975148
H	1.836080	-5.245665	-1.256443
C	0.953492	-2.250262	-0.350101
C	1.464334	-0.916724	-0.040513
C	0.629261	0.149463	0.278563
C	1.194016	2.558315	0.714691

C	1.252425	3.467214	-0.361210
C	0.861803	4.785629	-0.085647
H	0.884552	5.532291	-0.879952
C	0.440336	5.157566	1.191276
H	0.137762	6.190309	1.380587
C	0.411135	4.228914	2.233675
H	0.090886	4.550532	3.224684
C	0.795731	2.897867	2.022999
C	1.644883	3.027888	-1.769473
H	2.381050	2.217176	-1.670023
C	2.330224	4.133706	-2.582288
H	2.723715	3.714763	-3.520861
H	3.170116	4.582761	-2.030565
H	1.630018	4.937947	-2.858015
C	0.428376	2.462212	-2.528417
H	-0.331242	3.243506	-2.688916
H	-0.060184	1.635480	-1.992575
H	0.734659	2.090217	-3.518719
C	0.839799	1.874832	3.154988
H	0.483092	0.915212	2.745439
C	-0.073211	2.218200	4.337860
H	-1.108786	2.414391	4.018856
H	0.284972	3.102053	4.888623
H	-0.092441	1.380020	5.050710
C	2.291831	1.667704	3.633514
H	2.963881	1.380928	2.811590
H	2.335780	0.879754	4.401953
H	2.686899	2.596134	4.075324
C	3.986121	-1.137366	-0.325065
C	4.430417	-1.142223	-1.658057
C	5.639476	-1.802962	-1.910778
H	6.034561	-1.829968	-2.928304
C	6.346079	-2.424583	-0.879963
H	7.287613	-2.933305	-1.099991
C	5.862734	-2.401689	0.428921
H	6.428866	-2.894695	1.221849
C	4.661202	-1.752118	0.741788
C	3.656775	-0.463185	-2.781280
H	2.701036	-0.098018	-2.373437
C	4.415137	0.765417	-3.311581
H	3.820981	1.282282	-4.081943
H	5.374958	0.474062	-3.766654
H	4.630779	1.481480	-2.503532
C	3.306796	-1.451127	-3.905841
H	2.761999	-2.322908	-3.511204
H	4.209965	-1.821051	-4.415939
H	2.676095	-0.961154	-4.664242
C	4.124184	-1.740993	2.167148
H	3.193819	-1.151641	2.183087

C	3.764951	-3.163921	2.628684
H	3.056318	-3.639307	1.932566
H	3.309407	-3.141919	3.631373
H	4.659992	-3.803709	2.682741
C	5.099198	-1.052581	3.135647
H	5.328002	-0.026525	2.808759
H	6.050069	-1.603356	3.209596
H	4.664111	-1.002333	4.146228
C	-3.298913	-1.246809	0.368292
C	-3.642644	-2.200148	1.345274
H	-2.968255	-2.411814	2.180483
C	-4.848585	-2.894438	1.267439
H	-5.127666	-3.636009	2.018938
C	-5.748084	-2.643422	0.213793
C	-5.418367	-1.680276	-0.754718
H	-6.095988	-1.456436	-1.578816
C	-4.205242	-0.984063	-0.666347
H	-3.974612	-0.225933	-1.418461
C	-7.858084	-3.157683	-0.784489
H	-8.687502	-3.842665	-0.565629
H	-7.459539	-3.385842	-1.789518
H	-8.237529	-2.120165	-0.777254
N	1.751127	-3.252382	-0.692485
O	-2.684277	1.929883	-1.315609
O	-3.627528	2.721260	-3.190750
C	-3.256314	2.914113	-2.071645
C	-3.295554	4.194596	-1.245170
H	-4.224501	4.747038	-1.438346
H	-2.454427	4.828117	-1.576240
C	-3.101565	3.697593	0.188297
C	-2.245483	2.438801	-0.036039
H	-1.193338	2.734356	-0.149442
C	-2.373728	1.363386	1.015856
H	-1.854895	1.665595	1.936325
H	-3.424452	1.148461	1.240416
H	-2.602124	4.414952	0.853407
H	-4.069339	3.420220	0.636225

102

TS-DA+cis SCF Done: -2264.75640369 A.U.

Au	1.342202	0.614055	0.505093
O	5.918247	-2.263923	-2.345159
N	-0.881293	2.726430	-0.571726
N	-0.992136	-1.337699	0.454403
N	-2.264747	-1.571496	0.210051
N	-2.758003	-0.404856	-0.136727
C	-1.020591	4.012828	-0.900968
H	-0.101179	4.601270	-0.996260
C	-2.285351	4.577399	-1.110337

H	-2.400094	5.627891	-1.382341
C	-3.386918	3.735938	-0.947511
H	-4.410598	4.102031	-1.081127
C	-2.001993	2.002081	-0.459837
C	-1.798866	0.581654	-0.122234
C	-0.613877	-0.034548	0.272152
C	-0.114858	-2.416748	0.844596
C	0.554727	-3.115463	-0.178247
C	1.443771	-4.119205	0.225899
H	1.984211	-4.694682	-0.526635
C	1.652175	-4.391218	1.579792
H	2.349588	-5.180205	1.872023
C	0.978522	-3.666559	2.563026
H	1.156440	-3.891147	3.616720
C	0.071294	-2.654185	2.216554
C	0.290302	-2.818073	-1.648842
H	-0.046159	-1.771118	-1.721444
C	-0.849940	-3.709909	-2.175493
H	-1.078499	-3.466137	-3.224586
H	-1.768901	-3.578982	-1.584741
H	-0.564242	-4.772850	-2.126314
C	1.545414	-2.931567	-2.523139
H	1.897958	-3.971849	-2.605629
H	2.367895	-2.319845	-2.125423
H	1.323472	-2.585718	-3.544463
C	-0.641076	-1.842629	3.290972
H	-1.354693	-1.166600	2.796382
C	0.359044	-0.963094	4.061420
H	0.913565	-0.301074	3.376562
H	1.093981	-1.577664	4.605518
H	-0.164741	-0.334085	4.798417
C	-1.458006	-2.737032	4.236995
H	-2.184317	-3.347904	3.679803
H	-2.014263	-2.119792	4.959794
H	-0.811233	-3.418451	4.811807
C	-4.158841	-0.340227	-0.502100
C	-4.497719	-0.574828	-1.843509
C	-5.861631	-0.509418	-2.163936
H	-6.180534	-0.676081	-3.194479
C	-6.815181	-0.234139	-1.185103
H	-7.872703	-0.192145	-1.456646
C	-6.433110	-0.011243	0.140058
H	-7.196990	0.202036	0.888982
C	-5.085757	-0.052811	0.514885
C	-3.450549	-0.833405	-2.920282
H	-2.491412	-1.050405	-2.424533
C	-3.786532	-2.060071	-3.781501
H	-2.969654	-2.259782	-4.492614
H	-4.702266	-1.905531	-4.373056

H	-3.929225	-2.958314	-3.161947
C	-3.246568	0.423710	-3.785368
H	-2.972775	1.294509	-3.170860
H	-4.170765	0.678348	-4.328503
H	-2.450724	0.258782	-4.529090
C	-4.638399	0.166594	1.954604
H	-3.601616	0.541038	1.925916
C	-5.465638	1.230921	2.686846
H	-5.502648	2.170419	2.114505
H	-5.021226	1.443367	3.671672
H	-6.499662	0.896718	2.865557
C	-4.627119	-1.167004	2.726213
H	-3.992778	-1.915970	2.228677
H	-5.645195	-1.582439	2.794878
H	-4.249346	-1.020294	3.750827
C	3.217000	-0.034361	-0.142201
C	3.640224	0.392762	-1.409034
H	3.249992	1.313237	-1.848166
C	4.544572	-0.388010	-2.122120
H	4.873279	-0.096659	-3.121389
C	5.053172	-1.582152	-1.572865
C	4.637739	-1.984111	-0.290005
H	5.006509	-2.904985	0.160502
C	3.728085	-1.201712	0.428321
H	3.408099	-1.528599	1.419749
C	6.480265	-3.476020	-1.874730
H	7.143359	-3.839428	-2.669925
H	5.701674	-4.235148	-1.680070
H	7.071752	-3.320175	-0.955279
N	-3.249074	2.447177	-0.622589
O	2.571383	3.461112	-0.171046
O	1.888269	5.522038	-0.729140
C	2.680455	4.819358	-0.168604
C	3.913685	5.210514	0.630201
H	3.731825	6.137463	1.189484
H	4.727590	5.408447	-0.088454
C	4.186474	3.967883	1.480306
C	3.634394	2.841052	0.581034
H	4.409769	2.520905	-0.132974
C	3.089231	1.653619	1.336305
H	3.889152	1.090082	1.825362
H	2.375234	1.987984	2.114548
H	5.244674	3.813159	1.730618
H	3.615851	4.015926	2.421878

102
TS-DA+trans SCF Done: -2264.75913160 A.U.

Au	1.405561	-0.490311	0.342778
O	6.196295	-4.030581	-0.874485

N	-0.425409	-2.894663	-0.197654
N	-1.219942	1.168741	0.337401
N	-2.534190	1.144693	0.208414
N	-2.832351	-0.119895	0.042859
C	-0.332393	-4.211407	-0.373259
H	0.678010	-4.628994	-0.440528
C	-1.467501	-5.023207	-0.470588
H	-1.391004	-6.101172	-0.620498
C	-2.702897	-4.380579	-0.362626
H	-3.642665	-4.940137	-0.420826
C	-1.659829	-2.374007	-0.111993
C	-1.710540	-0.915567	0.071506
C	-0.627632	-0.063052	0.267088
C	-0.530383	2.431381	0.478160
C	-0.051284	2.776014	1.758479
C	0.671946	3.972284	1.856837
H	1.059344	4.294723	2.823777
C	0.890432	4.768764	0.730527
H	1.453569	5.700312	0.829613
C	0.385717	4.400023	-0.515863
H	0.575663	5.036961	-1.378071
C	-0.347037	3.214529	-0.678803
C	-0.369164	1.919278	2.981091
H	-0.324723	0.863900	2.666632
C	-1.804761	2.197432	3.471319
H	-2.061991	1.534185	4.312474
H	-2.547768	2.042897	2.675588
H	-1.898572	3.239218	3.816936
C	0.636003	2.079570	4.128108
H	0.574663	3.075935	4.593231
H	1.673749	1.926499	3.791458
H	0.424535	1.342335	4.917539
C	-0.924486	2.800533	-2.032813
H	-1.930842	2.398723	-1.834114
C	-0.097167	1.689382	-2.711832
H	0.017480	0.799609	-2.077903
H	0.907924	2.058999	-2.956692
H	-0.594192	1.375256	-3.643484
C	-1.102019	3.979874	-2.997450
H	-1.664163	4.807147	-2.537406
H	-1.657764	3.647413	-3.887501
H	-0.130358	4.364778	-3.343917
C	-4.214379	-0.461777	-0.219293
C	-5.047604	-0.743524	0.873905
C	-6.383942	-1.046175	0.582055
H	-7.074647	-1.269421	1.397888
C	-6.843777	-1.065520	-0.735494
H	-7.890366	-1.303340	-0.940258
C	-5.979844	-0.779867	-1.793519

H	-6.357385	-0.795985	-2.818094
C	-4.634045	-0.467519	-1.560022
C	-4.541419	-0.734712	2.309904
H	-3.467039	-0.492417	2.295114
C	-5.234347	0.356730	3.141874
H	-4.816367	0.388338	4.160608
H	-6.315846	0.166828	3.229962
H	-5.103295	1.349981	2.685388
C	-4.679895	-2.123561	2.955478
H	-4.170149	-2.889303	2.351241
H	-5.737217	-2.417256	3.051986
H	-4.241328	-2.124677	3.966100
C	-3.690523	-0.160911	-2.716862
H	-2.689982	0.045754	-2.308049
C	-3.544942	-1.372007	-3.653328
H	-3.217257	-2.265115	-3.098675
H	-2.804840	-1.162052	-4.441718
H	-4.499004	-1.615448	-4.147184
C	-4.125072	1.102340	-3.477979
H	-4.206192	1.967240	-2.801621
H	-5.103594	0.962787	-3.964058
H	-3.392991	1.347627	-4.263867
C	3.333543	-1.228950	0.213524
C	3.912288	-1.232164	-1.072421
H	3.610240	-0.487020	-1.811721
C	4.857236	-2.193529	-1.405975
H	5.302085	-2.229156	-2.402277
C	5.272258	-3.150555	-0.456058
C	4.722300	-3.124363	0.840911
H	5.033023	-3.843802	1.598201
C	3.771489	-2.156274	1.171024
H	3.365882	-2.135004	2.185803
C	6.695249	-5.015416	0.014939
H	7.428537	-5.601507	-0.553159
H	5.892578	-5.686557	0.368132
H	7.195709	-4.558149	0.886417
N	-2.801574	-3.061279	-0.180661
O	3.043094	1.907379	-1.052929
O	2.700871	3.571297	-2.520070
C	3.245521	3.169844	-1.532946
C	4.231916	3.888200	-0.626052
H	3.923728	4.932859	-0.488216
H	5.210677	3.894450	-1.135267
C	4.239833	3.037272	0.644301
C	3.868954	1.641326	0.096851
H	4.772398	1.117340	-0.253342
C	3.116491	0.792638	1.092385
H	3.774183	0.405018	1.874385
H	2.306309	1.375849	1.553229

H	5.199981	3.027373	1.177422
H	3.458364	3.386192	1.336669

102
TS-DA+ad SCF Done: -2264.84157315 A.U.

Au	1.383115	-0.231942	-0.740510
O	3.352509	-3.293862	-1.733239
N	-0.326439	2.594072	-1.058535
N	-1.153049	-1.236479	0.486676
N	-2.437283	-1.052158	0.722377
N	-2.705465	0.121992	0.199922
C	-0.246909	3.812252	-1.601289
H	0.747381	4.270595	-1.637953
C	-1.386282	4.474249	-2.075111
H	-1.323232	5.472490	-2.511374
C	-2.601604	3.796770	-1.964710
H	-3.537614	4.240231	-2.321301
C	-1.542110	2.037986	-0.991370
C	-1.594904	0.701500	-0.367964
C	-0.552418	-0.203775	-0.183521
C	-0.500736	-2.442205	0.940657
C	-0.433950	-3.528407	0.048930
C	0.211131	-4.683108	0.511158
H	0.269961	-5.560813	-0.134491
C	0.778734	-4.726759	1.786363
H	1.273502	-5.639426	2.128196
C	0.721418	-3.615341	2.627420
H	1.182453	-3.662761	3.615978
C	0.074117	-2.438717	2.223723
C	-1.055333	-3.463597	-1.339785
H	-1.165676	-2.401103	-1.606943
C	-2.463758	-4.084280	-1.328154
H	-2.935732	-3.993679	-2.319196
H	-3.112688	-3.585284	-0.592684
H	-2.417821	-5.154160	-1.068529
C	-0.157744	-4.090797	-2.416478
H	-0.074380	-5.182895	-2.297098
H	0.856794	-3.662814	-2.390913
H	-0.584652	-3.908960	-3.414988
C	0.050353	-1.212698	3.128178
H	-0.613129	-0.461608	2.674572
C	1.452292	-0.582987	3.219795
H	1.835839	-0.316664	2.222716
H	2.169831	-1.281007	3.680761
H	1.425004	0.330012	3.835865
C	-0.520807	-1.529789	4.518706
H	-1.527422	-1.968691	4.446588
H	-0.591922	-0.610036	5.119906
H	0.118739	-2.235703	5.071552

C	-4.052213	0.639722	0.329216
C	-5.004482	0.249395	-0.624096
C	-6.299335	0.761816	-0.462757
H	-7.076738	0.490446	-1.179834
C	-6.605752	1.616735	0.594845
H	-7.621557	2.005016	0.700873
C	-5.627074	1.980749	1.522297
H	-5.890648	2.646420	2.345301
C	-4.317222	1.498812	1.411461
C	-4.668401	-0.657357	-1.800452
H	-3.619374	-0.977549	-1.702701
C	-5.526944	-1.932251	-1.795545
H	-5.221834	-2.602856	-2.614175
H	-6.594145	-1.699973	-1.938073
H	-5.424876	-2.480440	-0.846436
C	-4.785869	0.106930	-3.130359
H	-4.156217	1.008763	-3.118096
H	-5.825399	0.420248	-3.317873
H	-4.472780	-0.532265	-3.971359
C	-3.248313	1.836413	2.444875
H	-2.263499	1.744999	1.957948
C	-3.343280	3.275765	2.967821
H	-3.361396	4.003654	2.141980
H	-2.476541	3.502682	3.607945
H	-4.245407	3.432091	3.579714
C	-3.284217	0.815824	3.598668
H	-3.165797	-0.213810	3.229013
H	-4.245561	0.871477	4.133780
H	-2.478654	1.017800	4.322482
C	4.250714	0.221838	0.381780
C	3.743536	0.245431	-0.945690
H	3.673813	1.192132	-1.483758
C	3.438739	-0.955553	-1.629477
H	3.216063	-0.955723	-2.699751
C	3.659648	-2.215933	-0.997163
C	4.175663	-2.235780	0.301442
H	4.361943	-3.178398	0.814482
C	4.462779	-1.029860	0.962102
H	4.862158	-1.083181	1.978721
C	3.541891	-4.592725	-1.187338
H	3.212585	-5.299709	-1.958668
H	2.931675	-4.733036	-0.279836
H	4.604802	-4.775853	-0.955215
N	-2.683656	2.577038	-1.425431
O	3.659355	3.171409	-0.355264
O	2.696913	5.090121	-1.002478
C	3.579395	4.527844	-0.417206
C	4.753914	5.124693	0.342227
H	4.441162	6.026267	0.885065

H	5.508860	5.431917	-0.402046
C	5.237044	3.961510	1.209525
C	4.836747	2.737884	0.370758
H	5.617393	2.512253	-0.378967
C	4.509251	1.487524	1.172576
H	5.342014	1.312043	1.873227
H	3.624497	1.715051	1.794836
H	6.313375	3.978744	1.429054
H	4.693572	3.945539	2.168403

29
PRODUCT SCF Done: -690.908657943 A.U.

O	2.087290	0.707886	-0.003409
O	3.643696	2.325477	-0.045463
O	-4.399121	0.196565	-0.421134
C	3.357186	1.169280	-0.158632
C	4.267086	-0.010472	-0.490120
H	5.242482	0.110836	-0.000483
H	4.437917	-0.001755	-1.580636
C	3.443855	-1.218512	-0.046848
C	2.002717	-0.716101	-0.231128
H	1.672777	-0.862472	-1.276216
C	0.973185	-1.325446	0.715947
H	1.062025	-2.421645	0.635390
H	1.269788	-1.055216	1.744188
C	-0.452971	-0.902975	0.442000
C	-0.887502	0.399419	0.721821
H	-0.183230	1.116936	1.147657
C	-2.196422	0.809040	0.449228
H	-2.490414	1.832465	0.682321
C	-3.107843	-0.095567	-0.118593
C	-2.685737	-1.404382	-0.404663
H	-3.406868	-2.096746	-0.843615
C	-1.378476	-1.794274	-0.127412
H	-1.070954	-2.819377	-0.355753
C	-4.889899	1.493045	-0.165415
H	-5.940415	1.500463	-0.486863
H	-4.337224	2.263864	-0.733881
H	-4.844099	1.747866	0.909699
H	3.638766	-2.137226	-0.618158
H	3.623457	-1.435741	1.019285

12
PhI SCF Done: -242.900927617 A.U.

C	-2.667477	-1.208741	0.000001
C	-1.268172	-1.217177	0.000004
C	-0.582218	-0.000020	-0.000011
C	-1.268163	1.217170	-0.000004
C	-2.667444	1.208761	0.000009

C	-3.369225	0.000005	-0.000005
H	-3.207306	-2.159237	0.000005
H	-0.725073	-2.163922	0.000006
H	-0.725018	2.163888	-0.000003
H	-3.207294	2.159245	0.000013
H	-4.461837	0.000036	-0.000007
I	1.570995	0.000000	0.000000

85

A-ad+11-N SCF Done: -1816.81447381 A.U.

Au	-1.266899	-1.099249	-0.243766
N	1.411119	-3.045513	0.011087
N	0.732129	1.138725	-0.238044
N	1.989716	1.510060	-0.102330
N	2.644012	0.401259	0.142297
C	1.788596	-4.301661	0.252301
H	1.114166	-5.094070	-0.089617
C	2.987677	-4.603728	0.905588
H	3.295303	-5.633830	1.092232
C	3.765256	-3.517498	1.313377
H	4.713000	-3.660758	1.843444
C	2.237300	-2.079506	0.433015
C	1.811992	-0.694519	0.160677
C	0.532006	-0.209044	-0.097367
C	-0.274135	2.136125	-0.521861
C	-0.538186	2.429888	-1.873665
C	-1.515233	3.402023	-2.125766
H	-1.751810	3.675981	-3.154875
C	-2.182251	4.036616	-1.075244
H	-2.931176	4.801509	-1.296067
C	-1.902902	3.703342	0.249497
H	-2.444864	4.198513	1.057353
C	-0.939281	2.733782	0.561889
C	0.240634	1.763618	-3.001014
H	0.611184	0.796185	-2.625128
C	1.467643	2.616080	-3.377056
H	2.061372	2.116074	-4.157948
H	2.119188	2.787913	-2.507209
H	1.153194	3.598654	-3.763641
C	-0.624832	1.452620	-4.229368
H	-0.954336	2.368196	-4.745034
H	-1.520527	0.873086	-3.955284
H	-0.046192	0.863744	-4.957632
C	-0.676067	2.337219	2.008769
H	0.174279	1.638534	2.026169
C	-1.890330	1.592704	2.592634
H	-2.146695	0.708788	1.987983
H	-2.780586	2.240551	2.621114
H	-1.680170	1.258842	3.620930

C	-0.280398	3.544441	2.873565
H	0.593713	4.066796	2.456497
H	-0.027361	3.215150	3.893536
H	-1.103262	4.271796	2.956750
C	4.085143	0.475787	0.279966
C	4.860288	0.322585	-0.880342
C	6.251069	0.406296	-0.717327
H	6.902577	0.287780	-1.584970
C	6.812258	0.641702	0.536890
H	7.897988	0.709120	0.639340
C	6.001545	0.792166	1.664767
H	6.463177	0.974742	2.636145
C	4.608914	0.704601	1.564060
C	4.245247	0.028217	-2.243724
H	3.165426	0.237721	-2.188061
C	4.809980	0.931368	-3.350378
H	4.277680	0.748787	-4.297000
H	5.877450	0.734228	-3.534516
H	4.699553	1.995863	-3.094459
C	4.407799	-1.461582	-2.597317
H	3.963295	-2.111522	-1.828030
H	5.473345	-1.728387	-2.682199
H	3.922905	-1.688053	-3.560128
C	3.696239	0.876155	2.770645
H	2.755950	0.344281	2.550690
C	4.264747	0.244284	4.047538
H	4.539022	-0.808508	3.880860
H	3.515667	0.282752	4.853691
H	5.156257	0.779565	4.410159
C	3.352371	2.362933	2.980610
H	2.897757	2.801919	2.079741
H	4.260497	2.941230	3.214501
H	2.646277	2.483919	3.817551
N	3.389832	-2.257806	1.083679
I	-3.805612	-2.070357	-0.384165
C	-4.667034	-0.256453	0.449632
C	-4.431656	0.954705	-0.197804
C	-5.425663	-0.360695	1.613446
H	-3.814532	1.017187	-1.095542
C	-4.999465	2.110370	0.350229
C	-5.980089	0.811015	2.143798
H	-5.588345	-1.320167	2.107300
H	-4.824658	3.067653	-0.143350
H	-6.579601	0.751306	3.055119
C	-5.770874	2.040817	1.513565
H	-6.211143	2.948778	1.931657

85

TS-AB-cis+11-N SCF Done: -1816.78769304 A.U.

Au	-1.529678	-0.944774	-0.129136
N	0.770452	-2.860182	-0.694280
N	0.648344	1.186024	0.360973
N	1.946674	1.417919	0.414558
N	2.506897	0.300731	0.019796
C	0.970680	-4.127833	-1.052279
H	0.094088	-4.784378	-1.056383
C	2.239645	-4.601045	-1.402393
H	2.404346	-5.640732	-1.689295
C	3.282277	-3.672306	-1.368738
H	4.306344	-3.955233	-1.634490
C	1.839501	-2.049757	-0.690536
C	1.571915	-0.661123	-0.278591
C	0.327938	-0.076363	-0.052722
C	-0.270183	2.225005	0.764947
C	-0.593061	3.220675	-0.176086
C	-1.475174	4.222049	0.248635
H	-1.754039	5.021666	-0.438892
C	-2.002255	4.213120	1.541558
H	-2.683886	5.008606	1.852753
C	-1.666036	3.201377	2.440247
H	-2.084201	3.214339	3.448929
C	-0.782598	2.175389	2.073193
C	0.033472	3.238989	-1.565093
H	0.344591	2.208260	-1.804339
C	1.297216	4.120221	-1.568376
H	1.780967	4.097960	-2.557290
H	2.027541	3.779772	-0.819973
H	1.038173	5.166190	-1.338727
C	-0.947675	3.669849	-2.663272
H	-1.230684	4.729954	-2.567911
H	-1.866837	3.066899	-2.638895
H	-0.480055	3.549912	-3.652803
C	-0.382995	1.099652	3.076713
H	0.282755	0.383696	2.572029
C	-1.598285	0.299812	3.572576
H	-2.123104	-0.189938	2.736804
H	-2.320960	0.941614	4.100769
H	-1.280151	-0.487397	4.273673
C	0.415550	1.707564	4.243025
H	1.303178	2.246997	3.878529
H	0.753941	0.916099	4.930250
H	-0.196318	2.416414	4.823000
C	3.953063	0.217292	0.042964
C	4.661964	0.625074	-1.098225
C	6.058282	0.530041	-1.033847
H	6.656942	0.830612	-1.896012
C	6.694376	0.056389	0.114325
H	7.784727	-0.007807	0.141818

C	5.950551	-0.329997	1.229779
H	6.465065	-0.690127	2.123079
C	4.551378	-0.258621	1.221827
C	3.969995	1.116593	-2.362891
H	2.892179	1.206483	-2.153444
C	4.460919	2.512384	-2.778563
H	3.893731	2.872093	-3.651570
H	5.525256	2.499104	-3.060973
H	4.335482	3.240970	-1.963697
C	4.130977	0.095435	-3.502779
H	3.762658	-0.895956	-3.199802
H	5.189705	-0.012412	-3.788101
H	3.574377	0.421072	-4.395983
C	3.743152	-0.682666	2.442400
H	2.673017	-0.560926	2.212752
C	3.962806	-2.169257	2.769376
H	3.732330	-2.806061	1.901031
H	3.319151	-2.479112	3.607845
H	5.006457	-2.366281	3.060995
C	4.039761	0.220675	3.650971
H	3.845846	1.277953	3.413071
H	5.090606	0.133431	3.969101
H	3.406294	-0.061090	4.506971
N	3.084118	-2.398939	-1.017515
I	-3.844222	-2.268609	0.068207
C	-3.436376	0.156456	-0.679090
C	-3.611325	0.308242	-2.054032
C	-3.813401	1.113884	0.259466
H	-3.346719	-0.483718	-2.754936
C	-4.151104	1.519357	-2.505367
C	-4.347979	2.313340	-0.224070
H	-3.686563	0.950231	1.328778
H	-4.290924	1.667767	-3.578877
H	-4.630965	3.087881	0.491203
C	-4.521451	2.515139	-1.596601
H	-4.954547	3.449560	-1.959606

85

TS-AB-trans+11-N SCF Done: -1816.78473828 A.U.

Au	-1.610747	0.654938	0.000001
N	0.321774	2.774609	-0.000006
N	0.809716	-1.325667	0.000054
N	2.128395	-1.409400	0.000055
N	2.546228	-0.165340	0.000081
C	0.282525	4.105213	0.000030
H	-0.709403	4.569519	-0.000089
C	1.454040	4.869965	0.000211
H	1.426140	5.960572	0.000233
C	2.659543	4.164129	0.000367

H	3.622905	4.684913	0.000526
C	1.532904	2.188383	0.000144
C	1.495606	0.719371	0.000099
C	0.336619	-0.046817	0.000074
C	-0.000176	-2.522440	-0.000010
C	-0.395138	-3.045804	1.243847
C	-1.202694	-4.190402	1.213359
H	-1.533186	-4.644206	2.148547
C	-1.595012	-4.757252	-0.000154
H	-2.223159	-5.651305	-0.000210
C	-1.202352	-4.190492	-1.213595
H	-1.532566	-4.644369	-2.148846
C	-0.394788	-3.045891	-1.243935
C	0.065564	-2.418587	2.553793
H	0.269138	-1.352508	2.359117
C	1.384632	-3.061810	3.021943
H	1.748326	-2.577374	3.941800
H	2.168624	-2.974865	2.254677
H	1.238748	-4.132807	3.234933
C	-1.004984	-2.464863	3.652167
H	-1.191508	-3.492146	4.002070
H	-1.960724	-2.046273	3.299351
H	-0.673534	-1.882983	4.526233
C	0.066271	-2.418795	-2.553811
H	0.270170	-1.352783	-2.359112
C	-1.004186	-2.464717	-3.652293
H	-1.959811	-2.045777	-3.299577
H	-1.191049	-3.491942	-4.002184
H	-0.672446	-1.882978	-4.526343
C	1.385185	-3.062435	-3.021814
H	2.169114	-2.975717	-2.254457
H	1.749131	-2.578120	-3.941634
H	1.238993	-4.133388	-3.234811
C	3.971202	0.090681	0.000002
C	4.616521	0.207110	1.242643
C	5.991508	0.474398	1.211694
H	6.539776	0.577400	2.150239
C	6.669434	0.610272	-0.000163
H	7.741888	0.819105	-0.000228
C	5.991345	0.474477	-1.211938
H	6.539488	0.577544	-2.150548
C	4.616356	0.207182	-1.242722
C	3.876169	0.082896	2.568684
H	2.841983	-0.233562	2.360561
C	4.494342	-0.996572	3.471204
H	3.898947	-1.112226	4.390452
H	5.519881	-0.733344	3.773906
H	4.530809	-1.971370	2.961856
C	3.803060	1.444643	3.281775

H	3.342325	2.207880	2.636397
H	4.809169	1.801836	3.553719
H	3.212778	1.365776	4.208585
C	3.875819	0.083073	-2.568671
H	2.841735	-0.233655	-2.360445
C	3.802305	1.444958	-3.281460
H	3.341449	2.207950	-2.635880
H	3.211955	1.366150	-4.208233
H	4.808305	1.802451	-3.553413
C	4.494066	-0.996063	-3.471535
H	4.530891	-1.970950	-2.962386
H	5.519468	-0.732518	-3.774427
H	3.898492	-1.111686	-4.390671
N	2.701234	2.828409	0.000339
I	-3.853200	-0.931965	0.000130
C	-3.444054	1.683835	-0.000113
C	-3.781380	2.277765	-1.228087
C	-3.781400	2.278000	1.227741
H	-3.584106	1.763933	-2.170780
C	-4.364112	3.548006	-1.216570
C	-4.364131	3.548239	1.215972
H	-3.584141	1.764348	2.170536
H	-4.609455	4.035457	-2.163249
H	-4.609490	4.035870	2.162553
C	-4.654634	4.179219	-0.000362
H	-5.134706	5.160670	-0.000460

85

B-cis+11-N SCF Done: -1816.81979362 A.U.

Au	1.547016	-0.976775	-0.096567
N	-0.089604	-2.602252	-0.140502
N	-0.653690	1.404946	0.089957
N	-1.977553	1.478660	0.142155
N	-2.405119	0.241565	0.091703
C	0.050643	-3.929488	-0.211887
H	1.074918	-4.308769	-0.278676
C	-1.070296	-4.762850	-0.199188
H	-0.965614	-5.846939	-0.258360
C	-2.320018	-4.146626	-0.106271
H	-3.243406	-4.734419	-0.087950
C	-1.345771	-2.095835	-0.055996
C	-1.361243	-0.635642	0.013164
C	-0.198169	0.123733	0.011448
C	0.104350	2.641183	0.090858
C	0.687011	3.059845	1.303212
C	1.406547	4.259927	1.263295
H	1.889418	4.630636	2.167149
C	1.513412	4.995736	0.081812
H	2.076616	5.931833	0.079574

C	0.915595	4.548026	-1.094366
H	1.015495	5.138444	-2.006099
C	0.198346	3.344535	-1.122872
C	0.485835	2.277576	2.597250
H	0.523747	1.204535	2.341619
C	-0.904012	2.576121	3.196683
H	-1.082300	1.953821	4.087739
H	-1.718895	2.392843	2.482469
H	-0.967772	3.632335	3.502385
C	1.577475	2.523647	3.645460
H	1.517869	3.541499	4.061776
H	2.582787	2.382315	3.225466
H	1.452549	1.824538	4.486472
C	-0.472378	2.853923	-2.400891
H	-0.625240	1.764961	-2.306316
C	0.387033	3.070775	-3.655323
H	1.400155	2.657645	-3.536356
H	0.485917	4.138763	-3.903335
H	-0.082077	2.581294	-4.522653
C	-1.857117	3.508710	-2.566062
H	-2.507139	3.303592	-1.703090
H	-2.356512	3.134952	-3.473035
H	-1.755358	4.601502	-2.659731
C	-3.827978	-0.030721	0.131946
C	-4.411150	-0.265956	1.388607
C	-5.783223	-0.550233	1.396580
H	-6.286433	-0.738760	2.346983
C	-6.513723	-0.594891	0.209299
H	-7.582332	-0.820270	0.239429
C	-5.894327	-0.348743	-1.016967
H	-6.484966	-0.385444	-1.933890
C	-4.526606	-0.054701	-1.088799
C	-3.616686	-0.233843	2.688173
H	-2.578092	0.049743	2.456211
C	-4.158952	0.831341	3.655014
H	-3.531952	0.882065	4.558789
H	-5.186227	0.598127	3.975822
H	-4.168767	1.828037	3.188223
C	-3.571527	-1.625883	3.341924
H	-3.171760	-2.379612	2.646319
H	-4.577244	-1.954734	3.647824
H	-2.937698	-1.608976	4.242542
C	-3.841190	0.173415	-2.430928
H	-2.854077	0.623440	-2.241216
C	-3.608457	-1.167403	-3.151328
H	-3.033795	-1.867051	-2.525799
H	-3.061935	-1.011953	-4.094945
H	-4.568166	-1.652354	-3.391152
C	-4.609051	1.160540	-3.322665

H	-4.784126	2.116260	-2.806650
H	-5.584763	0.756367	-3.633549
H	-4.036052	1.365312	-4.240306
C	2.860312	0.589576	-0.009669
C	3.601324	0.794927	1.153598
H	3.514893	0.122017	2.007947
C	4.488285	1.878186	1.201753
H	5.079537	2.043684	2.105830
C	3.881440	2.498175	-1.055995
H	3.983723	3.158634	-1.920205
C	2.991431	1.419531	-1.121145
H	2.414354	1.244258	-2.030011
I	3.602811	-2.662963	-0.283594
N	-2.455767	-2.816831	-0.034499
C	4.627308	2.729245	0.102561
H	5.322751	3.570334	0.146620

85
TS-decomp-cis+11-N SCF Done: -1816.78599006 A.U.

Au	-1.946313	-0.481944	-0.407246
N	0.199238	-2.106374	-2.080397
N	0.489020	1.145369	0.708938
N	1.744226	0.914145	1.033403
N	2.092841	-0.158713	0.371457
C	0.189190	-3.283676	-2.709194
H	-0.565541	-3.419906	-3.490439
C	1.097672	-4.295218	-2.385381
H	1.091082	-5.256863	-2.900718
C	2.008326	-4.014592	-1.362990
H	2.750641	-4.752831	-1.041711
C	1.109594	-1.942471	-1.114668
C	1.064913	-0.657778	-0.386796
C	-0.010323	0.218485	-0.178661
C	-0.168995	2.305952	1.269245
C	0.161178	3.567955	0.727422
C	-0.523728	4.669466	1.253998
H	-0.312458	5.668269	0.870927
C	-1.478576	4.510208	2.260372
H	-2.004210	5.385283	2.649801
C	-1.756482	3.248762	2.784739
H	-2.485319	3.150198	3.590398
C	-1.097452	2.105411	2.307771
C	1.231002	3.741510	-0.346027
H	1.269788	2.807141	-0.932043
C	2.613189	3.963560	0.301279
H	3.384643	4.067478	-0.477055
H	2.898013	3.135093	0.963677
H	2.607956	4.889114	0.898449
C	0.928659	4.875039	-1.336430

H	1.027577	5.865015	-0.864799
H	-0.081863	4.792165	-1.757832
H	1.648687	4.843069	-2.168440
C	-1.315885	0.753213	2.976403
H	-0.932470	-0.035162	2.310744
C	-2.796978	0.441252	3.230057
H	-3.394368	0.534755	2.311074
H	-3.229754	1.105694	3.993690
H	-2.907804	-0.591649	3.592263
C	-0.490499	0.680857	4.275495
H	0.579667	0.851537	4.084212
H	-0.602786	-0.308796	4.745775
H	-0.829585	1.440775	4.997259
C	3.470283	-0.606826	0.462157
C	4.366302	-0.142442	-0.516876
C	5.688943	-0.594480	-0.411663
H	6.429009	-0.271036	-1.145292
C	6.075943	-1.444138	0.625357
H	7.114296	-1.777665	0.690680
C	5.153444	-1.866645	1.584130
H	5.479324	-2.524413	2.391634
C	3.814880	-1.461734	1.521032
C	3.931344	0.778959	-1.651564
H	2.986692	1.261420	-1.354418
C	4.936896	1.910827	-1.911320
H	4.516751	2.631917	-2.629745
H	5.875887	1.532267	-2.343894
H	5.182899	2.450263	-0.984775
C	3.657065	-0.022157	-2.937738
H	2.894160	-0.800005	-2.783026
H	4.574025	-0.523210	-3.286179
H	3.306250	0.644145	-3.741765
C	2.798705	-1.899911	2.565381
H	1.791790	-1.694893	2.166922
C	2.858430	-3.407475	2.848362
H	2.756259	-3.985919	1.918017
H	2.042218	-3.697726	3.528028
H	3.803673	-3.696954	3.333302
C	2.965123	-1.069480	3.850906
H	2.883164	0.008592	3.643087
H	3.952183	-1.249093	4.306074
H	2.194929	-1.339511	4.590213
C	-1.262700	1.124996	-1.626274
C	-0.728009	0.860843	-2.889739
H	-0.213258	-0.080538	-3.079511
C	-0.908943	1.816313	-3.894490
H	-0.504385	1.625301	-4.891226
C	-2.129147	3.238782	-2.354595
H	-2.685304	4.155569	-2.146578

C	-1.938009	2.313762	-1.322617
H	-2.339292	2.511238	-0.329276
I	-3.841485	-2.153770	0.206451
N	2.010124	-2.843277	-0.722727
C	-1.612887	2.996621	-3.631508
H	-1.758664	3.732697	-4.425182

100

TS-CD-cis+11-N SCF Done: -3210.36383458 A.U.

Au	-1.349108	-0.252445	-1.299970
N	0.276729	-1.729532	-1.839209
N	0.624702	1.498348	0.562052
N	1.898850	1.426745	0.926876
N	2.394509	0.396940	0.279731
C	0.182468	-2.864518	-2.543037
H	-0.781780	-3.116017	-2.977079
C	1.332112	-3.629938	-2.810520
H	1.259877	-4.541759	-3.402682
C	2.525246	-3.204736	-2.252636
H	3.456736	-3.757114	-2.407271
C	1.492000	-1.415063	-1.310956
C	1.444697	-0.201004	-0.496118
C	0.277739	0.529892	-0.328260
C	-0.196931	2.540502	1.148071
C	-0.148729	3.812688	0.552884
C	-0.907848	4.820058	1.163934
H	-0.906685	5.826651	0.743945
C	-1.662951	4.551837	2.304206
H	-2.242813	5.352785	2.768907
C	-1.698814	3.268491	2.851692
H	-2.314063	3.081612	3.731713
C	-0.970656	2.217549	2.281127
C	0.720273	4.099345	-0.665846
H	0.884724	3.144064	-1.193024
C	2.096875	4.636768	-0.230197
H	2.741779	4.806249	-1.106396
H	2.609583	3.937387	0.446407
H	1.984238	5.595849	0.299857
C	0.050688	5.047132	-1.670946
H	-0.033955	6.070554	-1.273631
H	-0.958433	4.700758	-1.937628
H	0.653240	5.107998	-2.590522
C	-1.017728	0.802493	2.851458
H	-1.017172	0.104610	1.996957
C	-2.293498	0.499752	3.649014
H	-3.198975	0.797988	3.100509
H	-2.300921	1.021129	4.619393
H	-2.368638	-0.580608	3.838327
C	0.221754	0.509393	3.719423

H	1.165151	0.694061	3.188072
H	0.213658	-0.538847	4.061368
H	0.223354	1.148502	4.616499
C	3.795697	0.061842	0.425084
C	4.708896	0.704266	-0.431080
C	6.056559	0.350798	-0.285237
H	6.806567	0.815638	-0.927205
C	6.454884	-0.588696	0.666401
H	7.512073	-0.846706	0.762775
C	5.515885	-1.199602	1.497170
H	5.847800	-1.929215	2.238609
C	4.153540	-0.889440	1.394694
C	4.264491	1.683946	-1.510823
H	3.242010	2.017092	-1.274594
C	5.134580	2.948037	-1.560431
H	4.722068	3.660972	-2.291030
H	6.165825	2.723898	-1.874163
H	5.175801	3.447068	-0.580616
C	4.217122	0.978086	-2.878962
H	3.578275	0.082534	-2.848239
H	5.224749	0.652904	-3.183549
H	3.831502	1.659400	-3.653936
C	3.136366	-1.570358	2.302408
H	2.139714	-1.162052	2.068809
C	3.089612	-3.086410	2.040532
H	2.819542	-3.308409	0.996692
H	2.368423	-3.576718	2.717689
H	4.064550	-3.557623	2.237349
C	3.408014	-1.258826	3.783304
H	3.431251	-0.174081	3.965340
H	4.372702	-1.677198	4.109827
H	2.622860	-1.694362	4.421604
C	-2.610851	1.310878	-0.941831
C	-2.671687	2.344638	-1.880656
H	-2.047799	2.334281	-2.777203
C	-3.554311	3.410411	-1.661182
H	-3.611612	4.219132	-2.394162
C	-4.274981	2.401616	0.415580
H	-4.889512	2.423251	1.319134
C	-3.402676	1.326490	0.204842
H	-3.329261	0.514591	0.926797
N	2.603088	-2.100630	-1.479944
O	-2.331064	-1.708502	0.822780
O	-2.935832	-2.725287	2.714526
C	-3.175942	-2.323726	1.566354
C	-4.522351	-2.638311	0.906647
H	-5.343270	-2.341151	1.575426
H	-4.574397	-3.736268	0.820756
C	-4.617647	-1.974198	-0.462915

C -3.380564 -2.142514 -1.262860
H -2.813231 -3.079298 -1.139935
C -3.009599 -1.279731 -2.320396
H -2.478070 -1.749231 -3.157800
H -3.736557 -0.515940 -2.617388
H -5.417530 -2.443464 -1.073569
H -4.882016 -0.905990 -0.401471
K -0.518030 -3.468844 1.938061
Cl -0.924414 -4.605708 -0.702806
C -4.353248 3.441563 -0.514417
H -5.035128 4.278105 -0.345551

100
TS-CD-trans+11-N SCF Done: -3210.35734769 A.U.
Au -1.553431 -0.646469 -0.663551
N -0.371870 -2.321267 0.041260
N 1.042617 1.424446 -0.378788
N 2.349735 1.254441 -0.196177
N 2.510855 -0.038084 -0.043563
C -0.828498 -3.526952 0.392408
H -1.904464 -3.691035 0.315059
C 0.054858 -4.513441 0.827308
H -0.309772 -5.495072 1.131021
C 1.415092 -4.196532 0.823234
H 2.170640 -4.937075 1.104934
C 0.965965 -2.074110 0.151976
C 1.316270 -0.689091 -0.120692
C 0.338181 0.263779 -0.362503
C 0.512724 2.762456 -0.513192
C 0.410615 3.305097 -1.809031
C -0.167688 4.578074 -1.912976
H -0.274855 5.047383 -2.891543
C -0.599523 5.261230 -0.774521
H -1.047404 6.252521 -0.878279
C -0.455232 4.699739 0.495171
H -0.789957 5.259524 1.368741
C 0.111794 3.427526 0.664522
C 0.976343 2.575439 -3.023936
H 0.888249 1.490840 -2.836387
C 2.478326 2.891033 -3.177651
H 2.905575 2.329713 -4.022947
H 3.042320 2.630012 -2.271019
H 2.625043 3.965496 -3.370496
C 0.227876 2.874430 -4.330075
H 0.392685 3.910003 -4.665336
H -0.859383 2.725338 -4.234194
H 0.593266 2.215617 -5.132588
C 0.346480 2.819600 2.041956
H 0.221216 1.726620 1.967748

C	-0.634043	3.312612	3.112994
H	-1.686793	3.170081	2.831321
H	-0.480747	4.376894	3.353540
H	-0.454874	2.755670	4.046721
C	1.796319	3.085128	2.497251
H	2.533258	2.730678	1.764185
H	1.988943	2.564434	3.447220
H	1.964352	4.163476	2.650956
C	3.850168	-0.589158	0.033847
C	4.461517	-0.939722	-1.186699
C	5.747524	-1.486802	-1.108778
H	6.264781	-1.781250	-2.023580
C	6.379846	-1.658212	0.124537
H	7.385451	-2.084082	0.161609
C	5.741846	-1.286478	1.306277
H	6.252283	-1.422284	2.261736
C	4.449399	-0.740541	1.293932
C	3.754700	-0.785301	-2.529689
H	2.903337	-0.099848	-2.395358
C	4.652501	-0.153963	-3.603915
H	4.073430	0.027405	-4.523223
H	5.492605	-0.811819	-3.874938
H	5.068308	0.806550	-3.264443
C	3.188007	-2.138477	-2.997422
H	2.522968	-2.580545	-2.239794
H	4.001725	-2.858649	-3.178750
H	2.622191	-2.022275	-3.935985
C	3.758935	-0.348833	2.590582
H	2.747749	0.014809	2.360054
C	3.572248	-1.565672	3.510621
H	3.032340	-2.367772	2.988366
H	2.965656	-1.280987	4.382527
H	4.538798	-1.959372	3.865900
C	4.516428	0.785728	3.299476
H	4.625651	1.667544	2.649672
H	5.525337	0.467616	3.608252
H	3.972708	1.094272	4.206031
C	-3.152161	-1.920149	-1.037370
C	-4.198683	-2.043037	-0.117160
H	-4.233590	-1.382270	0.751411
C	-5.194584	-3.005183	-0.338568
H	-6.014321	-3.104318	0.378009
C	-4.105167	-3.689023	-2.389393
H	-4.065505	-4.325467	-3.277192
C	-3.102842	-2.732231	-2.177398
H	-2.286747	-2.641383	-2.900223
N	1.862575	-2.987699	0.477902
O	-3.077665	0.448147	1.265313
O	-3.758300	1.839124	2.879443

C	-3.582891	1.543859	1.685933
C	-4.031288	2.550046	0.617711
H	-3.984244	3.563959	1.037661
H	-5.096869	2.346400	0.410082
C	-3.214174	2.456451	-0.664573
C	-3.269722	1.152205	-1.364559
H	-4.195246	0.572598	-1.275594
C	-2.310491	0.711444	-2.272826
H	-2.593799	-0.028238	-3.027414
H	-1.476713	1.372921	-2.516052
H	-3.622120	3.167760	-1.414471
H	-2.170413	2.764976	-0.512469
K	-2.324829	-0.105230	3.896451
Cl	0.224202	-0.891808	2.826846
C	-5.148861	-3.827136	-1.469245
H	-5.930060	-4.572253	-1.636793

15
PhCH3I SCF Done: -282.192694281 A.U.

C	-2.267661	1.201948	-0.011332
C	-0.869654	1.213144	-0.005661
C	-0.178422	0.000130	-0.001830
C	-0.869811	-1.212927	-0.005665
C	-2.267691	-1.201605	-0.011336
C	-2.991925	0.000253	-0.011394
H	-2.803231	2.155760	-0.017782
H	-0.331782	2.162932	-0.007526
H	-0.331951	-2.162723	-0.007529
H	-2.803414	-2.155348	-0.017814
I	1.974494	-0.000054	0.002394
C	-4.500518	-0.000247	0.017547
H	-4.915153	-0.886277	-0.487658
H	-4.872961	-0.012255	1.057097
H	-4.915621	0.896599	-0.467643

88
A-ad+12-N SCF Done: -1856.10739792 A.U.

Au	1.088513	1.204601	-0.472809
N	-1.527546	2.997125	0.358522
N	-0.791850	-1.127047	-0.266342
N	-2.018046	-1.551688	-0.031756
N	-2.728292	-0.461248	0.120252
C	-1.963149	4.248367	0.507459
H	-1.203265	5.018074	0.680282
C	-3.322426	4.572450	0.451474
H	-3.674055	5.597045	0.582181
C	-4.203588	3.516124	0.210560
H	-5.284178	3.679092	0.136772
C	-2.457343	2.056977	0.143888

C	-1.962195	0.675490	-0.003991
C	-0.663584	0.236476	-0.258240
C	0.261520	-2.096375	-0.462696
C	0.491341	-2.562467	-1.770816
C	1.502641	-3.519566	-1.927766
H	1.714894	-3.923096	-2.918867
C	2.234735	-3.974946	-0.828907
H	3.009009	-4.732733	-0.973593
C	1.990248	-3.470190	0.447640
H	2.583496	-3.825063	1.292638
C	0.995498	-2.506191	0.663364
C	-0.357095	-2.089428	-2.944281
H	-0.765262	-1.099628	-2.682483
C	-1.549293	-3.041395	-3.157231
H	-2.198973	-2.673928	-3.967054
H	-2.156621	-3.134055	-2.244866
H	-1.196955	-4.048370	-3.432577
C	0.451214	-1.904232	-4.235494
H	0.812691	-2.863925	-4.637000
H	1.322984	-1.250358	-4.075606
H	-0.180755	-1.447800	-5.012835
C	0.753946	-1.936402	2.055028
H	-0.048795	-1.187014	1.986870
C	2.002986	-1.204484	2.573977
H	2.323586	-0.417204	1.874332
H	2.852026	-1.893361	2.704325
H	1.796825	-0.737228	3.549754
C	0.273343	-3.021155	3.032553
H	-0.640316	-3.512387	2.664000
H	0.051487	-2.579634	4.016928
H	1.039670	-3.798734	3.179874
C	-4.120421	-0.604214	0.495582
C	-5.073655	-0.770124	-0.521595
C	-6.407118	-0.905175	-0.113908
H	-7.187432	-1.034326	-0.866664
C	-6.752880	-0.873200	1.237605
H	-7.799889	-0.978633	1.531629
C	-5.771976	-0.714568	2.216938
H	-6.058750	-0.700185	3.270510
C	-4.421640	-0.578983	1.868155
C	-4.706217	-0.780454	-1.999318
H	-3.610030	-0.714139	-2.082931
C	-5.129222	-2.093515	-2.677515
H	-4.793402	-2.108484	-3.726300
H	-6.224363	-2.210741	-2.679254
H	-4.697172	-2.967102	-2.166218
C	-5.291869	0.446249	-2.719653
H	-4.967283	1.377674	-2.233022
H	-6.393374	0.423682	-2.709904

H	-4.967607	0.466486	-3.772324
C	-3.355441	-0.403187	2.943489
H	-2.369212	-0.366627	2.455681
C	-3.536845	0.929386	3.690318
H	-3.530811	1.782454	2.994007
H	-2.727498	1.075931	4.423018
H	-4.492641	0.952396	4.237392
C	-3.321357	-1.599411	3.908670
H	-3.164638	-2.543797	3.365151
H	-4.260477	-1.688064	4.477042
H	-2.502315	-1.481836	4.635941
N	-3.773532	2.262796	0.052229
I	3.571550	2.308346	-0.669505
C	4.511242	0.564289	0.219316
C	4.384566	-0.665292	-0.423132
C	5.190974	0.710672	1.425030
H	3.826799	-0.771797	-1.354845
C	4.973834	-1.782087	0.173640
C	5.767911	-0.427274	1.999762
H	5.271893	1.677954	1.923806
H	4.871902	-2.750914	-0.319543
C	5.672259	-1.686605	1.388073
H	6.300009	-0.323951	2.949019
C	6.314580	-2.903922	2.002114
H	7.299989	-3.095044	1.543796
H	6.472715	-2.780405	3.083365
H	5.703138	-3.805437	1.842355

88

TS-AB-cis+12-N SCF Done: -1856.08158424 A.U.

Au	-1.368714	-1.146486	-0.110770
N	1.047267	-2.825379	-0.798673
N	0.640992	1.137787	0.466674
N	1.919678	1.469230	0.501468
N	2.555412	0.421019	0.038334
C	1.327467	-4.050424	-1.241139
H	0.505008	-4.773637	-1.247089
C	2.611101	-4.398993	-1.674248
H	2.842328	-5.404003	-2.030397
C	3.578278	-3.392266	-1.630938
H	4.606934	-3.575801	-1.958847
C	2.048761	-1.932787	-0.791734
C	1.690492	-0.595927	-0.287625
C	0.411013	-0.124954	-0.003438
C	-0.344332	2.091985	0.924832
C	-0.735518	3.110974	0.038315
C	-1.665109	4.041705	0.522531
H	-1.995259	4.858181	-0.121254
C	-2.170922	3.941554	1.818190

H	-2.889577	4.682584	2.176795
C	-1.772039	2.903286	2.661309
H	-2.182604	2.844601	3.670142
C	-0.842726	1.945555	2.233905
C	-0.136373	3.236304	-1.356945
H	0.247488	2.244047	-1.647279
C	1.054905	4.212589	-1.339689
H	1.516897	4.276097	-2.336933
H	1.825246	3.894289	-0.622404
H	0.721076	5.222853	-1.053633
C	-1.168607	3.638109	-2.419324
H	-1.529118	4.668332	-2.271926
H	-2.037501	2.964479	-2.406964
H	-0.713218	3.597640	-3.420874
C	-0.331639	0.853265	3.166545
H	-0.014849	0.000893	2.545700
C	-1.401789	0.316882	4.125432
H	-2.301164	-0.016297	3.583728
H	-1.709149	1.069760	4.867738
H	-1.007002	-0.544945	4.684891
C	0.908763	1.353778	3.932004
H	1.696617	1.695793	3.244196
H	1.326425	0.551974	4.560919
H	0.645233	2.199623	4.586741
C	4.003751	0.453118	-0.000781
C	4.624647	0.976042	-1.147208
C	6.025530	0.969718	-1.158166
H	6.557127	1.360663	-2.027897
C	6.750830	0.468203	-0.076947
H	7.842939	0.471305	-0.109105
C	6.093420	-0.030036	1.048063
H	6.676977	-0.410761	1.888646
C	4.694068	-0.046948	1.116773
C	3.839411	1.494100	-2.345457
H	2.772543	1.523031	-2.073001
C	4.242440	2.929521	-2.718327
H	3.608527	3.302457	-3.538172
H	5.286958	2.980214	-3.063290
H	4.134293	3.612523	-1.862610
C	3.981387	0.538273	-3.543257
H	3.675763	-0.483277	-3.273242
H	5.026629	0.494771	-3.889070
H	3.362218	0.880895	-4.387612
C	3.983156	-0.606918	2.343354
H	2.902974	-0.422005	2.235311
C	4.177323	-2.130213	2.443980
H	3.835434	-2.639715	1.530294
H	3.615190	-2.536837	3.299588
H	5.239534	-2.384054	2.588321

C	4.423417	0.104327	3.632866
H	4.271616	1.192110	3.561818
H	5.487521	-0.075171	3.851839
H	3.842854	-0.267812	4.491468
N	3.299015	-2.161182	-1.194577
I	-3.567427	-2.663597	-0.017555
C	-3.346878	-0.140583	-0.511745
C	-3.565233	0.149877	-1.856775
C	-3.773934	0.683412	0.525665
H	-3.271985	-0.541807	-2.646808
C	-4.177449	1.369806	-2.163026
C	-4.380874	1.893601	0.180772
H	-3.626647	0.412257	1.570239
H	-4.343123	1.620802	-3.214258
H	-4.696321	2.563440	0.983570
C	-4.587376	2.261541	-1.158576
C	-5.195599	3.593843	-1.509293
H	-5.764884	3.547695	-2.449422
H	-5.865368	3.957901	-0.716518
H	-4.401704	4.349689	-1.644026

88

TS-AB-trans+12-N SCF Done: -1856.07869825 A.U.

Au	-1.563635	0.376854	0.004272
N	0.105588	2.713826	0.001415
N	1.079199	-1.300463	0.003911
N	2.398500	-1.225413	-0.000020
N	2.664061	0.059657	-0.001347
C	-0.090838	4.030281	-0.000107
H	-1.130909	4.373548	0.000937
C	0.981776	4.928531	-0.002996
H	0.825026	6.008176	-0.004273
C	2.262344	4.370305	-0.004125
H	3.157363	5.001380	-0.006298
C	1.377411	2.274959	0.000023
C	1.514807	0.811945	0.001455
C	0.455514	-0.087521	0.004866
C	0.419551	-2.586344	0.006334
C	0.100047	-3.156345	1.251387
C	-0.565668	-4.388876	1.223490
H	-0.832622	-4.880664	2.159718
C	-0.897001	-4.995988	0.011358
H	-1.414420	-5.958371	0.013303
C	-0.583616	-4.384100	-1.203132
H	-0.864422	-4.872182	-2.137239
C	0.081604	-3.151441	-1.236067
C	0.495209	-2.483094	2.559931
H	0.561228	-1.398919	2.369931
C	1.890144	-2.959192	3.007793

H	2.202353	-2.437887	3.926395
H	2.647470	-2.771698	2.231866
H	1.881148	-4.041064	3.215522
C	-0.545992	-2.668864	3.671623
H	-0.597125	-3.713199	4.017134
H	-1.551434	-2.371923	3.334248
H	-0.278180	-2.054286	4.545119
C	0.457292	-2.473114	-2.547715
H	0.525592	-1.389677	-2.354834
C	-0.600178	-2.654908	-3.644560
H	-1.600240	-2.358551	-3.291002
H	-0.656881	-3.698029	-3.992858
H	-0.345079	-2.037421	-4.519817
C	1.845932	-2.946721	-3.017178
H	2.614069	-2.761702	-2.251283
H	2.144918	-2.421775	-3.938127
H	1.834725	-4.027816	-3.228788
C	4.048012	0.484676	-0.006471
C	4.679104	0.678871	1.233712
C	6.011615	1.110279	1.197773
H	6.546711	1.279296	2.134355
C	6.663831	1.326326	-0.016585
H	7.703168	1.663291	-0.020597
C	6.002812	1.108574	-1.225862
H	6.531101	1.276189	-2.166559
C	4.670057	0.677197	-1.251484
C	3.963594	0.466756	2.562213
H	2.975540	0.025393	2.357463
C	4.712239	-0.527171	3.464152
H	4.138390	-0.713197	4.385557
H	5.698713	-0.139679	3.763129
H	4.866209	-1.491088	2.956101
C	3.725942	1.810233	3.274102
H	3.173421	2.510275	2.629213
H	4.681382	2.288466	3.542964
H	3.152111	1.660806	4.202557
C	3.945033	0.463023	-2.574498
H	2.957705	0.023750	-2.361959
C	3.704508	1.805058	-3.288107
H	3.157394	2.507509	-2.641204
H	3.124088	1.654144	-4.212218
H	4.658719	2.281275	-3.564776
C	4.685944	-0.534408	-3.478966
H	4.841716	-1.497304	-2.969494
H	5.671083	-0.149338	-3.785391
H	4.105642	-0.721834	-4.396027
N	2.461794	3.049000	-0.002637
I	-3.566239	-1.505455	-0.017007
C	-3.506162	1.160011	0.005305

C -3.938979 1.706430 -1.214929
C -3.945579 1.685403 1.232441
H -3.661874 1.243828 -2.164196
C -4.714266 2.865743 -1.193170
C -4.720778 2.844937 1.226464
H -3.673632 1.206486 2.175090
H -5.025317 3.314457 -2.140510
H -5.036922 3.277321 2.179695
C -5.122023 3.450957 0.020682
C -6.018546 4.660419 0.028902
H -5.860499 5.277908 0.925557
H -5.863588 5.288026 -0.861192
H -7.078205 4.350495 0.029126

88
B-cis+12-N SCF Done: -1856.11324539 A.U.
Au 1.312950 -1.230452 -0.108938
N -0.508530 -2.646951 -0.142312
N -0.583232 1.400460 0.096794
N -1.888920 1.632373 0.153520
N -2.462635 0.456383 0.095243
C -0.530312 -3.981293 -0.213571
H 0.440749 -4.481930 -0.276855
C -1.744316 -4.672189 -0.205091
H -1.772245 -5.760976 -0.263975
C -2.910100 -3.908377 -0.116929
H -3.898179 -4.379527 -0.102496
C -1.693849 -1.991297 -0.062528
C -1.532314 -0.539759 0.008174
C -0.285950 0.073707 0.006998
C 0.314698 2.540205 0.112010
C 0.951643 2.866377 1.325769
C 1.801146 3.978857 1.301284
H 2.330961 4.273529 2.206826
C 1.977335 4.724599 0.134431
H 2.639003 5.593996 0.145289
C 1.322900 4.370646 -1.043487
H 1.479370 4.965226 -1.944476
C 0.476428 3.254727 -1.088008
C 0.674689 2.087006 2.607513
H 0.597671 1.020829 2.332991
C -0.672479 2.521414 3.221360
H -0.913539 1.903511 4.100613
H -1.505525 2.443145 2.509111
H -0.620187 3.571645 3.549085
C 1.790016 2.197591 3.653773
H 1.839847 3.208597 4.087792
H 2.773117 1.957700 3.226439
H 1.594368 1.501014 4.483309

C	-0.250970	2.867027	-2.370267
H	-0.527002	1.801228	-2.293307
C	0.624627	3.005768	-3.624750
H	1.584564	2.478703	-3.516177
H	0.843291	4.059644	-3.855941
H	0.101057	2.586924	-4.497893
C	-1.551713	3.679101	-2.517722
H	-2.218851	3.534249	-1.655625
H	-2.092696	3.381519	-3.429007
H	-1.325527	4.754543	-2.592642
C	-3.908138	0.358091	0.134243
C	-4.516592	0.181890	1.388516
C	-5.913121	0.067251	1.394494
H	-6.436017	-0.068587	2.343268
C	-6.642920	0.125091	0.207404
H	-7.731081	0.031658	0.235841
C	-5.997301	0.306952	-1.016569
H	-6.587469	0.352412	-1.933377
C	-4.603868	0.432527	-1.086206
C	-3.725290	0.104566	2.687948
H	-2.658548	0.250381	2.456554
C	-4.124496	1.228392	3.658132
H	-3.497385	1.193857	4.562602
H	-5.173984	1.130314	3.976892
H	-4.003494	2.219378	3.194754
C	-3.862019	-1.283454	3.337347
H	-3.564886	-2.080512	2.638689
H	-4.901489	-1.479299	3.644436
H	-3.230116	-1.352382	4.236836
C	-3.894473	0.587592	-2.426343
H	-2.862587	0.920285	-2.232966
C	-3.816100	-0.767710	-3.153061
H	-3.327036	-1.531986	-2.530363
H	-3.253634	-0.671742	-4.095240
H	-4.824909	-1.137714	-3.396224
C	-4.541845	1.660589	-3.314369
H	-4.609133	2.627070	-2.793123
H	-5.555900	1.371761	-3.631061
H	-3.945710	1.804082	-4.228895
C	2.789873	0.180933	-0.050340
C	3.567475	0.322893	1.095838
H	3.444454	-0.341345	1.952492
C	4.535080	1.334750	1.131364
H	5.147395	1.446831	2.030378
C	3.952892	2.000120	-1.105187
H	4.093196	2.650105	-1.973089
C	2.981320	0.998122	-1.162569
H	2.382584	0.874590	-2.066176
I	3.155097	-3.146105	-0.291210

N	-2.883490	-2.571919	-0.045431
C	4.737071	2.194526	0.042752
C	5.738347	3.319256	0.103804
H	6.453079	3.187600	0.929008
H	6.308732	3.403592	-0.834078
H	5.223908	4.283989	0.258432

88

TS-decomp-cis+12-N SCF Done: -1856.08044464 A.U.

Au	-1.878521	-0.642582	-0.374060
N	0.304956	-1.687826	-2.417261
N	0.476780	0.910239	0.995812
N	1.760895	0.723739	1.223618
N	2.156314	-0.155908	0.340084
C	0.352198	-2.701943	-3.283760
H	-0.419667	-2.724854	-4.059807
C	1.339073	-3.688498	-3.204593
H	1.379127	-4.516184	-3.914314
C	2.265863	-3.564552	-2.165898
H	3.068494	-4.296532	-2.027171
C	1.237278	-1.666013	-1.458702
C	1.132331	-0.572165	-0.471797
C	0.005763	0.147092	-0.048666
C	-0.237450	1.875655	1.803156
C	-0.028299	3.244147	1.521162
C	-0.764090	4.156890	2.285826
H	-0.645227	5.226353	2.109425
C	-1.654019	3.720536	3.269534
H	-2.221318	4.453131	3.848490
C	-1.814743	2.360847	3.530596
H	-2.495140	2.041822	4.321454
C	-1.098967	1.394999	2.806534
C	0.968533	3.718708	0.468276
H	1.041492	2.930289	-0.300374
C	2.364077	3.915042	1.094809
H	3.083376	4.238252	0.326853
H	2.742704	2.994469	1.558786
H	2.324616	4.695035	1.871551
C	0.535329	5.003257	-0.253281
H	0.595597	5.881775	0.407649
H	-0.490430	4.929872	-0.638063
H	1.206999	5.195286	-1.104076
C	-1.195570	-0.078205	3.184869
H	-0.767490	-0.680423	2.368922
C	-2.643016	-0.551559	3.378021
H	-3.266290	-0.320328	2.501532
H	-3.108048	-0.092083	4.263794
H	-2.667378	-1.642059	3.521638
C	-0.339840	-0.348412	4.437206

H	0.709141	-0.055175	4.280738
H	-0.363660	-1.419387	4.693378
H	-0.722617	0.216257	5.302115
C	3.566247	-0.496379	0.286759
C	4.377729	0.245813	-0.589318
C	5.733439	-0.108778	-0.629158
H	6.412168	0.429046	-1.292796
C	6.230191	-1.134137	0.176195
H	7.291886	-1.388288	0.132349
C	5.388079	-1.834270	1.042163
H	5.799757	-2.627356	1.668315
C	4.022541	-1.535387	1.113767
C	3.820936	1.356380	-1.473979
H	2.870853	1.696730	-1.032768
C	4.740798	2.585096	-1.531129
H	4.233906	3.411964	-2.052634
H	5.669765	2.375409	-2.083597
H	5.015075	2.929627	-0.523121
C	3.512890	0.832676	-2.889059
H	2.805680	-0.010096	-2.871596
H	4.433231	0.482241	-3.382451
H	3.075366	1.630397	-3.510142
C	3.088882	-2.269905	2.064404
H	2.056108	-2.106736	1.715694
C	3.314650	-3.787688	2.064149
H	3.257492	-4.194214	1.043266
H	2.546592	-4.283980	2.677356
H	4.293587	-4.057999	2.489721
C	3.200438	-1.676783	3.480997
H	2.998054	-0.594646	3.478196
H	4.213567	-1.827182	3.886798
H	2.484330	-2.161650	4.162799
C	-1.389750	1.238139	-1.229290
C	-0.877015	1.316185	-2.525126
H	-0.301474	0.487921	-2.937553
C	-1.151848	2.460870	-3.277602
H	-0.759787	2.528096	-4.295765
C	-2.420600	3.390804	-1.448399
H	-3.035984	4.188746	-1.024645
C	-2.138152	2.275623	-0.658636
H	-2.525816	2.215664	0.357936
I	-3.601771	-2.570858	-0.082134
N	2.211390	-2.559659	-1.288893
C	-1.926992	3.511900	-2.759594
C	-2.199042	4.755786	-3.562517
H	-3.248523	5.073297	-3.464204
H	-1.573402	5.590417	-3.201017
H	-1.979233	4.611826	-4.629793

103
TS-CD-cis+12-N SCF Done: -3249.65706383 A.U.

Au	-1.153097	-0.615550	-1.309018
N	0.763928	-1.697337	-1.835906
N	0.374176	1.539160	0.551769
N	1.631003	1.751171	0.922886
N	2.345146	0.853944	0.282826
C	0.928862	-2.828014	-2.533387
H	0.047049	-3.290119	-2.969791
C	2.221447	-3.319717	-2.791008
H	2.357431	-4.227724	-3.377787
C	3.286747	-2.636634	-2.230431
H	4.318735	-2.968334	-2.377733
C	1.875454	-1.117839	-1.303645
C	1.554983	0.058323	-0.494463
C	0.254307	0.513606	-0.335037
C	-0.658094	2.380185	1.129633
C	-0.874970	3.636492	0.537823
C	-1.838480	4.456610	1.140957
H	-2.046401	5.442114	0.722390
C	-2.532039	4.029575	2.271888
H	-3.273639	4.687292	2.731686
C	-2.298823	2.766273	2.817588
H	-2.869386	2.449248	3.690359
C	-1.356902	1.896803	2.254487
C	-0.071540	4.109159	-0.667973
H	0.298501	3.214069	-1.196587
C	1.153511	4.924345	-0.211074
H	1.757845	5.233945	-1.077772
H	1.795389	4.345751	0.469205
H	0.832064	5.833492	0.321482
C	-0.912728	4.901402	-1.678743
H	-1.216731	5.881094	-1.278398
H	-1.822074	4.352203	-1.962200
H	-0.324076	5.094852	-2.588907
C	-1.106606	0.502928	2.824101
H	-0.955025	-0.178234	1.969727
C	-2.292084	-0.064136	3.616786
H	-3.238315	0.038513	3.065917
H	-2.411583	0.440841	4.588587
H	-2.137227	-1.136591	3.803361
C	0.163573	0.477177	3.696899
H	1.049010	0.857114	3.169640
H	0.375150	-0.549583	4.038696
H	0.027147	1.101167	4.594249
C	3.784764	0.834812	0.437899
C	4.540890	1.660782	-0.414002
C	5.932245	1.611187	-0.258886
H	6.566477	2.228271	-0.897347

C	6.520352	0.782817	0.697568
H	7.607709	0.762873	0.801334
C	5.732438	-0.018373	1.523580
H	6.211111	-0.656921	2.268814
C	4.335976	-0.014676	1.411396
C	3.899882	2.517759	-1.499201
H	2.828385	2.621530	-1.268273
C	4.475173	3.940383	-1.550004
H	3.920897	4.544909	-2.284548
H	5.531761	3.944650	-1.859430
H	4.403054	4.438915	-0.571743
C	4.013651	1.814787	-2.864923
H	3.584617	0.801887	-2.833580
H	5.069363	1.715550	-3.164015
H	3.492965	2.393916	-3.644011
C	3.486892	-0.901364	2.314245
H	2.426787	-0.726593	2.068960
C	3.781241	-2.391045	2.062344
H	3.576164	-2.673194	1.018242
H	3.181386	-3.026222	2.737429
H	4.834725	-2.632689	2.269759
C	3.667398	-0.530068	3.795299
H	3.446508	0.533456	3.969983
H	4.697873	-0.721219	4.132774
H	2.992740	-1.126567	4.429845
C	-2.719951	0.636685	-0.943546
C	-2.975978	1.673801	-1.842578
H	-2.363615	1.815837	-2.735925
C	-4.036804	2.550611	-1.586202
H	-4.236905	3.361503	-2.292246
C	-4.560956	1.353538	0.432526
H	-5.168048	1.223335	1.333002
C	-3.510567	0.463292	0.190097
H	-3.293719	-0.345526	0.886129
N	3.112373	-1.539423	-1.463822
O	-1.802481	-2.254813	0.813810
O	-2.180971	-3.374177	2.706519
C	-2.493100	-3.042925	1.553099
C	-3.724373	-3.659170	0.881370
H	-4.597708	-3.561878	1.542610
H	-3.519525	-4.739076	0.794648
C	-3.958700	-3.032608	-0.489094
C	-2.710643	-2.911626	-1.280855
H	-1.944835	-3.693957	-1.154037
C	-2.541009	-1.984544	-2.334841
H	-1.914059	-2.318622	-3.171334
H	-3.419862	-1.401993	-2.631976
H	-4.625288	-3.671895	-1.105456
H	-4.461547	-2.053612	-0.430161

K	0.348903	-3.555557	1.948554
Cl	0.231631	-4.767015	-0.686753
C	-4.836895	2.416322	-0.441207
C	-5.925548	3.410729	-0.128218
H	-6.289042	3.919044	-1.033473
H	-6.784231	2.931401	0.365901
H	-5.546944	4.188584	0.558175

103

TS-CD-trans+12-N SCF Done: -3249.64995063 A.U.

Au	-1.539072	-0.366906	-0.543691
N	-0.610225	-2.147770	0.271045
N	1.363679	1.272837	-0.509334
N	2.633686	0.908828	-0.349646
N	2.593129	-0.376339	-0.091023
C	-1.238476	-3.231504	0.735848
H	-2.329134	-3.224224	0.700888
C	-0.506261	-4.309925	1.229466
H	-1.009633	-5.192178	1.625736
C	0.885743	-4.218810	1.161334
H	1.524597	-5.046993	1.484772
C	0.753168	-2.112416	0.323515
C	1.307934	-0.829031	-0.077486
C	0.484716	0.246990	-0.373344
C	1.046990	2.663415	-0.743206
C	0.977624	3.109517	-2.077420
C	0.603523	4.446357	-2.274028
H	0.530900	4.846538	-3.285913
C	0.332721	5.279265	-1.186735
H	0.042957	6.318084	-1.362574
C	0.438923	4.805573	0.121900
H	0.232932	5.479971	0.953338
C	0.804389	3.476456	0.383757
C	1.369389	2.202698	-3.240430
H	1.117816	1.165329	-2.957721
C	2.894730	2.257873	-3.462822
H	3.191962	1.569058	-4.268636
H	3.447309	1.982481	-2.553423
H	3.202042	3.275565	-3.751003
C	0.624420	2.512752	-4.545975
H	0.937489	3.477745	-4.973483
H	-0.467800	2.549433	-4.407214
H	0.847078	1.741181	-5.298760
C	0.995241	2.950471	1.801135
H	0.696551	1.889358	1.823343
C	0.147964	3.677796	2.852165
H	-0.924571	3.683204	2.612061
H	0.475769	4.719756	2.996896
H	0.274760	3.175813	3.824822

C	2.485754	3.014660	2.193548
H	3.127536	2.490288	1.472938
H	2.631423	2.545264	3.178014
H	2.828028	4.060661	2.252293
C	3.829276	-1.130173	-0.007768
C	4.323995	-1.668820	-1.212617
C	5.507852	-2.410741	-1.127486
H	5.932078	-2.855121	-2.029495
C	6.156305	-2.587628	0.096689
H	7.081300	-3.167775	0.139237
C	5.637032	-2.026856	1.261861
H	6.159209	-2.170107	2.209852
C	4.449566	-1.279819	1.242248
C	3.594097	-1.504846	-2.542191
H	2.875987	-0.676050	-2.442071
C	4.535973	-1.123616	-3.693722
H	3.954860	-0.919937	-4.607036
H	5.240965	-1.934675	-3.932814
H	5.121558	-0.224190	-3.451125
C	2.789070	-2.774762	-2.874319
H	2.091634	-3.033952	-2.063119
H	3.461965	-3.635598	-3.015152
H	2.211452	-2.639689	-3.803127
C	3.886819	-0.680154	2.520902
H	2.935004	-0.181121	2.291119
C	3.554643	-1.772518	3.549567
H	2.876119	-2.520448	3.116130
H	3.036814	-1.325019	4.410287
H	4.463228	-2.282464	3.909973
C	4.842287	0.374099	3.103424
H	5.057902	1.171307	2.375585
H	5.802409	-0.073827	3.406805
H	4.394282	0.838024	3.996036
C	-3.331923	-1.392464	-0.760286
C	-4.344107	-1.281659	0.198242
H	-4.242012	-0.559969	1.011198
C	-5.480663	-2.093198	0.092811
H	-6.267304	-2.004466	0.848115
C	-4.609882	-3.095030	-1.914514
H	-4.701413	-3.800883	-2.745097
C	-3.465247	-2.292995	-1.823052
H	-2.681000	-2.395692	-2.578884
N	1.505618	-3.130132	0.701627
O	-2.792846	1.109482	1.329692
O	-3.181108	2.715690	2.837417
C	-3.102862	2.301536	1.669039
C	-3.430619	3.277409	0.531132
H	-3.208549	4.301262	0.861324
H	-4.522364	3.230099	0.370189

C	-2.690260	2.951519	-0.759712
C	-2.978485	1.620497	-1.343052
H	-3.978283	1.204149	-1.177314
C	-2.140171	0.961909	-2.239025
H	-2.568211	0.218737	-2.918174
H	-1.224470	1.460525	-2.562868
H	-3.010586	3.656236	-1.556818
H	-1.605701	3.100328	-0.662206
K	-2.025937	0.651202	3.968270
Cl	0.325482	-0.617598	2.908551
C	-5.636097	-3.009958	-0.960259
C	-6.883354	-3.849654	-1.080030
H	-7.290821	-4.112437	-0.092078
H	-7.672213	-3.299244	-1.621276
H	-6.695565	-4.781060	-1.634666

15

PhCF3I SCF Done: -579.692134948 A.U.

C	-1.316805	1.211478	-0.033617
C	0.079723	1.216711	-0.020321
C	0.767899	0.000064	-0.012271
C	0.079620	-1.216550	-0.020306
C	-1.316882	-1.211199	-0.033617
C	-2.015491	0.000178	-0.038301
H	-1.864120	2.155847	-0.047766
H	0.620301	2.164583	-0.019467
H	0.620136	-2.164457	-0.019444
H	-1.864273	-2.155533	-0.047802
I	2.914599	-0.000033	0.007080
C	-3.523485	0.000030	0.004530
F	-4.038887	-1.084934	-0.600212
F	-3.979011	-0.002407	1.272286
F	-4.039129	1.087013	-0.596222

88

A-ad+13-N SCF Done: -2153.60535996 A.U.

Au	0.576130	1.605624	-0.318752
N	-2.230381	2.845247	0.734707
N	-0.911806	-1.017414	-0.410657
N	-2.060128	-1.641164	-0.230226
N	-2.917834	-0.695376	0.061094
C	-2.842217	3.988805	1.044340
H	-2.200618	4.835258	1.311806
C	-4.235201	4.111109	1.035249
H	-4.730873	5.047000	1.297779
C	-4.956263	2.974361	0.663392
H	-6.050407	2.982514	0.614208
C	-3.016080	1.811397	0.405025
C	-2.327585	0.547399	0.081344

C	-0.984431	0.338336	-0.229257
C	0.255923	-1.809219	-0.728479
C	0.517989	-2.081431	-2.084331
C	1.615823	-2.908613	-2.358692
H	1.854070	-3.163269	-3.392619
C	2.399952	-3.425192	-1.324699
H	3.247098	-4.075521	-1.557304
C	2.124394	-3.107156	0.004845
H	2.767373	-3.496499	0.795434
C	1.040537	-2.282071	0.337390
C	-0.385013	-1.553406	-3.191723
H	-0.908088	-0.665365	-2.801167
C	-1.455191	-2.600764	-3.552192
H	-2.147431	-2.203303	-4.311034
H	-2.043176	-2.892362	-2.669560
H	-0.986249	-3.510182	-3.960743
C	0.393666	-1.094913	-4.432309
H	0.870905	-1.938571	-4.954968
H	1.178779	-0.367986	-4.169963
H	-0.289141	-0.615391	-5.150534
C	0.765509	-1.913074	1.788719
H	-0.128858	-1.272706	1.820919
C	1.929319	-1.087950	2.364249
H	2.116522	-0.190172	1.753914
H	2.860835	-1.673430	2.392345
H	1.701618	-0.764643	3.392310
C	0.462342	-3.153983	2.643391
H	-0.389073	-3.719641	2.234795
H	0.213350	-2.857586	3.674506
H	1.328916	-3.832067	2.690788
C	-4.266327	-1.088173	0.418576
C	-5.207038	-1.261575	-0.608961
C	-6.497447	-1.641987	-0.218230
H	-7.266890	-1.787736	-0.979086
C	-6.814580	-1.833704	1.127072
H	-7.828417	-2.128651	1.407985
C	-5.846011	-1.658978	2.115879
H	-6.108700	-1.821642	3.163225
C	-4.537626	-1.283066	1.783802
C	-4.874281	-1.030768	-2.076894
H	-3.801277	-0.794762	-2.154832
C	-5.117658	-2.295095	-2.916762
H	-4.803872	-2.128833	-3.959245
H	-6.184440	-2.568508	-2.932043
H	-4.555006	-3.154542	-2.521726
C	-5.646957	0.178037	-2.632241
H	-5.450154	1.078064	-2.031470
H	-6.733077	-0.006625	-2.621808
H	-5.352376	0.378156	-3.674754

C -3.485348 -1.090566 2.869932
H -2.523814 -0.855589 2.387968
C -3.837545 0.101896 3.775840
H -3.964153 1.026009 3.190467
H -3.043350 0.270570 4.520252
H -4.776910 -0.077590 4.322395
C -3.264241 -2.377997 3.680652
H -2.989490 -3.218259 3.024761
H -4.169041 -2.666003 4.238473
H -2.453787 -2.235105 4.412887
N -4.349682 1.829295 0.344568
I 2.856242 3.128743 -0.373328
C 4.009165 1.329400 -0.007399
C 3.961247 0.317128 -0.962977
C 4.696707 1.199519 1.197845
H 3.414838 0.436710 -1.899207
C 4.604668 -0.888898 -0.678898
C 5.332616 -0.015961 1.465541
H 4.726069 2.007179 1.930719
H 4.567720 -1.701534 -1.402712
C 5.266827 -1.060877 0.538483
H 5.866948 -0.151436 2.408072
C 5.832552 -2.412134 0.908760
F 4.991338 -3.051704 1.759199
F 7.011242 -2.307358 1.529990
F 5.984553 -3.205028 -0.161138

88
TS-AB-cis+13-N SCF Done: -2153.57953518 A.U.
Au -0.769263 -1.569903 -0.072623
N 1.794294 -2.747433 -0.705139
N 0.754486 1.126055 0.415423
N 1.954357 1.678706 0.404901
N 2.765479 0.730952 0.004073
C 2.266073 -3.942538 -1.056116
H 1.543384 -4.763805 -1.108516
C 3.621432 -4.138808 -1.341020
H 4.010617 -5.117426 -1.625925
C 4.448604 -3.017463 -1.241803
H 5.521944 -3.081818 -1.449543
C 2.671650 -1.733069 -0.639539
C 2.092474 -0.441189 -0.237544
C 0.750250 -0.185058 0.031622
C -0.377351 1.918159 0.842813
C -1.021399 2.721149 -0.119064
C -2.121559 3.466889 0.322421
H -2.669709 4.092371 -0.381668
C -2.538617 3.410944 1.653827
H -3.407128 3.993893 1.968342

C	-1.864430	2.614451	2.578165
H	-2.200186	2.592251	3.616908
C	-0.754649	1.846257	2.195204
C	-0.494465	2.832933	-1.545004
H	0.020658	1.887232	-1.786171
C	0.543838	3.969072	-1.636622
H	0.962680	4.026703	-2.653170
H	1.373385	3.822054	-0.930203
H	0.071480	4.937455	-1.407245
C	-1.597954	3.021539	-2.593140
H	-2.089149	4.002266	-2.501383
H	-2.374053	2.249819	-2.505210
H	-1.167038	2.968279	-3.604714
C	-0.001675	1.000318	3.216815
H	0.915281	0.619648	2.741447
C	-0.825778	-0.222535	3.653926
H	-1.047949	-0.880217	2.799128
H	-1.781089	0.083493	4.110130
H	-0.273178	-0.816012	4.398990
C	0.443815	1.838065	4.426788
H	1.034772	2.711483	4.112592
H	1.064839	1.229401	5.102300
H	-0.416407	2.201961	5.009858
C	4.178938	1.034352	-0.105381
C	4.645466	1.569840	-1.315767
C	6.017553	1.850178	-1.386111
H	6.435178	2.262447	-2.306524
C	6.855568	1.608307	-0.298532
H	7.921164	1.837152	-0.375187
C	6.346974	1.079023	0.889791
H	7.020801	0.903520	1.729429
C	4.986989	0.773037	1.015568
C	3.737860	1.794403	-2.518806
H	2.692633	1.689636	-2.187488
C	3.876804	3.210713	-3.097432
H	3.150190	3.361281	-3.911187
H	4.879121	3.381775	-3.519868
H	3.695776	3.977707	-2.329618
C	3.990901	0.719028	-3.590913
H	3.868457	-0.293471	-3.177709
H	5.016743	0.794622	-3.985711
H	3.294144	0.840955	-4.435477
C	4.399556	0.233212	2.314170
H	3.484876	-0.329414	2.062145
C	5.330847	-0.749274	3.036394
H	5.663081	-1.555709	2.364530
H	4.808727	-1.205296	3.891786
H	6.225972	-0.247828	3.436100
C	3.983264	1.394855	3.236830

H	3.271483	2.070338	2.738880
H	4.862946	1.990243	3.528570
H	3.510887	1.011800	4.155135
N	3.976638	-1.817057	-0.893731
I	-2.616392	-3.512078	-0.047939
C	-2.968098	-1.010371	-0.227842
C	-3.335166	-0.607193	-1.514331
C	-3.459920	-0.407249	0.928011
H	-2.979688	-1.134178	-2.399880
C	-4.166393	0.507610	-1.633764
C	-4.284304	0.710216	0.781029
H	-3.193450	-0.770777	1.919149
H	-4.454616	0.856829	-2.627642
H	-4.649342	1.227308	1.668125
C	-4.621127	1.176938	-0.491402
C	-5.464432	2.418619	-0.662613
F	-4.845347	3.302819	-1.475966
F	-5.686449	3.039092	0.507320
F	-6.650842	2.140728	-1.216688

88

TS-AB-trans+13-N SCF Done: -2153.57234899 A.U.

Au	1.241865	0.509591	0.037358
N	0.654737	-2.141145	0.047501
N	-1.868525	1.100973	-0.013272
N	-3.045375	0.498767	-0.053929
N	-2.771330	-0.784745	-0.039072
C	1.397393	-3.247337	0.061627
H	2.484845	-3.120547	0.081358
C	0.805157	-4.514570	0.050951
H	1.406992	-5.424404	0.062295
C	-0.591371	-4.553252	0.025098
H	-1.132296	-5.505288	0.015157
C	-0.685013	-2.281754	0.023185
C	-1.418522	-1.008451	0.007458
C	-0.813095	0.241750	0.024866
C	-1.782448	2.543531	-0.002101
C	-1.891655	3.195138	1.238843
C	-1.781147	4.592432	1.224143
H	-1.866406	5.151008	2.157292
C	-1.559072	5.279797	0.030662
H	-1.476810	6.369248	0.042308
C	-1.433132	4.591911	-1.177789
H	-1.250054	5.152139	-2.095327
C	-1.538337	3.195922	-1.225092
C	-2.155232	2.424807	2.526912
H	-1.804222	1.390020	2.376346
C	-3.666996	2.362512	2.815060
H	-3.864833	1.771409	3.722845

H	-4.215800	1.903814	1.978982
H	-4.072672	3.374707	2.971759
C	-1.377179	2.980779	3.727680
H	-1.741724	3.975227	4.028769
H	-0.301466	3.065734	3.507259
H	-1.500481	2.316412	4.597070
C	-1.443771	2.421473	-2.534649
H	-1.039465	1.422723	-2.301459
C	-0.480325	3.054134	-3.546688
H	0.509760	3.235478	-3.099655
H	-0.860949	4.011614	-3.935136
H	-0.351761	2.385488	-4.411998
C	-2.846443	2.221999	-3.140733
H	-3.526054	1.722230	-2.434159
H	-2.791588	1.610400	-4.055269
H	-3.294314	3.192861	-3.405968
C	-3.862471	-1.735403	-0.082690
C	-4.413300	-2.159865	1.138468
C	-5.454918	-3.093877	1.063631
H	-5.915580	-3.459469	1.983427
C	-5.911894	-3.562952	-0.168524
H	-6.725864	-4.290985	-0.202396
C	-5.343221	-3.105870	-1.357718
H	-5.717837	-3.479228	-2.312919
C	-4.297754	-2.173218	-1.344839
C	-3.897205	-1.671473	2.486419
H	-3.184133	-0.851326	2.307451
C	-5.021913	-1.094910	3.360461
H	-4.606200	-0.685752	4.294649
H	-5.756256	-1.866777	3.638715
H	-5.558391	-0.286756	2.841155
C	-3.133593	-2.792889	3.213177
H	-2.317611	-3.188827	2.589675
H	-3.804624	-3.632705	3.454530
H	-2.705637	-2.421816	4.158036
C	-3.666976	-1.691895	-2.645667
H	-2.926954	-0.912154	-2.406165
C	-2.912327	-2.836448	-3.344349
H	-2.161041	-3.283933	-2.675723
H	-2.402465	-2.468721	-4.249004
H	-3.603627	-3.637165	-3.651791
C	-4.704388	-1.043681	-3.576269
H	-5.227639	-0.214712	-3.075891
H	-5.461765	-1.771545	-3.906942
H	-4.213040	-0.644620	-4.477471
N	-1.335715	-3.442405	0.011340
I	2.663135	2.850182	-0.028458
C	3.331800	0.297871	0.043837
C	3.838461	-0.193982	-1.169325

C	3.833677	-0.129743	1.282841
H	3.496942	0.209014	-2.124324
C	4.757098	-1.244052	-1.131292
C	4.752192	-1.180983	1.303215
H	3.489194	0.322730	2.214383
H	5.136047	-1.673835	-2.060663
H	5.126992	-1.562022	2.255211
C	5.188922	-1.750646	0.100967
C	6.041704	-2.997359	0.133543
F	5.236788	-4.087757	0.146290
F	6.807934	-3.056116	1.227711
F	6.828886	-3.097662	-0.942806

88

B-cis+13-N SCF Done: -2153.61221036 A.U.

Au	-0.646324	1.616033	-0.102389
N	1.434888	2.579494	-0.073592
N	0.601498	-1.383626	0.026008
N	1.819373	-1.905055	0.095717
N	2.643682	-0.887165	0.104802
C	1.753550	3.877505	-0.107713
H	0.920692	4.582893	-0.184742
C	3.089009	4.281587	-0.045623
H	3.358184	5.338192	-0.074621
C	4.053794	3.277123	0.056508
H	5.120377	3.517220	0.113029
C	2.443738	1.675496	0.023327
C	1.962958	0.295347	0.045100
C	0.612319	-0.021800	-0.005531
C	-0.533313	-2.284096	-0.050837
C	-1.262411	-2.536323	1.128909
C	-2.363877	-3.390763	1.005667
H	-2.990057	-3.599969	1.871998
C	-2.687787	-3.976134	-0.219916
H	-3.559669	-4.629676	-0.285734
C	-1.926097	-3.717042	-1.357098
H	-2.198217	-4.186374	-2.303452
C	-0.828522	-2.846563	-1.305033
C	-0.830110	-1.956222	2.472141
H	-0.490851	-0.921103	2.290629
C	0.359983	-2.754523	3.044770
H	0.721456	-2.292646	3.976901
H	1.204058	-2.816442	2.343942
H	0.049248	-3.785601	3.275556
C	-1.957432	-1.883650	3.508730
H	-2.260681	-2.886627	3.847223
H	-2.845758	-1.374600	3.111452
H	-1.613319	-1.333551	4.397806
C	0.014652	-2.561741	-2.542817

H	0.535237	-1.601290	-2.385206
C	-0.821713	-2.403844	-3.821343
H	-1.628852	-1.665673	-3.697269
H	-1.281105	-3.355375	-4.129940
H	-0.180802	-2.071586	-4.652415
C	1.088361	-3.652025	-2.721500
H	1.738883	-3.727346	-1.837971
H	1.717858	-3.435992	-3.598243
H	0.614730	-4.634296	-2.876479
C	4.072330	-1.116690	0.187105
C	4.655217	-1.136502	1.465799
C	6.041428	-1.334427	1.516345
H	6.544644	-1.360047	2.484909
C	6.785836	-1.499493	0.348374
H	7.865966	-1.650791	0.411908
C	6.163810	-1.479429	-0.900821
H	6.764246	-1.614139	-1.802138
C	4.780596	-1.289699	-1.015713
C	3.850857	-0.939908	2.744796
H	2.785048	-0.859623	2.478951
C	3.979669	-2.148468	3.686088
H	3.339961	-2.012515	4.571966
H	5.014528	-2.274925	4.040447
H	3.677478	-3.080567	3.184928
C	4.244076	0.372075	3.445947
H	4.136590	1.233457	2.768909
H	5.291900	0.343280	3.784189
H	3.611708	0.541318	4.331720
C	4.107390	-1.228264	-2.381735
H	3.018922	-1.308074	-2.233499
C	4.384456	0.126605	-3.058752
H	4.068613	0.967417	-2.422731
H	3.852409	0.196410	-4.020677
H	5.460987	0.250267	-3.257371
C	4.513939	-2.399696	-3.288448
H	4.321446	-3.368583	-2.804199
H	5.581048	-2.357610	-3.555800
H	3.942522	-2.365996	-4.229000
C	-2.411260	0.591783	-0.054082
C	-3.175183	0.604297	1.113814
H	-2.882247	1.202512	1.977568
C	-4.343288	-0.161296	1.164403
H	-4.953707	-0.163647	2.069558
C	-3.976676	-0.893850	-1.116280
H	-4.291201	-1.478311	-1.981986
C	-2.809261	-0.129070	-1.178551
H	-2.219965	-0.118143	-2.095843
I	-2.012151	3.897727	-0.272144
N	3.730003	1.978646	0.091187

C	-4.732002	-0.922517	0.058619
C	-5.921577	-1.844316	0.169935
F	-5.551071	-3.033861	0.704656
F	-6.468150	-2.106968	-1.025152
F	-6.872948	-1.339715	0.966244

88

TS-decomp-cis+13-N SCF Done: -2153.57392158 A.U.

Au	-0.929925	-1.738259	-0.352440
N	0.900919	-0.761179	-2.753646
N	0.383088	0.599056	1.289339
N	1.566098	1.170312	1.371453
N	2.166951	0.917266	0.237276
C	1.301479	-1.325886	-3.895152
H	0.518904	-1.627263	-4.598845
C	2.655675	-1.523907	-4.178674
H	2.983298	-1.983151	-5.112570
C	3.566908	-1.111477	-3.202466
H	4.646159	-1.234986	-3.340625
C	1.854908	-0.406064	-1.886385
C	1.391392	0.161191	-0.603125
C	0.189538	-0.047684	0.088532
C	-0.539964	0.750768	2.393950
C	-1.195909	1.995363	2.529560
C	-2.124310	2.098104	3.572196
H	-2.664324	3.033487	3.721658
C	-2.375663	1.019247	4.422557
H	-3.110056	1.123445	5.224732
C	-1.688073	-0.183578	4.268716
H	-1.878827	-1.004361	4.961635
C	-0.737971	-0.349105	3.249263
C	-0.893927	3.183633	1.621542
H	-0.573148	2.782690	0.644200
C	0.262411	4.023630	2.201955
H	0.490603	4.867523	1.533296
H	1.177536	3.432354	2.339676
H	-0.025128	4.436695	3.181652
C	-2.108690	4.084850	1.357833
H	-2.392627	4.656230	2.255062
H	-2.985923	3.520364	1.019213
H	-1.865189	4.819234	0.575182
C	0.090216	-1.626017	3.168988
H	0.592165	-1.659726	2.189677
C	-0.761403	-2.898434	3.280668
H	-1.580580	-2.905863	2.546356
H	-1.197931	-3.010343	4.285014
H	-0.140033	-3.786337	3.091243
C	1.198590	-1.592229	4.238649
H	1.839845	-0.704906	4.128278

H	1.833829	-2.488515	4.159858
H	0.764851	-1.571380	5.250947
C	3.474279	1.503470	0.001926
C	3.516376	2.751341	-0.644549
C	4.790958	3.291533	-0.865806
H	4.888174	4.256101	-1.366240
C	5.938051	2.617715	-0.445358
H	6.920214	3.061982	-0.623272
C	5.845480	1.386174	0.205933
H	6.755714	0.880876	0.532429
C	4.601257	0.789811	0.440922
C	2.256296	3.473069	-1.110796
H	1.405669	3.076291	-0.534225
C	2.303021	4.983646	-0.836458
H	1.321346	5.435960	-1.046686
H	3.038003	5.493210	-1.478499
H	2.562824	5.194571	0.211370
C	1.978785	3.192309	-2.599397
H	1.880477	2.115661	-2.804703
H	2.799871	3.574410	-3.226272
H	1.047771	3.686474	-2.919601
C	4.472566	-0.540933	1.167399
H	3.477608	-0.955893	0.937037
C	5.498050	-1.579041	0.692775
H	5.454194	-1.705898	-0.399352
H	5.294005	-2.553686	1.162471
H	6.526338	-1.296418	0.966880
C	4.544668	-0.323202	2.690097
H	3.780177	0.393096	3.028425
H	5.529560	0.076808	2.979218
H	4.391490	-1.272917	3.226125
C	-1.774659	0.198757	-0.599316
C	-1.719661	0.844529	-1.836990
H	-0.899948	0.637165	-2.524430
C	-2.746748	1.730182	-2.171015
H	-2.728830	2.246130	-3.132384
C	-3.844904	1.280912	-0.050969
H	-4.682220	1.439931	0.631054
C	-2.813350	0.416694	0.316824
H	-2.847414	-0.088704	1.281066
I	-1.162332	-4.314563	-0.580838
N	3.167478	-0.560196	-2.054016
C	-3.802794	1.947526	-1.280466
C	-4.880200	2.961902	-1.599592
F	-6.086356	2.529219	-1.210847
F	-4.932158	3.240014	-2.907504
F	-4.637994	4.116806	-0.946579

103

TS-CD-cis+13-N SCF Done: -3547.15585547 A.U.

Au	-0.545177	-1.042553	-1.315495
N	1.606886	-1.426832	-1.848728
N	0.189059	1.485527	0.560207
N	1.306766	2.097288	0.930607
N	2.275036	1.490340	0.283633
C	2.127386	-2.438019	-2.556066
H	1.442359	-3.158047	-2.995871
C	3.508264	-2.478611	-2.822217
H	3.929210	-3.287923	-3.418153
C	4.295967	-1.490201	-2.258455
H	5.378910	-1.466553	-2.411178
C	2.472193	-0.518907	-1.315642
C	1.788799	0.483887	-0.498996
C	0.412245	0.484984	-0.335471
C	-1.064012	1.920776	1.150564
C	-1.739100	2.988634	0.531441
C	-2.938885	3.400234	1.126357
H	-3.519235	4.206072	0.676968
C	-3.410736	2.784075	2.284536
H	-4.351781	3.118855	2.725447
C	-2.710833	1.726637	2.865586
H	-3.111744	1.249622	3.759921
C	-1.520138	1.249959	2.302520
C	-1.167563	3.696318	-0.691744
H	-0.546559	2.967026	-1.240625
C	-0.254302	4.859861	-0.257313
H	0.198496	5.345122	-1.135841
H	0.554980	4.520032	0.405030
H	-0.837206	5.618848	0.288035
C	-2.243316	4.188212	-1.669240
H	-2.826190	5.022125	-1.248510
H	-2.944252	3.384714	-1.936035
H	-1.771652	4.558945	-2.592338
C	-0.772694	0.057413	2.893173
H	-0.373387	-0.528642	2.047584
C	-1.671271	-0.900054	3.688075
H	-2.582263	-1.164533	3.131096
H	-1.980984	-0.463912	4.650908
H	-1.134930	-1.837776	3.891236
C	0.408531	0.516746	3.769745
H	1.091876	1.192164	3.237258
H	0.985250	-0.352602	4.126222
H	0.041690	1.056595	4.656953
C	3.639932	1.952367	0.427071
C	4.072088	2.973678	-0.438850
C	5.402448	3.389639	-0.299085
H	5.790571	4.175804	-0.948812
C	6.239896	2.812546	0.656081

H	7.273348	3.154754	0.747479
C	5.768831	1.804353	1.496864
H	6.438544	1.368145	2.240766
C	4.449043	1.344241	1.401104
C	3.174507	3.562559	-1.520890
H	2.132757	3.287809	-1.293590
C	3.222230	5.096930	-1.558807
H	2.497438	5.478696	-2.294444
H	4.214183	5.468785	-1.858542
H	2.976138	5.531403	-0.578273
C	3.525240	2.952618	-2.890897
H	3.472214	1.853569	-2.867507
H	4.550189	3.226430	-3.188002
H	2.837013	3.322017	-3.667641
C	3.950308	0.233717	2.317809
H	2.884262	0.059218	2.098721
C	4.702167	-1.082213	2.048041
H	4.576445	-1.414251	1.005881
H	4.354301	-1.876518	2.731570
H	5.781840	-0.971130	2.230502
C	4.038739	0.642799	3.797175
H	3.493036	1.579172	3.985855
H	5.083847	0.791436	4.109948
H	3.606677	-0.138828	4.441994
C	-2.434919	-0.375726	-0.952367
C	-3.044552	0.456141	-1.896989
H	-2.541041	0.721247	-2.828968
C	-4.317815	0.968988	-1.633561
H	-4.806397	1.621601	-2.359670
C	-4.355191	-0.196636	0.490978
H	-4.864543	-0.439868	1.425003
C	-3.086969	-0.721988	0.229878
H	-2.594792	-1.372821	0.950930
N	3.777511	-0.514620	-1.482504
O	-0.604555	-2.764858	0.827714
O	-0.568092	-3.961635	2.710510
C	-0.996376	-3.735462	1.569452
C	-1.991606	-4.701860	0.918904
H	-2.846197	-4.863827	1.592009
H	-1.473269	-5.671515	0.838922
C	-2.422245	-4.193373	-0.452924
C	-1.281286	-3.706954	-1.264421
H	-0.306529	-4.205525	-1.134577
C	-1.415090	-2.791345	-2.334171
H	-0.714205	-2.921368	-3.168263
H	-2.433894	-2.525519	-2.637007
H	-2.868057	-5.013801	-1.054189
H	-3.201254	-3.415645	-0.395096
K	1.874935	-3.338578	1.927640

Cl	2.092924	-4.502583	-0.726801
C	-4.962195	0.658974	-0.431885
C	-6.270038	1.329786	-0.090788
F	-7.008640	0.590856	0.749572
F	-7.004264	1.579635	-1.184521
F	-6.049473	2.518918	0.516689

103
TS-CD-trans+13-N SCF Done: -3547.14563806 A.U.

Au	1.214392	0.092271	0.414923
N	0.702327	-1.810373	-0.487031
N	-1.989429	0.964319	0.633450
N	-3.140440	0.310618	0.498553
N	-2.806965	-0.906289	0.141752
C	1.544252	-2.673570	-1.063344
H	2.600554	-2.401442	-1.080113
C	1.067415	-3.865385	-1.605809
H	1.744962	-4.568368	-2.091204
C	-0.298177	-4.125217	-1.469253
H	-0.735641	-5.062903	-1.826918
C	-0.629854	-2.108993	-0.477611
C	-1.453744	-1.029436	0.042056
C	-0.899059	0.194604	0.383441
C	-2.008154	2.371954	0.961849
C	-1.980445	2.732589	2.323245
C	-1.933403	4.104568	2.608245
H	-1.907736	4.443110	3.644606
C	-1.931584	5.047989	1.579072
H	-1.894745	6.112320	1.823684
C	-1.989938	4.649755	0.242552
H	-2.000745	5.407338	-0.541313
C	-2.034300	3.291760	-0.107464
C	-2.080597	1.683636	3.427012
H	-1.603480	0.758817	3.057372
C	-3.560669	1.351697	3.707340
H	-3.640473	0.560730	4.468971
H	-4.078450	1.009477	2.800143
H	-4.087525	2.243219	4.082456
C	-1.363395	2.079750	4.724857
H	-1.876053	2.909251	5.235739
H	-0.321086	2.390845	4.550465
H	-1.353615	1.229778	5.424114
C	-2.172778	2.829388	-1.552790
H	-1.626280	1.878905	-1.670089
C	-1.591489	3.811826	-2.576753
H	-0.540416	4.066116	-2.380386
H	-2.171088	4.747609	-2.625334
H	-1.649509	3.360206	-3.580162
C	-3.654613	2.549331	-1.877957

H	-4.106131	1.835650	-1.175840
H	-3.739702	2.125831	-2.889911
H	-4.243924	3.479834	-1.839353
C	-3.828792	-1.931334	0.045850
C	-4.112697	-2.653736	1.222284
C	-5.087141	-3.653607	1.123078
H	-5.342157	-4.247470	2.002483
C	-5.740815	-3.899838	-0.086247
H	-6.500766	-4.683128	-0.139869
C	-5.436126	-3.152535	-1.222243
H	-5.960267	-3.354346	-2.158405
C	-4.463000	-2.142383	-1.188320
C	-3.371506	-2.407252	2.532615
H	-2.876032	-1.425736	2.469929
C	-4.313240	-2.339144	3.743859
H	-3.748237	-2.063048	4.648209
H	-4.793011	-3.309229	3.945597
H	-5.106800	-1.592054	3.592745
C	-2.272543	-3.466450	2.736060
H	-1.580293	-3.498630	1.880736
H	-2.714519	-4.470456	2.837051
H	-1.692290	-3.257686	3.649439
C	-4.131356	-1.340528	-2.436759
H	-3.320147	-0.636756	-2.203242
C	-3.596295	-2.248643	-3.555291
H	-2.733468	-2.831221	-3.203430
H	-3.250419	-1.633217	-4.398409
H	-4.370049	-2.944117	-3.919914
C	-5.344435	-0.520494	-2.905514
H	-5.710735	0.149981	-2.113015
H	-6.180138	-1.171599	-3.208877
H	-5.070203	0.096207	-3.775643
C	3.215156	-0.462781	0.481359
C	4.112969	-0.032081	-0.501888
H	3.783664	0.689444	-1.252098
C	5.423279	-0.523465	-0.500061
H	6.129411	-0.200099	-1.267873
C	4.936733	-1.853165	1.468474
H	5.259861	-2.566828	2.228954
C	3.624631	-1.367052	1.469781
H	2.930002	-1.715824	2.239074
N	-1.133833	-3.253986	-0.900579
O	1.960814	1.928527	-1.371706
O	1.863158	3.682295	-2.755278
C	1.953963	3.182260	-1.622618
C	2.104220	4.128408	-0.424340
H	1.631960	5.090045	-0.666606
H	3.183763	4.330822	-0.307130
C	1.529917	3.555004	0.865412

C	2.158830	2.302965	1.345590
H	3.223091	2.161218	1.126592
C	1.546291	1.399320	2.209286
H	2.169052	0.735937	2.816542
H	0.550387	1.630643	2.591374
H	1.712923	4.268596	1.697286
H	0.438064	3.441680	0.816376
K	1.174573	1.502227	-4.034256
Cl	-0.710590	-0.378120	-2.946824
C	5.833762	-1.431366	0.482190
C	7.267116	-1.912270	0.510226
F	7.785690	-1.980641	-0.726533
F	8.041881	-1.078154	1.224570
F	7.366967	-3.129675	1.068319

14
PhNO2I SCF Done: -447.249537102 A.U.

C	1.666034	-1.219578	-0.000073
C	0.270742	-1.219079	-0.000062
C	-0.417267	0.000051	-0.000019
C	0.270669	1.219207	0.000072
C	1.665973	1.219815	0.000116
H	2.234622	-2.149291	-0.000160
H	-0.271098	-2.166107	-0.000165
H	-0.271223	2.166207	0.000177
H	2.234467	2.149569	0.000160
I	-2.559384	-0.000024	-0.000002
C	2.343027	0.000134	0.000020
O	4.385547	1.082584	-0.000224
O	4.385120	-1.082862	0.000214
N	3.822885	-0.000024	-0.000023

87
A-ad+14-N SCF Done: -2021.15952914 A.U.

Au	0.817615	1.539700	-0.086466
N	-2.009874	2.886253	0.778249
N	-0.768143	-1.024324	-0.326582
N	-1.959405	-1.588354	-0.275225
N	-2.790974	-0.606577	-0.031685
C	-2.598816	4.057585	1.022249
H	-1.954050	4.871173	1.371556
C	-3.973156	4.247868	0.847546
H	-4.450479	5.206705	1.055788
C	-4.699570	3.148065	0.385434
H	-5.778779	3.209653	0.208812
C	-2.800594	1.893621	0.349230
C	-2.142179	0.600750	0.084096
C	-0.788501	0.328156	-0.108731
C	0.375791	-1.873052	-0.577071

C	0.787983	-2.054234	-1.909804
C	1.862188	-2.929499	-2.123314
H	2.215035	-3.107452	-3.141264
C	2.478778	-3.586619	-1.057640
H	3.312252	-4.267176	-1.243708
C	2.052504	-3.364416	0.252518
H	2.557876	-3.873893	1.075597
C	0.984695	-2.497968	0.526230
C	0.132077	-1.325650	-3.075805
H	-0.762054	-0.805505	-2.699509
C	-0.342272	-2.292324	-4.172188
H	-0.881454	-1.740803	-4.958232
H	-1.020676	-3.056570	-3.764291
H	0.502316	-2.809872	-4.653358
C	1.079937	-0.250596	-3.637423
H	1.998872	-0.706098	-4.041004
H	1.369800	0.469352	-2.854799
H	0.595162	0.308426	-4.453235
C	0.539067	-2.246089	1.960538
H	-0.343084	-1.588784	1.939913
C	1.636520	-1.500382	2.737929
H	1.888644	-0.548396	2.243826
H	2.557270	-2.101968	2.801679
H	1.303502	-1.280628	3.764594
C	0.115624	-3.544308	2.665270
H	-0.679190	-4.059379	2.104445
H	-0.265082	-3.324251	3.674861
H	0.961706	-4.240876	2.773038
C	-4.189356	-0.938699	0.160010
C	-5.024288	-0.946853	-0.970561
C	-6.359538	-1.305430	-0.757667
H	-7.052606	-1.334288	-1.599582
C	-6.821521	-1.623575	0.522528
H	-7.869056	-1.899392	0.665312
C	-5.960694	-1.591336	1.618180
H	-6.340608	-1.839540	2.611353
C	-4.609698	-1.246821	1.462283
C	-4.483408	-0.610565	-2.353652
H	-3.607398	0.044513	-2.213698
C	-4.001382	-1.886068	-3.070677
H	-3.556083	-1.637995	-4.047354
H	-4.844309	-2.573625	-3.245279
H	-3.248641	-2.423785	-2.474696
C	-5.481589	0.174237	-3.214332
H	-5.848952	1.066102	-2.684082
H	-6.350754	-0.439361	-3.498626
H	-4.999253	0.501802	-4.148382
C	-3.674904	-1.193455	2.664370
H	-2.661430	-0.953192	2.307313

C	-4.087436	-0.069497	3.630442
H	-4.122535	0.904912	3.117888
H	-3.373180	0.004937	4.465729
H	-5.084989	-0.257561	4.058034
C	-3.580612	-2.553697	3.373831
H	-3.257359	-3.342941	2.678026
H	-4.549660	-2.855193	3.801427
H	-2.853597	-2.505630	4.199960
N	-4.115395	1.975418	0.131992
I	3.168889	2.972600	-0.080678
C	4.131335	1.046565	0.158393
C	4.267950	0.233915	-0.967496
C	4.465003	0.625190	1.444884
H	3.996747	0.585723	-1.963525
C	4.719344	-1.073705	-0.788544
C	4.895166	-0.691675	1.618631
H	4.358086	1.283331	2.308214
H	4.822047	-1.756750	-1.630196
C	4.989694	-1.521212	0.503638
H	5.125243	-1.085022	2.608619
O	5.212340	-3.386025	1.840509
O	5.613942	-3.601529	-0.278973
N	5.311343	-2.950088	0.705152

87

TS-AB-cis+14-N SCF Done: -2021.13457303 A.U.

Au	-0.987952	1.507031	-0.017577
N	1.465151	2.846247	-0.102026
N	0.613263	-1.213180	0.071247
N	1.845741	-1.694809	0.073735
N	2.627168	-0.645526	0.010028
C	1.863086	4.115849	-0.160650
H	1.079313	4.880843	-0.163227
C	3.219434	4.452741	-0.215815
H	3.549614	5.491454	-0.263279
C	4.126976	3.390729	-0.207254
H	5.207223	3.565285	-0.248417
C	2.416089	1.896890	-0.098684
C	1.903552	0.519221	-0.032173
C	0.561581	0.149897	0.007358
C	-0.485296	-2.155756	0.131733
C	-0.968364	-2.524051	1.401811
C	-1.971753	-3.501644	1.431862
H	-2.383473	-3.828706	2.387308
C	-2.458251	-4.067060	0.252668
H	-3.247200	-4.820431	0.301155
C	-1.971962	-3.655695	-0.988311
H	-2.385625	-4.098252	-1.895160
C	-0.963539	-2.685902	-1.080836

C	-0.376663	-1.943555	2.680625
H	0.037769	-0.950751	2.438173
C	0.784943	-2.826494	3.176336
H	1.256640	-2.383729	4.067376
H	1.557317	-2.950711	2.403335
H	0.417665	-3.829087	3.447089
C	-1.416639	-1.729572	3.788667
H	-1.805385	-2.683133	4.178014
H	-2.271698	-1.135930	3.431742
H	-0.960266	-1.196979	4.637140
C	-0.356848	-2.288946	-2.421390
H	0.107542	-1.295899	-2.301372
C	-1.394831	-2.157401	-3.543885
H	-2.201659	-1.463196	-3.266439
H	-1.852391	-3.125794	-3.798299
H	-0.914735	-1.776429	-4.458403
C	0.757287	-3.280306	-2.808984
H	1.531949	-3.340260	-2.030386
H	1.237955	-2.974729	-3.751201
H	0.342093	-4.290858	-2.949641
C	4.063019	-0.844462	-0.002852
C	4.733659	-0.858015	1.231962
C	6.124391	-1.021597	1.186714
H	6.693867	-1.035494	2.118049
C	6.791437	-1.166852	-0.029841
H	7.876787	-1.291690	-0.040902
C	6.083638	-1.162456	-1.232089
H	6.621976	-1.284728	-2.173854
C	4.691874	-1.002747	-1.249705
C	4.015375	-0.676066	2.563601
H	2.929811	-0.691896	2.377880
C	4.313894	-1.827414	3.537006
H	3.726439	-1.708018	4.460708
H	5.376545	-1.849546	3.824458
H	4.062558	-2.802982	3.094307
C	4.354104	0.690140	3.186015
H	4.118061	1.513771	2.495625
H	5.426586	0.756925	3.428885
H	3.788288	0.841862	4.118919
C	3.928699	-0.968966	-2.568398
H	2.850197	-0.990642	-2.345651
C	4.214894	0.337832	-3.329683
H	3.980090	1.219158	-2.714244
H	3.618246	0.386538	-4.254430
H	5.278065	0.403022	-3.611094
C	4.224593	-2.201702	-3.436898
H	4.012399	-3.135378	-2.894741
H	5.276972	-2.228014	-3.759528
H	3.604183	-2.184298	-4.346494

N 3.728176 2.116616 -0.149176
I -2.964396 3.332040 -0.024557
C -3.170778 0.855372 -0.049976
C -3.560610 0.289233 1.168472
C -3.538351 0.319760 -1.289485
H -3.300900 0.757742 2.117758
C -4.258876 -0.916155 1.139909
C -4.238214 -0.885163 -1.302408
H -3.263175 0.811349 -2.222726
H -4.547836 -1.423078 2.060170
H -4.513959 -1.368429 -2.239422
C -4.569148 -1.493798 -0.091637
O -5.490532 -3.327259 0.959724
O -5.390024 -3.338687 -1.204274
N -5.214724 -2.819372 -0.114574

87
TS-AB-trans+14-N SCF Done: -2021.12546035 A.U.
Au 1.373495 0.122674 0.025453
N 0.307824 -2.464016 0.068339
N -1.578171 1.197217 -0.062515
N -2.843985 0.817689 -0.052854
N -2.805270 -0.493499 -0.009137
C 0.816876 -3.694127 0.091960
H 1.908509 -3.777516 0.117599
C -0.008080 -4.823742 0.085020
H 0.404710 -5.833441 0.103803
C -1.385486 -4.590806 0.052846
H -2.101185 -5.419650 0.045902
C -1.033321 -2.344763 0.039461
C -1.513776 -0.957236 0.010566
C -0.693086 0.162082 -0.023165
C -1.232656 2.598812 -0.124220
C -1.009828 3.272718 1.091518
C -0.630629 4.617782 1.000615
H -0.452081 5.192106 1.910432
C -0.473911 5.236374 -0.241919
H -0.176185 6.286637 -0.288198
C -0.689038 4.527291 -1.423242
H -0.550352 5.026425 -2.384075
C -1.077049 3.180121 -1.393735
C -1.214294 2.578405 2.432704
H -1.013586 1.504210 2.284178
C -2.679322 2.715546 2.890123
H -2.844832 2.168485 3.831622
H -3.376810 2.320436 2.136347
H -2.933140 3.773772 3.061191
C -0.241110 3.058241 3.517294
H -0.444847 4.096291 3.823049

H	0.803843	3.002042	3.173840
H	-0.342940	2.432829	4.417682
C	-1.259274	2.387532	-2.682502
H	-1.731243	1.424997	-2.432860
C	0.108169	2.076002	-3.317372
H	0.759592	1.531283	-2.615332
H	0.630646	3.002702	-3.603335
H	-0.015009	1.461818	-4.223569
C	-2.196807	3.091394	-3.675122
H	-3.174117	3.307376	-3.217459
H	-2.365305	2.452779	-4.556268
H	-1.772513	4.041304	-4.035760
C	-4.052712	-1.229042	0.017472
C	-4.628680	-1.498862	1.268378
C	-5.834814	-2.213990	1.259048
H	-6.324043	-2.454734	2.204887
C	-6.416685	-2.621556	0.059470
H	-7.358519	-3.175115	0.075737
C	-5.810077	-2.327740	-1.164137
H	-6.286696	-2.652927	-2.089855
C	-4.602239	-1.622375	-1.215947
C	-3.976698	-1.081604	2.580689
H	-3.121215	-0.427187	2.350442
C	-4.930705	-0.263315	3.464545
H	-4.408692	0.082126	4.370625
H	-5.795545	-0.861464	3.791278
H	-5.311624	0.620439	2.930671
C	-3.425279	-2.310558	3.325329
H	-2.727302	-2.880117	2.692552
H	-4.239956	-2.992056	3.617820
H	-2.897328	-2.004265	4.242421
C	-3.940049	-1.250963	-2.537431
H	-2.861198	-1.120213	-2.347481
C	-4.066679	-2.345814	-3.604871
H	-3.717611	-3.318723	-3.225761
H	-3.465145	-2.083018	-4.488826
H	-5.106142	-2.466239	-3.947665
C	-4.482352	0.097991	-3.048052
H	-4.344937	0.896330	-2.303351
H	-5.559685	0.023403	-3.265387
H	-3.967536	0.398052	-3.974494
N	-1.898149	-3.356587	0.030652
I	3.074017	2.274161	0.229266
C	3.448920	-0.277317	0.045904
C	3.950877	-0.674805	-1.207390
C	3.900033	-0.867217	1.241440
H	3.644764	-0.160778	-2.120204
C	4.839037	-1.748505	-1.270193
C	4.786569	-1.941188	1.172048

H	3.554033	-0.501164	2.209631
H	5.235603	-2.107712	-2.220190
H	5.142794	-2.447448	2.069602
C	5.234162	-2.365914	-0.081542
O	6.474840	-4.049934	0.900301
O	6.563289	-3.846421	-1.257066
N	6.173287	-3.515962	-0.151993

87
B-cis+14-N SCF Done: -2021.16666529 A.U.

Au	-0.888753	1.552519	-0.103680
N	1.129410	2.619711	-0.086966
N	0.512722	-1.383721	0.057424
N	1.758667	-1.836268	0.118097
N	2.526914	-0.776020	0.098570
C	1.377113	3.933201	-0.129033
H	0.506728	4.592879	-0.194341
C	2.689628	4.408541	-0.089656
H	2.900856	5.478063	-0.124664
C	3.708720	3.457654	-0.002564
H	4.761675	3.754764	0.035219
C	2.187189	1.771058	-0.005359
C	1.782420	0.366862	0.031133
C	0.450602	-0.022984	0.002834
C	-0.567727	-2.353829	0.028988
C	-1.250320	-2.622587	1.232332
C	-2.288983	-3.560106	1.161682
H	-2.864986	-3.796623	2.056364
C	-2.601808	-4.201359	-0.038573
H	-3.425570	-4.917337	-0.071008
C	-1.887717	-3.918505	-1.201587
H	-2.153321	-4.428836	-2.128121
C	-0.851495	-2.975011	-1.200765
C	-0.827501	-1.978326	2.549227
H	-0.546519	-0.933090	2.330456
C	0.413514	-2.695139	3.121450
H	0.768276	-2.185678	4.031037
H	1.245316	-2.735211	2.404521
H	0.162069	-3.733127	3.390218
C	-1.938390	-1.931606	3.605045
H	-2.184021	-2.937195	3.980263
H	-2.859323	-1.480667	3.211581
H	-1.607134	-1.337466	4.470298
C	-0.060300	-2.668748	-2.467280
H	0.390435	-1.667651	-2.351227
C	-0.935035	-2.616366	-3.728831
H	-1.797400	-1.943578	-3.605696
H	-1.321895	-3.610677	-3.999381
H	-0.342026	-2.258088	-4.584232

C	1.087641	-3.681825	-2.640440
H	1.762333	-3.682148	-1.772045
H	1.678651	-3.447121	-3.538885
H	0.684843	-4.700539	-2.754787
C	3.967435	-0.927724	0.153933
C	4.577809	-0.897104	1.419399
C	5.973431	-1.020406	1.441488
H	6.498193	-1.004764	2.398747
C	6.700087	-1.163749	0.259628
H	7.787837	-1.256844	0.301094
C	6.051084	-1.195784	-0.975586
H	6.638376	-1.312591	-1.887934
C	4.657662	-1.081132	-1.062223
C	3.792159	-0.725686	2.713496
H	2.718207	-0.701552	2.470034
C	4.001914	-1.915670	3.663974
H	3.376125	-1.801816	4.562789
H	5.049458	-1.986130	3.995838
H	3.736140	-2.867444	3.179454
C	4.134089	0.611820	3.392965
H	3.969232	1.459470	2.710195
H	5.188895	0.638992	3.708813
H	3.513064	0.758843	4.290634
C	3.952196	-1.074557	-2.413374
H	2.874290	-1.220639	-2.240133
C	4.129369	0.288836	-3.106809
H	3.775797	1.114427	-2.470659
H	3.573361	0.316695	-4.057228
H	5.191578	0.477278	-3.330000
C	4.410343	-2.226264	-3.320634
H	4.291756	-3.200870	-2.824300
H	5.465659	-2.119085	-3.615135
H	3.815141	-2.237546	-4.246840
C	-2.583159	0.423410	-0.077095
C	-3.349878	0.355500	1.088788
H	-3.123970	0.981349	1.952984
C	-4.416395	-0.544220	1.145865
H	-5.028311	-0.647714	2.041980
C	-3.956321	-1.238763	-1.147187
H	-4.202157	-1.883179	-1.990187
C	-2.899671	-0.330683	-1.208138
H	-2.313163	-0.249185	-2.123842
I	-2.388328	3.746372	-0.277640
N	3.455677	2.143750	0.039719
C	-4.677688	-1.350948	0.039719
O	-6.393028	-2.421231	1.156184
O	-5.762288	-3.226083	-0.754463
N	-5.702131	-2.410789	0.153027

87

TS-decomp-cis+14-N SCF Done: -2021.12671202 A.U.

Au	-1.543723	-1.249744	-0.373840
N	0.638563	-1.158800	-2.673788
N	0.402582	0.645425	1.221572
N	1.703472	0.788316	1.358793
N	2.239873	0.248159	0.294899
C	0.882471	-1.922166	-3.741805
H	0.084696	-1.998873	-4.487478
C	2.098680	-2.592370	-3.900221
H	2.299267	-3.214162	-4.774042
C	3.040249	-2.432248	-2.879729
H	4.016137	-2.927250	-2.920797
C	1.606909	-1.075533	-1.755320
C	1.298021	-0.274516	-0.552407
C	0.063034	-0.003070	0.053656
C	-0.468683	1.188162	2.242840
C	-0.671413	2.587641	2.257118
C	-1.565425	3.075630	3.217373
H	-1.766110	4.145920	3.271263
C	-2.211696	2.213344	4.105749
H	-2.910194	2.619859	4.840905
C	-1.962920	0.842206	4.074705
H	-2.455958	0.189872	4.796853
C	-1.071691	0.289063	3.142434
C	0.061423	3.533329	1.309876
H	0.260595	2.979668	0.375245
C	1.414892	3.960051	1.915872
H	1.947962	4.626319	1.220758
H	2.061491	3.100981	2.138021
H	1.250565	4.512283	2.854484
C	-0.746302	4.784338	0.930956
H	-0.836922	5.479678	1.779586
H	-1.759144	4.549925	0.578268
H	-0.227208	5.329875	0.128093
C	-0.718858	-1.192609	3.201210
H	-0.211268	-1.471945	2.265082
C	-1.952694	-2.097010	3.329276
H	-2.691423	-1.892538	2.540043
H	-2.448303	-1.975586	4.304629
H	-1.657343	-3.153041	3.240028
C	0.284461	-1.439853	4.344159
H	1.191371	-0.828180	4.225261
H	0.584045	-2.499447	4.367819
H	-0.164378	-1.192008	5.319028
C	3.678270	0.343075	0.120763
C	4.168772	1.441879	-0.606781
C	5.559822	1.505976	-0.768233
H	6.000124	2.333588	-1.326366

C 6.391377 0.531325 -0.215653
H 7.473088 0.606429 -0.349467
C 5.858461 -0.534532 0.511960
H 6.528627 -1.280748 0.941506
C 4.476333 -0.660456 0.693344
C 3.249770 2.495702 -1.216090
H 2.285690 2.453646 -0.684488
C 3.785848 3.923814 -1.035329
H 3.025645 4.655309 -1.350723
H 4.682193 4.104479 -1.648433
H 4.045465 4.126345 0.014181
C 2.973646 2.195021 -2.701083
H 2.531036 1.197711 -2.844773
H 3.907318 2.228930 -3.284338
H 2.281055 2.939537 -3.124787
C 3.872638 -1.800516 1.500095
H 2.806795 -1.877828 1.229344
C 4.505266 -3.159736 1.173023
H 4.467787 -3.362068 0.092095
H 3.963426 -3.963962 1.694367
H 5.555763 -3.213253 1.498491
C 3.948310 -1.483433 3.005049
H 3.457393 -0.525777 3.237379
H 4.996941 -1.410555 3.334627
H 3.460003 -2.275479 3.594055
C -1.637546 0.830684 -0.800808
C -1.285831 1.321355 -2.063843
H -0.562173 0.781543 -2.674254
C -1.890041 2.493825 -2.517839
H -1.649575 2.917925 -3.492984
C -3.184010 2.636306 -0.448174
H -3.926081 3.164437 0.150253
C -2.574259 1.473945 0.022312
H -2.846462 1.081362 1.001015
I -2.651320 -3.593593 -0.468155
N 2.792403 -1.680769 -1.804313
C -2.816221 3.136941 -1.696093
O -3.223402 4.740143 -3.307569
O -4.018333 5.068604 -1.315301
N -3.406938 4.421091 -2.147928

102
TS-CD-cis+14-N SCF Done: -3414.71029907 A.U.
Au -0.725547 -1.006318 -1.341362
N 1.406445 -1.497969 -1.834989
N 0.100975 1.500630 0.535270
N 1.243587 2.055423 0.919146
N 2.189184 1.394757 0.292282
C 1.886560 -2.539573 -2.527120

H	1.173439	-3.226105	-2.975664
C	3.267728	-2.651443	-2.770149
H	3.656699	-3.484366	-3.355213
C	4.095220	-1.700848	-2.198530
H	5.180291	-1.732712	-2.333544
C	2.308042	-0.632381	-1.291164
C	1.663762	0.408896	-0.491020
C	0.286793	0.481608	-0.349618
C	-1.133750	2.014991	1.103037
C	-1.724336	3.127330	0.476466
C	-2.905321	3.617618	1.052356
H	-3.413798	4.469675	0.599618
C	-3.439337	3.032336	2.199739
H	-4.365493	3.423391	2.626347
C	-2.817530	1.932208	2.792269
H	-3.265842	1.482830	3.678307
C	-1.649976	1.380045	2.250930
C	-1.080113	3.805910	-0.727073
H	-0.479339	3.047368	-1.258495
C	-0.123137	4.919971	-0.257963
H	0.380488	5.384785	-1.119570
H	0.646715	4.536171	0.426979
H	-0.683507	5.705548	0.273093
C	-2.096201	4.355365	-1.737202
H	-2.656226	5.211141	-1.329884
H	-2.822465	3.588271	-2.040271
H	-1.574373	4.712686	-2.638296
C	-0.986674	0.146499	2.857585
H	-0.611155	-0.467600	2.021182
C	-1.952569	-0.749998	3.644659
H	-2.872461	-0.959184	3.078929
H	-2.245447	-0.292006	4.602502
H	-1.477282	-1.718335	3.856861
C	0.208641	0.537366	3.748427
H	0.940242	1.165594	3.222268
H	0.724957	-0.364135	4.117419
H	-0.135837	1.104301	4.627611
C	3.573823	1.786564	0.456468
C	4.071079	2.783266	-0.403368
C	5.418474	3.131013	-0.242357
H	5.856747	3.895314	-0.886159
C	6.209760	2.513854	0.727090
H	7.257601	2.803397	0.835069
C	5.674426	1.532753	1.561368
H	6.308812	1.064993	2.316965
C	4.334716	1.139957	1.444455
C	3.221587	3.414770	-1.500176
H	2.163017	3.205429	-1.280902
C	3.361851	4.942989	-1.552436

H	2.666258	5.360092	-2.296997
H	4.376135	5.251615	-1.849103
H	3.136107	5.401420	-0.577969
C	3.548587	2.771789	-2.861004
H	3.428608	1.678236	-2.829388
H	4.591426	2.979980	-3.148859
H	2.892432	3.175377	-3.648408
C	3.765094	0.059117	2.355359
H	2.696223	-0.064872	2.116101
C	4.457447	-1.292896	2.105583
H	4.334341	-1.624524	1.063036
H	4.059737	-2.065844	2.786379
H	5.537715	-1.232915	2.307304
C	3.844569	0.470966	3.834526
H	3.341754	1.433780	4.008713
H	4.889438	0.570141	4.167027
H	3.362471	-0.285396	4.474029
C	-2.576840	-0.237565	-0.997268
C	-3.123477	0.651428	-1.930935
H	-2.611428	0.875341	-2.868893
C	-4.329387	1.292799	-1.639708
H	-4.779034	2.008550	-2.327849
C	-4.436480	0.116791	0.494828
H	-4.951669	-0.047372	1.440638
C	-3.241875	-0.537722	0.192015
H	-2.797876	-1.237022	0.898491
N	3.614115	-0.695921	-1.436097
O	-0.912185	-2.690848	0.825104
O	-0.971317	-3.889554	2.706018
C	-1.363840	-3.642877	1.556291
C	-2.389234	-4.563533	0.885953
H	-3.263445	-4.684848	1.542196
H	-1.914478	-5.556124	0.817996
C	-2.771751	-4.042564	-0.495526
C	-1.598101	-3.621024	-1.295408
H	-0.649139	-4.161614	-1.141330
C	-1.669793	-2.709508	-2.373747
H	-0.961796	-2.876609	-3.195009
H	-2.668609	-2.393344	-2.694514
H	-3.247687	-4.845225	-1.098242
H	-3.514114	-3.228675	-0.456130
K	1.515071	-3.400742	1.979713
Cl	1.712170	-4.572206	-0.676739
C	-4.945877	1.041331	-0.414326
N	-6.132227	1.838369	-0.034095
O	-6.523366	1.740108	1.118151
O	-6.621150	2.560478	-0.885175

102

TS-CD-trans+14-N SCF Done: -3414.70055010 A.U.

Au	1.366416	-0.041169	0.447774
N	0.740718	-1.915066	-0.441576
N	-1.767850	1.052885	0.601908
N	-2.959316	0.479506	0.453566
N	-2.706566	-0.761833	0.115552
C	1.530119	-2.840230	-0.995874
H	2.602773	-2.642242	-0.998199
C	0.981291	-4.001603	-1.535977
H	1.616621	-4.754491	-2.003330
C	-0.401264	-4.164872	-1.421323
H	-0.896675	-5.073431	-1.778549
C	-0.609020	-2.120698	-0.453207
C	-1.364096	-0.981025	0.041250
C	-0.730843	0.205120	0.378989
C	-1.694397	2.461381	0.919519
C	-1.673466	2.830523	2.278829
C	-1.537290	4.198250	2.555639
H	-1.512374	4.542776	3.590056
C	-1.446067	5.130625	1.520482
H	-1.340867	6.191828	1.758712
C	-1.501785	4.726307	0.185607
H	-1.442446	5.476412	-0.603228
C	-1.632734	3.371871	-0.156388
C	-1.874061	1.801017	3.387289
H	-1.460802	0.839816	3.033875
C	-3.380283	1.583705	3.639232
H	-3.534665	0.806954	4.404000
H	-3.904071	1.274383	2.723710
H	-3.845739	2.515245	3.997903
C	-1.155062	2.152643	4.696974
H	-1.613215	3.023012	5.191310
H	-0.088671	2.381952	4.542378
H	-1.224278	1.310448	5.402249
C	-1.771014	2.908411	-1.601422
H	-1.284531	1.923890	-1.701009
C	-1.107287	3.844139	-2.619009
H	-0.046048	4.031348	-2.403204
H	-1.624340	4.814842	-2.684527
H	-1.174801	3.390581	-3.620957
C	-3.260659	2.721534	-1.956653
H	-3.772737	2.044863	-1.259561
H	-3.351211	2.295306	-2.966977
H	-3.789255	3.688382	-1.938054
C	-3.796070	-1.713566	0.008922
C	-4.154535	-2.399894	1.186604
C	-5.194644	-3.330050	1.076551
H	-5.509634	-3.893787	1.956304
C	-5.838074	-3.544624	-0.144296

H	-6.649844	-4.273397	-0.206265
C	-5.457628	-2.834537	-1.281336
H	-5.974722	-3.010693	-2.226560
C	-4.417043	-1.894536	-1.236704
C	-3.424737	-2.190017	2.509658
H	-2.866194	-1.242766	2.449224
C	-4.383020	-2.051053	3.701647
H	-3.818834	-1.802560	4.614446
H	-4.926760	-2.987007	3.902275
H	-5.124791	-1.256916	3.528930
C	-2.400243	-3.315694	2.743064
H	-1.693099	-3.398353	1.903435
H	-2.907565	-4.288680	2.840220
H	-1.827510	-3.137843	3.667646
C	-4.003499	-1.132411	-2.485625
H	-3.147908	-0.486806	-2.242364
C	-3.514427	-2.088719	-3.584904
H	-2.706491	-2.732157	-3.209361
H	-3.104816	-1.509093	-4.424833
H	-4.330422	-2.727138	-3.961528
C	-5.144340	-0.231448	-2.986143
H	-5.475955	0.472351	-2.207396
H	-6.019239	-0.823496	-3.299713
H	-4.809376	0.354204	-3.856458
C	3.324734	-0.727742	0.557037
C	4.268552	-0.367164	-0.413503
H	4.000472	0.366964	-1.175771
C	5.545310	-0.937915	-0.383995
H	6.304269	-0.681254	-1.123675
C	4.931391	-2.215388	1.603147
H	5.219123	-2.933060	2.371778
C	3.655983	-1.643319	1.566851
H	2.925889	-1.932332	2.327782
N	-1.183709	-3.231964	-0.875277
O	2.268008	1.715671	-1.331742
O	2.325507	3.459416	-2.729700
C	2.358816	2.964633	-1.591984
C	2.559426	3.908410	-0.399201
H	2.167674	4.901092	-0.658992
H	3.649090	4.028043	-0.263355
C	1.918929	3.395576	0.885127
C	2.441845	2.106437	1.391776
H	3.498877	1.887431	1.203243
C	1.742603	1.256128	2.243715
H	2.298555	0.554576	2.872547
H	0.755956	1.561563	2.596478
H	2.142352	4.103151	1.712476
H	0.822679	3.366099	0.817455
K	1.504408	1.333598	-4.019823

Cl	-0.511897	-0.409515	-2.935056
C	5.853888	-1.853978	0.621759
O	7.441567	-3.259172	1.543387
O	7.987420	-2.130249	-0.224089
N	7.207488	-2.464520	0.649862

21
ACETAT-15 SCF Done: -1023.45161125 A.U.

C	0.727198	0.126597	0.027575
O	1.064404	-1.066667	0.269801
O	1.538969	1.064369	-0.224142
C	-0.784335	0.493266	0.020443
C	-1.626614	-0.756321	0.383396
H	-1.396323	-1.552768	-0.340992
H	-1.267013	-1.126685	1.358640
C	-3.109523	-0.527398	0.449202
H	-3.464723	0.138602	1.246467
C	-4.013432	-1.064759	-0.376853
H	-3.712095	-1.735707	-1.189126
H	-5.083448	-0.859851	-0.274461
K	3.496955	-0.492932	-0.033172
C	-1.129088	0.993825	-1.394796
H	-0.971801	0.197930	-2.142244
H	-2.184015	1.305516	-1.455919
H	-0.484274	1.844466	-1.658567
C	-0.994385	1.634656	1.033455
H	-2.021655	2.030604	0.986384
H	-0.808554	1.289336	2.064776
H	-0.294149	2.453450	0.815506

110
TS-CD-cis+15-N SCF Done: -3403.39269856 A.U.

Au	-0.889590	-0.414206	-1.427657
O	-5.182756	3.923137	-0.449290
N	0.941336	-1.672657	-1.863733
N	0.727589	1.566197	0.547042
N	1.977312	1.655660	0.986066
N	2.637350	0.699669	0.374077
C	1.038838	-2.801774	-2.575571
H	0.144366	-3.169923	-3.071599
C	2.288903	-3.419747	-2.757763
H	2.370388	-4.330421	-3.350372
C	3.378567	-2.854820	-2.117329
H	4.379178	-3.288582	-2.203072
C	2.069606	-1.210463	-1.255315
C	1.818152	-0.013346	-0.452359
C	0.559726	0.563507	-0.358136
C	-0.249692	2.495517	1.085135
C	-0.314657	3.774164	0.504760

C	-1.225031	4.676044	1.072625
H	-1.318534	5.680944	0.659308
C	-2.012402	4.304549	2.160725
H	-2.710670	5.025522	2.592683
C	-1.929195	3.018290	2.696048
H	-2.570429	2.746894	3.534561
C	-1.048270	2.068004	2.165317
C	0.590416	4.181617	-0.651779
H	0.899209	3.261262	-1.176476
C	1.862962	4.869457	-0.121127
H	2.536938	5.130628	-0.951635
H	2.411694	4.223716	0.579907
H	1.602827	5.798519	0.410517
C	-0.118307	5.063988	-1.689127
H	-0.345965	6.063689	-1.287727
H	-1.061438	4.610200	-2.025483
H	0.531477	5.209918	-2.565952
C	-0.963331	0.649322	2.722279
H	-0.833347	-0.033613	1.865912
C	-2.236579	0.189721	3.445791
H	-3.139572	0.388397	2.850302
H	-2.357218	0.693054	4.418369
H	-2.191463	-0.894514	3.622137
C	0.251853	0.487720	3.656574
H	1.194994	0.789880	3.181705
H	0.346427	-0.560533	3.984796
H	0.129939	1.107346	4.559084
C	4.060926	0.552745	0.595699
C	4.925255	1.310723	-0.215908
C	6.298189	1.139120	0.003075
H	7.013007	1.699213	-0.601838
C	6.766580	0.261790	0.981824
H	7.842042	0.146930	1.135780
C	5.874114	-0.467525	1.766983
H	6.259831	-1.145167	2.531368
C	4.490064	-0.341718	1.589852
C	4.412268	2.223912	-1.323345
H	3.347479	2.430102	-1.133900
C	5.122243	3.585055	-1.348256
H	4.655025	4.239609	-2.100215
H	6.184888	3.488638	-1.619632
H	5.062547	4.088245	-0.371577
C	4.511790	1.512829	-2.685981
H	3.984991	0.546749	-2.676533
H	5.563999	1.310908	-2.942653
H	4.081862	2.139914	-3.483081
C	3.525100	-1.149317	2.448913
H	2.497236	-0.890459	2.147499
C	3.709163	-2.659656	2.217301

H	3.549741	-2.927775	1.161313
H	3.016254	-3.237857	2.853435
H	4.723346	-2.987971	2.490979
C	3.657584	-0.783368	3.936305
H	3.514171	0.295722	4.095558
H	4.650825	-1.053659	4.327230
H	2.904887	-1.319174	4.536163
C	-2.354050	0.964292	-1.101720
C	-2.473332	2.054933	-1.972471
H	-1.810812	2.168041	-2.833444
C	-3.449001	3.019468	-1.732502
H	-3.564035	3.882257	-2.391441
C	-4.302827	2.913839	-0.618055
C	-4.184668	1.805909	0.235524
H	-4.827969	1.693167	1.107863
C	-3.205041	0.835699	-0.010312
H	-3.094294	-0.007575	0.669426
C	-6.067951	3.898609	0.653655
H	-6.675000	4.810622	0.586798
H	-5.521217	3.899009	1.613996
H	-6.736513	3.019688	0.621909
N	3.265749	-1.753504	-1.345113
O	-1.704722	-2.030207	0.607166
O	-2.269354	-3.086290	2.483773
C	-2.506385	-2.733663	1.315774
C	-3.792331	-3.235458	0.618208
C	-3.883769	-2.573568	-0.769690
C	-2.618166	-2.562723	-1.535882
H	-1.925472	-3.408392	-1.406000
C	-2.338581	-1.631203	-2.561050
H	-1.706324	-1.990679	-3.382708
H	-3.152314	-0.966790	-2.870844
H	-4.596199	-3.141911	-1.404021
H	-4.293802	-1.551691	-0.719105
K	0.238613	-3.574214	1.768656
Cl	0.032108	-4.780568	-0.874332
C	-5.014394	-2.864836	1.470685
H	-5.941497	-3.252077	1.018507
H	-4.906407	-3.292806	2.476981
H	-5.114702	-1.771780	1.575794
C	-3.669829	-4.769896	0.499974
H	-3.574326	-5.206925	1.504151
H	-4.567690	-5.192963	0.021214
H	-2.781990	-5.070702	-0.079539

110

TS-CD-trans+15-N SCF Done: -3403.38298946 A.U.

Au	1.292572	-0.468035	0.440797
O	6.557994	-3.791873	0.345344

N	0.339676	-2.159726	-0.531377
N	-1.602049	1.155394	0.662148
N	-2.877451	0.811655	0.500281
N	-2.848978	-0.438630	0.103806
C	0.954689	-3.189401	-1.119293
H	2.046051	-3.183758	-1.109648
C	0.208818	-4.212488	-1.702581
H	0.700720	-5.048765	-2.200055
C	-1.180969	-4.130400	-1.590927
H	-1.828663	-4.921574	-1.982295
C	-1.024566	-2.121657	-0.544393
C	-1.566380	-0.888014	0.004126
C	-0.731927	0.149541	0.390890
C	-1.271297	2.512961	1.031724
C	-1.174666	2.820855	2.402747
C	-0.788771	4.129259	2.726968
H	-0.692880	4.423583	3.772694
C	-0.538709	5.068542	1.725082
H	-0.239876	6.083152	1.999142
C	-0.677875	4.731690	0.377790
H	-0.494171	5.489354	-0.384168
C	-1.051070	3.436583	-0.011011
C	-1.553964	1.803519	3.475190
H	-1.320821	0.797955	3.083023
C	-3.073906	1.850732	3.733453
H	-3.363150	1.086785	4.471655
H	-3.647039	1.673729	2.812381
H	-3.363722	2.836966	4.129119
C	-0.779829	1.971009	4.789818
H	-1.071882	2.890979	5.319327
H	0.309809	2.009082	4.632896
H	-0.998258	1.129476	5.464909
C	-1.284174	3.060450	-1.469346
H	-0.998136	2.004920	-1.608772
C	-0.456133	3.883755	-2.463148
H	0.622470	3.847483	-2.255537
H	-0.767837	4.940308	-2.485824
H	-0.622016	3.491472	-3.479490
C	-2.783839	3.177522	-1.811388
H	-3.411609	2.587992	-1.129708
H	-2.962008	2.812045	-2.833889
H	-3.115425	4.227033	-1.753648
C	-4.090453	-1.177836	-0.021140
C	-4.552492	-1.837985	1.135312
C	-5.742973	-2.563244	1.009317
H	-6.142605	-3.098298	1.872500
C	-6.428905	-2.609138	-0.206339
H	-7.358414	-3.178730	-0.281073
C	-5.941059	-1.931413	-1.321825

H	-6.492078	-1.973222	-2.263380
C	-4.748778	-1.194253	-1.260430
C	-3.781679	-1.816931	2.451630
H	-3.061734	-0.984435	2.416994
C	-4.685445	-1.556359	3.665618
H	-4.075197	-1.450103	4.576487
H	-5.386843	-2.386640	3.840908
H	-5.273711	-0.635484	3.536673
C	-2.974928	-3.117281	2.622853
H	-2.305472	-3.292090	1.766777
H	-3.648868	-3.985846	2.694172
H	-2.367310	-3.082441	3.541669
C	-4.220095	-0.467055	-2.486199
H	-3.258934	0.002500	-2.234407
C	-3.924311	-1.448856	-3.630884
H	-3.238317	-2.239383	-3.295956
H	-3.427991	-0.917031	-4.455506
H	-4.845988	-1.916005	-4.015293
C	-5.185081	0.644986	-2.928558
H	-5.373802	1.363908	-2.116514
H	-6.156741	0.234429	-3.247752
H	-4.760008	1.196634	-3.781631
C	3.096131	-1.495433	0.493661
C	4.064832	-1.296420	-0.502285
H	3.917646	-0.511742	-1.246754
C	5.210419	-2.089109	-0.522605
H	5.978228	-1.952578	-1.287231
C	5.416787	-3.082753	0.455107
C	4.451258	-3.269494	1.458937
H	4.580280	-4.027422	2.231597
C	3.297428	-2.470651	1.472622
H	2.555360	-2.642330	2.258080
C	6.856336	-4.795654	1.297527
H	7.825843	-5.220665	1.007798
H	6.097226	-5.598478	1.295440
H	6.936800	-4.378899	2.317408
N	-1.788091	-3.096115	-1.005591
O	2.453137	1.190854	-1.276244
O	2.896892	2.926845	-2.606167
C	2.844931	2.387375	-1.486587
C	3.351571	3.191569	-0.259969
C	2.585293	2.744685	1.000715
C	2.781330	1.359325	1.478484
H	3.764105	0.904604	1.324751
C	1.875425	0.669437	2.278393
H	2.235883	-0.158999	2.895491
H	0.973165	1.182850	2.613890
H	2.959247	3.350922	1.855060
H	1.512903	2.978744	0.923999

K	1.636610	1.036119	-3.922574
Cl	-0.679865	-0.373041	-2.975440
C	3.126113	4.695852	-0.465332
H	3.570803	5.276364	0.359476
H	3.578288	5.014841	-1.412786
H	2.052363	4.932332	-0.510130
C	4.863154	2.906317	-0.140615
H	5.384243	3.307675	-1.021850
H	5.287281	3.389279	0.755752
H	5.084377	1.828282	-0.095314

21
ACETAT-16 SCF Done: -1023.45038094 A.U.

C	-0.890295	0.462074	-0.046672
O	-1.201733	-0.760478	0.065170
O	-1.725451	1.408566	-0.091487
C	0.587147	0.884910	-0.134344
H	0.664764	1.504484	-1.044648
H	0.753178	1.587504	0.700233
C	1.698259	-0.197003	-0.131508
C	3.017216	0.534737	-0.316661
H	3.075669	1.114750	-1.248668
C	4.076677	0.548199	0.497752
H	4.098389	-0.000819	1.442911
H	4.975060	1.118464	0.243553
K	-3.659724	-0.171023	0.119165
C	1.661626	-1.001320	1.176918
H	2.460895	-1.759982	1.198376
H	1.795252	-0.342991	2.051251
H	0.690625	-1.504656	1.267381
C	1.518465	-1.148733	-1.336859
H	0.548966	-1.659888	-1.265904
H	1.549642	-0.590047	-2.288042
H	2.325217	-1.899269	-1.361796

110
TS-CD-cis+16-N SCF Done: -3403.38453310 A.U.

Au	-0.988316	-0.616980	-1.205055
O	-5.377128	3.678125	-0.499739
N	0.889875	-1.796109	-1.710540
N	0.650056	1.581466	0.505326
N	1.925948	1.786637	0.809061
N	2.595453	0.845457	0.185434
C	1.013870	-2.973215	-2.335482
H	0.110082	-3.450201	-2.707497
C	2.287092	-3.502081	-2.609980
H	2.388179	-4.448950	-3.139691
C	3.383098	-2.800389	-2.136940
H	4.404036	-3.157579	-2.300898

C	2.027961	-1.202393	-1.260612
C	1.757632	0.025988	-0.513549
C	0.472584	0.515309	-0.323999
C	-0.332838	2.465782	1.106344
C	-0.600536	3.680707	0.452730
C	-1.508465	4.543660	1.082176
H	-1.757397	5.497639	0.616159
C	-2.091001	4.201472	2.301573
H	-2.784473	4.896103	2.782051
C	-1.809250	2.977332	2.909394
H	-2.295268	2.722616	3.851479
C	-0.928838	2.063223	2.318098
C	0.103778	4.070244	-0.841202
H	0.400320	3.138941	-1.353473
C	1.385676	4.867969	-0.531595
H	1.921794	5.114200	-1.461159
H	2.068433	4.307094	0.123224
H	1.134758	5.812732	-0.023686
C	-0.799703	4.841566	-1.812799
H	-1.034984	5.849917	-1.437957
H	-1.746381	4.310885	-1.986761
H	-0.288170	4.969882	-2.779363
C	-0.648051	0.700260	2.944082
H	-0.526160	-0.018337	2.116544
C	-1.807671	0.162541	3.794324
H	-2.771785	0.254046	3.272472
H	-1.891237	0.693497	4.755976
H	-1.660090	-0.908127	3.997441
C	0.650993	0.726942	3.771765
H	1.514480	1.077596	3.189855
H	0.880606	-0.276386	4.168282
H	0.544203	1.401883	4.635642
C	4.041895	0.821951	0.258456
C	4.749530	1.581865	-0.691290
C	6.147442	1.530221	-0.616725
H	6.744885	2.097406	-1.332320
C	6.788207	0.762886	0.356609
H	7.879729	0.739963	0.396030
C	6.047537	0.026581	1.280799
H	6.567032	-0.565136	2.037229
C	4.646900	0.035569	1.252498
C	4.050023	2.371842	-1.791389
H	2.992847	2.495115	-1.509668
C	4.623521	3.785884	-1.962449
H	4.031110	4.345714	-2.702577
H	5.661934	3.765736	-2.327704
H	4.604792	4.345211	-1.015077
C	4.087375	1.585033	-3.114924
H	3.658460	0.578455	-2.997518

H	5.124770	1.461777	-3.464789
H	3.525700	2.117728	-3.898636
C	3.850287	-0.783300	2.260559
H	2.778559	-0.604077	2.074284
C	4.111213	-2.289437	2.078108
H	3.833294	-2.629479	1.068834
H	3.553291	-2.877696	2.827447
H	5.174248	-2.531786	2.227729
C	4.132409	-0.329735	3.702212
H	3.938389	0.745810	3.828095
H	5.179893	-0.519546	3.983163
H	3.493221	-0.876878	4.413318
C	-2.476591	0.752843	-0.940387
C	-2.834770	1.578540	-2.012397
H	-2.350086	1.488725	-2.987312
C	-3.829102	2.539258	-1.832664
H	-4.132889	3.196214	-2.649950
C	-4.456067	2.694514	-0.582310
C	-4.091778	1.851222	0.479683
H	-4.557195	1.943306	1.460209
C	-3.099213	0.882555	0.294465
H	-2.813858	0.227776	1.116170
C	-6.052842	3.898423	0.723204
H	-6.739689	4.737567	0.553269
H	-5.350763	4.165600	1.533478
H	-6.637132	3.013322	1.032541
N	3.252764	-1.653670	-1.439009
O	-1.613607	-1.971715	1.032778
O	-1.873112	-3.158214	2.902511
C	-2.166508	-2.894687	1.722018
C	-3.144038	-3.817345	0.990816
H	-3.990123	-4.060677	1.650123
H	-2.578970	-4.753203	0.840718
C	-3.634520	-3.281360	-0.365304
C	-2.460662	-2.909731	-1.210165
H	-1.593699	-3.578846	-1.080220
C	-2.422278	-1.967226	-2.248826
H	-1.779413	-2.201862	-3.107297
H	-3.330532	-1.411593	-2.495554
K	0.644959	-3.182881	2.229131
Cl	0.401015	-4.705428	-0.248296
C	-4.687351	-2.182208	-0.188911
H	-4.285254	-1.350482	0.399667
H	-5.044097	-1.781265	-1.149528
H	-5.558775	-2.593649	0.341739
C	-4.250978	-4.467972	-1.181505
H	-3.516041	-5.264966	-1.372902
H	-5.070360	-4.899597	-0.586452
H	-4.662467	-4.122367	-2.141659

110

TS-CD-trans+16-N SCF Done: -3403.37737875 A.U.

Au	1.354597	-0.337200	0.304825
O	6.587390	-3.720415	0.358751
N	0.444611	-2.065732	-0.629650
N	-1.621520	1.137733	0.680641
N	-2.878880	0.704522	0.611140
N	-2.791519	-0.541109	0.209997
C	1.079030	-3.060887	-1.256849
H	2.167817	-3.002337	-1.302932
C	0.356439	-4.117658	-1.807212
H	0.863526	-4.925902	-2.334823
C	-1.027959	-4.108877	-1.618492
H	-1.653938	-4.932767	-1.976391
C	-0.917565	-2.097236	-0.572478
C	-1.492099	-0.896556	0.012819
C	-0.702120	0.196855	0.340937
C	-1.404749	2.522143	1.033656
C	-1.232296	2.840459	2.393945
C	-1.065320	4.197023	2.705489
H	-0.931575	4.502450	3.744143
C	-1.087391	5.167985	1.701868
H	-0.966964	6.220977	1.967697
C	-1.258134	4.809311	0.364675
H	-1.265230	5.585287	-0.401876
C	-1.414830	3.467150	-0.009898
C	-1.302582	1.769071	3.477017
H	-0.985401	0.813964	3.024621
C	-2.757814	1.591748	3.953104
H	-2.826648	0.783994	4.698024
H	-3.428322	1.347342	3.116549
H	-3.124493	2.519949	4.419331
C	-0.366953	2.036013	4.664063
H	-0.692219	2.906694	5.254155
H	0.669750	2.219601	4.339942
H	-0.364378	1.170420	5.344443
C	-1.636884	3.052413	-1.459470
H	-1.325094	2.003156	-1.580139
C	-0.811565	3.868204	-2.464635
H	0.270144	3.815072	-2.266555
H	-1.103632	4.930047	-2.483942
H	-0.993724	3.478019	-3.479026
C	-3.137270	3.114802	-1.802711
H	-3.735756	2.519264	-1.098834
H	-3.307867	2.714497	-2.813634
H	-3.507183	4.152629	-1.769268
C	-3.979138	-1.373717	0.187158
C	-4.269182	-2.092498	1.364659

C	-5.402507	-2.913560	1.335280
H	-5.668608	-3.497119	2.218372
C	-6.201066	-2.992823	0.192578
H	-7.083392	-3.637431	0.193161
C	-5.885555	-2.252173	-0.944585
H	-6.523941	-2.319709	-1.827617
C	-4.756909	-1.419353	-0.980286
C	-3.384558	-2.027107	2.606172
H	-2.727551	-1.147882	2.517556
C	-4.193717	-1.834581	3.897532
H	-3.514653	-1.698621	4.754048
H	-4.824765	-2.708822	4.119861
H	-4.846687	-0.951343	3.833327
C	-2.480860	-3.270556	2.694261
H	-1.874815	-3.394709	1.784074
H	-3.085895	-4.183629	2.812072
H	-1.802013	-3.199388	3.559557
C	-4.420353	-0.623066	-2.230485
H	-3.477207	-0.084406	-2.064681
C	-4.172861	-1.549451	-3.431225
H	-3.386208	-2.281157	-3.200237
H	-3.821900	-0.957746	-4.289250
H	-5.088383	-2.088152	-3.726397
C	-5.512262	0.416717	-2.531530
H	-5.673330	1.093328	-1.678062
H	-6.475800	-0.064461	-2.765478
H	-5.224106	1.026797	-3.401800
C	3.169965	-1.353779	0.380931
C	4.092431	-1.297306	-0.675342
H	3.919517	-0.602466	-1.499634
C	5.224007	-2.109837	-0.650371
H	5.955996	-2.083683	-1.460604
C	5.463318	-2.979727	0.432427
C	4.545861	-3.020466	1.495835
H	4.700398	-3.680641	2.349304
C	3.405257	-2.202946	1.464011
H	2.700159	-2.262720	2.298471
C	6.914288	-4.608236	1.411446
H	7.861331	-5.087081	1.131530
H	6.142394	-5.387867	1.542143
H	7.051005	-4.073185	2.368227
N	-1.654165	-3.109570	-0.995936
O	2.555931	1.136635	-1.690723
O	2.830416	2.907006	-3.039138
C	3.041501	2.278555	-1.987803
C	3.951758	2.904836	-0.923483
H	4.219105	3.926961	-1.224126
H	4.880880	2.311696	-0.867378
C	3.254551	2.933650	0.452438

C	3.140731	1.573165	1.036526
H	4.041963	0.955152	0.966490
C	2.090105	1.079905	1.820192
H	2.349649	0.334713	2.581852
H	1.264258	1.749501	2.065493
K	1.247079	1.114007	-4.126516
Cl	-0.959407	-0.330461	-3.002351
C	1.924661	3.683667	0.369434
H	1.213155	3.128018	-0.252415
H	1.466399	3.844015	1.354977
H	2.077609	4.669692	-0.090957
C	4.205580	3.640558	1.480731
H	5.187898	3.148628	1.545121
H	4.362582	4.671767	1.130039
H	3.758922	3.682351	2.485592

90

A-ad+cis-CH SCF Done: -1915.23717347 A.U.

Au	0.852878	1.434643	-0.327068
N	-1.995337	2.912790	0.279009
N	-0.753857	-1.090778	-0.034309
N	-1.941114	-1.638063	0.134637
N	-2.780489	-0.628851	0.092119
C	-2.486601	4.149858	0.279387
H	-1.813151	4.940479	0.627394
C	-3.790038	4.457767	-0.131784
H	-4.148573	5.488653	-0.105460
C	-4.602679	3.416048	-0.578419
H	-5.621648	3.608849	-0.921603
C	-2.781358	1.906050	-0.137126
C	-2.142818	0.576097	-0.092858
C	-0.783330	0.271650	-0.175561
C	0.413713	-1.943840	-0.049086
C	0.772989	-2.550599	-1.264801
C	1.901941	-3.382287	-1.244105
H	2.225714	-3.872442	-2.164057
C	2.625404	-3.579674	-0.069528
H	3.516842	-4.210243	-0.077884
C	2.246541	-2.943062	1.113284
H	2.838661	-3.098372	2.015716
C	1.126651	-2.103774	1.155103
C	0.033576	-2.264098	-2.565568
H	-0.932793	-1.799623	-2.317492
C	-0.279714	-3.536380	-3.365895
H	-0.890607	-3.287669	-4.247739
H	-0.836416	-4.265459	-2.758090
H	0.635377	-4.026523	-3.733292
C	0.829012	-1.247111	-3.405711
H	1.810121	-1.660392	-3.691304

H	1.002969	-0.315792	-2.843368
H	0.285618	-0.994507	-4.330061
C	0.673233	-1.429195	2.444167
H	0.149990	-0.500399	2.166910
C	1.841079	-1.014567	3.348377
H	2.584475	-0.422335	2.792872
H	2.355514	-1.886344	3.783010
H	1.469889	-0.407365	4.188340
C	-0.333156	-2.322525	3.194224
H	-1.194775	-2.578939	2.559605
H	-0.709640	-1.811901	4.094742
H	0.143773	-3.264446	3.509394
C	-4.185985	-0.896222	0.295293
C	-4.937910	-1.330336	-0.814347
C	-6.300343	-1.566917	-0.594339
H	-6.929167	-1.909491	-1.417368
C	-6.867898	-1.368213	0.666831
H	-7.934313	-1.554629	0.814264
C	-6.087286	-0.938640	1.739151
H	-6.548863	-0.793412	2.717868
C	-4.714676	-0.695612	1.581859
C	-4.291483	-1.561271	-2.175496
H	-3.371213	-0.954164	-2.216827
C	-3.875156	-3.036621	-2.327556
H	-3.367647	-3.197122	-3.291552
H	-4.759502	-3.692780	-2.293830
H	-3.192189	-3.344543	-1.522341
C	-5.174104	-1.106659	-3.346345
H	-5.503275	-0.062317	-3.227214
H	-6.071824	-1.735360	-3.452331
H	-4.614280	-1.181796	-4.291265
C	-3.865654	-0.234430	2.760427
H	-2.815346	-0.187203	2.434031
C	-4.267293	1.179756	3.215233
H	-4.190748	1.905536	2.391611
H	-3.613294	1.520050	4.033358
H	-5.304829	1.197602	3.585321
C	-3.922954	-1.240348	3.922078
H	-3.622516	-2.247415	3.595316
H	-4.935877	-1.309996	4.348673
H	-3.244614	-0.925930	4.730748
C	-4.095208	2.115071	-0.587690
I	3.248019	2.740068	-0.441442
C	4.259138	0.851011	-0.112982
C	4.298030	-0.076868	-1.158107
C	4.787583	0.571148	1.141402
H	3.873919	0.149621	-2.137437
C	4.872786	-1.319071	-0.922851
C	5.362384	-0.685328	1.371839

H	4.751673	1.302255	1.950924
H	4.911261	-2.073821	-1.709137
C	5.393054	-1.642365	0.344965
H	5.766232	-0.901733	2.360280
O	5.871419	-2.895045	0.479995
C	6.442678	-3.300816	1.712722
H	6.772905	-4.338035	1.574688
H	5.704046	-3.262968	2.533349
H	7.313443	-2.677478	1.980077
H	-4.708371	1.288374	-0.941285

90

TS-AB+cis-CH SCF Done: -1915.21210376 A.U.

Au	-1.113778	1.360636	-0.015788
N	1.267054	2.820701	-0.180708
N	0.642854	-1.237351	0.119304
N	1.889648	-1.676008	0.111271
N	2.624445	-0.591610	0.009163
C	1.500564	4.124521	-0.274514
H	0.620074	4.776263	-0.288284
C	2.794968	4.655145	-0.352181
H	2.947106	5.733333	-0.428107
C	3.869287	3.766706	-0.329249
H	4.896539	4.133509	-0.387457
C	2.295999	1.952910	-0.157337
C	1.859741	0.547702	-0.048269
C	0.532419	0.119572	0.023596
C	-0.426457	-2.207738	0.216561
C	-0.900721	-2.543362	1.498427
C	-1.895817	-3.527096	1.562272
H	-2.298525	-3.826872	2.530359
C	-2.381932	-4.132716	0.403356
H	-3.157925	-4.896714	0.480783
C	-1.898621	-3.758660	-0.849982
H	-2.293878	-4.241348	-1.745177
C	-0.906214	-2.776766	-0.976296
C	-0.312741	-1.912549	2.754748
H	0.060790	-0.912099	2.480731
C	0.887135	-2.740430	3.254715
H	1.354598	-2.257968	4.127472
H	1.652692	-2.857345	2.473882
H	0.560347	-3.748121	3.557132
C	-1.343434	-1.708121	3.872815
H	-1.683195	-2.664990	4.298952
H	-2.228065	-1.163730	3.510660
H	-0.896672	-1.129670	4.696047
C	-0.329562	-2.393264	-2.333476
H	0.114332	-1.388836	-2.237235
C	-1.393845	-2.298292	-3.435211

H	-2.216658	-1.631199	-3.138414
H	-1.822370	-3.283019	-3.679478
H	-0.945921	-1.903700	-4.360303
C	0.798128	-3.365782	-2.727431
H	1.589859	-3.392275	-1.964203
H	1.251435	-3.067128	-3.685549
H	0.404804	-4.388625	-2.840954
C	4.061835	-0.728536	-0.028884
C	4.760104	-0.677321	1.192171
C	6.158552	-0.737092	1.117378
H	6.747367	-0.695796	2.035829
C	6.808191	-0.850221	-0.112575
H	7.899450	-0.891258	-0.146514
C	6.076535	-0.922480	-1.299007
H	6.602440	-1.023585	-2.250286
C	4.675993	-0.868957	-1.287889
C	4.057342	-0.542743	2.537893
H	2.971596	-0.607881	2.369234
C	4.429815	-1.696156	3.483646
H	3.856218	-1.620299	4.420353
H	5.498509	-1.674071	3.748442
H	4.213161	-2.674016	3.027701
C	4.340004	0.827407	3.178806
H	4.029628	1.653734	2.520681
H	5.413394	0.952399	3.393225
H	3.794759	0.926350	4.130548
C	3.882496	-0.932408	-2.587919
H	2.813811	-1.011691	-2.336278
C	4.066987	0.354372	-3.411925
H	3.762111	1.246859	-2.843946
H	3.462456	0.312698	-4.331594
H	5.119941	0.487290	-3.707793
C	4.238168	-2.182203	-3.409050
H	4.095828	-3.101819	-2.821801
H	5.283148	-2.157645	-3.755407
H	3.598005	-2.243658	-4.302706
C	3.626737	2.393145	-0.230668
I	-3.141141	3.134103	-0.040337
C	-3.280708	0.636107	0.006464
C	-3.652375	0.098396	1.244346
C	-3.674322	0.061266	-1.199932
H	-3.383104	0.593751	2.177534
C	-4.351310	-1.101786	1.258960
C	-4.380611	-1.145466	-1.171691
H	-3.424138	0.525808	-2.154259
H	-4.629081	-1.574365	2.202535
C	-4.708443	-1.743235	0.056803
H	-4.657978	-1.608956	-2.117761
O	-5.350538	-2.919744	0.186729

C	-5.785096	-3.612778	-0.969542
H	-6.286136	-4.522858	-0.616129
H	-4.933773	-3.895089	-1.614212
H	-6.501203	-3.012557	-1.557399
H	4.456688	1.690767	-0.212317

90
TS-AB+trans-CH SCF Done: -1915.21140697 A.U.

Au	1.448821	-0.101466	-0.024886
N	0.132630	-2.550683	-0.141803
N	-1.378367	1.249663	0.077551
N	-2.677469	1.013278	0.064258
N	-2.774193	-0.297440	0.007189
C	0.602193	-3.790397	-0.222600
H	1.692013	-3.894477	-0.255747
C	-0.236667	-4.911704	-0.266849
H	0.185689	-5.915881	-0.334899
C	-1.614782	-4.702031	-0.222094
H	-2.307218	-5.546291	-0.253295
C	-1.197456	-2.334496	-0.099990
C	-1.541853	-0.904145	-0.019127
C	-0.606427	0.127131	0.029204
C	-0.887683	2.607488	0.142057
C	-0.725233	3.184936	1.413367
C	-0.227852	4.494656	1.448550
H	-0.088457	4.994558	2.408049
C	0.096351	5.168342	0.270513
H	0.481427	6.189642	0.320939
C	-0.055820	4.549246	-0.971186
H	0.217736	5.091778	-1.876856
C	-0.548921	3.241555	-1.067119
C	-1.106489	2.435650	2.684224
H	-1.052611	1.356478	2.463699
C	-2.560087	2.755266	3.080912
H	-2.853785	2.182617	3.974793
H	-3.260427	2.509894	2.268525
H	-2.671554	3.826857	3.311228
C	-0.137999	2.693761	3.846599
H	-0.207225	3.728339	4.217427
H	0.906033	2.508317	3.549334
H	-0.379537	2.031876	4.692881
C	-0.748930	2.549831	-2.410329
H	-0.644384	1.465376	-2.242627
C	0.309536	2.932252	-3.452764
H	1.328224	2.790485	-3.059536
H	0.209163	3.979979	-3.776778
H	0.195491	2.305970	-4.351266
C	-2.174882	2.802508	-2.936437
H	-2.936527	2.480423	-2.210494

H	-2.344001	2.256773	-3.878389
H	-2.330656	3.875461	-3.131944
C	-4.085397	-0.899553	-0.024801
C	-4.685931	-1.234963	1.203262
C	-5.927464	-1.882188	1.137476
H	-6.433167	-2.169073	2.061545
C	-6.527799	-2.161805	-0.091327
H	-7.495510	-2.668332	-0.117750
C	-5.909112	-1.790536	-1.286036
H	-6.400755	-2.004944	-2.237031
C	-4.666132	-1.142593	-1.283483
C	-4.027627	-0.940913	2.546252
H	-3.131402	-0.328360	2.364250
C	-4.948557	-0.116766	3.460303
H	-4.422966	0.145245	4.391709
H	-5.853270	-0.679028	3.739789
H	-5.265031	0.817102	2.971999
C	-3.565399	-2.239044	3.231418
H	-2.865274	-2.804527	2.596765
H	-4.421292	-2.895509	3.456051
H	-3.057318	-2.012906	4.182071
C	-3.991827	-0.742349	-2.590319
H	-3.066487	-0.197002	-2.349705
C	-3.591862	-1.979086	-3.413535
H	-2.925466	-2.645177	-2.843337
H	-3.066182	-1.675284	-4.332400
H	-4.476760	-2.563323	-3.712479
C	-4.874072	0.219239	-3.403282
H	-5.140586	1.110464	-2.815206
H	-5.808202	-0.265387	-3.728138
H	-4.341278	0.552678	-4.307319
C	-2.112181	-3.397943	-0.137388
I	3.289595	1.973230	-0.198499
C	3.475851	-0.636615	-0.073813
C	3.935453	-1.197318	-1.287023
C	4.030766	-1.048900	1.151952
H	3.572729	-0.817638	-2.244114
C	4.833427	-2.251523	-1.252615
C	4.934987	-2.109039	1.185204
H	3.746339	-0.551376	2.081172
H	5.181687	-2.730216	-2.169864
C	5.342672	-2.720669	-0.019056
H	5.328358	-2.440134	2.145995
O	6.215910	-3.731645	-0.098097
C	6.839533	-4.235648	1.074592
H	7.512021	-5.036851	0.744224
H	6.097581	-4.651863	1.777999
H	7.428645	-3.453892	1.583695
H	-3.184707	-3.218543	-0.102360

90
B+cis-CH SCF Done: -1915.24575752 A.U.

Au	-1.047028	1.436992	-0.103327
O	-5.764702	-2.531396	0.301273
N	0.912683	2.629735	-0.196263
N	0.530523	-1.384547	0.157082
N	1.797691	-1.771486	0.195905
N	2.503898	-0.670977	0.081276
C	1.026932	3.956036	-0.299431
H	0.089822	4.517733	-0.340534
C	2.273124	4.588402	-0.352179
H	2.324637	5.675030	-0.436223
C	3.423915	3.804205	-0.296065
H	4.413584	4.264595	-0.335621
C	2.027661	1.853198	-0.140041
C	1.701988	0.430865	-0.023234
C	0.391295	-0.034923	0.022739
C	-0.491954	-2.412290	0.241615
C	-1.133603	-2.611566	1.480475
C	-2.098112	-3.625261	1.524975
H	-2.640376	-3.814799	2.450870
C	-2.377618	-4.403526	0.400592
H	-3.130339	-5.192612	0.466519
C	-1.713941	-4.178792	-0.803369
H	-1.951320	-4.795292	-1.671471
C	-0.756432	-3.161808	-0.917984
C	-0.748115	-1.808409	2.718721
H	-0.568516	-0.769647	2.392412
C	0.560931	-2.353305	3.327152
H	0.888205	-1.721642	4.168098
H	1.378720	-2.401731	2.595116
H	0.405900	-3.374061	3.710789
C	-1.842178	-1.756515	3.791845
H	-1.983461	-2.736504	4.274178
H	-2.806778	-1.437143	3.375183
H	-1.555407	-1.046881	4.582952
C	-0.026460	-2.912437	-2.232768
H	0.367447	-1.881844	-2.209634
C	-0.948638	-3.003857	-3.458048
H	-1.841103	-2.370076	-3.346467
H	-1.287712	-4.035710	-3.637907
H	-0.409246	-2.680397	-4.361640
C	1.171090	-3.870661	-2.376322
H	1.873182	-3.768111	-1.536256
H	1.716549	-3.672309	-3.311881
H	0.824891	-4.916098	-2.400799
C	3.947973	-0.737134	0.081256
C	4.611231	-0.574790	1.311974

C	6.012594	-0.572725	1.271225
H	6.575944	-0.453555	2.198797
C	6.695914	-0.725256	0.064403
H	7.788379	-0.716869	0.056503
C	5.998285	-0.899195	-1.132519
H	6.553231	-1.027044	-2.063380
C	4.597207	-0.916501	-1.156225
C	3.871040	-0.411968	2.633876
H	2.789205	-0.433011	2.432302
C	4.169970	-1.587629	3.579520
H	3.578460	-1.494517	4.503360
H	5.233658	-1.612821	3.863920
H	3.922169	-2.551636	3.109880
C	4.181665	0.941742	3.294895
H	3.935427	1.783446	2.628435
H	5.246619	1.024713	3.563192
H	3.596498	1.058415	4.220314
C	3.833269	-1.080040	-2.465743
H	2.788247	-1.325773	-2.223163
C	3.827122	0.236991	-3.263448
H	3.378652	1.061652	-2.687936
H	3.251208	0.121607	-4.194967
H	4.852231	0.536329	-3.534448
C	4.372394	-2.242660	-3.312848
H	4.387968	-3.182786	-2.741613
H	5.392541	-2.046017	-3.677232
H	3.735117	-2.389928	-4.198322
C	-2.664295	0.192337	-0.018961
C	-3.434926	0.117699	1.144672
H	-3.235231	0.768122	1.997566
C	-4.480338	-0.800014	1.209693
H	-5.097123	-0.887226	2.106231
C	-4.766055	-1.640769	0.118792
C	-4.012907	-1.520001	-1.059164
H	-4.216302	-2.146100	-1.927161
C	-2.959072	-0.600554	-1.122168
H	-2.372429	-0.530031	-2.039769
C	-6.153169	-3.371745	-0.767716
H	-6.983113	-3.986314	-0.395715
H	-5.328558	-4.035558	-1.085161
H	-6.500412	-2.789115	-1.639626
I	-2.704039	3.517191	-0.291690
C	3.306063	2.414547	-0.188411
H	4.189168	1.779927	-0.144151

90

TS-decomp+cis-CH SCF Done: -1915.21078647 A.U.

Au	-1.761625	-0.785554	-0.420850
O	-2.166349	4.730545	-3.027097

N	0.284541	-1.766934	-2.228915
N	0.478931	0.603741	1.199411
N	1.779836	0.529301	1.390645
N	2.255720	-0.071759	0.326899
C	0.282697	-2.527014	-3.318779
H	-0.683661	-2.949443	-3.613345
C	1.445448	-2.788366	-4.056728
H	1.405898	-3.424381	-4.943039
C	2.640512	-2.212244	-3.626963
H	3.569709	-2.380936	-4.175730
C	1.433874	-1.218713	-1.798489
C	1.273554	-0.426002	-0.564305
C	0.080190	0.033278	0.014884
C	-0.343583	1.241442	2.205523
C	-0.332668	2.650803	2.270677
C	-1.184174	3.236489	3.216797
H	-1.221775	4.322453	3.308818
C	-1.986847	2.451168	4.045299
H	-2.650343	2.931936	4.768058
C	-1.934779	1.058387	3.978228
H	-2.545777	0.466424	4.659789
C	-1.094556	0.410603	3.061916
C	0.602990	3.499809	1.415485
H	0.794607	2.948026	0.479095
C	1.948324	3.700823	2.144450
H	2.641612	4.282512	1.516856
H	2.427411	2.745275	2.396561
H	1.791989	4.258041	3.081576
C	0.017267	4.863101	1.021672
H	-0.054174	5.540617	1.886642
H	-0.980793	4.765261	0.575264
H	0.673545	5.350970	0.284659
C	-0.905437	-1.103005	3.090779
H	-0.677035	-1.444953	2.069566
C	-2.150799	-1.875270	3.537905
H	-3.034436	-1.595225	2.945974
H	-2.373547	-1.712362	4.604000
H	-1.987930	-2.954707	3.400883
C	0.316588	-1.449757	3.966042
H	1.224434	-0.928044	3.626120
H	0.512452	-2.533277	3.938545
H	0.137441	-1.162100	5.014165
C	3.677945	-0.319280	0.251790
C	4.501134	0.721927	-0.225382
C	5.863378	0.423163	-0.353700
H	6.551847	1.185922	-0.718970
C	6.359365	-0.838116	-0.011228
H	7.426219	-1.045562	-0.122753
C	5.512700	-1.826786	0.488141

H	5.923961	-2.797483	0.771801
C	4.138521	-1.589663	0.638834
C	3.933601	2.087464	-0.606457
H	3.051528	2.263303	0.026220
C	4.907089	3.239336	-0.323423
H	4.398502	4.203585	-0.476326
H	5.774893	3.222786	-1.001210
H	5.279128	3.209191	0.711785
C	3.462663	2.114577	-2.073279
H	2.656405	1.391584	-2.265979
H	4.294828	1.882933	-2.757159
H	3.078189	3.113372	-2.332821
C	3.219040	-2.664419	1.206720
H	2.191815	-2.268389	1.232358
C	3.198040	-3.922826	0.322523
H	2.923055	-3.682833	-0.715735
H	2.466889	-4.648884	0.710453
H	4.181903	-4.417903	0.306465
C	3.600137	-2.998734	2.659388
H	3.580090	-2.100841	3.295607
H	4.609779	-3.434498	2.719474
H	2.894203	-3.732282	3.078952
C	-1.382838	1.184832	-1.069981
C	-0.865058	1.424617	-2.351331
H	-0.268267	0.671535	-2.864485
C	-1.148599	2.631272	-2.975972
H	-0.771244	2.848424	-3.976879
C	-1.949687	3.602259	-2.336785
C	-2.454993	3.343002	-1.049108
H	-3.090970	4.064054	-0.536944
C	-2.147615	2.143480	-0.400719
H	-2.537451	1.961732	0.600607
C	-2.978600	5.756545	-2.476443
H	-3.008415	6.559701	-3.223073
H	-2.549802	6.150276	-1.538380
H	-4.005759	5.399510	-2.287803
I	-3.545113	-2.684939	-0.269639
C	2.645967	-1.410767	-2.481531
H	3.571269	-0.954064	-2.139851

105
TS-CD+cis-CH SCF Done: -3308.78572702 A.U.

Au	-0.908477	-0.814589	-1.326079
O	-6.033526	2.523484	-0.415562
N	1.136716	-1.652635	-1.771421
N	0.272474	1.558181	0.508631
N	1.475281	1.949323	0.905427
N	2.320892	1.140637	0.302939
C	1.407008	-2.766051	-2.452687

H	0.565371	-3.330881	-2.844901
C	2.725609	-3.181577	-2.707500
H	2.892226	-4.102227	-3.266425
C	3.772076	-2.425447	-2.206657
H	4.808237	-2.721192	-2.383412
C	2.163516	-0.918358	-1.244627
C	1.672676	0.223531	-0.475089
C	0.315815	0.507352	-0.355564
C	-0.878395	2.267495	1.039239
C	-1.231401	3.479524	0.420891
C	-2.309289	4.179664	0.979814
H	-2.627398	5.124168	0.536889
C	-2.979938	3.683782	2.096033
H	-3.813821	4.248572	2.519793
C	-2.607456	2.467396	2.670110
H	-3.160644	2.092339	3.531022
C	-1.546680	1.715151	2.150813
C	-0.455262	4.036314	-0.766932
H	0.049863	3.190769	-1.264473
C	0.631754	5.016485	-0.285393
H	1.220142	5.391850	-1.137167
H	1.319550	4.540668	0.428832
H	0.171635	5.882334	0.216657
C	-1.357471	4.692978	-1.821439
H	-1.805020	5.628255	-1.450865
H	-2.174301	4.022787	-2.124649
H	-0.767134	4.950537	-2.714527
C	-1.141243	0.372673	2.753705
H	-0.883867	-0.299619	1.918132
C	-2.266065	-0.324626	3.530703
H	-3.203486	-0.350611	2.956457
H	-2.471854	0.177759	4.489461
H	-1.981375	-1.365600	3.741488
C	0.099147	0.519060	3.656833
H	0.940959	1.004520	3.145217
H	0.430793	-0.469201	4.016278
H	-0.137965	1.130008	4.542214
C	3.742942	1.315588	0.485692
C	4.408081	2.200019	-0.386470
C	5.797801	2.299654	-0.235170
H	6.363089	2.965961	-0.888710
C	6.470675	1.561629	0.739988
H	7.554354	1.657967	0.839290
C	5.771632	0.708461	1.594423
H	6.314617	0.151067	2.360320
C	4.381856	0.561638	1.487410
C	3.675866	2.976392	-1.475658
H	2.601969	2.971732	-1.235938
C	4.104557	4.450007	-1.534406

H	3.482728	4.992845	-2.262679
H	5.151522	4.561214	-1.856534
H	3.992501	4.940862	-0.556052
C	3.849015	2.286876	-2.841819
H	3.482878	1.248651	-2.824819
H	4.910186	2.263112	-3.137316
H	3.292857	2.830974	-3.621236
C	3.625478	-0.362733	2.434115
H	2.561580	-0.349949	2.149745
C	4.115522	-1.817171	2.322111
H	3.986953	-2.216158	1.303005
H	3.569283	-2.462178	3.031576
H	5.181168	-1.908428	2.581600
C	3.707374	0.151449	3.881519
H	3.342177	1.186414	3.957430
H	4.743540	0.129630	4.253767
H	3.096286	-0.475196	4.549842
C	-2.635414	0.223001	-1.016530
C	-2.993658	1.235261	-1.916453
H	-2.375798	1.462125	-2.788256
C	-4.152988	1.974155	-1.693285
H	-4.455083	2.771314	-2.375319
C	-4.957660	1.721667	-0.566198
C	-4.598952	0.691531	0.316972
H	-5.198283	0.469997	1.199791
C	-3.434751	-0.052642	0.086916
H	-3.139663	-0.832266	0.787615
C	-6.887180	2.341389	0.697053
H	-7.676924	3.098954	0.611833
H	-6.349156	2.487512	1.651261
H	-7.350898	1.338624	0.697262
C	3.492121	-1.275666	-1.447427
O	-1.377557	-2.495049	0.804728
O	-1.640672	-3.607255	2.720864
C	-1.967508	-3.354571	1.551631
C	-3.076663	-4.160410	0.868525
H	-3.966633	-4.195558	1.513492
H	-2.700911	-5.194522	0.796499
C	-3.377864	-3.591975	-0.514277
C	-2.146470	-3.303642	-1.288864
H	-1.284895	-3.977247	-1.141561
C	-2.090549	-2.368830	-2.348409
H	-1.415132	-2.621207	-3.175680
H	-3.036264	-1.912750	-2.660500
H	-3.932437	-4.330843	-1.130256
H	-4.019578	-2.696649	-0.477928
K	0.902401	-3.526798	1.952672
Cl	0.892984	-4.890937	-0.583309
H	4.295922	-0.670135	-1.032900

105
TS-CD+trans-CH SCF Done: -3308.78237320 A.U.

Au	1.496917	-0.115388	0.374247
O	7.200164	-2.614600	0.196159
N	0.790989	-1.867200	-0.682871
N	-1.599385	1.027280	0.761975
N	-2.809279	0.492525	0.639796
N	-2.599518	-0.709616	0.150362
C	1.587329	-2.743239	-1.311803
H	2.657894	-2.540087	-1.277272
C	1.069019	-3.859855	-1.963045
H	1.747388	-4.548152	-2.468955
C	-0.311352	-4.071942	-1.942983
H	-0.746788	-4.941859	-2.439090
C	-0.560104	-2.036779	-0.689581
C	-1.270053	-0.935343	-0.053798
C	-0.594911	0.197696	0.379137
C	-1.468329	2.395310	1.209304
C	-1.332472	2.629420	2.591528
C	-1.143624	3.961245	2.987050
H	-1.029405	4.202458	4.044496
C	-1.112332	4.989185	2.042384
H	-0.966500	6.020449	2.373019
C	-1.276229	4.717637	0.683240
H	-1.258008	5.539134	-0.033487
C	-1.460137	3.404454	0.224012
C	-1.461807	1.496237	3.605647
H	-1.084757	0.574727	3.128239
C	-2.946747	1.260077	3.949104
H	-3.050967	0.413133	4.644972
H	-3.540832	1.043088	3.050079
H	-3.374345	2.153606	4.430794
C	-0.642411	1.712874	4.885159
H	-1.049534	2.533986	5.495190
H	0.412734	1.946850	4.672636
H	-0.671334	0.805855	5.508185
C	-1.701986	3.083145	-1.245173
H	-1.281140	2.087982	-1.461558
C	-1.040263	4.067499	-2.216395
H	0.042857	4.164607	-2.058546
H	-1.492031	5.071048	-2.161837
H	-1.193126	3.710750	-3.247104
C	-3.215850	2.999212	-1.526102
H	-3.722148	2.297945	-0.849418
H	-3.380016	2.651262	-2.557061
H	-3.691345	3.986583	-1.408114
C	-3.689443	-1.657715	0.062697
C	-3.840692	-2.545181	1.149736

C	-4.840565	-3.518501	1.032695
H	-4.992419	-4.234398	1.842771
C	-5.653660	-3.578315	-0.100943
H	-6.429262	-4.344829	-0.170531
C	-5.495306	-2.658113	-1.136151
H	-6.154803	-2.704210	-2.004696
C	-4.503622	-1.665828	-1.084282
C	-2.972544	-2.472649	2.402958
H	-2.389252	-1.539839	2.367027
C	-3.822458	-2.404721	3.682288
H	-3.174703	-2.254425	4.560456
H	-4.387253	-3.335217	3.847747
H	-4.541947	-1.573474	3.639969
C	-1.970634	-3.639408	2.457057
H	-1.320597	-3.657874	1.568592
H	-2.495248	-4.606892	2.506174
H	-1.329417	-3.558749	3.349391
C	-4.366674	-0.644088	-2.201850
H	-3.455345	-0.055554	-2.030160
C	-4.187331	-1.306756	-3.575292
H	-3.299655	-1.955176	-3.585271
H	-4.018370	-0.531545	-4.337091
H	-5.071801	-1.893735	-3.872325
C	-5.569085	0.316073	-2.186471
H	-5.678070	0.811113	-1.209104
H	-6.510401	-0.215479	-2.401843
H	-5.440040	1.096589	-2.952043
C	3.435236	-0.862252	0.392783
C	4.362829	-0.495671	-0.594894
H	4.097901	0.272815	-1.324317
C	5.613978	-1.108090	-0.631430
H	6.351846	-0.840232	-1.390818
C	5.966229	-2.085117	0.320612
C	5.040080	-2.439196	1.316466
H	5.281187	-3.188879	2.070091
C	3.780711	-1.821474	1.347295
H	3.073032	-2.121006	2.126080
C	7.643199	-3.588849	1.121893
H	8.664560	-3.858863	0.824352
H	7.010422	-4.494275	1.095885
H	7.662144	-3.192775	2.153022
C	-1.136305	-3.152770	-1.299200
O	2.354609	1.672393	-1.473321
O	2.321249	3.435214	-2.852811
C	2.449987	2.920448	-1.730568
C	2.774403	3.839078	-0.543525
H	2.408036	4.851431	-0.761150
H	3.874781	3.909336	-0.478652
C	2.200025	3.323618	0.770232

C	2.709059	2.008495	1.218111
H	3.740248	1.751639	0.951803
C	2.036461	1.168795	2.105146
H	2.618685	0.456480	2.697695
H	1.093207	1.516001	2.531200
H	2.501342	4.003658	1.596631
H	1.100470	3.333894	0.775821
K	1.293758	1.332332	-4.028772
Cl	-0.983238	-0.099058	-3.033110
H	-2.218231	-3.270399	-1.291220

86

A-ad+11-CH SCF Done: -1800.79110271 A.U.

Au	-1.254350	1.106002	0.084986
N	1.393333	2.983813	-0.231585
N	0.742339	-1.117132	0.364231
N	1.999541	-1.503149	0.289204
N	2.651538	-0.430708	-0.099635
C	1.672619	4.250272	-0.530069
H	0.968607	5.003757	-0.160837
C	2.798936	4.633601	-1.270009
H	2.988057	5.687716	-1.482200
C	3.656692	3.633855	-1.727581
H	4.540620	3.882456	-2.319279
C	2.226082	2.019849	-0.659203
C	1.822553	0.654376	-0.270454
C	0.540988	0.197529	0.041120
C	-0.265379	-2.063599	0.784498
C	-0.558110	-2.135342	2.160514
C	-1.539023	-3.058402	2.546134
H	-1.798512	-3.162209	3.600528
C	-2.181862	-3.858977	1.598715
H	-2.934555	-4.581236	1.924988
C	-1.874759	-3.743398	0.243617
H	-2.399134	-4.365040	-0.484423
C	-0.907081	-2.831262	-0.201485
C	0.194174	-1.288060	3.179564
H	0.545759	-0.380231	2.663372
C	1.436483	-2.043840	3.689234
H	2.014702	-1.413800	4.383163
H	2.096973	-2.337191	2.859551
H	1.140333	-2.959617	4.225450
C	-0.689478	-0.815024	4.341212
H	-1.001150	-1.648833	4.989655
H	-1.596372	-0.306978	3.977104
H	-0.131742	-0.106982	4.973272
C	-0.621205	-2.661444	-1.687816
H	0.248647	-1.995651	-1.796478
C	-1.810416	-1.978076	-2.387441

H	-2.043570	-1.006721	-1.923803
H	-2.718380	-2.598701	-2.326216
H	-1.585865	-1.807776	-3.452216
C	-0.253150	-3.991663	-2.363107
H	0.598529	-4.474049	-1.860378
H	0.022177	-3.820185	-3.415629
H	-1.097171	-4.699191	-2.356612
C	4.087070	-0.516626	-0.242481
C	4.883487	-0.070935	0.826101
C	6.272485	-0.141664	0.642772
H	6.936607	0.196959	1.440404
C	6.816119	-0.642952	-0.539153
H	7.901157	-0.691728	-0.657596
C	5.988290	-1.087858	-1.573095
H	6.436022	-1.482521	-2.486288
C	4.594456	-1.031601	-1.451966
C	4.295449	0.472601	2.122699
H	3.202372	0.345653	2.086826
C	4.793720	-0.319223	3.342982
H	4.297246	0.040940	4.257705
H	5.878843	-0.200327	3.488367
H	4.580722	-1.393665	3.237904
C	4.577715	1.978094	2.271787
H	4.188404	2.548927	1.415306
H	5.659801	2.172712	2.342666
H	4.105706	2.368592	3.186956
C	3.670321	-1.531484	-2.556567
H	2.692820	-1.037104	-2.425496
C	4.163819	-1.169443	-3.964364
H	4.363085	-0.090544	-4.061238
H	3.403918	-1.444230	-4.712012
H	5.086033	-1.710129	-4.228125
C	3.439545	-3.048110	-2.416024
H	3.029163	-3.299940	-1.427103
H	4.386952	-3.596296	-2.541211
H	2.734936	-3.404034	-3.183923
C	3.370187	2.301094	-1.424087
I	-3.789987	2.093398	0.127133
C	-4.676007	0.205930	-0.488975
C	-4.380971	-0.944366	0.240229
C	-5.514691	0.204786	-1.601512
H	-3.702830	-0.926777	1.095101
C	-4.970028	-2.146397	-0.167925
C	-6.089074	-1.011570	-1.991514
H	-5.723823	1.117500	-2.162126
H	-4.749241	-3.056548	0.391807
H	-6.750590	-1.033683	-2.860662
C	-5.820674	-2.181871	-1.276261
H	-6.276608	-3.125310	-1.584609

H	4.021424	1.505169	-1.779636

86

TS-AB-cis+11-CH SCF Done: -1800.76606042 A.U.

Au	-1.498854	-0.951666	-0.088437
N	0.634288	-2.819876	-0.538735
N	0.692146	1.210152	0.336794
N	1.992911	1.427341	0.372285
N	2.532428	0.273615	0.046582
C	0.660996	-4.119299	-0.813005
H	-0.305107	-4.635461	-0.817904
C	1.849248	-4.812322	-1.080295
H	1.828335	-5.881537	-1.299012
C	3.045078	-4.096210	-1.055938
H	3.996565	-4.592926	-1.258826
C	1.783464	-2.118667	-0.514300
C	1.584502	-0.692834	-0.191319
C	0.350777	-0.067040	-0.001988
C	-0.211985	2.288624	0.664161
C	-0.540998	3.201950	-0.355511
C	-1.417556	4.238222	-0.011021
H	-1.701447	4.976392	-0.762174
C	-1.933805	4.341226	1.282100
H	-2.613888	5.160281	1.528739
C	-1.588583	3.411603	2.261828
H	-1.999692	3.510844	3.268563
C	-0.708268	2.357046	1.977504
C	0.075743	3.102175	-1.745511
H	0.368719	2.051003	-1.905449
C	1.352350	3.961198	-1.825140
H	1.824481	3.860698	-2.814796
H	2.086008	3.665661	-1.061012
H	1.110965	5.024884	-1.669949
C	-0.905180	3.462157	-2.868934
H	-1.165372	4.532309	-2.859323
H	-1.836145	2.883016	-2.787746
H	-0.448824	3.251229	-3.848510
C	-0.309313	1.365964	3.065047
H	0.439210	0.676014	2.646601
C	-1.506200	0.513165	3.518240
H	-1.927500	-0.063462	2.679960
H	-2.308176	1.139706	3.940418
H	-1.197848	-0.203053	4.295798
C	0.355498	2.079790	4.254383
H	1.218317	2.680324	3.929157
H	0.709173	1.342820	4.992473
H	-0.349361	2.751182	4.769321
C	3.973552	0.177467	-0.001008
C	4.617921	0.511205	-1.205218

C	6.012141	0.357957	-1.230786
H	6.562158	0.594700	-2.143378
C	6.705110	-0.091528	-0.107171
H	7.791165	-0.202485	-0.150579
C	6.027346	-0.396289	1.076103
H	6.592941	-0.736066	1.944485
C	4.635379	-0.269675	1.160074
C	3.857781	0.975491	-2.442257
H	2.823097	1.203887	-2.144391
C	4.445275	2.269655	-3.026200
H	3.823852	2.622573	-3.863819
H	5.463104	2.116484	-3.417378
H	4.486656	3.068079	-2.270268
C	3.797291	-0.142828	-3.498434
H	3.319954	-1.051825	-3.100507
H	4.808075	-0.416323	-3.841273
H	3.221148	0.187211	-4.377164
C	3.873814	-0.551087	2.450260
H	2.850531	-0.857691	2.176452
C	4.471980	-1.699860	3.272339
H	4.597108	-2.610262	2.665735
H	3.809931	-1.940522	4.118175
H	5.452572	-1.434060	3.697131
C	3.758389	0.737422	3.288225
H	3.281853	1.548484	2.717780
H	4.755865	1.085172	3.600729
H	3.160168	0.556169	4.194873
C	3.022181	-2.727756	-0.769921
I	-3.830315	-2.270522	0.094298
C	-3.477151	0.119732	-0.590259
C	-3.642486	0.310925	-1.963004
C	-3.842012	1.068741	0.364283
H	-3.388692	-0.469630	-2.680669
C	-4.156736	1.541077	-2.390883
C	-4.349960	2.288202	-0.094507
H	-3.725329	0.878432	1.430422
H	-4.289312	1.714850	-3.461494
H	-4.621695	3.052893	0.635627
C	-4.511373	2.524734	-1.463255
H	-4.922820	3.475837	-1.807640
H	3.947725	-2.157221	-0.751021

86

TS-AB-trans+11-CH SCF Done: -1800.76437344 A.U.

Au	1.603696	0.582456	0.072451
N	-0.181356	2.668347	0.107799
N	-0.859491	-1.361765	-0.038539
N	-2.178602	-1.403973	-0.087945
N	-2.551815	-0.142847	-0.049384

C	0.023955	3.980260	0.149001
H	1.069025	4.306103	0.180405
C	-1.028487	4.905064	0.151141
H	-0.822541	5.976379	0.185563
C	-2.333641	4.414974	0.107988
H	-3.184789	5.099612	0.107896
C	-1.438348	2.181683	0.066009
C	-1.477399	0.709611	0.020987
C	-0.345904	-0.101700	0.029391
C	-0.089339	-2.584566	-0.044405
C	0.447125	-3.018331	-1.270934
C	1.211484	-4.191735	-1.238844
H	1.648225	-4.578231	-2.160326
C	1.425582	-4.875428	-0.040567
H	2.022685	-5.790482	-0.040593
C	0.893747	-4.399067	1.157922
H	1.084069	-4.944006	2.083629
C	0.124009	-3.228038	1.187111
C	0.168863	-2.269179	-2.568865
H	0.044358	-1.203762	-2.315367
C	-1.153211	-2.754092	-3.194679
H	-1.390667	-2.173137	-4.100162
H	-1.994337	-2.654994	-2.492159
H	-1.079461	-3.815428	-3.480625
C	1.325052	-2.344461	-3.573900
H	1.451715	-3.359683	-3.981327
H	2.277387	-2.038514	-3.113342
H	1.124707	-1.678687	-4.427748
C	-0.480293	-2.697653	2.481524
H	-0.669955	-1.619899	2.344637
C	0.468125	-2.825602	3.681656
H	1.451476	-2.378735	3.467006
H	0.627739	-3.876414	3.969502
H	0.039598	-2.313643	4.557344
C	-1.835346	-3.374892	2.759246
H	-2.536253	-3.224940	1.924457
H	-2.293855	-2.965610	3.673152
H	-1.704134	-4.459577	2.900614
C	-3.959789	0.172326	-0.091729
C	-4.545177	0.392085	-1.352503
C	-5.897219	0.761848	-1.360079
H	-6.398314	0.945279	-2.312658
C	-6.611832	0.894852	-0.168605
H	-7.664197	1.186978	-0.198596
C	-5.998162	0.647488	1.060564
H	-6.577396	0.744878	1.980830
C	-4.649673	0.271650	1.131195
C	-3.767672	0.249461	-2.655468
H	-2.747900	-0.088279	-2.415498

C	-4.389533	-0.826934	-3.560422
H	-3.776149	-0.966233	-4.464167
H	-5.402991	-0.544487	-3.886204
H	-4.457662	-1.794661	-3.040700
C	-3.640886	1.599498	-3.382196
H	-3.158138	2.356923	-2.744728
H	-4.627738	1.988626	-3.679309
H	-3.037411	1.487980	-4.296695
C	-3.979000	0.012675	2.475276
H	-2.972040	-0.389579	2.285979
C	-3.809991	1.320082	3.269353
H	-3.223912	2.063113	2.706185
H	-3.291624	1.127986	4.221913
H	-4.787726	1.770559	3.503950
C	-4.733379	-1.049635	3.290834
H	-4.840491	-1.987044	2.724790
H	-5.740183	-0.705597	3.574866
H	-4.187442	-1.271841	4.220882
C	-2.552121	3.034370	0.064465
I	3.951251	-0.883170	-0.067739
C	3.452273	1.623884	0.129448
C	3.744747	2.153989	1.398791
C	3.717650	2.345344	-1.048329
H	3.599298	1.554727	2.299440
C	4.216537	3.466615	1.484519
C	4.189723	3.655874	-0.939264
H	3.550155	1.893203	-2.027590
H	4.427416	3.898304	2.465913
H	4.379776	4.235290	-1.846018
C	4.439644	4.213706	0.321181
H	4.831809	5.230651	0.396311
H	-3.564235	2.636356	0.030658

86

B-cis+11-CH SCF Done: -1800.80167700 A.U.

Au	1.532108	-0.982901	-0.083807
N	-0.101986	-2.597702	-0.118500
N	-0.652927	1.404427	0.086723
N	-1.973941	1.492622	0.134093
N	-2.408469	0.254575	0.089961
C	0.091947	-3.917230	-0.183835
H	1.132033	-4.248407	-0.244163
C	-0.974139	-4.822227	-0.175531
H	-0.773879	-5.893336	-0.231189
C	-2.273484	-4.324675	-0.095402
H	-3.129615	-5.002840	-0.085938
C	-1.365047	-2.099435	-0.041576
C	-1.375291	-0.637478	0.020675
C	-0.207089	0.118818	0.018087

C	0.113940	2.635570	0.077262
C	0.695491	3.064380	1.286482
C	1.420974	4.260454	1.235930
H	1.904031	4.637662	2.136981
C	1.534225	4.983670	0.047377
H	2.102516	5.916617	0.036751
C	0.936650	4.526881	-1.125346
H	1.041589	5.107304	-2.042926
C	0.213925	3.326530	-1.143064
C	0.486706	2.297404	2.588434
H	0.519702	1.221619	2.344322
C	-0.903142	2.610417	3.180616
H	-1.088174	1.996987	4.076591
H	-1.716508	2.427228	2.464774
H	-0.960698	3.669537	3.477454
C	1.575555	2.549967	3.638014
H	1.518656	3.572447	4.043317
H	2.581663	2.400059	3.223390
H	1.444703	1.860166	4.485809
C	-0.456182	2.826703	-2.417922
H	-0.617372	1.740100	-2.311448
C	0.409196	3.022624	-3.671728
H	1.418142	2.602274	-3.544324
H	0.517976	4.086967	-3.931026
H	-0.060870	2.527478	-4.535386
C	-1.834892	3.490889	-2.595694
H	-2.489011	3.302172	-1.732050
H	-2.334620	3.110777	-3.500028
H	-1.723862	4.581554	-2.702613
C	-3.828161	-0.014434	0.126565
C	-4.416457	-0.249583	1.383505
C	-5.778700	-0.580661	1.383701
H	-6.284440	-0.770480	2.332479
C	-6.496795	-0.666460	0.190408
H	-7.556863	-0.929652	0.214764
C	-5.877426	-0.408947	-1.034101
H	-6.460648	-0.471198	-1.954411
C	-4.519734	-0.067568	-1.099526
C	-3.638098	-0.155564	2.690251
H	-2.598957	0.123318	2.457778
C	-4.204340	0.954894	3.591182
H	-3.592248	1.057606	4.500486
H	-5.235444	0.730120	3.906048
H	-4.212468	1.925496	3.072438
C	-3.591855	-1.509689	3.418699
H	-3.152234	-2.295735	2.784469
H	-4.599157	-1.841279	3.716121
H	-2.984196	-1.431627	4.333636
C	-3.837531	0.191228	-2.438339

H	-2.863722	0.664490	-2.240475
C	-3.568454	-1.130218	-3.181305
H	-2.941931	-1.813118	-2.586713
H	-3.050921	-0.938691	-4.134273
H	-4.511547	-1.652707	-3.408146
C	-4.633284	1.171657	-3.313658
H	-4.831051	2.114828	-2.782915
H	-5.598840	0.747450	-3.629721
H	-4.066670	1.405063	-4.228160
C	2.849745	0.581349	-0.005011
C	3.580566	0.802515	1.162299
H	3.481948	0.142608	2.025546
C	4.473464	1.880938	1.204333
H	5.056315	2.056441	2.112022
C	3.894715	2.469866	-1.068539
H	4.010854	3.116118	-1.941774
C	2.999888	1.394951	-1.126573
H	2.433548	1.207447	-2.039827
I	3.612535	-2.643621	-0.271578
C	-2.478727	-2.942722	-0.027041
C	4.629569	2.714713	0.094243
H	5.329693	3.552208	0.132926
H	-3.483401	-2.528641	0.034605

86

TS-decomp-cis+11-CH SCF Done: -1800.76451280 A.U.

Au	-1.910473	-0.443866	-0.450523
N	-0.011616	-2.399470	-1.452398
N	0.489438	1.130405	0.716064
N	1.740623	0.930905	1.073147
N	2.136104	-0.118352	0.392822
C	-0.110719	-3.535966	-2.133828
H	-1.125406	-3.905734	-2.314683
C	1.012002	-4.237320	-2.595327
H	0.891048	-5.173047	-3.144382
C	2.274316	-3.704084	-2.335901
H	3.176239	-4.211442	-2.685305
C	1.201483	-1.886223	-1.184273
C	1.144305	-0.639377	-0.398367
C	0.039093	0.200748	-0.191804
C	-0.239909	2.237495	1.298744
C	0.028650	3.534180	0.812309
C	-0.741010	4.573192	1.351938
H	-0.582219	5.595977	1.008685
C	-1.709564	4.319858	2.324071
H	-2.304525	5.145456	2.721667
C	-1.910662	3.028185	2.812120
H	-2.648201	2.861657	3.597347
C	-1.165587	1.946672	2.321935

C	1.141030	3.813601	-0.193122
H	1.275560	2.906106	-0.806806
C	2.463055	4.094374	0.552061
H	3.282684	4.254636	-0.166060
H	2.742697	3.268697	1.220453
H	2.367332	5.005395	1.163598
C	0.829410	4.966559	-1.157117
H	0.833508	5.939766	-0.642372
H	-0.145173	4.840593	-1.646752
H	1.600151	5.015175	-1.941528
C	-1.256734	0.565336	2.963271
H	-1.045443	-0.191409	2.191916
C	-2.641423	0.234712	3.529522
H	-3.430742	0.376405	2.776978
H	-2.880599	0.848779	4.411732
H	-2.671210	-0.817848	3.848450
C	-0.161131	0.428653	4.040618
H	0.843356	0.609144	3.627803
H	-0.176792	-0.582612	4.476587
H	-0.324328	1.153320	4.853967
C	3.479870	-0.612200	0.592314
C	4.520403	0.004024	-0.134115
C	5.796643	-0.549782	0.025190
H	6.643562	-0.119056	-0.509795
C	6.006400	-1.644441	0.869319
H	7.012048	-2.058365	0.974074
C	4.952572	-2.200953	1.592357
H	5.142369	-3.039967	2.264611
C	3.650530	-1.692134	1.475464
C	4.261337	1.190989	-1.059527
H	3.425248	1.762343	-0.631441
C	5.449714	2.157931	-1.141537
H	5.155034	3.065250	-1.690834
H	6.303340	1.718488	-1.681070
H	5.794402	2.461010	-0.141380
C	3.832130	0.730940	-2.466056
H	2.903041	0.142113	-2.448624
H	4.615533	0.113015	-2.933316
H	3.656200	1.602994	-3.115203
C	2.506050	-2.285151	2.289211
H	1.580252	-1.744325	2.038164
C	2.270987	-3.766739	1.949659
H	2.075194	-3.909122	0.876059
H	1.404980	-4.153007	2.509130
H	3.142809	-4.383572	2.219027
C	2.744014	-2.079383	3.795471
H	2.886610	-1.015155	4.037135
H	3.635344	-2.627405	4.139155
H	1.881474	-2.449355	4.371296

C	-1.102075	0.992122	-1.792347
C	-0.545177	0.559361	-3.000094
H	-0.089396	-0.425768	-3.087448
C	-0.613635	1.410238	-4.106888
H	-0.189944	1.082589	-5.059102
C	-1.775682	3.073078	-2.780571
H	-2.275076	4.041053	-2.697579
C	-1.696852	2.250486	-1.650821
H	-2.122134	2.577132	-0.702638
I	-4.045190	-1.780275	0.218486
C	2.382528	-2.507445	-1.621455
C	-1.231042	2.661580	-4.000362
H	-1.290899	3.316305	-4.872461
H	3.360791	-2.079347	-1.417282

101
TS-CD-cis+11-CH SCF Done: -3194.34166975 A.U.

Au	-1.342994	-0.221371	-1.287852
N	0.240126	-1.740185	-1.805959
N	0.664359	1.495048	0.556653
N	1.934362	1.407900	0.925384
N	2.411739	0.362900	0.283155
C	0.067099	-2.854346	-2.517234
H	-0.931466	-3.059565	-2.893604
C	1.135065	-3.716021	-2.822642
H	0.939228	-4.616435	-3.404613
C	2.397322	-3.411183	-2.341559
H	3.248207	-4.060534	-2.557612
C	1.476983	-1.448950	-1.299420
C	1.456888	-0.229801	-0.493699
C	0.302066	0.528020	-0.329003
C	-0.140846	2.555966	1.132981
C	-0.063760	3.823929	0.531804
C	-0.805441	4.849746	1.133352
H	-0.781981	5.853762	0.707844
C	-1.572066	4.602872	2.270710
H	-2.138649	5.417626	2.727719
C	-1.636393	3.323504	2.824613
H	-2.260235	3.153395	3.701963
C	-0.926609	2.254829	2.263775
C	0.816595	4.087412	-0.684137
H	0.968304	3.125634	-1.203241
C	2.198948	4.606840	-0.244872
H	2.851345	4.760136	-1.118713
H	2.696459	3.905078	0.440593
H	2.098415	5.571797	0.276865
C	0.167856	5.038202	-1.700105
H	0.097218	6.065743	-1.310770
H	-0.845069	4.705823	-1.969617

H	0.776186	5.082652	-2.616875
C	-1.004769	0.844750	2.842648
H	-1.015552	0.140667	1.993514
C	-2.288133	0.572504	3.638844
H	-3.186217	0.883897	3.085609
H	-2.288254	1.100893	4.605535
H	-2.383307	-0.505052	3.834833
C	0.225617	0.532224	3.716658
H	1.173790	0.703504	3.189461
H	0.198667	-0.516325	4.056627
H	0.233120	1.170798	4.614163
C	3.801962	-0.001652	0.425887
C	4.725749	0.597449	-0.453087
C	6.057238	0.173668	-0.344472
H	6.812767	0.601323	-1.005597
C	6.432837	-0.785561	0.597353
H	7.477411	-1.098564	0.663616
C	5.488579	-1.342355	1.460595
H	5.805312	-2.080459	2.200199
C	4.140779	-0.963332	1.395721
C	4.308093	1.618333	-1.505841
H	3.304290	1.987288	-1.246752
C	5.232470	2.844857	-1.529030
H	4.842106	3.596139	-2.232555
H	6.249665	2.586601	-1.862029
H	5.305259	3.312155	-0.535523
C	4.214781	0.958241	-2.894017
H	3.504311	0.116974	-2.898899
H	5.195999	0.570068	-3.210965
H	3.881420	1.689658	-3.646836
C	3.119151	-1.568530	2.351249
H	2.130538	-1.147752	2.109443
C	3.023517	-3.095785	2.188134
H	2.723709	-3.381496	1.166948
H	2.297031	-3.513766	2.905964
H	3.984563	-3.588130	2.401006
C	3.428849	-1.174218	3.805437
H	3.482375	-0.081278	3.918813
H	4.390128	-1.596807	4.137092
H	2.645453	-1.548717	4.482732
C	-2.572595	1.370916	-0.945580
C	-2.607521	2.403318	-1.887520
H	-1.977758	2.377195	-2.779682
C	-3.470516	3.487051	-1.678451
H	-3.506603	4.294327	-2.414431
C	-4.226811	2.499113	0.395413
H	-4.848278	2.535243	1.293795
C	-3.374061	1.406725	0.194286
H	-3.322792	0.595424	0.919021

C	2.576697	-2.261637	-1.552109
O	-2.346652	-1.650645	0.837728
O	-2.961869	-2.647103	2.736798
C	-3.198985	-2.248686	1.586746
C	-4.549375	-2.551656	0.930267
H	-5.367323	-2.252765	1.601827
H	-4.604896	-3.649030	0.839344
C	-4.643538	-1.884187	-0.437667
C	-3.413712	-2.070434	-1.244637
H	-2.864695	-3.020495	-1.125935
C	-3.033578	-1.208519	-2.299891
H	-2.517826	-1.682629	-3.144375
H	-3.748212	-0.429756	-2.587656
H	-5.451864	-2.344082	-1.044447
H	-4.894829	-0.812944	-0.374221
K	-0.599610	-3.510352	1.896996
Cl	-1.159901	-4.683565	-0.672556
C	-4.277651	3.537668	-0.538106
H	-4.944667	4.387641	-0.377289
H	3.555994	-2.004645	-1.152612

101
TS-CD-trans+11-CH SCF Done: -3194.33898519 A.U.

Au	-1.593313	-0.661956	-0.564408
N	-0.444607	-2.273360	0.310707
N	1.038306	1.366649	-0.546991
N	2.346141	1.189629	-0.396007
N	2.482177	-0.080852	-0.086721
C	-0.969477	-3.423749	0.756386
H	-2.050166	-3.534650	0.666537
C	-0.169538	-4.428260	1.295232
H	-0.633102	-5.349035	1.651802
C	1.211673	-4.230369	1.356151
H	1.865699	-5.002417	1.766822
C	0.895231	-2.047121	0.402727
C	1.272081	-0.706738	-0.024253
C	0.309955	0.236092	-0.359332
C	0.527963	2.690387	-0.823239
C	0.389163	3.078202	-2.170189
C	-0.167602	4.343426	-2.404870
H	-0.302001	4.696002	-3.428112
C	-0.542223	5.167298	-1.341431
H	-0.971920	6.150699	-1.547248
C	-0.363878	4.755796	-0.019786
H	-0.654495	5.422097	0.792885
C	0.180150	3.497544	0.279913
C	0.891604	2.193495	-3.307860
H	0.783820	1.142217	-2.987459
C	2.393371	2.446531	-3.551916

H	2.773007	1.778512	-4.340576
H	2.985936	2.277460	-2.641698
H	2.557986	3.486424	-3.875543
C	0.100965	2.354224	-4.613478
H	0.274023	3.338428	-5.075490
H	-0.984498	2.243027	-4.462990
H	0.422953	1.596226	-5.343822
C	0.444844	3.046039	1.710438
H	0.327626	1.951539	1.761631
C	-0.517294	3.646040	2.742202
H	-1.573445	3.451199	2.509266
H	-0.379906	4.733570	2.854265
H	-0.308178	3.201063	3.727780
C	1.903284	3.358402	2.101931
H	2.621240	2.937992	1.385091
H	2.120091	2.925229	3.089981
H	2.071331	4.446645	2.150586
C	3.799761	-0.677367	-0.031160
C	4.255836	-1.307716	-1.208768
C	5.493694	-1.958138	-1.134712
H	5.887339	-2.468219	-2.015959
C	6.236584	-1.953192	0.047683
H	7.201121	-2.465424	0.082579
C	5.765953	-1.286343	1.177905
H	6.370042	-1.272445	2.086927
C	4.527494	-0.625389	1.171379
C	3.460689	-1.299099	-2.511509
H	2.629653	-0.585123	-2.405115
C	4.308648	-0.805247	-3.694953
H	3.683783	-0.715263	-4.597592
H	5.126336	-1.503442	-3.931751
H	4.752502	0.179282	-3.484890
C	2.846608	-2.680321	-2.800125
H	2.190686	-3.012565	-1.980564
H	3.632618	-3.441692	-2.927128
H	2.250997	-2.653985	-3.726642
C	4.042584	0.130336	2.398343
H	3.007107	0.453437	2.225719
C	4.003023	-0.759080	3.649364
H	3.347076	-1.627389	3.493215
H	3.577095	-0.191243	4.489568
H	5.006564	-1.107712	3.943233
C	4.906618	1.384365	2.619239
H	4.909798	2.034248	1.730520
H	5.951927	1.117408	2.845150
H	4.517057	1.967876	3.467598
C	-3.221390	-1.924688	-0.844466
C	-4.257294	-1.979773	0.094834
H	-4.261326	-1.280599	0.933875

C	-5.278435	-2.927058	-0.066142
H	-6.089032	-2.973067	0.666075
C	-4.235740	-3.732652	-2.096738
H	-4.223500	-4.412065	-2.952951
C	-3.208845	-2.790583	-1.945027
H	-2.401853	-2.753908	-2.682665
C	1.751799	-3.028464	0.905010
O	-3.019453	0.511031	1.408281
O	-3.570694	1.994691	2.990975
C	-3.490279	1.630329	1.807092
C	-4.016718	2.577562	0.719301
H	-3.978061	3.609894	1.092409
H	-5.084125	2.337237	0.567915
C	-3.254715	2.446351	-0.593799
C	-3.333893	1.122474	-1.250271
H	-4.256454	0.548128	-1.111022
C	-2.403132	0.648297	-2.173007
H	-2.717541	-0.108394	-2.898239
H	-1.582982	1.306290	-2.466154
H	-3.701859	3.128791	-1.348962
H	-2.207419	2.767611	-0.499615
K	-2.017060	0.137876	3.989764
Cl	0.612056	-0.439245	3.000951
C	-5.268545	-3.802418	-1.156911
H	-6.068844	-4.536331	-1.277793
H	2.819776	-2.828275	0.967039

89

A-ad+12-CH SCF Done: -1840.08463477 A.U.

Au	1.063994	1.275629	-0.079877
N	-1.709623	2.948194	0.338745
N	-0.777845	-1.072853	-0.421084
N	-2.002885	-1.549604	-0.333697
N	-2.719088	-0.541099	0.109217
C	-2.072824	4.178963	0.691381
H	-1.438510	4.995575	0.330103
C	-3.200992	4.450631	1.476556
H	-3.461382	5.479571	1.732354
C	-3.967325	3.373888	1.921573
H	-4.848445	3.534622	2.546905
C	-2.454687	1.910174	0.754251
C	-1.964362	0.592869	0.303885
C	-0.661513	0.240476	-0.052492
C	0.278442	-1.931637	-0.906183
C	0.504039	-1.962991	-2.295918
C	1.523389	-2.811455	-2.747952
H	1.732354	-2.883013	-3.816319
C	2.269064	-3.577564	-1.849245
H	3.051315	-4.241418	-2.226144

C	2.028063	-3.500192	-0.478223
H	2.633473	-4.091590	0.211238
C	1.023270	-2.665901	0.031754
C	-0.353576	-1.154512	-3.261755
H	-0.769340	-0.299721	-2.704371
C	-1.538011	-2.002419	-3.763243
H	-2.189598	-1.406045	-4.420910
H	-2.144969	-2.377742	-2.925690
H	-1.177871	-2.871700	-4.336466
C	0.449131	-0.565078	-4.429331
H	0.817844	-1.345651	-5.113132
H	1.315352	0.013340	-4.071247
H	-0.189118	0.107610	-5.022760
C	0.800092	-2.545863	1.533319
H	-0.087935	-1.917002	1.699876
C	1.993198	-1.836694	2.198567
H	2.173914	-0.848720	1.747733
H	2.919089	-2.422044	2.085496
H	1.808751	-1.697491	3.275490
C	0.514044	-3.908444	2.183893
H	-0.343228	-4.407642	1.707842
H	0.285451	-3.779474	3.253628
H	1.382039	-4.582712	2.112325
C	-4.141314	-0.733104	0.278748
C	-4.992286	-0.302882	-0.753579
C	-6.368285	-0.477095	-0.543353
H	-7.073279	-0.154594	-1.312054
C	-6.847432	-1.060610	0.628701
H	-7.923373	-1.189278	0.768392
C	-5.966112	-1.486900	1.625545
H	-6.363281	-1.947233	2.531370
C	-4.582801	-1.328932	1.476856
C	-4.474870	0.330210	-2.039666
H	-3.375300	0.273730	-2.035003
C	-4.953114	-0.442284	-3.280203
H	-4.502950	-0.015180	-4.190075
H	-6.046783	-0.386444	-3.396490
H	-4.671063	-1.504372	-3.224356
C	-4.857749	1.818707	-2.118565
H	-4.482362	2.379371	-1.249301
H	-5.951755	1.944186	-2.153772
H	-4.437729	2.275632	-3.028297
C	-3.600368	-1.805920	2.540562
H	-2.661291	-1.242629	2.407075
C	-4.082184	-1.530129	3.971786
H	-4.349936	-0.471324	4.114566
H	-3.287747	-1.780866	4.691475
H	-4.959958	-2.140731	4.234598
C	-3.273025	-3.297364	2.336601

H	-2.870610	-3.483781	1.330039
H	-4.178729	-3.912259	2.460700
H	-2.528167	-3.633289	3.075012
C	-3.591988	2.077800	1.561497
I	3.532718	2.426227	-0.085857
C	4.494046	0.531531	0.363876
C	4.377178	-0.512647	-0.550912
C	5.178856	0.395659	1.567988
H	3.814821	-0.402718	-1.479493
C	4.983064	-1.731594	-0.238077
C	5.772013	-0.838866	1.854930
H	5.251913	1.218140	2.281554
H	4.889532	-2.556926	-0.946801
C	5.688015	-1.918067	0.961933
H	6.308292	-0.956951	2.800123
C	6.349348	-3.237582	1.267272
H	7.336591	-3.298826	0.778165
H	6.507851	-3.374387	2.346899
H	5.750879	-4.083532	0.895385
H	-4.170822	1.223208	1.906108

89
TS-AB-cis+12-CH SCF Done: -1840.05962453 A.U.

Au	-1.342645	-1.151406	-0.089019
N	0.929328	-2.789975	-0.667417
N	0.659415	1.174837	0.428446
N	1.938786	1.502885	0.449870
N	2.566506	0.422662	0.042863
C	1.057035	-4.063180	-1.024286
H	0.141701	-4.664726	-1.016110
C	2.286533	-4.626215	-1.391269
H	2.350023	-5.678232	-1.675329
C	3.412723	-3.804437	-1.380822
H	4.392437	-4.197877	-1.661074
C	2.010558	-1.987899	-0.653147
C	1.700561	-0.608079	-0.232234
C	0.422507	-0.106055	0.020425
C	-0.320358	2.158389	0.834181
C	-0.702659	3.131503	-0.105840
C	-1.622184	4.096795	0.327637
H	-1.944348	4.880692	-0.359228
C	-2.126839	4.072838	1.627059
H	-2.838068	4.839150	1.944944
C	-1.737609	3.077601	2.524928
H	-2.149499	3.077064	3.534824
C	-0.819730	2.088015	2.149391
C	-0.106427	3.173419	-1.507528
H	0.264510	2.162016	-1.743584
C	1.095515	4.135888	-1.549152

H	1.551522	4.141739	-2.551304
H	1.866815	3.848148	-0.819973
H	0.774540	5.163715	-1.315908
C	-1.138408	3.527353	-2.587086
H	-1.485235	4.568854	-2.497382
H	-2.015768	2.866930	-2.531927
H	-0.688836	3.423552	-3.586770
C	-0.323756	1.036310	3.135583
H	-0.053418	0.138096	2.558448
C	-1.386413	0.597991	4.150819
H	-2.314064	0.274379	3.653382
H	-1.641235	1.402958	4.857632
H	-1.010344	-0.247424	4.746980
C	0.950572	1.535486	3.844685
H	1.732776	1.815377	3.123638
H	1.356712	0.755077	4.507475
H	0.729349	2.423122	4.458535
C	4.008442	0.449609	-0.036226
C	4.594993	0.891426	-1.237683
C	5.993695	0.836409	-1.307993
H	6.498873	1.159229	-2.220184
C	6.750604	0.377413	-0.228460
H	7.839726	0.342505	-0.307677
C	6.130184	-0.026356	0.954344
H	6.739564	-0.367834	1.793464
C	4.734635	0.002252	1.083000
C	3.769873	1.373815	-2.425539
H	2.729752	1.505205	-2.090500
C	4.246250	2.743051	-2.935480
H	3.579372	3.101721	-3.734848
H	5.262112	2.690942	-3.357128
H	4.249234	3.492341	-2.129828
C	3.762403	0.325695	-3.552864
H	3.359735	-0.639359	-3.208409
H	4.781119	0.148641	-3.933522
H	3.143204	0.670666	-4.395836
C	4.063061	-0.441079	2.377203
H	2.977149	-0.294611	2.272520
C	4.296893	-1.937619	2.646549
H	3.937347	-2.558564	1.811469
H	3.765535	-2.250710	3.558954
H	5.366919	-2.155179	2.792363
C	4.515416	0.426178	3.563776
H	4.328515	1.493976	3.373282
H	5.590419	0.301594	3.768110
H	3.968592	0.139853	4.475569
C	3.282844	-2.463219	-1.008072
I	-3.559100	-2.664232	-0.001355
C	-3.387191	-0.189788	-0.444044

C	-3.600427	0.137513	-1.783138
C	-3.793314	0.630837	0.606671
H	-3.324651	-0.545337	-2.587187
C	-4.181995	1.377315	-2.066075
C	-4.368786	1.862207	0.286643
H	-3.652376	0.335120	1.645668
H	-4.344720	1.651127	-3.112083
H	-4.665484	2.525562	1.101931
C	-4.566699	2.261319	-1.045196
C	-5.139698	3.615328	-1.371006
H	-5.723193	3.597771	-2.303470
H	-5.787446	3.989214	-0.564599
H	-4.325960	4.349327	-1.507695
H	4.153268	-1.811492	-0.998163

89

TS-AB-trans+12-CH SCF Done: -1840.05818579 A.U.

Au	-1.553588	0.300561	-0.000048
N	-0.026354	2.589004	-0.000216
N	1.126541	-1.334562	0.000248
N	2.442281	-1.221539	0.000264
N	2.662580	0.075676	0.000105
C	-0.386021	3.867685	-0.000368
H	-1.462878	4.067445	-0.000397
C	0.549354	4.910767	-0.000488
H	0.217676	5.950640	-0.000610
C	1.903929	4.578123	-0.000449
H	2.668056	5.358701	-0.000541
C	1.279819	2.253944	-0.000178
C	1.493334	0.796160	-0.000011
C	0.465453	-0.143296	0.000073
C	0.505377	-2.639297	0.000322
C	0.195332	-3.217261	1.244145
C	-0.431223	-4.470198	1.213740
H	-0.689517	-4.968568	2.148945
C	-0.734378	-5.089549	0.000418
H	-1.221894	-6.067401	0.000460
C	-0.430721	-4.470551	-1.212953
H	-0.688622	-4.969186	-2.148127
C	0.195827	-3.217609	-1.243455
C	0.560159	-2.529679	2.554189
H	0.592756	-1.444477	2.362057
C	1.966160	-2.961017	3.012834
H	2.254970	-2.429023	3.933171
H	2.722725	-2.751318	2.241797
H	1.989972	-4.042253	3.222764
C	-0.482089	-2.746071	3.659331
H	-0.503947	-3.791195	4.005638
H	-1.493693	-2.479596	3.315237

H	-0.238855	-2.122766	4.533885
C	0.561112	-2.530392	-2.553561
H	0.594088	-1.445168	-2.361633
C	-0.481080	-2.746598	-3.658798
H	-1.492619	-2.479643	-3.314884
H	-0.503320	-3.791788	-4.004884
H	-0.237485	-2.123577	-4.533454
C	1.967021	-2.962330	-3.011911
H	2.723542	-2.752673	-2.240817
H	2.256135	-2.430673	-3.932346
H	1.990502	-4.043629	-3.221555
C	4.024135	0.554177	0.000046
C	4.638648	0.788465	1.244397
C	5.935343	1.319796	1.212669
H	6.455021	1.524090	2.150864
C	6.572256	1.587917	-0.000098
H	7.582530	2.003933	-0.000156
C	5.935391	1.319339	-1.212790
H	6.455105	1.523292	-2.151039
C	4.638700	0.787991	-1.244372
C	3.942831	0.504310	2.570423
H	2.981703	0.011596	2.357842
C	4.757801	-0.469970	3.436470
H	4.203529	-0.716643	4.355329
H	5.722573	-0.033759	3.739442
H	4.962532	-1.407844	2.898504
C	3.631872	1.808814	3.324966
H	3.008137	2.486332	2.720993
H	4.556712	2.347003	3.587304
H	3.093216	1.592789	4.260995
C	3.942899	0.503406	-2.570317
H	2.981945	0.010413	-2.357601
C	3.631481	1.807717	-3.325009
H	3.007514	2.485089	-2.721114
H	3.092901	1.591402	-4.261015
H	4.556142	2.346191	-3.587394
C	4.758100	-0.470731	-3.436301
H	4.963183	-1.408465	-2.898225
H	5.722699	-0.034262	-3.739451
H	4.203811	-0.717710	-4.355069
C	2.284902	3.232556	-0.000294
I	-3.677244	-1.478923	-0.000318
C	-3.511691	1.096623	-0.000128
C	-3.889638	1.678128	-1.223359
C	-3.889810	1.677944	1.223144
H	-3.655936	1.186171	-2.169437
C	-4.554008	2.904491	-1.209235
C	-4.554177	2.904302	1.209114
H	-3.656238	1.185841	2.169179

H	-4.823121	3.372946	-2.159873
H	-4.823425	3.372615	2.159785
C	-4.901828	3.535487	-0.000039
C	-5.678225	4.825709	0.000041
H	-6.762864	4.619887	0.000736
H	-5.461898	5.430751	0.893075
H	-5.462918	5.430285	-0.893549
H	3.337509	2.956905	-0.000265

89
B-cis+12-CH SCF Done: -1840.09506737 A.U.

Au	1.297800	-1.234082	-0.098577
N	-0.519361	-2.640661	-0.126554
N	-0.582907	1.399693	0.097695
N	-1.884137	1.645045	0.150185
N	-2.464534	0.468979	0.093178
C	-0.486276	-3.973763	-0.193399
H	0.506597	-4.428192	-0.247873
C	-1.654285	-4.742833	-0.193924
H	-1.585243	-5.830292	-0.249988
C	-2.884154	-4.091158	-0.122492
H	-3.816356	-4.660338	-0.120916
C	-1.712840	-1.992617	-0.056912
C	-1.546286	-0.540089	0.011417
C	-0.295386	0.070059	0.012523
C	0.322507	2.534088	0.108334
C	0.958485	2.865229	1.321150
C	1.811535	3.974878	1.292082
H	2.341315	4.272221	2.196767
C	1.991909	4.714063	0.121797
H	2.656639	5.581142	0.129034
C	1.338024	4.355898	-1.055065
H	1.497936	4.944948	-1.959088
C	0.488340	3.242315	-1.094862
C	0.676780	2.094469	2.607022
H	0.595675	1.027239	2.338287
C	-0.669211	2.538707	3.216529
H	-0.915460	1.925076	4.097494
H	-1.500600	2.463616	2.502116
H	-0.611489	3.589617	3.541240
C	1.790279	2.207169	3.655033
H	1.842408	3.220362	4.083731
H	2.773408	1.961972	3.231187
H	1.590807	1.515500	4.487785
C	-0.237911	2.850804	-2.376714
H	-0.520264	1.787194	-2.293498
C	0.642086	2.975985	-3.629593
H	1.598173	2.443754	-3.514230
H	0.867997	4.026974	-3.866920

H	0.118672	2.554567	-4.501657
C	-1.532802	3.670495	-2.533495
H	-2.202305	3.537389	-1.671331
H	-2.073991	3.370044	-3.443924
H	-1.299208	4.743740	-2.616636
C	-3.906474	0.373091	0.128254
C	-4.519655	0.190568	1.382010
C	-5.912107	0.028150	1.379021
H	-6.437563	-0.114017	2.325420
C	-6.634823	0.050074	0.185682
H	-7.719089	-0.082236	0.207414
C	-5.988116	0.249867	-1.035512
H	-6.574248	0.273782	-1.955719
C	-4.598947	0.424294	-1.097518
C	-3.736307	0.168816	2.688836
H	-2.669681	0.311799	2.457874
C	-4.153701	1.335054	3.600567
H	-3.534307	1.348643	4.510629
H	-5.205727	1.243314	3.913165
H	-4.035040	2.303312	3.091013
C	-3.866527	-1.186535	3.404563
H	-3.533956	-2.017081	2.761980
H	-4.907887	-1.386899	3.701937
H	-3.252532	-1.196926	4.318536
C	-3.888760	0.617573	-2.432892
H	-2.869026	0.977921	-2.228888
C	-3.766108	-0.719545	-3.186481
H	-3.220149	-1.472519	-2.597090
H	-3.228760	-0.578870	-4.137352
H	-4.760971	-1.132336	-3.418263
C	-4.567252	1.687536	-3.301880
H	-4.662899	2.641811	-2.762818
H	-5.571778	1.375234	-3.626760
H	-3.973319	1.865574	-4.211487
C	2.777985	0.175214	-0.047917
C	3.548701	0.333723	1.101232
H	3.416981	-0.317415	1.966708
C	4.520417	1.341605	1.129488
H	5.126560	1.464804	2.031294
C	3.958637	1.974823	-1.121090
H	4.109090	2.610435	-1.998004
C	2.984013	0.975406	-1.170289
H	2.393528	0.838590	-2.077526
I	3.168230	-3.126469	-0.281410
C	-2.920760	-2.694599	-0.052772
C	4.734625	2.184000	0.029725
C	5.740006	3.305610	0.081741
H	6.450078	3.181480	0.912192
H	6.315658	3.376028	-0.854106

H	5.228941	4.274435	0.221784
H	-3.868327	-2.161946	0.002082

89

TS-decomp-cis+12-CH SCF Done: -1840.05851303 A.U.

Au	-1.871424	-0.521801	-0.421900
N	0.067699	-2.053656	-1.929625
N	0.494717	0.852773	1.034315
N	1.767121	0.642322	1.299525
N	2.182861	-0.193158	0.377906
C	-0.007721	-2.991768	-2.867653
H	-1.013121	-3.342297	-3.123234
C	1.127788	-3.518578	-3.498688
H	1.026528	-4.295207	-4.259114
C	2.376849	-3.020302	-3.129306
H	3.288011	-3.394207	-3.601489
C	1.268242	-1.578502	-1.555810
C	1.185372	-0.564355	-0.487745
C	0.051728	0.141198	-0.055183
C	-0.258490	1.760946	1.873245
C	-0.059470	3.145772	1.691075
C	-0.853036	3.996495	2.471757
H	-0.747168	5.076981	2.369275
C	-1.779539	3.482504	3.379872
H	-2.394581	4.166511	3.969417
C	-1.913697	2.105415	3.560978
H	-2.620065	1.729373	4.301192
C	-1.140995	1.200885	2.819418
C	1.006364	3.700285	0.751136
H	1.156629	2.964901	-0.057997
C	2.339676	3.866676	1.510318
H	3.127450	4.227014	0.830088
H	2.675987	2.924314	1.963588
H	2.226125	4.607473	2.317442
C	0.616614	5.026644	0.083996
H	0.594801	5.856015	0.807746
H	-0.365930	4.967065	-0.402223
H	1.361163	5.289799	-0.683063
C	-1.159853	-0.294323	3.122343
H	-0.946329	-0.840618	2.190553
C	-2.512439	-0.807930	3.626265
H	-3.329706	-0.531182	2.944280
H	-2.747281	-0.425915	4.632029
H	-2.492165	-1.905993	3.692036
C	-0.025433	-0.630198	4.112173
H	0.957577	-0.314406	3.729992
H	0.011021	-1.714940	4.299340
H	-0.188780	-0.123007	5.076250
C	3.554146	-0.648056	0.420041

C	4.537287	0.172962	-0.170925
C	5.842527	-0.334158	-0.178441
H	6.647936	0.252505	-0.621366
C	6.133814	-1.581088	0.382882
H	7.160483	-1.954132	0.362044
C	5.134560	-2.344318	0.984640
H	5.387593	-3.304467	1.438520
C	3.807295	-1.891894	1.023481
C	4.189442	1.525314	-0.789159
H	3.341705	1.935098	-0.221736
C	5.326137	2.548916	-0.670422
H	4.969601	3.540790	-0.988071
H	6.181408	2.293022	-1.315338
H	5.689252	2.631965	0.365062
C	3.734917	1.379303	-2.254093
H	2.837632	0.750708	-2.352774
H	4.531203	0.931211	-2.869748
H	3.493151	2.366437	-2.678351
C	2.722191	-2.716520	1.706161
H	1.763617	-2.183380	1.609178
C	2.544508	-4.090946	1.039159
H	2.316353	-3.992873	-0.033087
H	1.718459	-4.641474	1.515346
H	3.453382	-4.705379	1.136832
C	3.004568	-2.848884	3.212966
H	3.104294	-1.862132	3.690204
H	3.933342	-3.410768	3.399077
H	2.182385	-3.388114	3.708646
C	-1.202988	1.249714	-1.373952
C	-0.647468	1.190674	-2.653788
H	-0.135839	0.295213	-3.004396
C	-0.783758	2.299609	-3.492980
H	-0.355878	2.255571	-4.497812
C	-2.009760	3.472172	-1.780314
H	-2.563753	4.350556	-1.438442
C	-1.864377	2.392082	-0.908210
H	-2.290738	2.436595	0.093413
I	-3.908537	-2.104782	-0.041539
C	2.460031	-2.031759	-2.144500
C	-1.466645	3.454394	-3.076909
C	-1.612418	4.652379	-3.977645
H	-1.069776	5.519299	-3.564514
H	-1.222252	4.458037	-4.986527
H	-2.669333	4.949409	-4.069945
H	3.427969	-1.632197	-1.852580

104

TS-CD-cis+12-CH SCF Done: -3233.63484354 A.U.

Au	-1.150379	-0.603402	-1.292586

N	0.749184	-1.710063	-1.796770
N	0.388766	1.552083	0.543523
N	1.642344	1.767763	0.916365
N	2.354468	0.859090	0.284519
C	0.847498	-2.837271	-2.501484
H	-0.072411	-3.272538	-2.882786
C	2.089726	-3.427460	-2.792953
H	2.114412	-4.352206	-3.369118
C	3.241596	-2.833413	-2.305149
H	4.222476	-3.267244	-2.510094
C	1.879435	-1.134314	-1.284273
C	1.568809	0.052239	-0.489095
C	0.267513	0.519281	-0.334435
C	-0.643610	2.400690	1.111153
C	-0.852077	3.654241	0.510578
C	-1.814104	4.482860	1.104201
H	-2.015931	5.466438	0.678149
C	-2.514681	4.066236	2.234680
H	-3.255626	4.730064	2.686683
C	-2.289576	2.805456	2.789350
H	-2.865631	2.496241	3.661309
C	-1.349214	1.927816	2.236277
C	-0.042499	4.115372	-0.695726
H	0.328052	3.214997	-1.214940
C	1.181506	4.933296	-0.240878
H	1.790256	5.234587	-1.107653
H	1.818882	4.360400	0.448430
H	0.858873	5.847998	0.281324
C	-0.878090	4.900152	-1.717003
H	-1.182564	5.883559	-1.326256
H	-1.786891	4.349783	-1.999478
H	-0.285421	5.085045	-2.626402
C	-1.107062	0.537314	2.817337
H	-0.954893	-0.152130	1.969909
C	-2.296237	-0.019321	3.611747
H	-3.240701	0.081059	3.057533
H	-2.417018	0.494295	4.578939
H	-2.142984	-1.090397	3.807313
C	0.160090	0.513179	3.694457
H	1.045608	0.896243	3.169780
H	0.371530	-0.514238	4.034178
H	0.019993	1.136273	4.591951
C	3.790997	0.833900	0.432535
C	4.550700	1.626371	-0.450641
C	5.944150	1.529738	-0.335890
H	6.579983	2.117694	-0.999794
C	6.531990	0.694711	0.615670
H	7.620673	0.637846	0.686624
C	5.742581	-0.061595	1.482602

H	6.221749	-0.697303	2.229864
C	4.343657	-0.012241	1.411670
C	3.907432	2.510177	-1.513704
H	2.846152	2.641064	-1.254130
C	4.523936	3.916343	-1.556567
H	3.968292	4.548364	-2.266205
H	5.572016	3.895642	-1.893284
H	4.490481	4.400269	-0.568950
C	3.966414	1.828625	-2.893382
H	3.468170	0.846769	-2.885194
H	5.010234	1.672084	-3.209368
H	3.473101	2.454267	-3.653681
C	3.489848	-0.832098	2.371728
H	2.431099	-0.666029	2.118199
C	3.766165	-2.339614	2.233925
H	3.556354	-2.703568	1.215091
H	3.152629	-2.909290	2.952808
H	4.814645	-2.583576	2.462956
C	3.682812	-0.352696	3.820564
H	3.469365	0.722313	3.915753
H	4.715227	-0.524814	4.162897
H	3.007732	-0.895376	4.500595
C	-2.711502	0.659274	-0.939764
C	-2.961820	1.696132	-1.840941
H	-2.344684	1.834755	-2.731575
C	-4.021756	2.576052	-1.591766
H	-4.216137	3.386681	-2.299789
C	-4.560454	1.382221	0.424795
H	-5.173517	1.253930	1.321551
C	-3.510681	0.489659	0.188857
H	-3.300584	-0.319397	0.886931
C	3.140614	-1.669300	-1.523064
O	-1.781165	-2.232240	0.830005
O	-2.142688	-3.332817	2.736647
C	-2.465497	-3.013231	1.582834
C	-3.698197	-3.641190	0.925314
H	-4.565421	-3.555481	1.596026
H	-3.478502	-4.717761	0.833603
C	-3.953712	-3.017954	-0.442971
C	-2.716168	-2.895308	-1.250677
H	-1.952614	-3.683793	-1.134460
C	-2.559273	-1.963331	-2.302871
H	-1.946748	-2.295189	-3.150743
H	-3.441207	-1.378008	-2.584916
H	-4.623112	-3.663872	-1.049398
H	-4.462252	-2.042199	-0.380237
K	0.356971	-3.618268	1.886575
Cl	0.077582	-4.919010	-0.668031
C	-4.828918	2.444856	-0.451439

C	-5.917389	3.441925	-0.146036
H	-6.276294	3.948007	-1.054423
H	-6.779010	2.965392	0.345715
H	-5.540934	4.221534	0.539620
H	4.029511	-1.188444	-1.118983

104
TS-CD-trans+12-CH SCF Done: -3233.63152388 A.U.

Au	-1.570082	-0.364574	-0.471039
N	-0.663410	-2.084984	0.479268
N	1.350793	1.212846	-0.642198
N	2.618086	0.837640	-0.508242
N	2.560106	-0.417176	-0.120470
C	-1.349537	-3.108785	1.006667
H	-2.436419	-3.050761	0.945420
C	-0.702070	-4.194554	1.591071
H	-1.294047	-5.007101	2.014374
C	0.694177	-4.216003	1.611228
H	1.230361	-5.056713	2.056410
C	0.697514	-2.070601	0.528234
C	1.268440	-0.835691	0.008417
C	0.458084	0.227611	-0.365852
C	1.048839	2.582183	-0.992782
C	0.929473	2.904898	-2.358629
C	0.572948	4.226412	-2.662556
H	0.462663	4.533158	-3.703306
C	0.368874	5.162748	-1.646915
H	0.093938	6.187960	-1.906601
C	0.523142	4.809027	-0.305675
H	0.368747	5.561365	0.468260
C	0.870169	3.500338	0.062953
C	1.249160	1.884422	-3.447546
H	0.988542	0.884870	-3.057360
C	2.763282	1.880910	-3.741225
H	3.007787	1.114691	-4.493215
H	3.350958	1.675345	-2.835362
H	3.078414	2.860067	-4.135146
C	0.451498	2.089130	-4.742684
H	0.761840	3.003439	-5.271659
H	-0.632442	2.161147	-4.560748
H	0.626387	1.246607	-5.429143
C	1.106388	3.099873	1.513457
H	0.816909	2.044030	1.638491
C	0.287484	3.909433	2.525512
H	-0.793317	3.874963	2.329638
H	0.601332	4.965027	2.563365
H	0.454857	3.496898	3.532941
C	2.608114	3.195081	1.850687
H	3.225610	2.620726	1.147182

H	2.784883	2.793889	2.859988
H	2.949886	4.242640	1.823940
C	3.768082	-1.211813	-0.055995
C	4.085373	-1.970286	-1.203209
C	5.208188	-2.802456	-1.115804
H	5.491417	-3.416277	-1.972949
C	5.976950	-2.849507	0.049022
H	6.850358	-3.504508	0.094501
C	5.648888	-2.056333	1.147543
H	6.273288	-2.088476	2.042292
C	4.529101	-1.210008	1.126823
C	3.261535	-1.910671	-2.486621
H	2.559615	-1.066347	-2.408499
C	4.139272	-1.633523	-3.717878
H	3.508188	-1.497646	-4.610379
H	4.825676	-2.468492	-3.927206
H	4.742421	-0.723494	-3.581932
C	2.424757	-3.188904	-2.671678
H	1.751312	-3.360521	-1.817733
H	3.074076	-4.073762	-2.766933
H	1.810946	-3.121984	-3.584175
C	4.200490	-0.325002	2.318606
H	3.226500	0.151982	2.144459
C	4.047446	-1.133904	3.614884
H	3.260317	-1.894620	3.512635
H	3.733042	-0.465159	4.429502
H	4.989623	-1.622069	3.913292
C	5.258602	0.782910	2.461419
H	5.345613	1.379409	1.540130
H	6.252635	0.363023	2.686267
H	4.986328	1.462432	3.283671
C	-3.386038	-1.362580	-0.626171
C	-4.385204	-1.197198	0.338789
H	-4.253983	-0.456535	1.130623
C	-5.543093	-1.981876	0.270718
H	-6.318442	-1.850483	1.031498
C	-4.720171	-3.066326	-1.713597
H	-4.838415	-3.795525	-2.520322
C	-3.554894	-2.291172	-1.659178
H	-2.781864	-2.437314	-2.419375
C	1.403353	-3.144437	1.073746
O	-2.728373	1.153362	1.445427
O	-2.987842	2.803645	2.935222
C	-3.001439	2.356861	1.777004
C	-3.400638	3.306141	0.638057
H	-3.181311	4.339773	0.937967
H	-4.497393	3.236535	0.527033
C	-2.714226	2.965480	-0.678946
C	-3.026014	1.630072	-1.235644

H	-4.022281	1.221831	-1.032801
C	-2.216742	0.952091	-2.145560
H	-2.674318	0.209889	-2.806700
H	-1.312980	1.447682	-2.505021
H	-3.071762	3.658559	-1.471166
H	-1.626518	3.118925	-0.631894
K	-1.727087	0.781657	4.022231
Cl	0.742936	-0.278733	3.024797
C	-5.733264	-2.926032	-0.751968
C	-7.002287	-3.737423	-0.832790
H	-7.407188	-3.956211	0.166886
H	-7.782224	-3.186268	-1.386138
H	-6.842684	-4.691669	-1.356647
H	2.490933	-3.114133	1.100524

89

A-ad+13-CH SCF Done: -2137.58278291 A.U.

Au	-0.544918	1.616755	0.002098
N	2.415675	2.817262	-0.420689
N	0.893367	-1.002308	0.436042
N	2.037182	-1.654580	0.379779
N	2.899957	-0.775557	-0.075998
C	2.961000	3.970340	-0.800414
H	2.446508	4.881665	-0.477165
C	4.130611	4.049844	-1.567541
H	4.543039	5.021232	-1.847224
C	4.739196	2.859327	-1.963618
H	5.645367	2.870011	-2.573508
C	3.008477	1.668740	-0.788354
C	2.323555	0.451135	-0.310742
C	0.978518	0.303479	0.033396
C	-0.274362	-1.701723	0.925208
C	-0.510838	-1.681792	2.313146
C	-1.614179	-2.413173	2.773846
H	-1.832136	-2.444579	3.842574
C	-2.430752	-3.114672	1.884239
H	-3.282730	-3.685296	2.262801
C	-2.180189	-3.084379	0.512767
H	-2.849790	-3.611268	-0.168134
C	-1.088409	-2.371919	-0.003781
C	0.420980	-0.951030	3.272113
H	0.951923	-0.174887	2.697926
C	1.479628	-1.919488	3.832344
H	2.187085	-1.384809	4.485338
H	2.051693	-2.397596	3.023013
H	1.002577	-2.716147	4.425317
C	-0.329000	-0.224605	4.397082
H	-0.813990	-0.927772	5.092060
H	-1.103763	0.447666	3.995149

H	0.373991	0.381356	4.989286
C	-0.845743	-2.306270	-1.505250
H	0.116551	-1.800050	-1.677970
C	-1.939316	-1.460421	-2.182369
H	-1.995703	-0.453285	-1.739730
H	-2.926702	-1.933123	-2.069328
H	-1.734709	-1.353262	-3.259458
C	-0.741684	-3.703598	-2.136152
H	0.036743	-4.307265	-1.646116
H	-0.489933	-3.620688	-3.205221
H	-1.693741	-4.252255	-2.064282
C	4.280253	-1.180373	-0.218281
C	5.172904	-0.854552	0.817196
C	6.510428	-1.234880	0.632395
H	7.246137	-1.000687	1.404265
C	6.912095	-1.912105	-0.518255
H	7.958801	-2.201475	-0.638127
C	5.989383	-2.229227	-1.518236
H	6.324704	-2.766432	-2.406575
C	4.642847	-1.865317	-1.394955
C	4.739556	-0.119928	2.080156
H	3.643279	-0.018552	2.062495
C	5.090141	-0.919275	3.346088
H	4.696681	-0.408048	4.238667
H	6.179311	-1.016823	3.475939
H	4.659846	-1.931436	3.313313
C	5.331667	1.299847	2.126957
H	5.048737	1.885694	1.239461
H	6.431976	1.267748	2.172650
H	4.973888	1.836119	3.019833
C	3.614190	-2.220720	-2.462354
H	2.763471	-1.526798	-2.354149
C	4.146413	-2.048575	-3.891978
H	4.563219	-1.041847	-4.053098
H	3.332607	-2.200779	-4.617572
H	4.931590	-2.783079	-4.129340
C	3.075679	-3.645420	-2.231656
H	2.638337	-3.749124	-1.227709
H	3.886229	-4.385304	-2.328253
H	2.299942	-3.889541	-2.974134
C	4.172491	1.643744	-1.574183
I	-2.795937	3.180514	-0.063829
C	-3.977769	1.366078	-0.174814
C	-3.957160	0.504087	0.919268
C	-4.657771	1.072300	-1.355293
H	-3.415511	0.749726	1.833422
C	-4.623935	-0.717594	0.809375
C	-5.317059	-0.156635	-1.447927
H	-4.665008	1.763814	-2.199117

H	-4.610260	-1.415130	1.645414
C	-5.281624	-1.053788	-0.375876
H	-5.847131	-0.419224	-2.365646
C	-5.874536	-2.432899	-0.546272
F	-5.027966	-3.219389	-1.257657
F	-7.031928	-2.401017	-1.213974
F	-6.078373	-3.042279	0.629712
H	4.627081	0.703720	-1.880170

89
TS-AB-cis+13-CH SCF Done: -2137.55856323 A.U.

Au	0.745786	1.559374	-0.064993
N	-1.707496	2.713145	-0.645020
N	-0.766468	-1.155010	0.399578
N	-1.965543	-1.706528	0.388232
N	-2.774355	-0.744138	0.005827
C	-2.047206	3.955524	-0.969856
H	-1.237845	4.693463	-0.984543
C	-3.364330	4.321470	-1.276924
H	-3.604279	5.353818	-1.537793
C	-4.346043	3.332292	-1.236459
H	-5.386533	3.570671	-1.468253
C	-2.647175	1.748574	-0.604992
C	-2.103695	0.432056	-0.223271
C	-0.759153	0.159947	0.034914
C	0.366581	-1.952977	0.811905
C	1.011414	-2.737700	-0.163952
C	2.112435	-3.490019	0.263786
H	2.661295	-4.102168	-0.451302
C	2.530671	-3.456236	1.595552
H	3.400509	-4.042726	1.899071
C	1.857177	-2.675244	2.533455
H	2.195097	-2.668584	3.571728
C	0.746202	-1.902129	2.164406
C	0.486815	-2.819930	-1.592721
H	-0.028483	-1.869473	-1.813737
C	-0.549836	-3.954915	-1.711346
H	-0.966783	-3.991216	-2.729859
H	-1.380391	-3.824157	-1.002766
H	-0.077140	-4.927799	-1.502620
C	1.593107	-2.983038	-2.642209
H	2.086563	-3.964345	-2.570870
H	2.366908	-2.211444	-2.534667
H	1.164832	-2.908090	-3.653599
C	-0.003209	-1.069013	3.198896
H	-0.920888	-0.681505	2.730521
C	0.823902	0.147918	3.646634
H	1.045950	0.811785	2.796847
H	1.779173	-0.164869	4.098292

H	0.273572	0.735540	4.398081
C	-0.445725	-1.920483	4.400213
H	-1.039429	-2.789123	4.077821
H	-1.062540	-1.318769	5.085945
H	0.415898	-2.293193	4.975610
C	-4.185342	-1.034100	-0.110399
C	-4.657791	-1.526463	-1.339587
C	-6.039444	-1.750914	-1.432515
H	-6.461185	-2.125733	-2.366944
C	-6.880754	-1.503871	-0.348091
H	-7.953570	-1.687248	-0.443644
C	-6.367157	-1.030101	0.862038
H	-7.044350	-0.854646	1.698764
C	-4.997610	-0.780490	1.012919
C	-3.741867	-1.773948	-2.532534
H	-2.699176	-1.700493	-2.187276
C	-3.918298	-3.188248	-3.107154
H	-3.186955	-3.363593	-3.911322
H	-4.920596	-3.330873	-3.540193
H	-3.767969	-3.956703	-2.334216
C	-3.946787	-0.696606	-3.612793
H	-3.764079	0.314743	-3.217280
H	-4.975626	-0.722833	-4.006154
H	-3.259254	-0.861482	-4.457228
C	-4.402090	-0.306777	2.333753
H	-3.480981	0.255021	2.104557
C	-5.319955	0.648950	3.106926
H	-5.646632	1.493552	2.480480
H	-4.787921	1.056240	3.980266
H	-6.218123	0.137910	3.487351
C	-3.998345	-1.517722	3.197637
H	-3.295103	-2.176317	2.666649
H	-4.885071	-2.114678	3.463742
H	-3.519378	-1.183286	4.131098
C	-3.991961	2.022812	-0.896605
I	2.613906	3.496163	-0.012569
C	2.998085	1.056876	-0.205048
C	3.358697	0.649006	-1.493527
C	3.477273	0.430061	0.945561
H	3.014422	1.188119	-2.376338
C	4.166069	-0.481724	-1.620708
C	4.277358	-0.703349	0.792302
H	3.216824	0.791447	1.939269
H	4.447843	-0.829897	-2.616766
H	4.629836	-1.234854	1.676065
C	4.607432	-1.168001	-0.483167
C	5.437229	-2.416941	-0.662525
F	4.829770	-3.274204	-1.512870
F	5.621494	-3.068183	0.497873

F 6.642273 -2.143173 -1.178128
H -4.747357 1.241038 -0.863484

89
TS-AB-trans+13-CH SCF Done: -2137.55267173 A.U.

Au	-1.222696	0.506851	-0.041693
N	-0.646283	-2.105625	-0.054083
N	1.879659	1.102492	0.015392
N	3.059488	0.509870	0.060805
N	2.785719	-0.777017	0.042591
C	-1.461465	-3.156201	-0.072406
H	-2.534831	-2.946052	-0.092035
C	-0.984664	-4.473195	-0.066219
H	-1.685779	-5.309491	-0.081655
C	0.395387	-4.673483	-0.039908
H	0.810266	-5.683920	-0.034136
C	0.691358	-2.285166	-0.029141
C	1.434662	-1.013438	-0.010081
C	0.828123	0.239234	-0.028763
C	1.786798	2.544567	0.004935
C	1.904138	3.198140	-1.234164
C	1.791240	4.595236	-1.218866
H	1.882479	5.154965	-2.150797
C	1.558628	5.280940	-0.026427
H	1.474605	6.370265	-0.037417
C	1.423471	4.591139	1.179908
H	1.231290	5.149738	2.096622
C	1.530660	3.195212	1.226288
C	2.175659	2.429605	-2.521715
H	1.828172	1.393545	-2.372334
C	3.688365	2.374208	-2.805905
H	3.891270	1.785525	-3.714317
H	4.236656	1.916991	-1.968667
H	4.090410	3.388178	-2.960538
C	1.398297	2.982700	-3.724327
H	1.758398	3.979375	-4.023455
H	0.321622	3.061607	-3.506635
H	1.527173	2.319731	-4.594028
C	1.424015	2.419206	2.533971
H	1.030152	1.418052	2.294481
C	0.441382	3.044146	3.532296
H	-0.543563	3.216708	3.070710
H	0.808152	4.004943	3.925910
H	0.305738	2.374453	4.395757
C	2.819515	2.229664	3.159300
H	3.511918	1.735331	2.461266
H	2.756272	1.617307	4.072968
H	3.257142	3.203314	3.431440
C	3.871059	-1.727618	0.089614

C	4.419845	-2.161879	-1.131690
C	5.423735	-3.137065	-1.054899
H	5.879330	-3.512353	-1.973303
C	5.852002	-3.632105	0.178079
H	6.635715	-4.392549	0.212737
C	5.296651	-3.157325	1.367290
H	5.654781	-3.546166	2.322625
C	4.287518	-2.184606	1.353886
C	3.945825	-1.630964	-2.479605
H	3.253866	-0.794645	-2.296444
C	5.110878	-1.067345	-3.308929
H	4.731831	-0.623328	-4.242509
H	5.829922	-1.853616	-3.587030
H	5.655351	-0.287636	-2.755573
C	3.168842	-2.709061	-3.255779
H	2.302624	-3.075696	-2.682926
H	3.811639	-3.574340	-3.483667
H	2.799195	-2.304813	-4.211225
C	3.682633	-1.669475	2.654444
H	2.946957	-0.888098	2.409391
C	2.930942	-2.784170	3.402202
H	2.140343	-3.227667	2.776541
H	2.460829	-2.385006	4.314651
H	3.613713	-3.593561	3.705812
C	4.750477	-1.009512	3.542516
H	5.269729	-0.199775	3.007755
H	5.507554	-1.738022	3.872849
H	4.285222	-0.581197	4.443883
C	1.254042	-3.569334	-0.021006
I	-2.711457	2.830112	0.030672
C	-3.334416	0.339807	-0.045208
C	-3.830779	-0.171477	1.166334
C	-3.826830	-0.102154	-1.285069
H	-3.496140	0.234586	2.122378
C	-4.731473	-1.236049	1.126056
C	-4.727227	-1.168138	-1.307653
H	-3.490079	0.357401	-2.215880
H	-5.101266	-1.675176	2.054676
H	-5.093488	-1.554648	-2.260701
C	-5.158718	-1.744902	-0.106903
C	-5.996349	-3.001433	-0.142035
F	-5.181349	-4.083687	-0.157486
F	-6.762935	-3.065830	-1.236185
F	-6.782787	-3.112465	0.934367
H	2.333239	-3.707901	-0.000817

89

B-cis+13-CH SCF Done: -2137.59446616 A.U.

Au	-0.632908	1.613282	-0.088624

N	1.441614	2.570910	-0.054072
N	0.603422	-1.384003	0.022599
N	1.814728	-1.915848	0.085859
N	2.644017	-0.898282	0.101572
C	1.706217	3.879855	-0.083889
H	0.841871	4.544756	-0.158731
C	3.014063	4.370739	-0.022393
H	3.188298	5.447440	-0.050761
C	4.066293	3.462131	0.075173
H	5.099989	3.811053	0.126856
C	2.460348	1.673469	0.038393
C	1.976018	0.293080	0.052824
C	0.622195	-0.021559	0.002213
C	-0.536314	-2.277952	-0.063228
C	-1.265320	-2.541775	1.113904
C	-2.369321	-3.391615	0.981882
H	-2.995887	-3.608108	1.846145
C	-2.695647	-3.962616	-0.249728
H	-3.569798	-4.612355	-0.322285
C	-1.933637	-3.693264	-1.384240
H	-2.207718	-4.151164	-2.335628
C	-0.833855	-2.826277	-1.323129
C	-0.830053	-1.979243	2.463599
H	-0.485641	-0.943992	2.293613
C	0.355792	-2.790770	3.026521
H	0.721122	-2.340487	3.962963
H	1.197851	-2.851399	2.323254
H	0.038810	-3.822056	3.247801
C	-1.956111	-1.913788	3.502032
H	-2.263464	-2.919298	3.829212
H	-2.842397	-1.396330	3.111564
H	-1.608836	-1.375347	4.397026
C	0.009041	-2.530609	-2.558652
H	0.534091	-1.574520	-2.390094
C	-0.828180	-2.353316	-3.834098
H	-1.630970	-1.612415	-3.699895
H	-1.293122	-3.298705	-4.153117
H	-0.186796	-2.014551	-4.662237
C	1.076652	-3.624327	-2.752385
H	1.727318	-3.714510	-1.870347
H	1.706179	-3.401238	-3.627522
H	0.597645	-4.602013	-2.919317
C	4.069262	-1.126946	0.179017
C	4.658003	-1.147520	1.457625
C	6.051423	-1.294695	1.501132
H	6.558391	-1.318684	2.467768
C	6.797673	-1.415115	0.328156
H	7.883025	-1.525511	0.386587
C	6.170787	-1.406933	-0.919286

H	6.772514	-1.513681	-1.823512
C	4.780428	-1.268049	-1.028547
C	3.848552	-1.016626	2.742287
H	2.783348	-0.935454	2.476702
C	3.992070	-2.273050	3.617523
H	3.349622	-2.192662	4.507924
H	5.028664	-2.405115	3.965031
H	3.701129	-3.179976	3.066185
C	4.222168	0.259811	3.515553
H	4.081153	1.162396	2.899928
H	5.273262	0.237469	3.843827
H	3.594958	0.360390	4.414956
C	4.099168	-1.237557	-2.392180
H	3.014500	-1.341781	-2.236290
C	4.338784	0.110861	-3.095582
H	3.972218	0.956659	-2.493173
H	3.820818	0.136770	-4.067010
H	5.412637	0.272422	-3.281410
C	4.531694	-2.415233	-3.279427
H	4.361710	-3.379620	-2.778043
H	5.596959	-2.353386	-3.550493
H	3.956901	-2.410791	-4.218259
C	-2.400197	0.591082	-0.043757
C	-3.158461	0.587459	1.128463
H	-2.857733	1.171614	1.999233
C	-4.330171	-0.172414	1.175215
H	-4.935324	-0.185257	2.083843
C	-3.981432	-0.871804	-1.118239
H	-4.304485	-1.441071	-1.990937
C	-2.811206	-0.111076	-1.175744
H	-2.228820	-0.086885	-2.097235
I	-2.032847	3.877773	-0.262176
C	3.791335	2.090954	0.106596
C	-4.729906	-0.914937	0.060607
C	-5.922945	-1.832205	0.165662
F	-5.556107	-3.031000	0.683202
F	-6.477129	-2.077311	-1.029861
F	-6.868481	-1.334169	0.973332
H	4.594385	1.360039	0.181459

89

TS-decomp-cis+13-CH SCF Done: -2137.55219721 A.U.

Au	-1.147077	-1.564724	-0.416586
N	0.821097	-1.241399	-2.473616
N	0.462205	0.453123	1.325725
N	1.698460	0.884453	1.447461
N	2.260483	0.676965	0.280418
C	0.997786	-1.774120	-3.677920
H	0.220340	-2.463908	-4.022378

C	2.116459	-1.484798	-4.471618
H	2.231673	-1.948050	-5.453308
C	3.064574	-0.592815	-3.971796
H	3.947094	-0.330355	-4.559328
C	1.738056	-0.393651	-1.975301
C	1.410187	0.083397	-0.618055
C	0.200379	-0.067784	0.080144
C	-0.446551	0.592901	2.443520
C	-0.978649	1.874777	2.701226
C	-1.918190	1.959531	3.736999
H	-2.368906	2.922874	3.977776
C	-2.290332	0.828144	4.464553
H	-3.034192	0.918402	5.259676
C	-1.707131	-0.412981	4.204713
H	-1.989022	-1.274357	4.810518
C	-0.751361	-0.562937	3.190058
C	-0.518269	3.118591	1.946670
H	-0.191847	2.800229	0.941253
C	0.690069	3.750483	2.669764
H	1.055840	4.626723	2.112047
H	1.520181	3.040298	2.785052
H	0.393982	4.090155	3.674706
C	-1.615677	4.173289	1.747324
H	-1.899496	4.650503	2.697914
H	-2.519952	3.754978	1.288523
H	-1.249141	4.971915	1.084660
C	0.011423	-1.870604	3.002291
H	0.290246	-1.961533	1.941044
C	-0.806216	-3.120053	3.345048
H	-1.762784	-3.138804	2.802346
H	-1.011872	-3.192618	4.424355
H	-0.244874	-4.022087	3.059443
C	1.325278	-1.815161	3.808447
H	1.943360	-0.950128	3.521839
H	1.915774	-2.729843	3.642031
H	1.116348	-1.735350	4.886982
C	3.655266	1.020643	0.116043
C	3.968623	2.353245	-0.223006
C	5.323214	2.631587	-0.443986
H	5.630340	3.643322	-0.710962
C	6.293346	1.632918	-0.317845
H	7.343157	1.876671	-0.496985
C	5.939731	0.335881	0.051747
H	6.715797	-0.423081	0.168270
C	4.599712	-0.006730	0.284760
C	2.888586	3.422610	-0.367138
H	2.067004	3.151805	0.311499
C	3.365429	4.816178	0.063257
H	2.513116	5.512527	0.083681

H	4.104960	5.234077	-0.637538
H	3.815692	4.797090	1.067147
C	2.313867	3.456258	-1.796082
H	1.855837	2.498242	-2.083875
H	3.101678	3.686530	-2.530991
H	1.537311	4.232971	-1.876985
C	4.218127	-1.419123	0.712539
H	3.123710	-1.467852	0.825153
C	4.609495	-2.461981	-0.348044
H	4.155713	-2.234195	-1.324788
H	4.272330	-3.463823	-0.040111
H	5.701483	-2.506331	-0.484427
C	4.819561	-1.753730	2.088725
H	4.513868	-1.018659	2.848801
H	5.920223	-1.766193	2.052848
H	4.484315	-2.749103	2.419304
C	-1.686960	0.473480	-0.660287
C	-1.494809	1.124334	-1.882682
H	-0.706158	0.810640	-2.565169
C	-2.344072	2.179669	-2.221713
H	-2.210396	2.703979	-3.169288
C	-3.563195	1.881107	-0.142983
H	-4.384336	2.163231	0.518701
C	-2.703579	0.847164	0.229081
H	-2.850875	0.333188	1.177874
I	-1.941038	-4.040563	-0.535448
C	2.881676	-0.030284	-2.705061
C	-3.370257	2.559741	-1.350808
C	-4.241215	3.757006	-1.662457
F	-3.827341	4.826735	-0.952691
F	-5.519039	3.529859	-1.332113
F	-4.192297	4.087835	-2.958502
H	3.612626	0.669969	-2.309191

104

TS-CD-cis+13-CH SCF Done: -3531.13396374 A.U.

Au	0.535193	1.038418	-1.298171
N	-1.614013	1.423715	-1.812966
N	-0.179925	-1.501795	0.554838
N	-1.286075	-2.129427	0.926036
N	-2.262858	-1.524825	0.283954
C	-2.084168	2.446177	-2.528425
H	-1.364755	3.165903	-2.909307
C	-3.450559	2.574907	-2.833194
H	-3.783473	3.431469	-3.419072
C	-4.335553	1.627601	-2.346637
H	-5.403633	1.699942	-2.561801
C	-2.485568	0.499931	-1.302444
C	-1.796004	-0.505012	-0.496418

C	-0.415380	-0.498527	-0.334542
C	1.080495	-1.927288	1.137740
C	1.762244	-2.986638	0.511403
C	2.968315	-3.390102	1.099044
H	3.553981	-4.188617	0.643453
C	3.439925	-2.774639	2.257692
H	4.386268	-3.102325	2.692502
C	2.732942	-1.726279	2.846364
H	3.133512	-1.249103	3.740768
C	1.535576	-1.257547	2.290727
C	1.191727	-3.694982	-0.711986
H	0.558085	-2.970579	-1.252792
C	0.296751	-4.872761	-0.277805
H	-0.155472	-5.359667	-1.155923
H	-0.511761	-4.546874	0.392487
H	0.893218	-5.627199	0.259119
C	2.267046	-4.168015	-1.699266
H	2.863893	-4.996038	-1.286562
H	2.955214	-3.353962	-1.966784
H	1.794094	-4.540526	-2.621048
C	0.779987	-0.075308	2.891387
H	0.369076	0.511810	2.052251
C	1.672286	0.887140	3.687297
H	2.577694	1.164276	3.127429
H	1.991384	0.449239	4.646340
H	1.125646	1.817393	3.897086
C	-0.390989	-0.551697	3.772502
H	-1.065644	-1.237089	3.241768
H	-0.977979	0.310114	4.130357
H	-0.013284	-1.086815	4.658095
C	-3.621493	-1.996109	0.419779
C	-4.054381	-2.995828	-0.473670
C	-5.397137	-3.384794	-0.373668
H	-5.785647	-4.151522	-1.046080
C	-6.244915	-2.808443	0.573496
H	-7.287511	-3.129310	0.632776
C	-5.771363	-1.832354	1.450957
H	-6.447014	-1.404218	2.194137
C	-4.439959	-1.397848	1.395666
C	-3.138155	-3.601908	-1.531127
H	-2.100154	-3.334786	-1.281599
C	-3.203300	-5.136678	-1.546215
H	-2.464423	-5.537358	-2.257057
H	-4.191771	-5.502439	-1.864587
H	-2.985699	-5.557790	-0.553238
C	-3.446593	-3.011395	-2.919371
H	-3.340844	-1.915314	-2.928506
H	-4.476351	-3.251410	-3.228935
H	-2.761527	-3.426058	-3.675368

C	-3.930951	-0.337308	2.364740
H	-2.869911	-0.145652	2.139759
C	-4.687683	0.992241	2.196435
H	-4.583553	1.397421	1.176787
H	-4.318434	1.738376	2.920843
H	-5.763633	0.874613	2.396054
C	-3.996024	-0.842573	3.816034
H	-3.442798	-1.785964	3.934476
H	-5.036344	-1.017780	4.131580
H	-3.557306	-0.102184	4.502944
C	2.431112	0.383596	-0.946376
C	3.046294	-0.441066	-1.894007
H	2.541272	-0.709593	-2.824215
C	4.325758	-0.941497	-1.637313
H	4.817590	-1.588600	-2.366186
C	4.361572	0.223124	0.487606
H	4.873095	0.471269	1.419182
C	3.086997	0.735601	0.232446
H	2.592462	1.381466	0.956589
C	-3.851030	0.572413	-1.553330
O	0.555918	2.748410	0.841902
O	0.495388	3.921318	2.738770
C	0.932643	3.714519	1.597336
C	1.914748	4.703412	0.961429
H	2.757672	4.883831	1.644377
H	1.372975	5.659700	0.876028
C	2.370531	4.209036	-0.407434
C	1.246195	3.710770	-1.234725
H	0.265341	4.203977	-1.116496
C	1.405784	2.795908	-2.302124
H	0.718130	2.919383	-3.148020
H	2.431924	2.541297	-2.589329
H	2.807970	5.040932	-0.999078
H	3.162365	3.444784	-0.344762
K	-1.943993	3.376847	1.868592
Cl	-2.060908	4.664377	-0.707287
C	4.972885	-0.625908	-0.438548
C	6.288310	-1.284332	-0.104086
F	7.023548	-0.540340	0.734873
F	7.020901	-1.525466	-1.201068
F	6.082134	-2.477139	0.502075
H	-4.525976	-0.180182	-1.149800

TS-CD-trans+13-CH SCF Done: -3531.12750735 A.U.

Au	1.230666	0.098555	0.367050
N	0.714890	-1.740828	-0.647058
N	-1.962040	0.924822	0.732896
N	-3.110260	0.267058	0.617997
N	-2.778109	-0.918496	0.157915

C	1.596197	-2.541694	-1.263063
H	2.639608	-2.227716	-1.244726
C	1.197002	-3.721528	-1.885672
H	1.942854	-4.345148	-2.380332
C	-0.152748	-4.078546	-1.851157
H	-0.495177	-5.001609	-2.323521
C	-0.611355	-2.051240	-0.644486
C	-1.431765	-1.014230	-0.035165
C	-0.877471	0.191691	0.371686
C	-1.977345	2.307397	1.155082
C	-1.892005	2.578341	2.534751
C	-1.846731	3.929341	2.907148
H	-1.777058	4.199660	3.961411
C	-1.903534	4.938157	1.943371
H	-1.869261	5.984586	2.256193
C	-2.016550	4.627394	0.587398
H	-2.071336	5.433882	-0.144288
C	-2.058111	3.294545	0.150691
C	-1.926538	1.457316	3.570071
H	-1.458009	0.566222	3.116089
C	-3.386889	1.087473	3.901035
H	-3.419248	0.248838	4.613740
H	-3.945176	0.796554	2.999995
H	-3.904110	1.945601	4.358457
C	-1.148990	1.776042	4.854466
H	-1.641847	2.564958	5.443232
H	-0.118738	2.107874	4.650101
H	-1.098429	0.882410	5.495139
C	-2.245602	2.924440	-1.314933
H	-1.715404	1.978216	-1.509433
C	-1.685041	3.960156	-2.296383
H	-0.621122	4.178766	-2.129289
H	-2.245409	4.908288	-2.263498
H	-1.786638	3.572788	-3.322427
C	-3.737718	2.669850	-1.609761
H	-4.172527	1.928082	-0.926556
H	-3.850318	2.289528	-2.636159
H	-4.320308	3.600524	-1.513748
C	-3.769917	-1.969875	0.078910
C	-3.858346	-2.838151	1.188087
C	-4.760278	-3.903929	1.081140
H	-4.860735	-4.609279	1.908205
C	-5.541111	-4.070317	-0.064623
H	-6.240140	-4.907889	-0.125655
C	-5.447796	-3.168399	-1.123594
H	-6.081381	-3.300448	-2.002567
C	-4.554716	-2.086141	-1.082512
C	-3.021480	-2.652634	2.450854
H	-2.539165	-1.664166	2.404108

C	-3.890535	-2.652405	3.718906
H	-3.274098	-2.417001	4.600860
H	-4.353887	-3.635029	3.897284
H	-4.694608	-1.904477	3.652098
C	-1.903671	-3.706842	2.538336
H	-1.242873	-3.672790	1.658220
H	-2.325132	-4.722861	2.598778
H	-1.286842	-3.544646	3.436725
C	-4.485411	-1.090673	-2.229737
H	-3.644780	-0.406373	-2.051836
C	-4.193737	-1.776008	-3.572694
H	-3.245711	-2.331346	-3.532827
H	-4.077497	-1.013050	-4.356314
H	-5.004057	-2.459613	-3.874381
C	-5.778055	-0.257914	-2.285290
H	-5.969504	0.254845	-1.329989
H	-6.653421	-0.888669	-2.510557
H	-5.702115	0.506414	-3.073934
C	3.237694	-0.442311	0.409160
C	4.128281	0.012129	-0.570660
H	3.786552	0.735492	-1.313945
C	5.444755	-0.461782	-0.578290
H	6.144584	-0.119950	-1.343900
C	4.980076	-1.822328	1.374316
H	5.314087	-2.541974	2.124355
C	3.661883	-1.353049	1.385041
H	2.973548	-1.720990	2.150977
C	-1.067873	-3.236376	-1.223970
O	1.912593	1.903948	-1.481162
O	1.730085	3.656404	-2.859552
C	1.892174	3.157294	-1.735066
C	2.116448	4.105577	-0.548891
H	1.668091	5.080813	-0.781709
H	3.205513	4.271867	-0.468358
C	1.568350	3.559913	0.764854
C	2.196864	2.311948	1.253288
H	3.257082	2.163233	1.019866
C	1.592360	1.420083	2.135418
H	2.222568	0.763996	2.743244
H	0.605498	1.666605	2.530510
H	1.783779	4.284978	1.579406
H	0.473673	3.460668	0.748352
K	0.918112	1.481860	-4.069790
Cl	-1.159179	-0.193952	-3.018728
C	5.869362	-1.376439	0.391758
C	7.308944	-1.838010	0.410361
F	7.823108	-1.891643	-0.829103
F	8.076059	-0.997528	1.126152
F	7.428832	-3.057214	0.960591

H	-2.131286	-3.467460	-1.206376

88

A-ad+14-CH SCF Done: -2005.13646973 A.U.

Au	-0.823131	1.524026	0.219060
N	1.912014	2.844242	-0.784296
N	0.773083	-1.044362	0.318207
N	1.963706	-1.601840	0.228986
N	2.787839	-0.598915	0.031453
C	2.375106	4.061595	-1.056730
H	1.654854	4.774384	-1.472579
C	3.705943	4.442052	-0.839991
H	4.039407	5.451828	-1.087108
C	4.578474	3.499779	-0.296746
H	5.622119	3.753131	-0.097173
C	2.753818	1.929349	-0.274023
C	2.137937	0.612250	-0.016069
C	0.786024	0.317392	0.175205
C	-0.369065	-1.911318	0.504746
C	-0.822833	-2.143831	1.815324
C	-1.893641	-3.036904	1.962377
H	-2.277024	-3.254680	2.961491
C	-2.467772	-3.661634	0.854447
H	-3.299584	-4.356384	0.987908
C	-2.001122	-3.388597	-0.432197
H	-2.473376	-3.873529	-1.289075
C	-0.935399	-2.501577	-0.639825
C	-0.208254	-1.458303	3.028602
H	0.666912	-0.881656	2.692009
C	0.294567	-2.471977	4.068982
H	0.798993	-1.950144	4.897526
H	1.009817	-3.179072	3.622687
H	-0.533087	-3.055400	4.501929
C	-1.200852	-0.455776	3.643433
H	-2.102628	-0.968114	4.016104
H	-1.515471	0.294506	2.899994
H	-0.741915	0.075675	4.491947
C	-0.445543	-2.195674	-2.049409
H	0.416208	-1.515152	-1.978816
C	-1.533169	-1.458250	-2.848140
H	-1.822777	-0.523429	-2.342543
H	-2.437597	-2.077538	-2.958594
H	-1.169224	-1.207379	-3.857028
C	0.040345	-3.462676	-2.770815
H	0.827648	-3.970650	-2.192821
H	0.451704	-3.205762	-3.759484
H	-0.781346	-4.178992	-2.927495
C	4.186455	-0.900609	-0.172454
C	4.989816	-1.094782	0.968153

C	6.340611	-1.384823	0.739443
H	7.008611	-1.549990	1.586005
C	6.846394	-1.466623	-0.560503
H	7.904120	-1.692778	-0.714612
C	6.015611	-1.266058	-1.662287
H	6.429180	-1.338914	-2.670182
C	4.652946	-0.978554	-1.495564
C	4.408150	-1.025487	2.375507
H	3.486052	-0.421590	2.327580
C	4.010229	-2.432440	2.860806
H	3.547033	-2.379311	3.858653
H	4.897022	-3.082470	2.930313
H	3.295419	-2.905652	2.171520
C	5.341049	-0.331230	3.377736
H	5.655202	0.662347	3.021006
H	6.247979	-0.924232	3.573154
H	4.827231	-0.200533	4.342521
C	3.745840	-0.774727	-2.702886
H	2.730946	-0.549055	-2.341566
C	4.197561	0.427842	-3.548450
H	4.241945	1.347384	-2.944345
H	3.495292	0.596863	-4.379716
H	5.195256	0.261046	-3.984426
C	3.642013	-2.060456	-3.540725
H	3.279766	-2.903691	-2.932912
H	4.617439	-2.343734	-3.966651
H	2.941353	-1.915536	-4.378225
C	4.101076	2.220444	-0.002849
I	-3.186292	2.940713	0.276414
C	-4.113850	1.028845	-0.142934
C	-4.301329	0.139883	0.915884
C	-4.371666	0.692322	-1.471411
H	-4.089424	0.425732	1.946764
C	-4.725006	-1.156774	0.624498
C	-4.775471	-0.613162	-1.757220
H	-4.225921	1.408693	-2.281052
H	-4.864192	-1.896482	1.411191
C	-4.919247	-1.517914	-0.707789
H	-4.946698	-0.939880	-2.782647
O	-5.057392	-3.287196	-2.179990
O	-5.547607	-3.654989	-0.100921
N	-5.211638	-2.932334	-1.022547
H	4.764038	1.474732	0.430997

88

TS-AB-cis+14-CH SCF Done: -2005.11401764 A.U.

Au	-0.968669	1.493526	-0.016662
N	1.403609	2.806633	-0.089944
N	0.629016	-1.229381	0.066726

N	1.859121	-1.712844	0.069816
N	2.637314	-0.656501	0.010391
C	1.681177	4.104817	-0.141548
H	0.824006	4.786729	-0.138053
C	2.993187	4.592216	-0.197386
H	3.182118	5.666411	-0.238203
C	4.036421	3.667146	-0.199182
H	5.075699	4.000509	-0.242272
C	2.402934	1.902536	-0.090769
C	1.917682	0.511311	-0.029646
C	0.574848	0.132553	0.006758
C	-0.470979	-2.169947	0.122688
C	-0.956447	-2.541277	1.390675
C	-1.961374	-3.517433	1.416327
H	-2.375618	-3.846428	2.370053
C	-2.446708	-4.078573	0.234644
H	-3.237265	-4.830387	0.279408
C	-1.959283	-3.662650	-1.004375
H	-2.373784	-4.100374	-1.913195
C	-0.950014	-2.693478	-1.092185
C	-0.366869	-1.963947	2.671935
H	0.057618	-0.975454	2.430044
C	0.783772	-2.856068	3.176410
H	1.253755	-2.415797	4.069836
H	1.559212	-2.988083	2.407583
H	0.406774	-3.855111	3.447009
C	-1.412424	-1.737488	3.772223
H	-1.816255	-2.686086	4.158408
H	-2.256644	-1.133104	3.407760
H	-0.956312	-1.210179	4.624208
C	-0.345731	-2.287425	-2.431097
H	0.124572	-1.298344	-2.302904
C	-1.388496	-2.137470	-3.546877
H	-2.187998	-1.439669	-3.257476
H	-1.855178	-3.099788	-3.807800
H	-0.910754	-1.751094	-4.460435
C	0.760845	-3.281118	-2.833593
H	1.538200	-3.352908	-2.058519
H	1.238882	-2.969151	-3.775298
H	0.339909	-4.288110	-2.982582
C	4.069788	-0.844328	-0.002652
C	4.743780	-0.843101	1.233153
C	6.140680	-0.948142	1.184199
H	6.711971	-0.946825	2.114521
C	6.810860	-1.057084	-0.035122
H	7.900567	-1.134541	-0.048555
C	6.101579	-1.079974	-1.237036
H	6.643077	-1.179870	-2.179624
C	4.703651	-0.979216	-1.252511

C	4.018436	-0.714855	2.567589
H	2.934767	-0.750925	2.377306
C	4.344303	-1.892734	3.500332
H	3.752902	-1.819342	4.426047
H	5.407190	-1.900048	3.788052
H	4.115002	-2.857403	3.023114
C	4.321966	0.636720	3.237942
H	4.043160	1.482223	2.589991
H	5.394177	0.731894	3.472445
H	3.763061	0.731819	4.182102
C	3.935085	-0.990061	-2.568943
H	2.859244	-1.036813	-2.340476
C	4.180920	0.304625	-3.364080
H	3.895307	1.196886	-2.785527
H	3.594569	0.301022	-4.296311
H	5.243608	0.406436	-3.636357
C	4.264041	-2.236062	-3.406894
H	4.077582	-3.160909	-2.840652
H	5.316191	-2.241704	-3.731570
H	3.640721	-2.258293	-4.314093
C	3.747129	2.300050	-0.145550
I	-2.969361	3.308642	-0.022174
C	-3.205002	0.886474	-0.046139
C	-3.584000	0.309068	1.172292
C	-3.563291	0.337685	-1.284356
H	-3.332899	0.782726	2.121461
C	-4.257925	-0.909426	1.145610
C	-4.238476	-0.880583	-1.296437
H	-3.297961	0.832978	-2.218603
H	-4.537354	-1.420575	2.066438
H	-4.505444	-1.369590	-2.232967
C	-4.559112	-1.494414	-0.085134
O	-5.457068	-3.337895	0.968313
O	-5.355331	-3.351182	-1.195429
N	-5.186730	-2.827234	-0.106457
H	4.551784	1.568480	-0.147140

88

TS-AB-trans+14-CH SCF Done: -2005.10608704 A.U.

Au	-1.360952	0.189390	-0.032246
N	-0.415534	-2.334678	-0.111348
N	1.620602	1.201757	0.072127
N	2.873502	0.785494	0.064210
N	2.787899	-0.526883	0.018407
C	-1.066909	-3.491857	-0.160549
H	-2.158999	-3.432522	-0.211326
C	-0.405067	-4.726404	-0.152060
H	-0.973595	-5.657131	-0.194169
C	0.988273	-4.724347	-0.087145

H	1.544953	-5.664089	-0.075985
C	0.932838	-2.319007	-0.052757
C	1.483680	-0.954372	-0.005837
C	0.703216	0.196922	0.028866
C	1.319590	2.613451	0.133274
C	1.123668	3.294853	-1.082776
C	0.790405	4.652176	-0.992683
H	0.634203	5.232372	-1.902940
C	0.651697	5.275702	0.249465
H	0.389848	6.335494	0.295161
C	0.838742	4.559356	1.431255
H	0.713824	5.062678	2.391794
C	1.179927	3.199585	1.402396
C	1.307893	2.594591	-2.423842
H	1.080230	1.526131	-2.274201
C	2.774880	2.694545	-2.884312
H	2.924380	2.144033	-3.826640
H	3.462957	2.281360	-2.131453
H	3.055816	3.745856	-3.055741
C	0.344264	3.098168	-3.506276
H	0.571527	4.131414	-3.811775
H	-0.700920	3.066027	-3.160484
H	0.429108	2.471026	-4.407274
C	1.327123	2.398775	2.690496
H	1.773305	1.423818	2.441396
C	-0.055510	2.124725	3.309784
H	-0.714731	1.602681	2.597958
H	-0.552796	3.065424	3.595025
H	0.039844	1.502921	4.214205
C	2.274363	3.071569	3.695247
H	3.262103	3.261633	3.248473
H	2.415150	2.425188	4.575745
H	1.873925	4.031728	4.056002
C	4.003019	-1.307134	-0.010170
C	4.552937	-1.621654	-1.264816
C	5.706515	-2.419982	-1.258969
H	6.172119	-2.698168	-2.206472
C	6.267469	-2.859990	-0.060501
H	7.166977	-3.479823	-0.080179
C	5.696174	-2.512710	1.166892
H	6.159668	-2.861333	2.090585
C	4.541321	-1.722829	1.224294
C	3.933600	-1.151057	-2.575375
H	3.109626	-0.459908	-2.340065
C	4.941856	-0.362809	-3.426815
H	4.450969	0.028757	-4.331279
H	5.780220	-0.997109	-3.754604
H	5.357951	0.488719	-2.867543
C	3.331672	-2.332307	-3.356769

H	2.574906	-2.867017	-2.761270
H	4.109755	-3.058389	-3.641453
H	2.850369	-1.977147	-4.281519
C	3.921481	-1.287937	2.547471
H	2.846104	-1.114077	2.372653
C	4.023997	-2.353506	3.646514
H	3.627534	-3.324288	3.310258
H	3.450346	-2.037294	4.531159
H	5.063896	-2.504997	3.975432
C	4.533307	0.050421	3.006609
H	4.414614	0.832498	2.242204
H	5.610236	-0.066345	3.206705
H	4.049716	0.397937	3.933013
C	1.676273	-3.507522	-0.036538
I	-3.141858	2.300031	-0.259040
C	-3.459604	-0.186082	-0.050010
C	-3.926387	-0.600318	1.213281
C	-3.881532	-0.826222	-1.232266
H	-3.643278	-0.057020	2.116417
C	-4.746316	-1.724888	1.298986
C	-4.697344	-1.952334	-1.139474
H	-3.563047	-0.456552	-2.208394
H	-5.111527	-2.093881	2.257704
H	-5.023334	-2.495842	-2.026615
C	-5.110460	-2.385074	0.123539
O	-6.212181	-4.182846	-0.819493
O	-6.348390	-3.918824	1.328819
N	-5.965805	-3.594898	0.218959
H	2.763325	-3.487407	0.014390

88

B-cis+14-CH SCF Done: -2005.14913439 A.U.

Au	-0.874012	1.552241	-0.092039
N	1.138988	2.611269	-0.070661
N	0.512665	-1.383898	0.057548
N	1.752129	-1.848244	0.112526
N	2.526457	-0.788779	0.095999
C	1.333494	3.932559	-0.108706
H	0.434319	4.551018	-0.168821
C	2.614487	4.491637	-0.073819
H	2.731047	5.575945	-0.107427
C	3.714846	3.639922	0.003806
H	4.729558	4.042928	0.033502
C	2.205340	1.768494	0.003955
C	1.795454	0.364360	0.036291
C	0.459886	-0.021941	0.009784
C	-0.573916	-2.347551	0.024162
C	-1.256733	-2.621645	1.226069
C	-2.298272	-3.555593	1.150579

H	-2.874951	-3.794887	2.044090
C	-2.613613	-4.189251	-0.052967
H	-3.439885	-4.902130	-0.089260
C	-1.899042	-3.901923	-1.214512
H	-2.166838	-4.405856	-2.143920
C	-0.860245	-2.961363	-1.208687
C	-0.830921	-1.987475	2.546859
H	-0.544185	-0.942700	2.334171
C	0.405812	-2.715156	3.114748
H	0.764875	-2.211727	4.026164
H	1.235774	-2.758573	2.395966
H	0.147505	-3.752377	3.380034
C	-1.941039	-1.941685	3.603532
H	-2.191514	-2.948290	3.972852
H	-2.859762	-1.483833	3.213333
H	-1.606458	-1.354226	4.472096
C	-0.069395	-2.650502	-2.474387
H	0.387089	-1.652878	-2.351549
C	-0.946047	-2.583621	-3.733982
H	-1.803948	-1.906714	-3.603928
H	-1.339370	-3.573592	-4.011076
H	-0.352632	-2.222412	-4.587927
C	1.071630	-3.669616	-2.657575
H	1.747193	-3.681648	-1.789934
H	1.662689	-3.432097	-3.555424
H	0.662074	-4.684719	-2.779724
C	3.963360	-0.940949	0.146281
C	4.580045	-0.908291	1.411286
C	5.979911	-0.982109	1.425333
H	6.508494	-0.962941	2.380414
C	6.705523	-1.084291	0.237826
H	7.796164	-1.137321	0.273323
C	6.051956	-1.130581	-0.995063
H	6.638653	-1.222030	-1.910726
C	4.654231	-1.066013	-1.074986
C	3.793398	-0.798151	2.711870
H	2.719977	-0.772111	2.469005
C	4.017253	-2.034040	3.599501
H	3.391854	-1.972683	4.503427
H	5.066619	-2.110800	3.924505
H	3.758897	-2.961606	3.066583
C	4.121175	0.505233	3.460546
H	3.924088	1.391565	2.836752
H	5.178625	0.537887	3.766712
H	3.508750	0.587891	4.371869
C	3.942213	-1.093634	-2.423004
H	2.869570	-1.261492	-2.241173
C	4.083836	0.258441	-3.145840
H	3.680382	1.087886	-2.544219

H	3.543819	0.241543	-4.105375
H	5.141554	0.482155	-3.357832
C	4.425042	-2.253185	-3.308062
H	4.327584	-3.220028	-2.792311
H	5.477320	-2.129023	-3.606955
H	3.828111	-2.295652	-4.232000
C	-2.572404	0.427435	-0.070488
C	-3.336840	0.348110	1.096716
H	-3.105455	0.963667	1.966898
C	-4.407368	-0.546807	1.148162
H	-5.016625	-0.657394	2.045216
C	-3.958702	-1.217695	-1.153843
H	-4.210558	-1.851478	-2.003116
C	-2.899034	-0.312934	-1.208171
H	-2.316949	-0.221719	-2.125832
I	-2.403831	3.728984	-0.269925
C	3.513266	2.256224	0.043650
C	-4.676342	-1.340510	0.034352
O	-6.391080	-2.417087	1.145737
O	-5.769055	-3.204464	-0.774819
N	-5.703255	-2.397890	0.140388
H	4.355263	1.569056	0.103465

88
TS-decomp-cis+14-CH SCF Done: -2005.10518452 A.U.

Au	-1.677286	-0.977773	-0.423411
N	0.347528	-1.566789	-2.359688
N	0.483275	0.508129	1.261805
N	1.786658	0.486266	1.431037
N	2.278607	-0.017280	0.323998
C	0.364314	-2.240530	-3.504835
H	-0.594585	-2.645965	-3.843926
C	1.538754	-2.436452	-4.244823
H	1.515285	-3.002551	-5.177869
C	2.723686	-1.887284	-3.756292
H	3.660726	-2.006290	-4.304635
C	1.488030	-1.048185	-1.871222
C	1.304770	-0.358194	-0.580123
C	0.097084	-0.006116	0.045405
C	-0.355062	1.062405	2.303921
C	-0.405797	2.467844	2.430998
C	-1.294435	2.971961	3.389591
H	-1.383078	4.049953	3.527823
C	-2.073194	2.115485	4.169266
H	-2.767774	2.534278	4.901251
C	-1.958749	0.730307	4.042153
H	-2.551830	0.082778	4.688167
C	-1.079518	0.161313	3.110114
C	0.497127	3.396108	1.623755

H	0.720053	2.897495	0.664022
C	1.827655	3.613354	2.375986
H	2.504545	4.248379	1.783392
H	2.339931	2.666058	2.591863
H	1.640806	4.122472	3.334499
C	-0.134675	4.755015	1.288542
H	-0.259591	5.378473	2.187080
H	-1.114517	4.659305	0.802671
H	0.525544	5.311101	0.605439
C	-0.823160	-1.342139	3.072536
H	-0.561391	-1.624788	2.040943
C	-2.039658	-2.189109	3.459577
H	-2.924273	-1.924330	2.861878
H	-2.290748	-2.082845	4.526375
H	-1.824377	-3.253076	3.280786
C	0.399359	-1.673306	3.952989
H	1.292475	-1.110464	3.640031
H	0.632795	-2.748087	3.895599
H	0.198057	-1.423643	5.006715
C	3.709416	-0.203517	0.227129
C	4.488851	0.895606	-0.189398
C	5.860710	0.657419	-0.339483
H	6.517647	1.466486	-0.660445
C	6.406106	-0.602149	-0.074036
H	7.479582	-0.761250	-0.200222
C	5.600506	-1.651090	0.367175
H	6.050499	-2.620005	0.592102
C	4.218940	-1.476935	0.535135
C	3.866705	2.257922	-0.486011
H	2.980424	2.360240	0.156708
C	4.794327	3.428794	-0.135022
H	4.245850	4.379085	-0.223654
H	5.656592	3.491004	-0.817094
H	5.175213	3.348297	0.894213
C	3.390000	2.354647	-1.947818
H	2.630060	1.596659	-2.189684
H	4.232195	2.220494	-2.645236
H	2.945491	3.343704	-2.140234
C	3.344409	-2.618181	1.041334
H	2.301104	-2.268026	1.084441
C	3.376294	-3.826686	0.090437
H	3.067172	-3.547298	-0.928495
H	2.695100	-4.613142	0.450495
H	4.385408	-4.263690	0.029770
C	3.738027	-3.012833	2.475594
H	3.688006	-2.149770	3.156828
H	4.762392	-3.415429	2.513650
H	3.058558	-3.790968	2.856706
C	-1.437295	1.083322	-0.859021

C	-0.985612	1.501641	-2.117479
H	-0.347671	0.859263	-2.722788
C	-1.374983	2.753932	-2.591623
H	-1.047166	3.123199	-3.563539
C	-2.671970	3.125505	-0.554010
H	-3.336681	3.770464	0.020419
C	-2.271649	1.882418	-0.063984
H	-2.628023	1.545203	0.908135
I	-3.308357	-3.002904	-0.407494
C	2.709606	-1.177688	-2.551740
C	-2.200014	3.549325	-1.794996
O	-2.268861	5.193341	-3.415526
O	-3.128462	5.636770	-1.473339
N	-2.567961	4.905685	-2.271350
H	3.627092	-0.741284	-2.165546

103
TS-CD-cis+14-CH SCF Done: -3398.68853963 A.U.

Au	-0.714524	-1.001575	-1.325958
N	1.414238	-1.493253	-1.803077
N	0.094864	1.514403	0.532375
N	1.227010	2.084328	0.917961
N	2.180804	1.426520	0.294692
C	1.843417	-2.541771	-2.506923
H	1.094772	-3.225440	-2.897583
C	3.205984	-2.739926	-2.790163
H	3.504381	-3.613866	-3.368976
C	4.129934	-1.836492	-2.292796
H	5.196159	-1.963004	-2.491446
C	2.323336	-0.613202	-1.280752
C	1.673655	0.428299	-0.488192
C	0.292263	0.493737	-0.348026
C	-1.147482	2.019974	1.092522
C	-1.742917	3.126446	0.460225
C	-2.930034	3.610159	1.028873
H	-3.442413	4.457160	0.571029
C	-3.465585	3.024283	2.175224
H	-4.396904	3.409467	2.595909
C	-2.838655	1.930474	2.773823
H	-3.288094	1.480015	3.658730
C	-1.664576	1.384850	2.239903
C	-1.098180	3.806778	-0.742173
H	-0.484603	3.052818	-1.265351
C	-0.158760	4.935114	-0.271760
H	0.345335	5.402419	-1.131954
H	0.610030	4.564097	0.421424
H	-0.732775	5.716493	0.250837
C	-2.113443	4.338595	-1.762585
H	-2.687901	5.188608	-1.363383

H	-2.826991	3.560564	-2.067577
H	-1.589514	4.698784	-2.661381
C	-0.995321	0.158736	2.854746
H	-0.610138	-0.456622	2.023806
C	-1.957285	-0.741910	3.641799
H	-2.872729	-0.961542	3.072892
H	-2.258500	-0.281926	4.596176
H	-1.473632	-1.704751	3.859816
C	0.191929	0.563095	3.750244
H	0.916619	1.201346	3.226691
H	0.717285	-0.333292	4.118831
H	-0.162188	1.124998	4.628904
C	3.560205	1.825485	0.453262
C	4.059883	2.801883	-0.431203
C	5.419525	3.118879	-0.308299
H	5.859356	3.864582	-0.972622
C	6.219671	2.497648	0.651903
H	7.276627	2.762901	0.729013
C	5.680599	1.547561	1.520010
H	6.320115	1.084152	2.273915
C	4.329197	1.184053	1.441910
C	3.194204	3.456048	-1.502555
H	2.139207	3.254750	-1.262569
C	3.353320	4.983759	-1.527161
H	2.646061	5.423698	-2.246872
H	4.364588	5.286282	-1.840158
H	3.154230	5.424518	-0.538867
C	3.480924	2.838370	-2.883712
H	3.307630	1.750892	-2.888253
H	4.527101	3.012157	-3.182255
H	2.831659	3.289920	-3.650094
C	3.748610	0.152084	2.401670
H	2.683376	0.014778	2.157301
C	4.438788	-1.214889	2.247671
H	4.328854	-1.617760	1.227787
H	4.021339	-1.939016	2.968219
H	5.516186	-1.151900	2.463426
C	3.812983	0.655444	3.853717
H	3.308211	1.626876	3.961459
H	4.855008	0.776045	4.188604
H	3.323115	-0.059807	4.532688
C	-2.572390	-0.243677	-0.994155
C	-3.123444	0.639510	-1.931029
H	-2.608194	0.866930	-2.866413
C	-4.336928	1.269813	-1.647701
H	-4.789276	1.980919	-2.338878
C	-4.445418	0.094136	0.486814
H	-4.964612	-0.074522	1.429660
C	-3.243231	-0.549298	0.190858

H	-2.797531	-1.244679	0.900323
C	3.686980	-0.755596	-1.510038
O	-0.874508	-2.678996	0.838102
O	-0.916597	-3.859603	2.730700
C	-1.314685	-3.628552	1.579503
C	-2.327197	-4.569539	0.918457
H	-3.193477	-4.709760	1.581313
H	-1.829622	-5.550518	0.845399
C	-2.729514	-4.058016	-0.460853
C	-1.567922	-3.624231	-1.271693
H	-0.613328	-4.160976	-1.126735
C	-1.660749	-2.710715	-2.347307
H	-0.963349	-2.870977	-3.178794
H	-2.666380	-2.403478	-2.655153
H	-3.197081	-4.870359	-1.057169
H	-3.484086	-3.255622	-0.417799
K	1.567409	-3.450112	1.930160
Cl	1.669775	-4.722681	-0.656569
C	-4.958372	1.013471	-0.425787
N	-6.153604	1.799887	-0.052956
O	-6.547529	1.703145	1.098633
O	-6.647524	2.513442	-0.908568
H	4.392656	-0.036531	-1.098112

103

TS-CD-trans+14-CH SCF Done: -3398.68258006 A.U.

Au	-1.387833	-0.027376	-0.394477
N	-0.770673	-1.835557	0.616490
N	1.746193	1.004460	-0.711448
N	2.932759	0.421540	-0.583557
N	2.672335	-0.786210	-0.135457
C	-1.606879	-2.693224	1.218961
H	-2.667622	-2.445546	1.190969
C	-1.141793	-3.848844	1.840965
H	-1.852699	-4.520428	2.324177
C	0.228020	-4.120647	1.820528
H	0.622025	-5.023132	2.292425
C	0.572512	-2.061755	0.629516
C	1.332677	-0.970811	0.037217
C	0.707110	0.199355	-0.370202
C	1.678530	2.387654	-1.126869
C	1.602358	2.660288	-2.506801
C	1.475970	4.007618	-2.873863
H	1.409015	4.278860	-3.928072
C	1.448900	5.012438	-1.904653
H	1.352567	6.056202	-2.213358
C	1.556853	4.701948	-0.548159
H	1.546037	5.506263	0.187849
C	1.676770	3.372148	-0.116627

C	1.731312	1.550397	-3.546655
H	1.317545	0.626351	-3.105448
C	3.219149	1.283864	-3.853633
H	3.321363	0.454058	-4.570023
H	3.780346	1.026173	-2.944329
H	3.683928	2.178414	-4.297315
C	0.955786	1.822618	-4.842991
H	1.402853	2.647393	-5.418986
H	-0.098458	2.081020	-4.655395
H	0.978467	0.931701	-5.488988
C	1.861583	3.006552	1.350513
H	1.388293	2.027925	1.531444
C	1.220844	4.000456	2.326108
H	0.148206	4.152546	2.142040
H	1.721061	4.981950	2.305931
H	1.329850	3.615963	3.352474
C	3.361385	2.844082	1.670728
H	3.854191	2.135486	0.991745
H	3.479556	2.465046	2.696965
H	3.885893	3.810050	1.589999
C	3.729001	-1.771749	-0.046671
C	3.889603	-2.625494	-1.158997
C	4.856089	-3.632120	-1.043507
H	5.014184	-4.324426	-1.872577
C	5.628342	-3.755372	0.113476
H	6.378490	-4.546944	0.181040
C	5.461535	-2.867818	1.175577
H	6.088968	-2.964704	2.063490
C	4.501694	-1.844633	1.126379
C	3.062318	-2.485603	-2.433860
H	2.516440	-1.530629	-2.388934
C	3.949616	-2.419969	-3.687544
H	3.333321	-2.219033	-4.578073
H	4.478884	-3.369044	-3.864111
H	4.702072	-1.621809	-3.603007
C	2.016622	-3.609179	-2.545819
H	1.340973	-3.624033	-1.676423
H	2.503765	-4.595426	-2.605251
H	1.405084	-3.481093	-3.453280
C	4.351536	-0.862433	2.277291
H	3.470486	-0.233540	2.090702
C	4.087093	-1.572603	3.612952
H	3.177936	-2.188341	3.557783
H	3.911370	-0.823117	4.398497
H	4.936035	-2.203686	3.922646
C	5.585937	0.052947	2.354915
H	5.756853	0.582249	1.404751
H	6.497487	-0.520339	2.589893
H	5.449025	0.806430	3.145765

C	-3.356441	-0.693763	-0.471240
C	-4.289630	-0.303419	0.498685
H	-4.003609	0.434306	1.251332
C	-5.575572	-0.853288	0.484922
H	-6.325937	-0.573458	1.224952
C	-4.993096	-2.170538	-1.485626
H	-5.296337	-2.895752	-2.241066
C	-3.708538	-1.618557	-1.465152
H	-2.987200	-1.931624	-2.224834
C	1.095660	-3.219176	1.208546
O	-2.207775	1.704097	1.449003
O	-2.168225	3.458737	2.835401
C	-2.278883	2.955197	1.707024
C	-2.553668	3.892053	0.522407
H	-2.182924	4.896089	0.768739
H	-3.650686	3.977709	0.425180
C	-1.945914	3.400402	-0.786802
C	-2.476633	2.117612	-1.299555
H	-3.530221	1.897884	-1.093502
C	-1.793372	1.274430	-2.171756
H	-2.362490	0.580111	-2.797102
H	-0.815664	1.587847	-2.541148
H	-2.200300	4.117478	-1.597306
H	-0.847424	3.379660	-0.753117
K	-1.224052	1.350204	4.064324
Cl	0.960348	-0.188524	3.017703
C	-5.904459	-1.779250	-0.505346
O	-7.520942	-3.169386	-1.399558
O	-8.037052	-2.009681	0.356746
N	-7.267821	-2.367631	-0.517512
H	2.171735	-3.382553	1.202822

111
TS-CD-cis+15-CH SCF Done: -3387.37070186 A.U.

Au	-0.876082	-0.413777	-1.416716
O	-5.226086	3.883884	-0.497249
N	0.952526	-1.663327	-1.840509
N	0.723161	1.576248	0.549073
N	1.967667	1.683302	0.992912
N	2.639211	0.732241	0.379688
C	1.000053	-2.779774	-2.567184
H	0.070810	-3.132772	-3.006579
C	2.204381	-3.466321	-2.799512
H	2.186220	-4.380783	-3.391982
C	3.370832	-2.977050	-2.235028
H	4.323433	-3.486620	-2.393960
C	2.094644	-1.189168	-1.254658
C	1.835762	0.005719	-0.453192
C	0.569301	0.573846	-0.358786

C	-0.268086	2.493119	1.084296
C	-0.360478	3.764171	0.491104
C	-1.284591	4.654641	1.054650
H	-1.400349	5.652756	0.630726
C	-2.057470	4.280406	2.152283
H	-2.766636	4.992730	2.580869
C	-1.946316	3.002068	2.700858
H	-2.577230	2.727211	3.546084
C	-1.051993	2.062327	2.173632
C	0.532118	4.176446	-0.673493
H	0.847519	3.256758	-1.195432
C	1.799822	4.881846	-0.153968
H	2.467104	5.144637	-0.989610
H	2.358422	4.247908	0.550080
H	1.532191	5.812182	0.371671
C	-0.192395	5.045959	-1.710624
H	-0.427401	6.045444	-1.312869
H	-1.132855	4.580481	-2.037921
H	0.449956	5.193844	-2.592637
C	-0.939312	0.651104	2.743843
H	-0.790063	-0.037342	1.895016
C	-2.205572	0.171090	3.466456
H	-3.110098	0.341963	2.864565
H	-2.342685	0.682467	4.432648
H	-2.136636	-0.909749	3.654720
C	0.274413	0.525473	3.685264
H	1.210203	0.853411	3.213062
H	0.395706	-0.519379	4.015073
H	0.130705	1.144210	4.585266
C	4.060289	0.593605	0.597969
C	4.921515	1.336181	-0.234039
C	6.296066	1.130937	-0.053155
H	7.007170	1.676396	-0.675709
C	6.770337	0.241438	0.912295
H	7.846609	0.099982	1.035305
C	5.883196	-0.462365	1.727371
H	6.274555	-1.142281	2.486767
C	4.497371	-0.304512	1.589099
C	4.402097	2.281145	-1.312185
H	3.341166	2.487345	-1.105395
C	5.122525	3.637633	-1.296525
H	4.654080	4.319925	-2.022308
H	6.182729	3.543245	-1.578178
H	5.072148	4.108132	-0.303161
C	4.483425	1.617949	-2.699639
H	3.915099	0.675571	-2.735555
H	5.528147	1.389611	-2.964850
H	4.077507	2.289714	-3.472150
C	3.536839	-1.069196	2.491899

H	2.507554	-0.818867	2.190762
C	3.704770	-2.591285	2.340343
H	3.529031	-2.921385	1.303483
H	3.008506	-3.122207	3.011952
H	4.716673	-2.919114	2.623014
C	3.694167	-0.628149	3.957063
H	3.559405	0.458577	4.062031
H	4.692241	-0.884901	4.345190
H	2.946572	-1.127014	4.593277
C	-2.351415	0.956714	-1.105202
C	-2.502605	2.021026	-2.003385
H	-1.854552	2.120504	-2.877047
C	-3.491813	2.975394	-1.777333
H	-3.631169	3.816851	-2.458836
C	-4.329284	2.886059	-0.649343
C	-4.180204	1.804786	0.232951
H	-4.810064	1.704971	1.116590
C	-3.187247	0.845092	-0.000009
H	-3.055732	0.021152	0.699649
C	-6.099392	3.871240	0.614964
H	-6.725083	4.769166	0.530794
H	-5.543561	3.907563	1.569448
H	-6.750786	2.978896	0.613543
C	3.320192	-1.821234	-1.435882
O	-1.651685	-2.032954	0.623645
O	-2.191206	-3.087648	2.507911
C	-2.448240	-2.728269	1.346228
C	-3.755800	-3.210381	0.674804
C	-3.858702	-2.560739	-0.718472
C	-2.602837	-2.567020	-1.501180
H	-1.916805	-3.420512	-1.376965
C	-2.330811	-1.641101	-2.533889
H	-1.708311	-2.005554	-3.360426
H	-3.147073	-0.977587	-2.838930
H	-4.583048	-3.129747	-1.338409
H	-4.260219	-1.535373	-0.672920
K	0.276826	-3.660335	1.684822
Cl	-0.056921	-4.908480	-0.900364
C	-4.953848	-2.803597	1.545202
H	-5.897553	-3.172098	1.112030
H	-4.838865	-3.226420	2.552954
H	-5.026587	-1.707624	1.642993
C	-3.670841	-4.748217	0.569798
H	-3.560687	-5.177278	1.575931
H	-4.590260	-5.154293	0.117876
H	-2.804973	-5.074694	-0.028766
H	4.220974	-1.420796	-0.974366

111

TS-CD-trans+15-CH SCF Done: -3387.36450317 A.U.

Au	1.326656	-0.456072	0.386610
O	6.679413	-3.635383	0.144921
N	0.401381	-2.085216	-0.706391
N	-1.590977	1.080084	0.773189
N	-2.860674	0.716301	0.631107
N	-2.810515	-0.495419	0.123191
C	1.077964	-3.051237	-1.342766
H	2.165828	-2.992044	-1.301329
C	0.420293	-4.081561	-2.010529
H	1.004513	-4.847243	-2.522684
C	-0.975954	-4.109297	-1.998805
H	-1.519827	-4.907828	-2.507750
C	-0.960225	-2.074372	-0.720679
C	-1.521807	-0.896818	-0.072945
C	-0.704451	0.127226	0.385350
C	-1.277933	2.412399	1.238898
C	-1.133477	2.613364	2.625500
C	-0.757104	3.899657	3.037534
H	-0.625216	4.112896	4.098900
C	-0.560484	4.919325	2.104194
H	-0.266964	5.914549	2.446682
C	-0.748512	4.687422	0.740833
H	-0.607654	5.505685	0.034199
C	-1.117329	3.420053	0.265308
C	-1.450640	1.505333	3.626407
H	-1.204697	0.540706	3.148365
C	-2.961527	1.489551	3.936383
H	-3.204774	0.663576	4.622770
H	-3.560886	1.367317	3.023162
H	-3.263006	2.433892	4.416536
C	-0.637489	1.591105	4.925215
H	-0.934119	2.459218	5.533866
H	0.445043	1.669623	4.737975
H	-0.813169	0.694306	5.538872
C	-1.412017	3.156568	-1.205939
H	-1.138757	2.114915	-1.439358
C	-0.627650	4.050750	-2.172760
H	0.459567	3.987190	-2.028429
H	-0.926770	5.108478	-2.095768
H	-0.848514	3.739197	-3.206078
C	-2.925191	3.294099	-1.470752
H	-3.520789	2.660113	-0.800438
H	-3.146873	2.992740	-2.505633
H	-3.253455	4.337003	-1.331065
C	-4.018336	-1.284532	0.009937
C	-4.303758	-2.155528	1.083208
C	-5.425536	-2.981594	0.941872
H	-5.684414	-3.679218	1.740687

C	-6.224054	-2.916541	-0.201833
H	-7.096078	-3.568952	-0.290600
C	-5.927721	-2.014758	-1.222854
H	-6.575710	-1.960113	-2.099522
C	-4.810699	-1.167895	-1.146295
C	-3.450441	-2.216428	2.347188
H	-2.743892	-1.372618	2.329270
C	-4.299711	-2.044524	3.617192
H	-3.649023	-1.992866	4.504520
H	-4.989082	-2.890076	3.765607
H	-4.897598	-1.121893	3.575540
C	-2.619544	-3.510606	2.398838
H	-1.966313	-3.609103	1.517965
H	-3.272894	-4.397075	2.430019
H	-1.985356	-3.528442	3.299630
C	-4.520686	-0.162052	-2.249055
H	-3.542036	0.298252	-2.056869
C	-4.409098	-0.829198	-3.627553
H	-3.614838	-1.589173	-3.632677
H	-4.126873	-0.075854	-4.377633
H	-5.358855	-1.290762	-3.943744
C	-5.584881	0.949414	-2.240076
H	-5.641865	1.446125	-1.259173
H	-6.585154	0.549918	-2.474280
H	-5.341799	1.712344	-2.995588
C	3.154718	-1.443363	0.387190
C	4.109696	-1.188033	-0.609682
H	3.932664	-0.389051	-1.332526
C	5.277123	-1.946536	-0.662225
H	6.033971	-1.766258	-1.428719
C	5.519448	-2.961992	0.284203
C	4.568207	-3.205064	1.289372
H	4.724607	-3.982002	2.037697
C	3.392612	-2.439903	1.335657
H	2.662048	-2.655792	2.120905
C	7.011790	-4.659549	1.063123
H	7.989938	-5.049396	0.753522
H	6.274290	-5.482041	1.041729
H	7.089337	-4.272703	2.095011
C	-1.675700	-3.096960	-1.346349
O	2.380287	1.244551	-1.353560
O	2.674415	3.042935	-2.642353
C	2.717764	2.461224	-1.544361
C	3.281091	3.236718	-0.322162
C	2.574433	2.757804	0.962206
C	2.810714	1.371182	1.415062
H	3.798043	0.939257	1.228658
C	1.938364	0.651466	2.226442
H	2.331063	-0.178693	2.821238

H	1.039271	1.146904	2.594798
H	2.975677	3.357239	1.809200
H	1.496731	2.977367	0.934002
K	1.347159	1.170067	-3.924112
Cl	-1.083677	-0.067802	-3.041414
C	3.044011	4.745432	-0.475603
H	3.537242	5.304863	0.336087
H	3.438425	5.089621	-1.439833
H	1.969544	4.982549	-0.450232
C	4.796704	2.952154	-0.279924
H	5.277601	3.382616	-1.170359
H	5.260365	3.408219	0.611030
H	5.020628	1.873948	-0.278047
H	-2.763747	-3.070842	-1.343661

111

TS-CD-cis+16-CH SCF Done: -3387.36225937 A.U.

Au	-0.979526	-0.615356	-1.190833
O	-5.378667	3.680827	-0.530767
N	0.889047	-1.796382	-1.689304
N	0.656509	1.584474	0.504281
N	1.927974	1.798213	0.811223
N	2.601053	0.853199	0.191599
C	0.953173	-2.958977	-2.338252
H	0.016592	-3.403769	-2.665212
C	2.177350	-3.572644	-2.651800
H	2.174063	-4.524099	-3.183487
C	3.352185	-2.964571	-2.240976
H	4.321280	-3.415954	-2.463222
C	2.041489	-1.205508	-1.254978
C	1.772729	0.022394	-0.508903
C	0.484121	0.514685	-0.321627
C	-0.330164	2.470823	1.097669
C	-0.598968	3.680700	0.435186
C	-1.508774	4.546797	1.057361
H	-1.759282	5.496660	0.583813
C	-2.091373	4.212956	2.279088
H	-2.786811	4.909774	2.753522
C	-1.807190	2.994363	2.896555
H	-2.292955	2.745541	3.840369
C	-0.925108	2.076952	2.312681
C	0.106914	4.063369	-0.860022
H	0.408278	3.129442	-1.364639
C	1.384289	4.869250	-0.552653
H	1.922183	5.111306	-1.482455
H	2.066975	4.316844	0.109451
H	1.127480	5.816921	-0.053207
C	-0.796645	4.824294	-1.839713
H	-1.036995	5.834282	-1.472570

H	-1.740569	4.288618	-2.012391
H	-0.282962	4.947815	-2.805823
C	-0.641952	0.719909	2.949598
H	-0.513951	-0.004998	2.128911
C	-1.802564	0.183206	3.798912
H	-2.764853	0.267283	3.272491
H	-1.892262	0.719679	4.757061
H	-1.650592	-0.885763	4.007550
C	0.653243	0.757424	3.782619
H	1.514738	1.118284	3.203997
H	0.889849	-0.245495	4.175932
H	0.537276	1.429506	4.647659
C	4.043899	0.829120	0.260908
C	4.752207	1.561495	-0.712510
C	6.149832	1.464941	-0.673312
H	6.746373	2.007531	-1.408557
C	6.791190	0.687376	0.292127
H	7.882045	0.628991	0.302666
C	6.052515	-0.009582	1.248775
H	6.573423	-0.601364	2.004115
C	4.651919	0.042826	1.256801
C	4.052155	2.383597	-1.789286
H	3.001909	2.519462	-1.490302
C	4.652831	3.790415	-1.930050
H	4.063256	4.380508	-2.648283
H	5.686838	3.759470	-2.307020
H	4.654376	4.324975	-0.968343
C	4.056723	1.633631	-3.134232
H	3.570942	0.648809	-3.053809
H	5.086705	1.471372	-3.490174
H	3.521870	2.215490	-3.901162
C	3.855856	-0.717456	2.310536
H	2.784905	-0.540960	2.122529
C	4.101494	-2.234285	2.220715
H	3.810619	-2.641081	1.238773
H	3.539050	-2.764120	3.008496
H	5.161698	-2.482606	2.380441
C	4.156012	-0.179578	3.719887
H	3.969148	0.902762	3.781478
H	5.205794	-0.358838	3.999825
H	3.519773	-0.678233	4.467661
C	-2.466484	0.759448	-0.938601
C	-2.827309	1.573765	-2.018656
H	-2.342106	1.474864	-2.992502
C	-3.824694	2.533487	-1.850143
H	-4.129885	3.180903	-2.674517
C	-4.453567	2.699442	-0.602311
C	-4.087367	1.868453	0.468526
H	-4.554237	1.968708	1.447570

C	-3.091892	0.900859	0.293728
H	-2.806315	0.254358	1.121861
C	-6.058958	3.908031	0.688073
H	-6.749774	4.742082	0.509108
H	-5.360929	4.185883	1.498342
H	-6.639486	3.022601	1.003614
C	3.289694	-1.762310	-1.516633
O	-1.591260	-1.935554	1.063305
O	-1.857813	-3.127371	2.927863
C	-2.150903	-2.857569	1.748223
C	-3.129281	-3.779157	1.016818
H	-3.973971	-4.023322	1.677703
H	-2.560399	-4.712656	0.866593
C	-3.626772	-3.253047	-0.341137
C	-2.461178	-2.893529	-1.202176
H	-1.597531	-3.569077	-1.073157
C	-2.427975	-1.954307	-2.241782
H	-1.788102	-2.184062	-3.103351
H	-3.333446	-1.390823	-2.480460
K	0.645337	-3.243723	2.180576
Cl	0.291220	-4.810346	-0.230348
C	-4.680656	-2.154718	-0.169402
H	-4.276891	-1.316463	0.408478
H	-5.044477	-1.763916	-1.131627
H	-5.547815	-2.562546	0.371006
C	-4.245980	-4.450402	-1.140872
H	-3.509381	-5.246838	-1.327833
H	-5.059943	-4.877365	-0.535060
H	-4.665079	-4.116276	-2.101856
H	4.197392	-1.268279	-1.175616

111
TS-CD-trans+16-CH SCF Done: -3387.35918479 A.U.

Au	1.382113	-0.327818	0.250777
O	6.718695	-3.547864	0.146028
N	0.487563	-2.019328	-0.765157
N	-1.599932	1.065650	0.777020
N	-2.848256	0.610809	0.733909
N	-2.749890	-0.600744	0.234463
C	1.177954	-2.965284	-1.418552
H	2.263222	-2.863571	-1.419977
C	0.537482	-4.027745	-2.050764
H	1.132582	-4.776264	-2.575620
C	-0.855788	-4.108381	-1.989449
H	-1.386536	-4.931051	-2.473173
C	-0.871340	-2.065223	-0.724593
C	-1.455015	-0.909901	-0.057968
C	-0.676298	0.174753	0.328253
C	-1.395341	2.427011	1.218084

C	-1.137667	2.650913	2.584040
C	-0.984177	3.985693	2.983570
H	-0.785844	4.219305	4.030437
C	-1.101918	5.026286	2.058863
H	-0.991130	6.060323	2.394267
C	-1.355793	4.760569	0.713508
H	-1.437523	5.588803	0.008119
C	-1.501707	3.444581	0.251060
C	-1.103353	1.500179	3.584803
H	-0.766778	0.598704	3.044782
C	-2.521789	1.216044	4.117149
H	-2.513702	0.352848	4.800684
H	-3.224517	1.001983	3.298993
H	-2.904937	2.087663	4.671077
C	-0.121041	1.722996	4.742706
H	-0.456077	2.528593	5.414200
H	0.887767	1.982768	4.384919
H	-0.043048	0.809358	5.352134
C	-1.812340	3.131516	-1.207162
H	-1.523760	2.090582	-1.417570
C	-1.037914	4.003683	-2.205622
H	0.052856	3.918906	-2.084803
H	-1.308210	5.069070	-2.131999
H	-1.290550	3.685132	-3.229513
C	-3.329154	3.227256	-1.454952
H	-3.890168	2.596819	-0.750857
H	-3.561683	2.885786	-2.475182
H	-3.685302	4.264294	-1.340947
C	-3.898534	-1.481377	0.227946
C	-4.006201	-2.383106	1.308876
C	-5.063706	-3.299851	1.261028
H	-5.184907	-4.024069	2.068894
C	-5.972511	-3.292057	0.201213
H	-6.791254	-4.015479	0.184863
C	-5.854315	-2.356973	-0.825808
H	-6.588089	-2.347617	-1.633814
C	-4.809357	-1.419666	-0.842470
C	-3.039475	-2.381463	2.490468
H	-2.401543	-1.487480	2.416604
C	-3.784260	-2.274762	3.831470
H	-3.063709	-2.183437	4.659516
H	-4.398885	-3.166548	4.029120
H	-4.444836	-1.395236	3.853071
C	-2.114227	-3.610709	2.462365
H	-1.535441	-3.662931	1.527543
H	-2.694808	-4.543091	2.547193
H	-1.403852	-3.579659	3.304000
C	-4.719591	-0.378807	-1.947161
H	-3.766552	0.157553	-1.846917

C	-4.701033	-1.014681	-3.344594
H	-3.851251	-1.703910	-3.450156
H	-4.565213	-0.229624	-4.103121
H	-5.637608	-1.550386	-3.570170
C	-5.864284	0.639355	-1.800967
H	-5.859920	1.111271	-0.806183
H	-6.847415	0.161391	-1.942550
H	-5.763606	1.433548	-2.556591
C	3.223226	-1.299258	0.275056
C	4.135178	-1.176754	-0.785350
H	3.930708	-0.463674	-1.586920
C	5.293707	-1.950373	-0.796940
H	6.016872	-1.872890	-1.611795
C	5.570848	-2.848052	0.253499
C	4.664436	-2.955704	1.321514
H	4.848249	-3.639313	2.150398
C	3.497228	-2.175962	1.326495
H	2.801844	-2.287158	2.163798
C	7.081967	-4.461560	1.163634
H	8.041691	-4.899375	0.860514
H	6.336498	-5.269926	1.271448
H	7.208966	-3.957226	2.138346
C	-1.569617	-3.117938	-1.320148
O	2.474568	1.218785	-1.762019
O	2.563164	3.021528	-3.094365
C	2.876276	2.390492	-2.071247
C	3.815332	3.048688	-1.051884
H	4.014333	4.087332	-1.348737
H	4.773755	2.501483	-1.057740
C	3.194405	3.018831	0.359623
C	3.153089	1.639211	0.905655
H	4.067222	1.052388	0.768700
C	2.161513	1.090229	1.731959
H	2.495986	0.348236	2.467521
H	1.333729	1.730513	2.040181
K	0.952816	1.198103	-4.090440
Cl	-1.383603	-0.071613	-3.029782
C	1.839266	3.726621	0.371740
H	1.110792	3.164268	-0.223835
H	1.433575	3.845294	1.385718
H	1.933840	4.729015	-0.068554
C	4.177494	3.726323	1.357008
H	5.175949	3.263590	1.355844
H	4.284774	4.771126	1.028539
H	3.783361	3.726642	2.384424
H	-2.656857	-3.131807	-1.280364

61
A-ad-cis+H SCF Done: -1464.60342966 A.U.

Au	-1.059461	-0.528388	0.107901
N	1.596294	-2.267966	-0.528576
N	0.905849	1.683561	0.816781
N	2.161217	2.073668	0.866437
N	2.828344	1.066149	0.362919
C	1.995685	-3.423171	-1.062226
H	1.296970	-4.264232	-0.999526
C	3.246405	-3.565106	-1.671130
H	3.571920	-4.515006	-2.098059
C	4.051607	-2.424563	-1.712393
H	5.040113	-2.439810	-2.183746
C	2.451901	-1.240994	-0.608816
C	2.002150	0.028712	-0.005657
C	0.701850	0.435823	0.304012
C	4.277268	1.150195	0.330133
C	4.876244	1.774864	-0.775506
C	6.274163	1.857236	-0.766987
H	6.787866	2.341922	-1.599553
C	7.018855	1.323770	0.285625
H	8.108936	1.395889	0.269359
C	6.383158	0.699997	1.359051
H	6.981435	0.286420	2.173246
C	4.985980	0.600734	1.411924
C	4.068871	2.335262	-1.937881
H	3.012398	2.062095	-1.787413
C	4.142117	3.871318	-1.969030
H	3.526454	4.271589	-2.790139
H	5.176779	4.215270	-2.127004
H	3.783987	4.306476	-1.023102
C	4.500247	1.711575	-3.275190
H	4.438090	0.613902	-3.231031
H	5.534297	1.986573	-3.536109
H	3.849556	2.066321	-4.090006
C	4.301603	-0.101561	2.578565
H	3.213788	0.042520	2.483199
C	4.565704	-1.617492	2.531835
H	4.241630	-2.054200	1.574461
H	4.027290	-2.130110	3.344474
H	5.639465	-1.833823	2.649216
C	4.709098	0.501614	3.932491
H	4.511534	1.584144	3.962895
H	5.779643	0.347865	4.139696
H	4.144313	0.024329	4.748672
N	3.654314	-1.263624	-1.187738
I	-3.422244	-1.848203	-0.170052
C	-4.722756	-0.116330	-0.130561
C	-4.812841	0.689350	-1.269990
C	-5.455365	0.148964	1.021542
H	-4.237729	0.469451	-2.171108

C	-5.660246	1.791095	-1.239870
C	-6.309749	1.257608	1.042841
H	-5.378130	-0.486202	1.905879
H	-5.761158	2.444141	-2.108625
C	-6.418552	2.086736	-0.088224
H	-6.883384	1.457909	1.947285
O	-7.207640	3.169709	-0.168145
C	-8.028534	3.532202	0.930308
H	-8.579612	4.428981	0.620715
H	-7.426231	3.769602	1.824894
H	-8.749010	2.732985	1.177469
H	0.195009	2.325236	1.159785

61

A-ad-trans+H SCF Done: -1464.60617707 A.U.

Au	1.063218	-1.731014	-0.029066
N	0.062486	1.236987	0.192060
N	-1.899844	-2.488307	0.003426
N	-3.141255	-2.052306	0.025716
N	-3.023358	-0.748728	-0.003496
C	0.514727	2.490234	0.147600
H	1.575233	2.637326	0.375091
C	-0.322538	3.564802	-0.168785
H	0.047430	4.590942	-0.194806
C	-1.654410	3.254964	-0.453824
H	-2.377394	4.033114	-0.721078
C	-1.233232	1.048783	-0.090821
C	-1.707092	-0.348206	-0.040855
C	-0.942708	-1.518371	-0.030945
C	-4.233639	0.050026	0.066115
C	-4.896253	0.347626	-1.135322
C	-6.070640	1.103702	-1.032180
H	-6.628289	1.354265	-1.936788
C	-6.534699	1.544415	0.207707
H	-7.451710	2.135720	0.264188
C	-5.838704	1.234516	1.376161
H	-6.215606	1.588256	2.337796
C	-4.664629	0.470152	1.335706
C	-4.381215	-0.110799	-2.492469
H	-3.405145	-0.599533	-2.344624
C	-5.322240	-1.155627	-3.115717
H	-4.923049	-1.509895	-4.079321
H	-6.320449	-0.728677	-3.302765
H	-5.447453	-2.025039	-2.451842
C	-4.141643	1.081113	-3.433734
H	-3.466323	1.815715	-2.969679
H	-5.083410	1.593760	-3.685235
H	-3.690481	0.738243	-4.378234
C	-3.898500	0.155254	2.614848

H	-3.088189	-0.549978	2.371404
C	-3.240206	1.426404	3.181072
H	-2.580069	1.902067	2.439268
H	-2.642181	1.188139	4.074827
H	-4.001317	2.167642	3.472371
C	-4.785930	-0.536896	3.661863
H	-5.247256	-1.449208	3.253678
H	-5.595087	0.124477	4.008979
H	-4.188722	-0.816937	4.543821
N	-2.108715	2.000259	-0.421230
I	3.781314	-1.843742	-0.004984
C	4.043229	0.304280	0.071270
C	3.930461	0.958341	1.301706
C	4.321821	0.993300	-1.104671
H	3.710767	0.410895	2.219792
C	4.103116	2.338209	1.342520
C	4.495498	2.381685	-1.057569
H	4.406229	0.474875	-2.061505
H	4.029142	2.884202	2.284858
C	4.388349	3.064050	0.167522
H	4.717286	2.912621	-1.982817
O	4.526730	4.392386	0.317148
C	4.879930	5.197600	-0.796376
H	4.965381	6.224597	-0.419875
H	4.105427	5.164457	-1.583201
H	5.848271	4.889017	-1.227008
H	-1.749046	-3.494416	0.015616

61

TS-AB-cis+H SCF Done: -1464.57607067 A.U.

Au	1.400347	-0.626954	0.014632
N	-1.020626	-2.338294	-0.229115
N	-0.576980	1.767553	0.221952
N	-1.840977	2.139018	0.232214
N	-2.505163	1.018213	0.093216
C	-1.313065	-3.629596	-0.374806
H	-0.468805	-4.325487	-0.423557
C	-2.636216	-4.075669	-0.461212
H	-2.877270	-5.133075	-0.580163
C	-3.630628	-3.097659	-0.387472
H	-4.692376	-3.359088	-0.447012
C	-2.049251	-1.479081	-0.168340
C	-1.665624	-0.066141	-0.005692
C	-0.365400	0.431387	0.080457
C	-3.955416	1.075535	0.069011
C	-4.635258	0.968155	1.293416
C	-6.033645	1.036637	1.240603
H	-6.609364	0.960489	2.165214
C	-6.699759	1.200038	0.025845

H	-7.790918	1.252636	0.009012
C	-5.983877	1.297814	-1.167515
H	-6.521031	1.424510	-2.109622
C	-4.584338	1.235101	-1.176628
C	-3.918791	0.761793	2.621579
H	-2.832967	0.761218	2.435553
C	-4.202651	1.911600	3.602060
H	-3.634975	1.769823	4.535219
H	-5.270512	1.958930	3.867496
H	-3.919852	2.883659	3.169347
C	-4.272119	-0.607447	3.228241
H	-4.054225	-1.422473	2.521107
H	-5.341374	-0.664665	3.486527
H	-3.696125	-0.781326	4.150827
C	-3.812972	1.316154	-2.487381
H	-2.736323	1.253690	-2.262348
C	-4.151199	0.124003	-3.399333
H	-3.973133	-0.832044	-2.883599
H	-3.535198	0.149270	-4.312087
H	-5.207787	0.146216	-3.709743
C	-4.044176	2.661332	-3.195416
H	-3.769618	3.506405	-2.545301
H	-5.099918	2.787048	-3.482991
H	-3.440288	2.721038	-4.114657
N	-3.339708	-1.801673	-0.241591
I	3.619135	-2.119818	-0.045080
C	3.412371	0.422663	0.108992
C	3.714412	0.935637	1.375810
C	3.768377	1.076574	-1.067804
H	3.476603	0.381395	2.284486
C	4.324470	2.183189	1.450479
C	4.383336	2.331286	-0.978796
H	3.574065	0.631983	-2.044585
H	4.565194	2.628668	2.417774
C	4.665990	2.894383	0.280126
H	4.647267	2.847663	-1.901559
O	5.260153	4.082797	0.469431
C	5.698130	4.844131	-0.644927
H	6.162784	5.749899	-0.235970
H	4.853190	5.133792	-1.294445
H	6.444825	4.292439	-1.242094
H	0.133280	2.490126	0.320522

61
TS-AB-trans+H SCF Done: -1464.58040400 A.U.

Au	-1.319428	-0.622887	0.086302
N	0.185764	1.678116	-0.383986
N	1.450781	-2.146550	0.483578
N	2.759840	-1.984077	0.476120

N	2.923731	-0.712593	0.206316
C	-0.105921	2.944001	-0.673961
H	-1.167738	3.196351	-0.765804
C	0.900439	3.898866	-0.853547
H	0.668364	4.937734	-1.092569
C	2.216889	3.452762	-0.710982
H	3.064344	4.135399	-0.833506
C	1.483676	1.346277	-0.270811
C	1.720803	-0.070516	0.043341
C	0.722796	-1.027024	0.227784
C	4.272573	-0.185528	0.117927
C	4.859310	0.314441	1.292260
C	6.163116	0.814206	1.176868
H	6.665209	1.211669	2.061237
C	6.827828	0.811142	-0.049601
H	7.845030	1.204392	-0.115568
C	6.205085	0.308383	-1.192164
H	6.739699	0.313734	-2.144153
C	4.902447	-0.204629	-1.137626
C	4.132321	0.345943	2.630307
H	3.139762	-0.113290	2.498358
C	4.869653	-0.483939	3.694063
H	4.301399	-0.491440	4.637539
H	5.865872	-0.066773	3.909879
H	5.004775	-1.525384	3.363794
C	3.900381	1.794549	3.094428
H	3.362537	2.374836	2.328868
H	4.855001	2.306770	3.293567
H	3.311788	1.812231	4.025300
C	4.221806	-0.728001	-2.395723
H	3.221986	-1.100514	-2.121729
C	4.016516	0.402503	-3.419223
H	3.457325	1.241494	-2.977057
H	3.459960	0.033792	-4.295300
H	4.981100	0.795267	-3.777878
C	4.988876	-1.914730	-3.001848
H	5.105573	-2.729252	-2.270496
H	5.994645	-1.614792	-3.335835
H	4.452583	-2.311368	-3.878283
N	2.509753	2.181319	-0.421017
I	-3.310839	-2.550524	0.089111
C	-3.258772	0.124171	-0.071142
C	-3.671168	0.566510	-1.347941
C	-3.823198	0.680892	1.091204
H	-3.310986	0.069786	-2.250903
C	-4.517742	1.658370	-1.446344
C	-4.675663	1.778695	0.990973
H	-3.585312	0.269628	2.074124
H	-4.826592	2.051045	-2.417047

C	-5.028870	2.278237	-0.280971
H	-5.074045	2.225261	1.901656
O	-5.851091	3.311305	-0.487197
C	-6.481381	3.961757	0.608884
H	-7.107844	4.751606	0.176816
H	-5.738687	4.416882	1.286627
H	-7.117814	3.262450	1.177023
H	1.090140	-3.079683	0.674150

80
A-ad-cis+Mes SCF Done: -1813.39594240 A.U.

Au	-0.799821	-1.416740	-0.327706
N	2.106121	-2.774240	0.566800
N	0.752711	1.148897	-0.356234
N	1.919136	1.748610	-0.210255
N	2.774123	0.776120	-0.010606
C	2.729396	-3.940761	0.738587
H	2.113527	-4.786293	1.063429
C	4.103093	-4.087176	0.521539
H	4.608930	-5.042232	0.671866
C	4.790610	-2.948927	0.094008
H	5.865961	-2.975607	-0.112508
C	2.861871	-1.743702	0.166529
C	2.165471	-0.457500	-0.018593
C	0.812335	-0.215943	-0.243660
C	-0.426399	1.940510	-0.614149
C	-0.733092	2.255243	-1.946248
C	-1.890709	3.009102	-2.171974
H	-2.155684	3.271032	-3.199824
C	-2.712465	3.437753	-1.119372
C	-2.363395	3.083440	0.192370
H	-3.004966	3.399831	1.017838
C	-1.220449	2.328982	0.475713
C	4.151229	1.122806	0.277252
C	5.026814	1.299156	-0.807069
C	6.344071	1.654997	-0.496855
H	7.064524	1.812185	-1.301107
C	6.752177	1.806100	0.830652
H	7.786771	2.080514	1.050095
C	5.852773	1.610206	1.877779
H	6.190823	1.729965	2.909109
C	4.517117	1.264991	1.624783
C	4.551378	1.141592	-2.244252
H	3.638749	0.523964	-2.226868
C	4.172407	2.511906	-2.836042
H	3.771073	2.397631	-3.855872
H	5.054809	3.169521	-2.890430
H	3.416206	3.018899	-2.217050
C	5.563517	0.400829	-3.128167

H 5.844309 -0.565047 -2.681373
H 6.481738 0.988455 -3.284245
H 5.129446 0.209130 -4.121870
C 3.540730 1.032853 2.771613
H 2.536915 0.872663 2.347998
C 3.909683 -0.240490 3.553175
H 3.945470 -1.120755 2.892318
H 3.171838 -0.436211 4.347327
H 4.898329 -0.140440 4.028681
C 3.437972 2.258018 3.694032
H 3.158768 3.160037 3.127669
H 4.391555 2.463104 4.205339
H 2.676746 2.088428 4.472087
N 4.172374 -1.780744 -0.089321
I -3.184829 -2.744949 -0.328005
C -4.175669 -0.892703 0.211442
C -4.736186 -0.105549 -0.797606
C -4.183415 -0.498514 1.545019
H -4.720618 -0.421160 -1.841993
C -5.311886 1.111209 -0.449836
C -4.761683 0.729182 1.887506
H -3.741693 -1.121222 2.324894
H -5.756453 1.754796 -1.210072
C -5.320335 1.544231 0.889335
H -4.761356 1.034250 2.933443
O -5.862022 2.758982 1.111988
C -5.959665 3.257754 2.436194
H -6.453236 4.234838 2.362848
H -4.963031 3.390491 2.893858
H -6.566636 2.592301 3.073799
C 0.147630 1.783376 -3.074665
H 1.171207 2.179733 -2.978658
H 0.224764 0.683043 -3.081362
H -0.248542 2.102306 -4.047980
C -0.859119 1.925354 1.881855
H -1.554238 2.363436 2.610585
H -0.892488 0.828592 1.995814
H 0.160884 2.248985 2.145811
C -3.932261 4.282789 -1.384953
H -4.351929 4.085599 -2.382949
H -4.715152 4.106231 -0.633398
H -3.671863 5.354735 -1.348474

80
A-ad-trans+Mes SCF Done: -1813.39163030 A.U.
Au -1.030963 1.129117 -0.172942
N -0.678197 -2.093082 0.015838
N 1.992069 1.210184 0.011029
N 3.108825 0.509160 0.053990

N	2.728240	-0.740082	-0.049488
C	-1.356377	-3.232652	-0.123996
H	-2.422111	-3.201817	0.125732
C	-0.741764	-4.410860	-0.559471
H	-1.298947	-5.343175	-0.663814
C	0.620335	-4.329811	-0.857794
H	1.180203	-5.201939	-1.212502
C	0.624956	-2.124555	-0.294586
C	1.361161	-0.855057	-0.152948
C	0.862371	0.445819	-0.110136
C	2.052019	2.647238	0.119515
C	2.044812	3.404656	-1.060777
C	2.075330	4.796852	-0.921555
H	2.072174	5.416155	-1.822511
C	2.112855	5.414560	0.337811
C	2.115221	4.606316	1.485255
H	2.140932	5.076424	2.471983
C	2.086188	3.209933	1.404294
C	3.740132	-1.770945	0.068935
C	4.366423	-2.221478	-1.105886
C	5.358973	-3.194904	-0.950759
H	5.882907	-3.577579	-1.827687
C	5.686632	-3.690248	0.314511
H	6.464023	-4.451926	0.411352
C	5.031651	-3.223809	1.452838
H	5.298827	-3.625090	2.432627
C	4.034710	-2.241228	1.357439
C	3.993794	-1.648025	-2.465859
H	2.951513	-1.295550	-2.397756
C	4.877728	-0.431105	-2.799907
H	4.570214	0.020432	-3.756916
H	5.933674	-0.732370	-2.890373
H	4.817222	0.338989	-2.015783
C	4.028771	-2.692526	-3.588478
H	3.423438	-3.574495	-3.329137
H	5.054504	-3.030130	-3.804906
H	3.628762	-2.260476	-4.518897
C	3.308991	-1.742760	2.601187
H	2.615011	-0.941884	2.303320
C	2.459729	-2.862525	3.227089
H	1.741886	-3.273508	2.499407
H	1.894182	-2.482028	4.092476
H	3.091643	-3.693630	3.578302
C	4.282097	-1.124703	3.617922
H	4.868240	-0.311381	3.162407
H	4.990930	-1.872432	4.007068
H	3.729118	-0.713153	4.477190
N	1.301631	-3.189346	-0.733327
I	-3.636027	1.923151	-0.212609

C	-4.412813	-0.074497	0.097173
C	-3.995251	-0.796745	1.218704
C	-5.308084	-0.603252	-0.826227
H	-3.286912	-0.376989	1.934382
C	-4.493765	-2.081146	1.408211
C	-5.807857	-1.896440	-0.629198
H	-5.625860	-0.034828	-1.702354
H	-4.196464	-2.674468	2.274983
C	-5.401688	-2.645511	0.488962
H	-6.509953	-2.302153	-1.356931
O	-5.808447	-3.898194	0.760322
C	-6.763823	-4.528315	-0.077932
H	-6.953815	-5.516745	0.358915
H	-6.380887	-4.655402	-1.106039
H	-7.709829	-3.960299	-0.110443
C	1.993785	2.735457	-2.410500
H	1.047287	2.184032	-2.541183
H	2.073173	3.471220	-3.221888
H	2.812172	2.006972	-2.530234
C	2.085956	2.335208	2.632321
H	2.986302	1.700295	2.670476
H	2.058156	2.938133	3.549733
H	1.212962	1.661020	2.640223
C	2.185309	6.915815	0.456471
H	3.237164	7.248891	0.486719
H	1.708907	7.414989	-0.400320
H	1.703309	7.273816	1.378441

80
TS-AB-cis+Mes SCF Done: -1813.37105888 A.U.

Au	1.022640	-1.346486	0.137151
N	-1.574691	-2.779081	-0.203811
N	-0.651732	1.269981	0.253287
N	-1.873270	1.770688	0.175516
N	-2.652901	0.736409	-0.023198
C	-2.018165	-4.023645	-0.371705
H	-1.273137	-4.824872	-0.320574
C	-3.371222	-4.295327	-0.600888
H	-3.735758	-5.314756	-0.736148
C	-4.228979	-3.193845	-0.648250
H	-5.302956	-3.316098	-0.825090
C	-2.480777	-1.791164	-0.266176
C	-1.938645	-0.436349	-0.072670
C	-0.602647	-0.087474	0.108913
C	0.451777	2.169899	0.489587
C	0.799529	2.455925	1.816680
C	1.846203	3.364756	2.019475
H	2.138254	3.610474	3.043777
C	2.520970	3.966311	0.949190

C	2.139392	3.631004	-0.359457
H	2.670341	4.082566	-1.201474
C	1.106861	2.726930	-0.620337
C	-4.080428	0.963917	-0.135115
C	-4.834704	0.963717	1.048560
C	-6.207785	1.210346	0.915050
H	-6.841012	1.230229	1.803590
C	-6.775871	1.434477	-0.339168
H	-7.848517	1.625841	-0.420333
C	-5.988334	1.419082	-1.492017
H	-6.454115	1.598676	-2.462107
C	-4.611006	1.178398	-1.417808
C	-4.192124	0.758500	2.414356
H	-3.205464	0.291400	2.261142
C	-3.951606	2.117558	3.097476
H	-3.437086	1.982938	4.062448
H	-4.907236	2.630394	3.291319
H	-3.340531	2.778796	2.463786
C	-4.994327	-0.194447	3.311894
H	-5.185345	-1.153578	2.806064
H	-5.965417	0.235752	3.602381
H	-4.439354	-0.398225	4.240918
C	-3.722729	1.200442	-2.654936
H	-2.812970	0.620705	-2.427163
C	-4.373078	0.533682	-3.874413
H	-4.718323	-0.484132	-3.636012
H	-3.649069	0.468301	-4.701472
H	-5.235701	1.108683	-4.245653
C	-3.281494	2.643422	-2.965880
H	-2.781777	3.104563	-2.100055
H	-4.152478	3.268708	-3.219183
H	-2.588388	2.663002	-3.822314
N	-3.787098	-1.944294	-0.483177
I	3.005310	-3.143002	0.263365
C	3.139492	-0.617766	-0.147748
C	3.644820	0.099772	0.941672
C	3.453204	-0.299114	-1.465677
H	3.424714	-0.195710	1.967634
C	4.421705	1.221136	0.683139
C	4.229047	0.840035	-1.712318
H	3.100363	-0.910404	-2.296923
H	4.810603	1.826885	1.502634
C	4.709607	1.609809	-0.640247
H	4.449056	1.106055	-2.745821
O	5.439494	2.734942	-0.775420
C	5.806457	3.187402	-2.068028
H	6.395689	4.101099	-1.920520
H	4.917942	3.425131	-2.679945
H	6.423155	2.440998	-2.597546

C	0.073758	1.804885	2.966239
H	-0.999609	2.053220	2.956779
H	0.152370	0.705930	2.913388
H	0.485737	2.129279	3.931128
C	0.712820	2.343304	-2.022915
H	0.856708	1.262169	-2.185678
H	-0.347195	2.566968	-2.223001
H	1.318232	2.880294	-2.765333
C	3.661458	4.923715	1.178143
H	3.491516	5.874222	0.647256
H	3.801684	5.149587	2.244740
H	4.597952	4.495179	0.785421

80

TS-AB-trans+Mes SCF Done: -1813.36758525 A.U.

Au	1.358965	-0.201412	-0.047896
N	-0.224175	-2.626876	-0.145066
N	-1.363921	1.350444	0.095309
N	-2.678903	1.211229	0.109519
N	-2.882482	-0.083024	0.044324
C	0.012439	-3.934200	-0.226091
H	1.062129	-4.244110	-0.267801
C	-1.030978	-4.865623	-0.257674
H	-0.839994	-5.937694	-0.324862
C	-2.328299	-4.350523	-0.198885
H	-3.202641	-5.009989	-0.217440
C	-1.508996	-2.231718	-0.095390
C	-1.698415	-0.777371	-0.011367
C	-0.681798	0.170293	0.021938
C	-0.773002	2.665521	0.152760
C	-0.457269	3.195289	1.412564
C	0.161836	4.449051	1.438279
H	0.421019	4.888716	2.405098
C	0.461754	5.150821	0.260638
C	0.137760	4.565971	-0.972186
H	0.378505	5.096114	-1.897392
C	-0.480726	3.313476	-1.055424
C	-4.245591	-0.573923	0.036609
C	-4.846842	-0.853641	1.274470
C	-6.170694	-1.310348	1.237684
H	-6.690346	-1.536126	2.170108
C	-6.836297	-1.478120	0.022578
H	-7.869226	-1.834292	0.017142
C	-6.198159	-1.193808	-1.185662
H	-6.739104	-1.329749	-2.123365
C	-4.875162	-0.733980	-1.208576
C	-4.116063	-0.618118	2.590180
H	-3.033278	-0.626616	2.382437
C	-4.463654	0.775236	3.147750

H	-3.894495	0.979211	4.068904
H	-5.536579	0.839419	3.389717
H	-4.238854	1.565675	2.415316
C	-4.373311	-1.721466	3.625538
H	-4.156452	-2.717962	3.210491
H	-5.416630	-1.718901	3.977727
H	-3.734277	-1.568174	4.509101
C	-4.174237	-0.372150	-2.511899
H	-3.086905	-0.405354	-2.332355
C	-4.462595	-1.366272	-3.645020
H	-4.243512	-2.400050	-3.335812
H	-3.842535	-1.129280	-4.523573
H	-5.513506	-1.323742	-3.971107
C	-4.526046	1.070948	-2.920509
H	-4.278407	1.784579	-2.119769
H	-5.604075	1.163040	-3.128328
H	-3.977991	1.362443	-3.830842
N	-2.568963	-3.039084	-0.118174
I	3.191694	1.847932	-0.258196
C	3.330330	-0.859800	-0.100817
C	3.799054	-1.407961	-1.315720
C	3.923123	-1.230650	1.120275
H	3.414443	-1.047918	-2.271895
C	4.731019	-2.431919	-1.283813
C	4.860910	-2.260652	1.152555
H	3.639405	-0.729007	2.047530
H	5.084085	-2.907730	-2.200718
C	5.270786	-2.872254	-0.051855
H	5.279829	-2.567619	2.110558
O	6.175583	-3.852922	-0.133042
C	6.832423	-4.326525	1.035066
H	7.524158	-5.109556	0.701183
H	6.113763	-4.758588	1.752692
H	7.404359	-3.521783	1.527351
C	-0.751181	2.422396	2.672701
H	-1.820031	2.167983	2.752598
H	-0.189093	1.472990	2.690750
H	-0.471804	2.997286	3.565651
C	-0.787398	2.661212	-2.378698
H	-0.187264	1.744316	-2.508528
H	-1.847220	2.371053	-2.454315
H	-0.558887	3.334242	-3.215737
C	1.097355	6.516508	0.323533
H	1.660839	6.746094	-0.592908
H	0.324788	7.296319	0.438217
H	1.779766	6.606254	1.182166

85

A-ad-cis+Ad SCF Done: -1853.90363019 A.U.

Au	-0.855177	-1.630165	-0.171291
N	2.090123	-3.071550	0.008043
N	0.687925	0.936947	0.216020
N	1.866141	1.504235	0.338413
N	2.735627	0.530678	0.211823
C	2.712317	-4.236758	-0.174973
H	2.120836	-5.143619	-0.009260
C	4.055670	-4.308667	-0.556667
H	4.561575	-5.265076	-0.697673
C	4.711556	-3.091764	-0.756204
H	5.760565	-3.053239	-1.068901
C	2.815219	-1.964167	-0.196829
C	2.118705	-0.680776	0.008569
C	0.750863	-0.420287	0.019128
C	4.142106	0.855705	0.325516
C	4.788395	1.356447	-0.816789
C	6.141595	1.688554	-0.679171
H	6.686548	2.089983	-1.535831
C	6.804155	1.508417	0.536124
H	7.861701	1.769761	0.620546
C	6.126798	1.000168	1.644429
H	6.660497	0.866818	2.587627
C	4.767889	0.664338	1.567984
C	4.064941	1.542458	-2.143583
H	3.072955	1.070848	-2.063242
C	3.835716	3.035347	-2.436134
H	3.266762	3.165005	-3.370537
H	4.793943	3.567640	-2.547074
H	3.275672	3.519235	-1.620874
C	4.794515	0.834884	-3.295960
H	4.947955	-0.230194	-3.064626
H	5.778687	1.287233	-3.495148
H	4.205450	0.908473	-4.223757
C	4.036102	0.104786	2.781565
H	2.970245	-0.001704	2.525360
C	4.559892	-1.296803	3.141686
H	4.478201	-1.987755	2.288597
H	3.989564	-1.719116	3.984049
H	5.620046	-1.258547	3.438775
C	4.109392	1.062234	3.982281
H	3.719226	2.058369	3.722064
H	5.144234	1.188063	4.337531
H	3.517398	0.668059	4.823393
N	4.093533	-1.922036	-0.581833
I	-3.273819	-2.898164	-0.314370
C	-4.299279	-1.035925	0.118942
C	-5.119818	-0.464818	-0.857591
C	-4.096305	-0.428387	1.354594
H	-5.277079	-0.946956	-1.823995

C	-5.734404	0.753451	-0.584368
C	-4.714529	0.798482	1.621721
H	-3.459420	-0.886141	2.113549
H	-6.375271	1.235139	-1.324854
C	-5.527081	1.405212	0.647196
H	-4.548067	1.268678	2.590277
O	-6.126073	2.600239	0.789646
C	-6.004567	3.311951	2.010763
H	-6.590622	4.231632	1.891002
H	-4.953576	3.578615	2.219345
H	-6.410460	2.732997	2.858378
C	-0.561855	1.731818	0.196419
H	-1.284498	1.065678	0.689086
C	-1.039410	1.975631	-1.254399
C	-0.519462	3.063391	0.972879
H	-0.998567	1.020951	-1.803871
C	-0.164831	3.037950	-1.944016
C	-2.497512	2.460747	-1.162940
H	-0.119122	2.880932	1.983634
C	0.307335	4.150804	0.253638
C	-1.990231	3.538487	1.059921
H	-0.515277	3.174786	-2.980145
H	0.882823	2.699129	-2.007191
C	-0.245678	4.368220	-1.167027
H	-3.119232	1.685154	-0.691785
H	-2.897410	2.618982	-2.177658
C	-2.556993	3.772545	-0.357425
H	1.369082	3.874839	0.216367
H	0.238151	5.085110	0.834779
H	-2.601634	2.791526	1.593545
H	-2.034597	4.471110	1.645427
H	0.360139	5.130310	-1.683233
C	-1.711817	4.839866	-1.081148
H	-3.605568	4.104357	-0.280392
H	-1.765540	5.799895	-0.541015
H	-2.116126	5.017689	-2.091829

85
A-ad-trans+Ad SCF Done: -1853.90143242 A.U.

Au	1.037867	-1.265403	-0.309908
N	1.329524	1.768109	0.310193
N	-1.985424	-0.755283	-0.458341
N	-2.915360	0.171315	-0.411967
N	-2.278369	1.303570	-0.238573
C	2.255152	2.713664	0.467420
H	3.268365	2.373434	0.703303
C	1.949753	4.072849	0.343559
H	2.707591	4.845643	0.481521
C	0.624883	4.387153	0.032619

H	0.297990	5.425626	-0.088865
C	0.087145	2.175133	0.014384
C	-0.918230	1.112218	-0.169364
C	-0.715260	-0.259392	-0.318747
C	-3.077136	2.496121	-0.031723
C	-3.346905	3.320160	-1.138194
C	-4.156620	4.436638	-0.904820
H	-4.402017	5.108214	-1.728554
C	-4.657722	4.706183	0.371714
H	-5.289898	5.583254	0.529784
C	-4.359530	3.867497	1.443678
H	-4.760928	4.092729	2.433783
C	-3.553603	2.732591	1.267050
C	-2.815215	2.979544	-2.523039
H	-1.883716	2.406696	-2.386151
C	-3.814157	2.076493	-3.271909
H	-3.412747	1.784453	-4.255531
H	-4.766061	2.606514	-3.436049
H	-4.035953	1.161121	-2.701805
C	-2.449310	4.217501	-3.351132
H	-1.767836	4.880355	-2.796154
H	-3.339301	4.800323	-3.636056
H	-1.951298	3.913335	-4.284800
C	-3.241768	1.810381	2.439511
H	-2.516376	1.052355	2.104668
C	-2.580435	2.569501	3.601183
H	-1.675275	3.100173	3.266276
H	-2.294193	1.869986	4.402400
H	-3.262679	3.313661	4.041192
C	-4.502888	1.055531	2.894025
H	-4.945093	0.484033	2.063582
H	-5.269438	1.752600	3.268055
H	-4.261451	0.351722	3.706308
N	-0.302787	3.443219	-0.138024
I	3.477241	-2.476524	-0.262180
C	4.579454	-0.659667	0.148259
C	4.670283	-0.203672	1.466764
C	5.181094	0.019402	-0.906276
H	4.193034	-0.740146	2.288510
C	5.385524	0.961752	1.723205
C	5.900360	1.191335	-0.642656
H	5.102373	-0.341502	-1.933367
H	5.483462	1.346643	2.739945
C	6.009442	1.669603	0.675329
H	6.371194	1.714318	-1.474508
O	6.668454	2.785835	1.031338
C	7.380252	3.529148	0.055214
H	7.854773	4.363025	0.587503
H	6.704905	3.933155	-0.719934

H	8.161954	2.917562	-0.427930
C	-2.335622	-2.191580	-0.591626
H	-1.589926	-2.564805	-1.309387
C	-3.749651	-2.464179	-1.147238
C	-2.144851	-2.937090	0.750858
H	-3.892096	-1.886069	-2.074739
C	-4.864740	-2.131238	-0.130692
C	-3.804343	-3.980346	-1.452039
H	-1.152451	-2.686784	1.159617
C	-3.251795	-2.543691	1.746361
C	-2.215214	-4.446182	0.444143
H	-5.834619	-2.410050	-0.574227
H	-4.905418	-1.053439	0.070596
C	-4.633860	-2.917481	1.172470
H	-3.040155	-4.252076	-2.200255
H	-4.783615	-4.222179	-1.895011
C	-3.592619	-4.788867	-0.153971
H	-3.210792	-1.462834	1.963691
H	-3.078135	-3.063960	2.702522
H	-1.407789	-4.728631	-0.253372
H	-2.049553	-5.014593	1.373773
H	-5.416074	-2.654878	1.903103
C	-4.685502	-4.428131	0.871995
H	-3.636975	-5.865564	-0.384135
H	-4.538263	-5.008814	1.798247
H	-5.679721	-4.698493	0.479108

85
TS-AB-cis+Ad SCF Done: -1853.87753790 A.U.

Au	-1.217615	-1.477943	-0.000939
N	1.184056	-3.049340	0.068991
N	0.624256	1.088467	-0.032097
N	1.889343	1.460500	-0.027447
N	2.584695	0.350695	0.003429
C	1.507066	-4.340658	0.097675
H	0.678690	-5.057258	0.110059
C	2.841466	-4.759858	0.111235
H	3.109671	-5.817120	0.134799
C	3.810941	-3.754182	0.092892
H	4.879435	-3.994296	0.101642
C	2.189461	-2.160210	0.052955
C	1.769646	-0.750565	0.019720
C	0.461272	-0.270703	-0.003444
C	4.029361	0.455891	0.015599
C	4.694255	0.492564	-1.220566
C	6.088190	0.627303	-1.179636
H	6.651688	0.665558	-2.114152
C	6.765075	0.716065	0.037281
H	7.852427	0.822657	0.045822

C	6.064695	0.672857	1.243314
H	6.610011	0.746077	2.186508
C	4.670116	0.539720	1.262178
C	3.958659	0.393499	-2.550390
H	2.888327	0.235581	-2.343695
C	4.072542	1.704302	-3.346878
H	3.504283	1.635403	-4.288059
H	5.120644	1.925377	-3.603830
H	3.681313	2.556519	-2.769267
C	4.439110	-0.818027	-3.366467
H	4.346998	-1.747790	-2.784026
H	5.493859	-0.710534	-3.664860
H	3.843538	-0.922912	-4.287069
C	3.909238	0.489172	2.580477
H	2.841200	0.335904	2.358954
C	4.362511	-0.702024	3.441045
H	4.270405	-1.648083	2.885452
H	3.750606	-0.772844	4.354181
H	5.413370	-0.596173	3.753484
C	4.022035	1.821913	3.339691
H	3.652487	2.660628	2.728989
H	5.066925	2.039547	3.612070
H	3.433884	1.787344	4.270490
N	3.488656	-2.457894	0.063748
I	-3.276043	-3.204697	-0.020434
C	-3.346275	-0.678411	-0.069026
C	-3.688797	-0.141958	-1.316208
C	-3.772164	-0.106159	1.127756
H	-3.397290	-0.635536	-2.243883
C	-4.396891	1.053883	-1.347483
C	-4.486629	1.096349	1.083287
H	-3.546205	-0.571175	2.087959
H	-4.662212	1.520294	-2.298094
C	-4.787439	1.694200	-0.153631
H	-4.795302	1.554053	2.022707
O	-5.414882	2.874437	-0.300263
C	-5.885709	3.573074	0.841645
H	-6.369832	4.483725	0.467665
H	-5.054981	3.854995	1.512121
H	-6.623716	2.975990	1.404522
C	-0.455190	2.102213	-0.060081
H	-1.362412	1.484153	-0.072977
C	-0.441206	2.983419	-1.331424
C	-0.484075	2.996668	1.201850
H	-0.365860	2.329356	-2.216425
C	0.713275	4.006606	-1.310869
C	-1.785349	3.745228	-1.351341
H	-0.439081	2.351788	2.095701
C	0.671641	4.018319	1.209036

C	-1.827972	3.757704	1.167507
H	0.650584	4.625664	-2.220957
H	1.689344	3.501741	-1.335930
C	0.599349	4.897527	-0.056540
H	-2.627729	3.038861	-1.396056
H	-1.836555	4.363633	-2.261913
C	-1.902498	4.635931	-0.098065
H	1.645412	3.511600	1.269905
H	0.581629	4.645581	2.111279
H	-2.665401	3.043669	1.187920
H	-1.912495	4.383355	2.071114
H	1.431157	5.620027	-0.046017
C	-0.744548	5.652252	-0.081894
H	-2.869630	5.164437	-0.119205
H	-0.827797	6.309003	0.800405
H	-0.798512	6.301698	-0.971598

85

TS-AB-trans+Ad SCF Done: -1853.87785290 A.U.

Au	1.547399	0.004739	-0.201530
N	0.553753	-2.588261	0.070416
N	-1.469463	1.003727	-0.338118
N	-2.709867	0.563013	-0.317549
N	-2.618440	-0.739368	-0.185570
C	1.093897	-3.798455	0.191230
H	2.187568	-3.848646	0.225020
C	0.297769	-4.946121	0.268124
H	0.734604	-5.940849	0.367891
C	-1.084707	-4.751304	0.208041
H	-1.779380	-5.596498	0.260055
C	-0.787969	-2.504838	0.019683
C	-1.307848	-1.139053	-0.122659
C	-0.535091	0.012284	-0.226521
C	-3.839648	-1.505269	-0.045222
C	-4.415801	-1.570907	1.233128
C	-5.615106	-2.289232	1.345162
H	-6.109072	-2.363184	2.316009
C	-6.184221	-2.907890	0.233411
H	-7.119745	-3.461857	0.341844
C	-5.570703	-2.827521	-1.019303
H	-6.034336	-3.321254	-1.874246
C	-4.374815	-2.121013	-1.190313
C	-3.796192	-0.890435	2.447625
H	-2.800430	-0.513965	2.164584
C	-4.631818	0.327041	2.879352
H	-4.154952	0.843483	3.727529
H	-5.642319	0.021452	3.194039
H	-4.740077	1.047849	2.054581
C	-3.580546	-1.876094	3.607116

H	-2.983294	-2.743633	3.285353
H	-4.535887	-2.253495	4.004061
H	-3.052852	-1.380504	4.437235
C	-3.709344	-1.974385	-2.552438
H	-2.628866	-1.834981	-2.382179
C	-3.857023	-3.218814	-3.436986
H	-3.527422	-4.127250	-2.909493
H	-3.248956	-3.108401	-4.348120
H	-4.898248	-3.370665	-3.761850
C	-4.231408	-0.712563	-3.266889
H	-4.076758	0.190192	-2.656102
H	-5.311641	-0.801221	-3.464635
H	-3.718484	-0.571570	-4.231688
N	-1.627877	-3.537188	0.082287
I	3.163312	2.153972	-0.883287
C	3.601895	-0.346894	-0.069131
C	4.177095	-1.130429	-1.094002
C	4.159029	-0.344843	1.222321
H	3.808887	-1.059749	-2.119292
C	5.196904	-2.014004	-0.780584
C	5.185693	-1.233774	1.536501
H	3.781439	0.336870	1.986878
H	5.639187	-2.662226	-1.539587
C	5.711774	-2.078549	0.536184
H	5.580725	-1.249802	2.551962
O	6.705396	-2.952541	0.727962
C	7.344214	-3.050237	1.993949
H	8.122152	-3.815952	1.886188
H	6.634110	-3.361907	2.779337
H	7.812868	-2.093702	2.281123
C	-1.154497	2.451377	-0.416661
H	-0.314381	2.486960	-1.126431
C	-0.662600	2.981734	0.951171
C	-2.303557	3.337047	-0.941803
H	0.123451	2.308642	1.330506
C	-1.835298	3.063274	1.946192
C	-0.070806	4.384421	0.709630
H	-2.677745	2.920058	-1.891040
C	-3.458168	3.476048	0.075538
C	-1.692229	4.738071	-1.180984
H	-1.458292	3.415007	2.920494
H	-2.269364	2.063272	2.118314
C	-2.915996	4.024215	1.408143
H	0.782054	4.317055	0.014428
H	0.321299	4.781332	1.660213
C	-1.160086	5.316536	0.147811
H	-3.964793	2.515221	0.230322
H	-4.208101	4.168418	-0.340774
H	-0.879684	4.682988	-1.925418

H	-2.465966	5.399927	-1.601738
H	-3.738749	4.093593	2.138194
C	-2.306968	5.420066	1.172649
H	-0.731415	6.314932	-0.035679
H	-3.084400	6.110886	0.806089
H	-1.927636	5.838188	2.120349

15
m-PhCF3I SCF Done: -579.691790345 A.U.

C	1.921425	1.684675	-0.021317
C	1.779685	0.293545	-0.035002
C	-0.616976	0.525292	-0.004165
C	-0.493750	1.917418	0.013032
C	0.782168	2.490385	0.003171
H	2.917922	2.129057	-0.037141
H	-1.379888	2.554325	0.030757
H	0.882004	3.578326	0.011518
I	-2.570439	-0.367111	0.000986
C	0.510630	-0.296652	-0.028779
H	0.418696	-1.382961	-0.050378
C	3.006625	-0.586710	-0.001593
F	2.783810	-1.778101	-0.582607
F	4.046021	-0.012074	-0.631726
F	3.398580	-0.830890	1.263321

88
m-CF3-A-ad+ SCF Done: -2153.60627401 A.U.

Au	0.664192	1.503801	0.199729
N	-2.261193	2.769538	1.104262
N	-0.915179	-0.980505	-0.359967
N	-2.097503	-1.557477	-0.436416
N	-2.954642	-0.614986	-0.128468
C	-2.872028	3.909373	1.429956
H	-2.260260	4.675118	1.918895
C	-4.228671	4.125684	1.168086
H	-4.724688	5.057391	1.444598
C	-4.911457	3.089823	0.526620
H	-5.972737	3.177970	0.270442
C	-3.012377	1.836075	0.505348
C	-2.328897	0.578274	0.152849
C	-0.964744	0.339014	0.004358
C	0.272772	-1.756959	-0.632910
C	0.804005	-1.722679	-1.933499
C	1.957767	-2.486042	-2.161122
H	2.408136	-2.493928	-3.155736
C	2.536070	-3.233954	-1.136977
H	3.435500	-3.820793	-1.331259
C	1.986420	-3.229388	0.146201
H	2.467199	-3.808905	0.934249

C	0.837556	-2.482010	0.433796
C	0.190698	-0.892126	-3.053243
H	-0.720657	-0.409772	-2.668148
C	-0.233383	-1.767823	-4.243557
H	-0.731428	-1.154993	-5.011365
H	-0.933027	-2.556709	-3.929145
H	0.634090	-2.253798	-4.717376
C	1.149003	0.230858	-3.486652
H	2.079657	-0.180298	-3.909382
H	1.422057	0.870194	-2.631881
H	0.680639	0.864951	-4.255845
C	0.205305	-2.489784	1.819767
H	-0.419235	-1.586051	1.909477
C	1.241089	-2.418443	2.949964
H	1.920166	-1.562213	2.820366
H	1.858681	-3.327882	3.003949
H	0.731887	-2.312113	3.920280
C	-0.721711	-3.709461	1.976917
H	-1.502433	-3.720850	1.201422
H	-1.214519	-3.697018	2.961449
H	-0.147445	-4.645999	1.894261
C	-4.359738	-0.967224	-0.064855
C	-5.121276	-0.835323	-1.238796
C	-6.469012	-1.200901	-1.154225
H	-7.106510	-1.124766	-2.036129
C	-7.013227	-1.657688	0.048723
H	-8.069507	-1.933601	0.093305
C	-6.223211	-1.765880	1.192242
H	-6.668995	-2.122260	2.122538
C	-4.862541	-1.426075	1.162962
C	-4.493096	-0.348734	-2.537617
H	-3.624468	0.275323	-2.270145
C	-3.973600	-1.539992	-3.364799
H	-3.465982	-1.187840	-4.276977
H	-4.807281	-2.192860	-3.668987
H	-3.262689	-2.150820	-2.788176
C	-5.431144	0.541847	-3.362134
H	-5.825447	1.371636	-2.755977
H	-6.284318	-0.023751	-3.768331
H	-4.889266	0.968161	-4.220676
C	-4.004281	-1.506163	2.420168
H	-2.946664	-1.443418	2.119626
C	-4.292849	-0.309741	3.345547
H	-4.130897	0.649491	2.830419
H	-3.640563	-0.337813	4.232769
H	-5.338700	-0.328075	3.691480
C	-4.169623	-2.840405	3.162707
H	-3.973560	-3.695730	2.498877
H	-5.183141	-2.955530	3.577196

H	-3.465356	-2.893060	4.007667
N	-4.305156	1.949608	0.190868
I	3.034011	2.853520	0.326860
C	4.117296	1.102333	-0.354587
C	4.568893	1.058571	-1.671725
H	4.393544	1.884772	-2.362598
C	5.255289	-0.085486	-2.096522
H	5.618307	-0.140059	-3.125142
C	5.473643	-1.147880	-1.218852
H	6.004127	-2.040722	-1.554126
C	4.315028	0.058211	0.544799
H	3.942446	0.100009	1.568324
C	5.002953	-1.073679	0.095938
C	5.283332	-2.204330	1.061885
F	5.250028	-3.398775	0.442803
F	6.493699	-2.076876	1.619771
F	4.380028	-2.238217	2.059775

88
m-CF3-B+ SCF Done: -2153.61425297 A.U.

Au	-0.841804	1.515061	-0.003781
N	1.138491	2.603537	0.405036
N	0.651932	-1.351241	-0.297626
N	1.905166	-1.782570	-0.260236
N	2.640808	-0.720146	-0.044323
C	1.340613	3.901618	0.653179
H	0.448357	4.531911	0.714077
C	2.635208	4.398613	0.819818
H	2.808889	5.455632	1.025369
C	3.686564	3.486314	0.708542
H	4.728308	3.802984	0.822211
C	2.224570	1.792375	0.319609
C	1.866192	0.400222	0.057300
C	0.550067	-0.006608	-0.113886
C	-0.419140	-2.315462	-0.449380
C	-0.848797	-2.987037	0.709487
C	-1.903482	-3.895255	0.547411
H	-2.290539	-4.434480	1.412085
C	-2.483329	-4.100978	-0.702964
H	-3.319274	-4.796140	-0.799671
C	-2.019460	-3.418570	-1.829373
H	-2.491530	-3.601506	-2.794469
C	-0.962825	-2.505221	-1.734548
C	-0.162999	-2.793000	2.057893
H	0.328451	-1.804726	2.050872
C	0.935116	-3.858464	2.243389
H	1.461294	-3.708866	3.198495
H	1.675703	-3.824267	1.430838
H	0.491550	-4.866578	2.253881

C	-1.129729	-2.792129	3.249965
H	-1.597707	-3.777361	3.397998
H	-1.937548	-2.057688	3.128512
H	-0.580820	-2.553533	4.173927
C	-0.381063	-1.799462	-2.955586
H	-0.117704	-0.772184	-2.647062
C	-1.362094	-1.677779	-4.127502
H	-2.318853	-1.238514	-3.814192
H	-1.568234	-2.657756	-4.585672
H	-0.929547	-1.039900	-4.913373
C	0.911839	-2.502351	-3.419974
H	1.658184	-2.584898	-2.617523
H	1.369177	-1.953867	-4.258391
H	0.685964	-3.523425	-3.765564
C	4.082457	-0.844796	0.032989
C	4.654485	-1.108546	1.290325
C	6.052346	-1.192195	1.332036
H	6.550191	-1.390674	2.282800
C	6.817681	-1.026739	0.176891
H	7.906347	-1.097743	0.235017
C	6.206216	-0.778016	-1.052020
H	6.821294	-0.658255	-1.946042
C	4.812527	-0.679883	-1.156803
C	3.822142	-1.254787	2.558512
H	2.765837	-1.365919	2.267056
C	4.192571	-2.513875	3.357083
H	3.517168	-2.627656	4.219151
H	5.218284	-2.458230	3.753128
H	4.114539	-3.419804	2.737833
C	3.931935	0.012090	3.426467
H	3.642617	0.912414	2.863529
H	4.966986	0.158972	3.774000
H	3.285182	-0.068389	4.314391
C	4.151309	-0.388405	-2.498342
H	3.058640	-0.432099	-2.366501
C	4.495263	1.031663	-2.981198
H	4.223360	1.786138	-2.226930
H	3.961237	1.262334	-3.916501
H	5.574274	1.132645	-3.178251
C	4.508841	-1.449370	-3.551581
H	4.242996	-2.460504	-3.207832
H	5.585308	-1.442322	-3.783079
H	3.967298	-1.252852	-4.490035
C	-2.503750	0.431677	-0.499952
C	-3.081946	-0.417276	0.441498
H	-2.666482	-0.528980	1.441949
C	-4.223661	-1.141511	0.081506
C	-4.189712	-0.149000	-2.118666
H	-4.623552	-0.031594	-3.114288

C	-3.045171	0.582554	-1.776389
H	-2.604441	1.268974	-2.501029
I	-2.392627	3.669097	0.256251
N	3.479742	2.187523	0.458461
C	-4.778584	-1.011821	-1.196211
H	-5.668882	-1.584304	-1.463115
C	-4.906972	-2.045751	1.080621
F	-5.206335	-3.239851	0.533550
F	-6.056198	-1.512119	1.516386
F	-4.136903	-2.282000	2.161147

88

m-CF3-TS-AB+ SCF Done: -2153.57718336 A.U.

Au	-1.071541	-1.262155	-0.086277
N	1.179356	-2.382438	-1.723412
N	0.973274	0.810953	0.961711
N	2.233008	1.202460	0.913000
N	2.795476	0.432968	0.012883
C	1.397727	-3.363175	-2.598480
H	0.569123	-4.055229	-2.782436
C	2.627766	-3.506264	-3.249092
H	2.807877	-4.311831	-3.962550
C	3.610977	-2.564270	-2.937925
H	4.601697	-2.599539	-3.403364
C	2.189732	-1.529051	-1.495284
C	1.901733	-0.467911	-0.515103
C	0.683831	-0.219244	0.111862
C	0.059984	1.467970	1.869160
C	-0.486168	2.703395	1.469779
C	-1.367570	3.319424	2.367287
H	-1.812128	4.281576	2.109604
C	-1.683944	2.721699	3.588581
H	-2.370130	3.223974	4.275037
C	-1.127941	1.493433	3.943430
H	-1.380202	1.045353	4.906746
C	-0.230770	0.833327	3.090255
C	-0.093141	3.369261	0.156542
H	0.259337	2.579226	-0.527726
C	1.075653	4.346874	0.384771
H	1.385565	4.804035	-0.567353
H	1.947154	3.840337	0.824214
H	0.772006	5.156069	1.067995
C	-1.267939	4.065900	-0.541451
H	-1.615021	4.947383	0.020128
H	-2.119490	3.386925	-0.677699
H	-0.959752	4.419619	-1.537030
C	0.410614	-0.482231	3.516963
H	1.084764	-0.817167	2.714247
C	-0.637136	-1.589657	3.716903

H	-1.210122	-1.774254	2.794343
H	-1.350955	-1.331906	4.515342
H	-0.149191	-2.535035	4.000702
C	1.272809	-0.289665	4.776924
H	2.032569	0.491643	4.625751
H	1.791354	-1.227061	5.032518
H	0.658874	-0.001192	5.644530
C	4.208015	0.613386	-0.261458
C	4.577021	1.567253	-1.222548
C	5.950833	1.710233	-1.464038
H	6.293317	2.433262	-2.206531
C	6.884868	0.940743	-0.771996
H	7.950435	1.071001	-0.975346
C	6.473125	0.006863	0.181428
H	7.222316	-0.579343	0.715134
C	5.114803	-0.184040	0.459850
C	3.555123	2.375528	-2.012331
H	2.574009	2.260363	-1.525406
C	3.873353	3.878353	-2.013674
H	3.068113	4.435825	-2.517302
H	4.807188	4.096781	-2.554591
H	3.973565	4.268976	-0.990028
C	3.426088	1.825585	-3.444441
H	3.175939	0.754241	-3.439708
H	4.373917	1.942770	-3.993931
H	2.643957	2.367591	-3.999494
C	4.642023	-1.165183	1.526173
H	3.616257	-1.477027	1.267345
C	5.484635	-2.445922	1.587021
H	5.568263	-2.919662	0.596702
H	5.023694	-3.168737	2.277897
H	6.502561	-2.250152	1.958430
C	4.576530	-0.469890	2.899700
H	3.933425	0.422541	2.868882
H	5.581082	-0.148269	3.217412
H	4.179446	-1.155760	3.664731
N	3.393355	-1.576532	-2.065563
I	-3.216905	-2.862354	-0.139774
C	-3.122668	-0.325380	0.087679
C	-3.416959	0.160796	1.361888
C	-4.206019	1.528067	-0.960339
C	-4.104954	1.373991	1.450436
H	-3.111245	-0.373527	2.260593
H	-4.330783	1.781665	2.437146
C	-4.502376	2.058089	0.297989
H	-5.051863	2.997290	0.372706
C	-3.523040	0.311419	-1.083927
H	-3.328383	-0.114732	-2.067981
C	-4.577109	2.271489	-2.226032

F	-3.511097	2.942030	-2.710899
F	-4.980410	1.427316	-3.186993
F	-5.550617	3.162643	-2.011566

103
m-CF3-TS-CD+ SCF Done: -3547.14735707 A.U.

Au	1.373836	0.228388	0.124261
N	0.849714	-1.461940	-1.128986
N	-1.808939	0.736476	0.945120
N	-2.928203	0.030757	0.804159
N	-2.579708	-1.038350	0.129696
C	1.650668	-2.098604	-1.988599
H	2.676264	-1.736951	-2.073462
C	1.172462	-3.179076	-2.727531
H	1.815291	-3.696069	-3.440469
C	-0.143529	-3.580897	-2.488165
H	-0.572426	-4.450477	-2.996477
C	-0.447698	-1.872710	-1.022272
C	-1.249859	-1.014922	-0.164934
C	-0.723423	0.144264	0.384339
C	-1.860039	2.022939	1.602371
C	-1.658989	2.059932	2.996144
C	-1.650026	3.327393	3.595553
H	-1.496229	3.417807	4.671426
C	-1.848428	4.479590	2.832469
H	-1.837761	5.457409	3.320069
C	-2.073505	4.397743	1.457317
H	-2.238713	5.312020	0.886971
C	-2.090128	3.159125	0.798449
C	-1.540523	0.779685	3.818031
H	-1.072210	0.010733	3.178576
C	-2.943972	0.263875	4.197001
H	-2.869184	-0.685780	4.748900
H	-3.568505	0.097062	3.307989
H	-3.458537	0.994788	4.840520
C	-0.665382	0.926106	5.070434
H	-1.142990	1.566805	5.827593
H	0.322217	1.358757	4.844491
H	-0.507830	-0.058395	5.536693
C	-2.406118	3.033123	-0.686851
H	-1.828160	2.188426	-1.096887
C	-2.033501	4.273642	-1.507740
H	-0.981697	4.569049	-1.387358
H	-2.667318	5.139383	-1.257226
H	-2.206783	4.062701	-2.575304
C	-3.900462	2.704078	-0.883221
H	-4.205807	1.809863	-0.323494
H	-4.103864	2.520097	-1.948802
H	-4.530981	3.543704	-0.548421

C	-3.544907	-2.099923	-0.082308
C	-3.620468	-3.095113	0.912684
C	-4.540845	-4.127323	0.696495
H	-4.636776	-4.927368	1.432560
C	-5.340761	-4.146297	-0.448095
H	-6.054728	-4.960362	-0.594723
C	-5.239608	-3.134769	-1.401264
H	-5.875813	-3.162307	-2.288031
C	-4.330620	-2.077569	-1.245150
C	-2.718138	-3.092545	2.142636
H	-2.291127	-2.083702	2.255564
C	-3.483708	-3.385737	3.441299
H	-2.813680	-3.277541	4.308836
H	-3.876885	-4.413804	3.462431
H	-4.330146	-2.694967	3.572153
C	-1.544948	-4.071280	1.950627
H	-0.977930	-3.846772	1.034212
H	-1.912638	-5.105959	1.862315
H	-0.855091	-4.028503	2.808921
C	-4.220562	-0.984957	-2.296780
H	-3.425820	-0.285246	-2.002157
C	-3.794897	-1.561950	-3.656199
H	-2.860817	-2.132978	-3.560682
H	-3.603503	-0.741776	-4.363273
H	-4.571435	-2.219603	-4.080243
C	-5.531034	-0.188423	-2.405602
H	-5.821298	0.247751	-1.437484
H	-6.362594	-0.823152	-2.752007
H	-5.415081	0.632922	-3.129909
C	3.390896	-0.149610	-0.193244
C	4.139617	0.582623	-1.123112
H	3.684779	1.443471	-1.617423
C	5.462512	0.206087	-1.388750
H	6.051902	0.778064	-2.109786
C	5.282236	-1.608416	0.193841
C	3.959531	-1.236598	0.474491
H	3.392958	-1.814557	1.209123
N	-0.939979	-2.933863	-1.634810
O	1.777057	2.498215	-1.236809
O	1.362036	4.506270	-2.129533
C	1.648961	3.768756	-1.172948
C	1.906894	4.420669	0.191876
H	1.347106	5.363883	0.250039
H	2.977829	4.689505	0.222935
C	1.555712	3.510980	1.362842
C	2.315932	2.242168	1.437878
H	3.344384	2.251549	1.060289
C	1.885031	1.109661	2.123374
H	2.625175	0.381807	2.468742

H	0.942836	1.155587	2.672453
H	1.815598	4.021499	2.315007
H	0.476114	3.316049	1.425166
K	0.634404	2.621647	-3.798113
Cl	-0.962261	0.376858	-2.975273
C	6.036735	-0.889264	-0.736290
H	7.067766	-1.182319	-0.940649
C	5.844204	-2.836034	0.872071
F	5.371450	-3.958357	0.293067
F	5.483293	-2.884106	2.167978
F	7.179814	-2.881162	0.811509

88

m-CF3-TS-decomp+ SCF Done: -2153.57753423 A.U.

Au	-2.039434	0.014144	-0.587410
N	0.255750	0.033758	-2.808502
N	0.299747	-0.437406	1.472132
N	1.491215	-0.996250	1.502579
N	1.860028	-1.119012	0.253572
C	0.298221	-0.112468	-4.135078
H	-0.309840	0.577820	-4.728300
C	1.082749	-1.100440	-4.736310
H	1.121196	-1.218674	-5.820245
C	1.807124	-1.935418	-3.879905
H	2.437406	-2.741887	-4.268849
C	0.992434	-0.812585	-2.082625
C	0.907876	-0.662966	-0.617065
C	-0.125127	-0.166376	0.189326
C	-0.377609	-0.146370	2.715034
C	0.118265	0.914765	3.504260
C	-0.609910	1.218437	4.662148
H	-0.277498	2.031160	5.308704
C	-1.757016	0.499918	5.001603
H	-2.312301	0.764291	5.904652
C	-2.189938	-0.567723	4.214185
H	-3.067566	-1.137767	4.520110
C	-1.499183	-0.932521	3.050051
C	1.403791	1.664264	3.165888
H	1.541599	1.626881	2.071675
C	2.611159	0.967100	3.827308
H	3.539705	1.510554	3.593130
H	2.722362	-0.072502	3.492187
H	2.492453	0.962633	4.922392
C	1.377335	3.146786	3.565741
H	1.434353	3.274718	4.657802
H	0.472294	3.654537	3.207791
H	2.249610	3.661834	3.135206
C	-1.864571	-2.198925	2.281923
H	-1.547868	-2.079652	1.235074

C	-3.369051	-2.489928	2.249998
H	-3.940425	-1.625205	1.880482
H	-3.756109	-2.767094	3.242700
H	-3.571170	-3.334476	1.574601
C	-1.065724	-3.387198	2.854734
H	0.019457	-3.205580	2.812737
H	-1.282956	-4.304627	2.285261
H	-1.335249	-3.566734	3.907559
C	3.220757	-1.529029	-0.030245
C	4.138566	-0.513041	-0.357021
C	5.457266	-0.925749	-0.592428
H	6.213576	-0.181695	-0.850037
C	5.818259	-2.270239	-0.496708
H	6.854560	-2.565188	-0.677610
C	4.869432	-3.242843	-0.175672
H	5.171325	-4.289749	-0.111510
C	3.533506	-2.894491	0.057349
C	3.753444	0.958473	-0.484959
H	2.707757	1.082214	-0.161232
C	4.593075	1.863647	0.430615
H	4.218331	2.897379	0.377274
H	5.652123	1.875947	0.129906
H	4.543279	1.527279	1.477003
C	3.829645	1.421684	-1.951682
H	3.210890	0.792737	-2.610082
H	4.864259	1.370933	-2.325604
H	3.486053	2.462280	-2.044639
C	2.483353	-3.943609	0.393699
H	1.491494	-3.471680	0.305193
C	2.500100	-5.114730	-0.599920
H	2.394651	-4.752974	-1.633940
H	1.668075	-5.804069	-0.387561
H	3.432107	-5.697158	-0.531727
C	2.638323	-4.421052	1.848134
H	2.576102	-3.578017	2.553539
H	3.612458	-4.913603	1.997516
H	1.849361	-5.145598	2.103910
C	-0.932719	1.716885	0.077049
C	-0.102220	2.438666	-0.780729
H	0.362802	1.953894	-1.637846
C	0.104858	3.794766	-0.522489
C	-1.392968	3.689570	1.372030
H	-1.915715	4.177930	2.196963
C	-1.562169	2.314652	1.179051
H	-2.194614	1.742730	1.857745
I	-4.193402	-1.013830	-1.602491
N	1.758679	-1.796731	-2.552980
C	-0.551078	4.429309	0.538075
H	-0.396367	5.495566	0.711737

C 1.122984 4.537014 -1.357292
F 2.362242 4.366985 -0.837147
F 1.165815 4.071467 -2.616667
F 0.882642 5.848914 -1.398666

15
m-PhMeI SCF Done: -282.192807625 A.U.
C -2.981619 0.823276 -0.000085
C -2.555234 -0.512019 -0.000161
C -0.260008 0.276767 -0.000003
C -0.682918 1.608614 0.000070
C -2.055573 1.870179 0.000033
H -4.051803 1.046584 -0.000137
H 0.037566 2.428044 0.000144
H -2.402719 2.906736 0.000059
I 1.850828 -0.159607 0.000005
C -1.174032 -0.776135 -0.000119
H -0.824642 -1.811314 -0.000211
C -3.536921 -1.658754 0.000113
H -3.396296 -2.302038 -0.884587
H -3.400482 -2.297894 0.888498
H -4.577671 -1.302487 -0.003104

88
m-Me-A-ad+ SCF Done: -1856.10752949 A.U.
Au 1.122061 1.294724 0.121264
N -1.684559 2.953239 0.712835
N -0.690009 -1.070327 -0.172273
N -1.923219 -1.533078 -0.246638
N -2.687825 -0.476665 -0.118810
C -2.181173 4.180299 0.872923
H -1.504950 4.936243 1.286445
C -3.501158 4.496621 0.536085
H -3.904608 5.500521 0.677279
C -4.271230 3.461616 0.001202
H -5.310693 3.622206 -0.304460
C -2.512531 2.030915 0.204820
C -1.953106 0.676087 0.042851
C -0.615813 0.285145 0.013183
C 0.416980 -1.996372 -0.253184
C 1.080489 -2.134289 -1.487374
C 2.130235 -3.060067 -1.534301
H 2.679281 -3.207392 -2.464788
C 2.483392 -3.801944 -0.406438
H 3.298520 -4.526337 -0.471263
C 1.816051 -3.619759 0.803591
H 2.115846 -4.201330 1.676560
C 0.769639 -2.693836 0.915144
C 0.644489 -1.354332 -2.721156

H	0.179378	-0.417625	-2.377186
C	-0.425840	-2.141907	-3.499802
H	-0.789128	-1.557696	-4.360266
H	-1.288914	-2.385440	-2.862285
H	-0.012213	-3.089590	-3.880186
C	1.819386	-0.949770	-3.621134
H	2.275763	-1.818876	-4.120458
H	2.605256	-0.436851	-3.044394
H	1.470385	-0.269339	-4.413102
C	0.041628	-2.480245	2.235947
H	-0.515778	-1.532547	2.166465
C	1.010670	-2.328335	3.418126
H	1.747163	-1.530690	3.230348
H	1.560657	-3.260150	3.623387
H	0.455824	-2.071220	4.333695
C	-0.983524	-3.601974	2.478433
H	-1.712692	-3.655133	1.655912
H	-1.533517	-3.428816	3.416412
H	-0.482117	-4.580310	2.552139
C	-4.123813	-0.671076	-0.103307
C	-4.798526	-0.705637	-1.334209
C	-6.185399	-0.895211	-1.284371
H	-6.755575	-0.932019	-2.214861
C	-6.848178	-1.032670	-0.064297
H	-7.931391	-1.174851	-0.048904
C	-6.138384	-0.996985	1.136281
H	-6.672786	-1.112352	2.081220
C	-4.748073	-0.821353	1.146996
C	-4.083652	-0.536507	-2.667916
H	-3.011375	-0.383523	-2.469438
C	-4.204372	-1.804761	-3.528786
H	-3.628995	-1.693331	-4.461238
H	-5.252384	-2.002721	-3.804260
H	-3.824690	-2.688811	-2.993705
C	-4.584208	0.711575	-3.413887
H	-4.479524	1.609059	-2.786374
H	-5.644971	0.612954	-3.694235
H	-4.008579	0.861587	-4.341133
C	-3.978724	-0.769943	2.461905
H	-2.901854	-0.801622	2.233553
C	-4.252284	0.550062	3.204896
H	-4.003418	1.423419	2.583178
H	-3.657781	0.605336	4.130573
H	-5.315897	0.630973	3.480490
C	-4.274819	-1.986795	3.352714
H	-4.085219	-2.929433	2.818014
H	-5.320973	-1.995326	3.696109
H	-3.636216	-1.965697	4.249948
N	-3.778428	2.233254	-0.169184

I	3.619810	2.378815	0.149713
C	4.512784	0.409147	-0.107029
C	5.097575	0.097227	-1.330507
H	5.124893	0.809203	-2.156540
C	5.653513	-1.180919	-1.466412
H	6.121115	-1.459853	-2.413722
C	5.612897	-2.091766	-0.409916
H	6.052360	-3.084050	-0.539339
C	4.453455	-0.481047	0.961530
H	3.974390	-0.202412	1.901900
C	5.016598	-1.760211	0.816957
C	5.000033	-2.729794	1.970756
H	5.011054	-3.771758	1.619116
H	5.888887	-2.587570	2.608761
H	4.110994	-2.589269	2.602559

88

m-Me-B+ SCF Done: -1856.11354305 A.U.

Au	1.376527	-1.117671	-0.160741
N	-0.360096	-2.638067	-0.076770
N	-0.659759	1.402883	0.035993
N	-1.973813	1.563587	0.129046
N	-2.481595	0.356162	0.121361
C	-0.306093	-3.973081	-0.102021
H	0.690467	-4.419341	-0.175382
C	-1.477339	-4.731516	-0.036417
H	-1.443534	-5.821534	-0.056898
C	-2.682934	-4.032871	0.056758
H	-3.641298	-4.558958	0.113928
C	-1.579071	-2.048747	0.012767
C	-1.499554	-0.588848	0.031615
C	-0.290955	0.093237	-0.028101
C	0.176375	2.587625	0.002593
C	0.814073	2.979846	1.195849
C	1.603368	4.134025	1.127426
H	2.128688	4.482667	2.016466
C	1.725570	4.852103	-0.063284
H	2.344141	5.752208	-0.088611
C	1.073139	4.431335	-1.220251
H	1.187466	5.006042	-2.140181
C	0.283700	3.273692	-1.219722
C	0.597836	2.223316	2.502517
H	0.549705	1.148549	2.256717
C	-0.747041	2.627090	3.141071
H	-0.944117	2.024738	4.041829
H	-1.593753	2.500540	2.452020
H	-0.723550	3.686919	3.439975
C	1.737302	2.395713	3.513658
H	1.768924	3.418764	3.920023

H	2.715053	2.174035	3.065805
H	1.587023	1.715870	4.366219
C	-0.444982	2.811927	-2.476407
H	-0.649903	1.732554	-2.369837
C	0.391394	2.978778	-3.753783
H	1.387677	2.522763	-3.653760
H	0.530718	4.039195	-4.014742
H	-0.120489	2.502949	-4.604368
C	-1.798601	3.535401	-2.610937
H	-2.435968	3.370155	-1.730216
H	-2.339682	3.181497	-3.501907
H	-1.643688	4.620896	-2.716166
C	-3.917510	0.179283	0.204618
C	-4.482254	0.010414	1.480110
C	-5.869543	-0.181005	1.528242
H	-6.359274	-0.315139	2.494791
C	-6.632032	-0.201724	0.360579
H	-7.712136	-0.354143	0.422056
C	-6.029666	-0.023973	-0.885836
H	-6.644967	-0.040767	-1.786992
C	-4.647715	0.175844	-0.997785
C	-3.654013	0.017619	2.758690
H	-2.604778	0.222385	2.493989
C	-4.096499	1.140625	3.710816
H	-3.444354	1.169491	4.597595
H	-5.128725	0.986674	4.062131
H	-4.050195	2.124186	3.219051
C	-3.688048	-1.358057	3.446753
H	-3.359716	-2.154192	2.761143
H	-4.705627	-1.608565	3.785801
H	-3.031055	-1.363778	4.330771
C	-3.983194	0.326593	-2.361233
H	-2.963958	0.714305	-2.206876
C	-3.857822	-1.043776	-3.052156
H	-3.315859	-1.765101	-2.421988
H	-3.325564	-0.948198	-4.011762
H	-4.853307	-1.468838	-3.257208
C	-4.706536	1.341297	-3.259159
H	-4.805830	2.318141	-2.762927
H	-5.713991	0.995427	-3.537462
H	-4.143966	1.486617	-4.194433
C	2.774896	0.373466	-0.237186
C	3.553436	0.637802	0.888396
H	3.450740	0.037608	1.794142
C	4.501273	1.674609	0.849154
C	3.859326	2.123492	-1.457565
H	3.979941	2.704941	-2.374918
C	2.912524	1.091829	-1.420110
H	2.306910	0.868329	-2.299395

I	3.322135	-2.929413	-0.337055
N	-2.732305	-2.695340	0.081461
C	4.638549	2.411520	-0.338262
H	5.373325	3.219638	-0.384959
C	5.349430	1.996489	2.054054
H	6.418420	2.023848	1.790016
H	5.092005	2.989593	2.459789
H	5.217549	1.259133	2.858856

88

m-Me-TS-AB+ SCF Done: -1856.08190941 A.U.

Au	-1.421166	-1.023576	-0.027750
N	0.874039	-2.821370	-0.881959
N	0.737310	1.116286	0.528757
N	2.030644	1.380075	0.521866
N	2.594749	0.305099	0.027343
C	1.082678	-4.058107	-1.331036
H	0.206585	-4.711319	-1.404042
C	2.359098	-4.504703	-1.689120
H	2.530170	-5.518983	-2.052984
C	3.400905	-3.583904	-1.556942
H	4.431345	-3.847736	-1.817911
C	1.942264	-2.014999	-0.791364
C	1.666019	-0.661506	-0.279256
C	0.423200	-0.125047	0.050729
C	-0.183888	2.114915	1.020810
C	-0.574012	3.137432	0.135137
C	-1.461795	4.097627	0.635689
H	-1.790299	4.915205	-0.007348
C	-1.933922	4.023583	1.947386
H	-2.623798	4.785923	2.317671
C	-1.532282	2.987978	2.789760
H	-1.909432	2.948487	3.813741
C	-0.636198	2.003414	2.347173
C	-0.014967	3.226877	-1.279559
H	0.319836	2.217691	-1.571735
C	1.214259	4.154344	-1.311491
H	1.643218	4.192657	-2.324665
H	1.996244	3.809701	-0.619174
H	0.931601	5.179033	-1.021796
C	-1.065169	3.656721	-2.312383
H	-1.375709	4.703891	-2.171531
H	-1.961683	3.023872	-2.252576
H	-0.648508	3.578025	-3.328518
C	-0.175667	0.897726	3.289912
H	0.554905	0.269940	2.757392
C	-1.342480	-0.015779	3.701262
H	-1.812289	-0.488381	2.824327
H	-2.120394	0.544888	4.243698

H	-0.987774	-0.819945	4.364605
C	0.543914	1.479035	4.519103
H	1.386547	2.121545	4.222804
H	0.937715	0.667104	5.150427
H	-0.138742	2.080558	5.139600
C	4.039667	0.283766	-0.092558
C	4.612815	0.803993	-1.263098
C	6.012162	0.765559	-1.343642
H	6.509872	1.150954	-2.235381
C	6.776409	0.240124	-0.302820
H	7.865661	0.222725	-0.386507
C	6.164184	-0.261887	0.847728
H	6.782572	-0.662784	1.651917
C	4.770884	-0.255960	0.980772
C	3.776890	1.336666	-2.420226
H	2.739928	1.457510	-2.069338
C	4.247417	2.720802	-2.892013
H	3.570608	3.106750	-3.670408
H	5.256963	2.680882	-3.329743
H	4.264516	3.443725	-2.062762
C	3.755906	0.323138	-3.578756
H	3.396084	-0.660445	-3.241876
H	4.767303	0.182904	-3.992823
H	3.103416	0.677045	-4.392867
C	4.080961	-0.760043	2.242886
H	3.060654	-1.074761	1.967049
C	4.764928	-1.988636	2.856748
H	4.899777	-2.787704	2.111455
H	4.155653	-2.386413	3.683078
H	5.753135	-1.742150	3.275332
C	3.946324	0.380519	3.270034
H	3.411850	1.243512	2.845239
H	4.940422	0.727705	3.593748
H	3.396821	0.038690	4.161396
N	3.194708	-2.341855	-1.111695
I	-3.692396	-2.423098	0.150652
C	-3.371458	0.068639	-0.422196
C	-3.732918	0.937677	0.601217
C	-4.202316	1.530460	-2.142988
C	-4.318734	2.149253	0.216198
H	-3.559902	0.702455	1.650165
H	-4.596017	2.867285	0.990527
C	-4.554415	2.437247	-1.128867
H	-5.031464	3.382147	-1.400793
C	-3.608146	0.310597	-1.774144
H	-3.349958	-0.428350	-2.533464
C	-4.445585	1.856968	-3.594922
H	-5.439809	2.307482	-3.737111
H	-3.702091	2.586378	-3.959084

H	-4.378152	0.964391	-4.233207

103

m-Me-TS-CD+ SCF Done: -3249.65016935 A.U.

Au	1.579659	-0.485265	0.398710
N	0.550573	-2.108951	-0.601916
N	-1.250442	1.257162	0.677596
N	-2.539076	0.964908	0.520127
N	-2.561295	-0.277057	0.099307
C	1.116565	-3.147178	-1.224111
H	2.207074	-3.186225	-1.229896
C	0.323381	-4.124205	-1.823284
H	0.775525	-4.966580	-2.347459
C	-1.060559	-3.989347	-1.692032
H	-1.744357	-4.744016	-2.093944
C	-0.811054	-2.014620	-0.598999
C	-1.297380	-0.772692	-0.018695
C	-0.420279	0.224550	0.380647
C	-0.868593	2.594049	1.072229
C	-0.737404	2.864480	2.448293
C	-0.301599	4.150689	2.796778
H	-0.179517	4.416456	3.847394
C	-0.031347	5.103906	1.813111
H	0.307362	6.100414	2.106833
C	-0.199788	4.804292	0.460323
H	0.007187	5.570422	-0.287151
C	-0.629274	3.533879	0.047746
C	-1.128516	1.832759	3.502262
H	-0.929460	0.831039	3.082489
C	-2.642255	1.915414	3.786736
H	-2.941183	1.141770	4.510806
H	-3.234980	1.777439	2.871387
H	-2.897992	2.899181	4.210993
C	-0.329292	1.947346	4.807486
H	-0.588673	2.861001	5.364221
H	0.758380	1.961938	4.633421
H	-0.558378	1.095075	5.465330
C	-0.888751	3.199763	-1.416210
H	-0.635284	2.139733	-1.583169
C	-0.047505	4.023084	-2.399080
H	1.031655	3.958692	-2.201036
H	-0.336909	5.086273	-2.399261
H	-0.226875	3.653447	-3.421480
C	-2.387502	3.368876	-1.740233
H	-3.026656	2.781185	-1.067764
H	-2.585050	3.034839	-2.769835
H	-2.687422	4.425866	-1.653410
C	-3.830973	-0.965469	-0.033084
C	-4.309361	-1.635791	1.110716

C	-5.527468	-2.312210	0.976979
H	-5.940881	-2.852633	1.830291
C	-6.223343	-2.302521	-0.233769
H	-7.174334	-2.834551	-0.314716
C	-5.717968	-1.616906	-1.336573
H	-6.276825	-1.615352	-2.274423
C	-4.498188	-0.926997	-1.267228
C	-3.529312	-1.676544	2.421086
H	-2.773713	-0.875803	2.398605
C	-4.412924	-1.402944	3.647020
H	-3.793118	-1.345405	4.555806
H	-5.150416	-2.204267	3.808258
H	-4.959386	-0.453599	3.542983
C	-2.779225	-3.014285	2.558480
H	-2.124918	-3.200295	1.693057
H	-3.490035	-3.853862	2.617800
H	-2.163576	-3.025387	3.472515
C	-3.950873	-0.192412	-2.480476
H	-2.973127	0.239138	-2.224441
C	-3.694790	-1.159840	-3.646865
H	-3.034429	-1.980132	-3.332658
H	-3.185469	-0.628268	-4.463693
H	-4.634083	-1.587338	-4.034708
C	-4.879666	0.960526	-2.894807
H	-5.040655	1.668216	-2.067125
H	-5.865928	0.589782	-3.217843
H	-4.439897	1.515285	-3.738324
C	3.331428	-1.602562	0.418597
C	4.316831	-1.399453	-0.549890
H	4.226585	-0.570521	-1.254232
C	5.414850	-2.272224	-0.580426
H	6.193991	-2.128813	-1.334047
C	4.542564	-3.513710	1.328055
C	3.440775	-2.639706	1.353105
H	2.663354	-2.795707	2.107670
N	-1.618159	-2.945764	-1.075017
O	2.835958	1.173029	-1.314166
O	3.234637	2.944851	-2.620141
C	3.178648	2.387995	-1.511371
C	3.578337	3.199345	-0.271801
H	3.381579	4.263448	-0.460469
H	4.672482	3.098339	-0.159232
C	2.869988	2.733200	0.993646
C	3.126702	1.328787	1.389476
H	4.102673	0.903100	1.131469
C	2.294196	0.589347	2.225885
H	2.714928	-0.250558	2.786488
H	1.411495	1.074369	2.647501
H	3.241807	3.318084	1.862072

H	1.788944	2.927678	0.956565
K	1.967968	1.079854	-3.960407
Cl	-0.406293	-0.217853	-2.989521
C	5.523856	-3.313771	0.342184
H	6.387514	-3.983356	0.302915
C	4.680956	-4.624864	2.339018
H	4.950102	-5.575844	1.852849
H	5.481703	-4.397060	3.062828
H	3.752377	-4.781783	2.907536

88

m-Me-TS-decomp+ SCF Done: -1856.07999102 A.U.

Au	-1.957260	-0.451978	-0.361334
N	0.160781	-1.946395	-2.160027
N	0.499680	1.004902	0.943907
N	1.754615	0.729087	1.234041
N	2.089240	-0.270316	0.459501
C	0.133777	-3.057049	-2.899474
H	-0.627271	-3.108997	-3.684741
C	1.032827	-4.104705	-2.681277
H	1.012355	-5.012081	-3.286771
C	1.952869	-3.933535	-1.643183
H	2.689163	-4.706923	-1.400261
C	1.078255	-1.886003	-1.189101
C	1.051403	-0.678983	-0.338039
C	-0.012483	0.183557	-0.035730
C	-0.146951	2.101755	1.631517
C	0.199678	3.414515	1.240907
C	-0.475022	4.455532	1.889438
H	-0.250741	5.489360	1.625229
C	-1.436212	4.192534	2.867528
H	-1.953602	5.022881	3.353872
C	-1.730501	2.882397	3.241361
H	-2.463634	2.699733	4.028227
C	-1.082396	1.794201	2.637345
C	1.275995	3.700870	0.198495
H	1.302643	2.844018	-0.496472
C	2.658983	3.823935	0.870218
H	3.433745	4.013880	0.111700
H	2.932279	2.917081	1.425894
H	2.662972	4.669043	1.576662
C	0.995220	4.951246	-0.647250
H	1.113850	5.874890	-0.059791
H	-0.016355	4.939779	-1.073524
H	1.714672	5.005750	-1.478678
C	-1.318627	0.377500	3.147655
H	-0.937591	-0.334626	2.400011
C	-2.804552	0.054209	3.355190
H	-3.394223	0.255790	2.448705

H	-3.236768	0.633647	4.185480
H	-2.927754	-1.011464	3.599286
C	-0.504408	0.151188	4.436136
H	0.568872	0.331564	4.273099
H	-0.629715	-0.883855	4.791421
H	-0.842380	0.828497	5.236427
C	3.469269	-0.719706	0.467380
C	4.339127	-0.140745	-0.473900
C	5.664403	-0.597338	-0.455867
H	6.385640	-0.187795	-1.164737
C	6.077894	-1.561362	0.464258
H	7.117662	-1.896906	0.463327
C	5.180770	-2.096922	1.390378
H	5.528197	-2.844336	2.105330
C	3.841116	-1.691650	1.409623
C	3.873650	0.907422	-1.479960
H	2.948978	1.362543	-1.091847
C	4.883960	2.051621	-1.651904
H	4.442815	2.856294	-2.260644
H	5.796502	1.717281	-2.169238
H	5.179785	2.476095	-0.681250
C	3.536113	0.263404	-2.837628
H	2.767823	-0.518520	-2.743700
H	4.430579	-0.202878	-3.280046
H	3.163011	1.022885	-3.543362
C	2.847518	-2.245138	2.420076
H	1.833248	-2.051075	2.034619
C	2.964132	-3.764835	2.597235
H	2.890252	-4.281576	1.628552
H	2.155364	-4.132510	3.247545
H	3.916043	-4.051976	3.070439
C	2.985503	-1.502484	3.761917
H	2.860151	-0.416430	3.632532
H	3.981026	-1.675027	4.201131
H	2.229602	-1.855533	4.480763
C	-1.215015	1.263133	-1.389911
C	-0.637959	1.129308	-2.654092
H	-0.133061	0.201965	-2.923366
C	-0.745818	2.184425	-3.573189
C	-2.009739	3.452952	-1.911400
H	-2.554723	4.356915	-1.629977
C	-1.878632	2.422248	-0.974462
H	-2.309385	2.521898	0.020976
I	-3.898081	-2.137386	0.038116
N	1.971412	-2.829920	-0.892577
C	-1.445378	3.339943	-3.183601
H	-1.544314	4.168666	-3.889432
C	-0.153145	2.059170	-4.954055
H	-0.925141	1.754402	-5.680957

H	0.259565	3.018422	-5.301388
H	0.647810	1.305764	-4.985519

14
m-PhNO2I SCF Done: -447.248828029 A.U.

C	2.321914	1.471101	-0.000050
C	2.071196	0.099552	-0.000007
C	-0.292091	0.462665	0.000047
C	-0.077233	1.845748	-0.000113
C	1.231391	2.341463	-0.000259
H	3.350961	1.828569	-0.000056
H	-0.921922	2.536915	-0.000241
H	1.397073	3.421164	-0.000312
I	-2.295958	-0.301899	0.000047
C	0.779523	-0.430058	-0.000046
H	0.647011	-1.511060	0.000012
O	2.970022	-2.034323	-0.000596
O	4.338618	-0.357137	0.000646
N	3.219337	-0.840872	0.000037

87
m-NO2-A-ad+ SCF Done: -2021.16395983 A.U.

Au	0.859973	1.417585	0.238513
N	-1.942744	2.899789	0.916721
N	-0.838264	-1.012056	-0.276997
N	-2.053741	-1.511239	-0.386554
N	-2.858437	-0.507851	-0.136676
C	-2.481290	4.085039	1.203695
H	-1.802867	4.856831	1.582901
C	-3.845859	4.340968	1.035282
H	-4.281704	5.311580	1.277180
C	-4.617909	3.289755	0.536302
H	-5.693711	3.402813	0.363887
C	-2.774124	1.957097	0.451251
C	-2.166042	0.650517	0.137923
C	-0.815533	0.318759	0.044341
C	0.298163	-1.880388	-0.493263
C	0.865262	-1.918312	-1.779914
C	1.956611	-2.778843	-1.962459
H	2.426605	-2.847876	-2.945592
C	2.445400	-3.551869	-0.909939
H	3.297534	-4.215736	-1.068368
C	1.867120	-3.471455	0.358060
H	2.285944	-4.062583	1.173475
C	0.771810	-2.631215	0.600043
C	0.356008	-1.063566	-2.933314
H	-0.527756	-0.505322	-2.588956
C	-0.092177	-1.922959	-4.126624
H	-0.518743	-1.286435	-4.917746

H	-0.857395	-2.654545	-3.826982
H	0.752086	-2.477386	-4.565773
C	1.414678	-0.026298	-3.347856
H	2.322142	-0.516781	-3.734954
H	1.711371	0.601445	-2.492197
H	1.023664	0.632206	-4.139449
C	0.175206	-2.501801	1.996595
H	-0.816838	-2.034635	1.904345
C	1.046632	-1.573417	2.863667
H	1.147126	-0.576866	2.403515
H	2.055716	-1.992952	2.992766
H	0.597286	-1.441882	3.860722
C	-0.036828	-3.861641	2.677494
H	-0.646944	-4.533062	2.054470
H	-0.553068	-3.724577	3.640097
H	0.918664	-4.365070	2.890818
C	-4.284954	-0.773291	-0.136644
C	-4.991039	-0.570899	-1.334399
C	-6.356656	-0.875030	-1.313029
H	-6.954879	-0.741493	-2.215349
C	-6.967595	-1.346676	-0.148560
H	-8.035149	-1.579277	-0.155173
C	-6.230494	-1.523274	1.021717
H	-6.727394	-1.894068	1.919683
C	-4.858382	-1.235605	1.057789
C	-4.288660	-0.081868	-2.594001
H	-3.411347	0.505798	-2.276765
C	-3.773520	-1.276107	-3.419880
H	-3.210409	-0.926954	-4.300059
H	-4.615101	-1.891673	-3.775546
H	-3.114165	-1.924448	-2.823407
C	-5.156434	0.854794	-3.443882
H	-5.553632	1.686177	-2.841648
H	-6.006828	0.325202	-3.901164
H	-4.560584	1.278391	-4.267262
C	-4.031300	-1.465711	2.316750
H	-3.094426	-0.892251	2.220246
C	-4.726515	-0.956612	3.587593
H	-5.027200	0.097795	3.486353
H	-4.047171	-1.040318	4.450187
H	-5.626437	-1.544534	3.826205
C	-3.647380	-2.951954	2.437723
H	-3.096134	-3.295535	1.549379
H	-4.547958	-3.578270	2.539088
H	-3.014564	-3.119515	3.323230
N	-4.084259	2.102952	0.241318
I	3.280030	2.682192	0.374841
C	4.297720	0.870643	-0.235014
C	4.955508	0.841641	-1.465236

H	4.978565	1.716851	-2.116763
C	5.587165	-0.345135	-1.858250
H	6.109931	-0.382111	-2.816293
C	5.549986	-1.474752	-1.039852
H	6.027022	-2.411050	-1.328218
C	4.244372	-0.230145	0.613358
H	3.726267	-0.226251	1.571328
C	4.879730	-1.393932	0.179769
O	5.241108	-3.636325	0.596568
O	4.321878	-2.452060	2.157653
N	4.815600	-2.589982	1.050974

87

m-NO2-B+ SCF Done: -2021.17168190 A.U.

Au	-1.058388	1.430652	0.041339
N	0.862714	2.631533	0.349789
N	0.583067	-1.363924	-0.236882
N	1.860631	-1.718597	-0.241959
N	2.539847	-0.610488	-0.080393
C	0.998904	3.945164	0.560398
H	0.075214	4.525618	0.641684
C	2.267815	4.518865	0.664961
H	2.387342	5.588835	0.839840
C	3.365309	3.665629	0.532631
H	4.390589	4.043821	0.597350
C	1.990311	1.881356	0.241001
C	1.704382	0.464648	0.026868
C	0.409144	-0.022170	-0.080956
C	-0.428999	-2.399031	-0.324971
C	-0.765841	-3.073939	0.862748
C	-1.764156	-4.053242	0.761436
H	-2.081847	-4.594936	1.652534
C	-2.378388	-4.324569	-0.460097
H	-3.170385	-5.074644	-0.508824
C	-2.006043	-3.635562	-1.617135
H	-2.502601	-3.870036	-2.558580
C	-1.010030	-2.652246	-1.582981
C	-0.048327	-2.803084	2.180871
H	0.353685	-1.775315	2.142963
C	1.144683	-3.767146	2.335215
H	1.687219	-3.561181	3.270360
H	1.852167	-3.681191	1.497593
H	0.789702	-4.809129	2.371587
C	-0.966369	-2.874236	3.409286
H	-1.306789	-3.903041	3.602721
H	-1.863420	-2.249787	3.297910
H	-0.416088	-2.543649	4.303638
C	-0.526668	-1.935564	-2.840176
H	-0.310070	-0.889381	-2.560463

C	-1.565928	-1.890806	-3.966441
H	-2.530117	-1.498185	-3.615447
H	-1.738640	-2.889009	-4.398140
H	-1.206483	-1.245439	-4.782457
C	0.783228	-2.570735	-3.351591
H	1.567357	-2.594386	-2.581797
H	1.170239	-2.012365	-4.218391
H	0.602945	-3.609567	-3.669889
C	3.988523	-0.648131	-0.060653
C	4.625782	-0.846806	1.177245
C	6.026692	-0.845418	1.161856
H	6.574025	-0.990472	2.094839
C	6.733088	-0.662970	-0.027765
H	7.825511	-0.667442	-0.013760
C	6.058426	-0.481856	-1.235203
H	6.628450	-0.348319	-2.156696
C	4.658263	-0.469414	-1.283525
C	3.855884	-1.011902	2.482154
H	2.799210	-1.203334	2.237450
C	4.343517	-2.218430	3.298541
H	3.710639	-2.353178	4.189298
H	5.376843	-2.080507	3.652379
H	4.305961	-3.145404	2.707336
C	3.912088	0.284413	3.310964
H	3.538076	1.146155	2.737687
H	4.947699	0.511989	3.609958
H	3.309126	0.185575	4.227354
C	3.927010	-0.251455	-2.602538
H	2.845057	-0.355666	-2.424079
C	4.165989	1.173330	-3.132527
H	3.880549	1.930068	-2.385538
H	3.581819	1.348073	-4.049798
H	5.228178	1.332161	-3.376998
C	4.304220	-1.316379	-3.644935
H	4.113552	-2.332227	-3.266904
H	5.368067	-1.252034	-3.921466
H	3.714361	-1.176195	-4.564166
C	-2.667802	0.241589	-0.366743
C	-3.089572	-0.686350	0.579787
H	-2.616584	-0.798252	1.552919
C	-4.155514	-1.521925	0.241152
C	-4.399013	-0.469717	-1.895215
H	-4.914151	-0.363975	-2.852413
C	-3.316363	0.368314	-1.598878
H	-3.000433	1.118491	-2.325238
I	-2.718467	3.502562	0.301379
N	3.224281	2.351412	0.320720
C	-4.820189	-1.434116	-0.980628
H	-5.644523	-2.113703	-1.193976

O	-5.311436	-3.438498	0.802847
O	-4.102301	-2.502718	2.334465
N	-4.562603	-2.566081	1.205190

87

m-NO2-TS-AB+ SCF Done: -2021.13123824 A.U.

Au	-1.205397	-1.220391	-0.014667
N	1.057050	-2.683805	-1.177332
N	0.826879	1.020144	0.722886
N	2.102453	1.359492	0.674426
N	2.689272	0.394833	0.009205
C	1.277953	-3.842332	-1.796972
H	0.425534	-4.523338	-1.891529
C	2.540103	-4.174878	-2.300046
H	2.722945	-5.124636	-2.804908
C	3.551827	-3.228253	-2.122550
H	4.568955	-3.406514	-2.487237
C	2.096008	-1.841588	-1.060324
C	1.795971	-0.579887	-0.363024
C	0.552394	-0.168926	0.108422
C	-0.106586	1.878251	1.418381
C	-0.607832	3.003862	0.733900
C	-1.507833	3.815176	1.436603
H	-1.922595	4.701484	0.955113
C	-1.880599	3.508572	2.746850
H	-2.580318	4.160258	3.276003
C	-1.364196	2.383942	3.387936
H	-1.660335	2.165372	4.415986
C	-0.452152	1.538064	2.738684
C	-0.149709	3.356448	-0.676671
H	0.134006	2.415034	-1.178398
C	1.098670	4.259896	-0.626306
H	1.448227	4.486932	-1.645047
H	1.924290	3.788470	-0.075175
H	0.859309	5.213180	-0.128790
C	-1.247929	4.009154	-1.527065
H	-1.467836	5.035053	-1.192944
H	-2.187226	3.442430	-1.493965
H	-0.924189	4.076927	-2.576584
C	0.144643	0.341371	3.471611
H	0.880568	-0.140098	2.810033
C	-0.924193	-0.713687	3.802116
H	-1.406075	-1.096996	2.888749
H	-1.709132	-0.300350	4.455475
H	-0.472352	-1.570651	4.325416
C	0.903456	0.786137	4.734008
H	1.674181	1.533857	4.494489
H	1.398676	-0.077293	5.205135
H	0.224307	1.226663	5.480530

C	4.120844	0.477071	-0.207179
C	4.577629	1.175265	-1.335810
C	5.967272	1.229356	-1.515039
H	6.377436	1.754048	-2.379767
C	6.832004	0.620786	-0.606519
H	7.911441	0.678021	-0.765496
C	6.333440	-0.058961	0.507106
H	7.029587	-0.521973	1.207650
C	4.955486	-0.151675	0.734226
C	3.631616	1.801132	-2.353154
H	2.621359	1.825910	-1.914975
C	4.005330	3.254414	-2.681964
H	3.250366	3.698122	-3.349720
H	4.974853	3.318885	-3.199785
H	4.063879	3.870291	-1.772127
C	3.561515	0.936147	-3.624798
H	3.270457	-0.098396	-3.389186
H	4.542291	0.898994	-4.125456
H	2.832966	1.352862	-4.338277
C	4.387352	-0.850786	1.963752
H	3.367519	-1.191392	1.717550
C	5.178562	-2.101432	2.368596
H	5.301813	-2.791496	1.519641
H	4.653136	-2.636103	3.175010
H	6.179836	-1.847873	2.750126
C	4.268126	0.142441	3.135706
H	3.657560	1.016320	2.862698
H	5.263374	0.508737	3.433619
H	3.806491	-0.341817	4.010711
N	3.331490	-2.063518	-1.506519
I	-3.405512	-2.750883	-0.016399
C	-3.248962	-0.231861	-0.045796
C	-3.615133	0.389498	1.151094
C	-4.114832	1.575893	-1.313431
C	-4.234318	1.640892	1.080973
H	-3.413594	-0.076992	2.114668
H	-4.513986	2.145863	2.006913
C	-4.485365	2.249315	-0.150916
H	-4.958114	3.227933	-0.226035
C	-3.516363	0.315762	-1.297493
H	-3.273956	-0.180150	-2.235927
O	-4.118148	1.550601	-3.626788
O	-4.724613	3.375254	-2.625360
N	-4.344319	2.218250	-2.633897

102

m-NO2-TS-CD+ SCF Done: -3414.70263198 A.U.

Au	1.491979	-0.061200	0.175777
N	0.758996	-1.715808	-1.021625

N	-1.597441	0.930570	0.887655
N	-2.804570	0.391595	0.735931
N	-2.600929	-0.743063	0.111689
C	1.478985	-2.500528	-1.828748
H	2.548308	-2.296594	-1.896222
C	0.866650	-3.531671	-2.538568
H	1.444565	-4.168272	-3.209102
C	-0.500373	-3.724827	-2.327384
H	-1.039986	-4.542735	-2.815561
C	-0.586765	-1.928995	-0.938649
C	-1.275191	-0.927969	-0.140372
C	-0.597829	0.163191	0.381988
C	-1.476695	2.232835	1.503931
C	-1.314411	2.287535	2.902278
C	-1.136804	3.559846	3.464047
H	-1.003335	3.663336	4.541451
C	-1.141741	4.702056	2.661265
H	-1.002219	5.683899	3.120068
C	-1.336738	4.607222	1.282358
H	-1.350389	5.516102	0.680502
C	-1.515213	3.362520	0.659888
C	-1.412224	1.033359	3.766127
H	-1.047986	0.180827	3.166208
C	-2.887250	0.747010	4.115052
H	-2.971341	-0.183243	4.697734
H	-3.505229	0.644422	3.211843
H	-3.303286	1.568676	4.719089
C	-0.559517	1.091102	5.041047
H	-0.956028	1.821765	5.762698
H	0.487662	1.363423	4.834049
H	-0.564238	0.110947	5.541701
C	-1.804464	3.233641	-0.830536
H	-1.344161	2.300994	-1.196592
C	-1.232566	4.379286	-1.674392
H	-0.152798	4.523737	-1.528195
H	-1.740475	5.335357	-1.469826
H	-1.405072	4.160536	-2.740549
C	-3.324843	3.118069	-1.065492
H	-3.772645	2.295843	-0.491368
H	-3.523730	2.931615	-2.131508
H	-3.835388	4.050334	-0.774288
C	-3.707931	-1.655413	-0.101561
C	-3.967308	-2.588035	0.922540
C	-5.024880	-3.478537	0.703675
H	-5.266387	-4.225606	1.461656
C	-5.777602	-3.422566	-0.471354
H	-6.600676	-4.125811	-0.619442
C	-5.491514	-2.475896	-1.453289
H	-6.093087	-2.442890	-2.363733

C	-4.439467	-1.561291	-1.295775
C	-3.117733	-2.671706	2.186765
H	-2.552725	-1.731508	2.285503
C	-3.962513	-2.804179	3.462420
H	-3.314365	-2.761728	4.351954
H	-4.500665	-3.763814	3.500165
H	-4.704156	-1.994822	3.538009
C	-2.093604	-3.815871	2.071768
H	-1.466277	-3.707613	1.173730
H	-2.604749	-4.788923	1.998199
H	-1.436704	-3.843637	2.956039
C	-4.127651	-0.539526	-2.378013
H	-3.250697	0.048102	-2.071809
C	-3.738667	-1.226380	-3.696789
H	-2.902494	-1.922289	-3.541114
H	-3.402807	-0.471714	-4.422699
H	-4.586063	-1.781630	-4.131322
C	-5.300308	0.435461	-2.572804
H	-5.561052	0.947812	-1.634065
H	-6.202257	-0.083777	-2.934985
H	-5.035212	1.201256	-3.318428
C	3.445735	-0.725245	-0.067839
C	4.316274	-0.143565	-0.999803
H	3.994326	0.746706	-1.544065
C	5.586884	-0.699998	-1.205692
H	6.264784	-0.244974	-1.931968
C	5.114793	-2.378521	0.437879
C	3.843137	-1.845939	0.665220
H	3.200815	-2.333898	1.399624
N	-1.214244	-2.931126	-1.526286
O	2.268921	2.080389	-1.232490
O	2.181208	4.097168	-2.195056
C	2.327660	3.357525	-1.208891
C	2.637690	4.009279	0.145072
H	2.223234	5.026363	0.156596
H	3.735245	4.117480	0.206864
C	2.118071	3.200983	1.327746
C	2.679102	1.837865	1.467806
H	3.709895	1.684015	1.129965
C	2.062463	0.804547	2.167914
H	2.672911	-0.013445	2.561832
H	1.119547	1.006820	2.678819
H	2.419130	3.700002	2.273896
H	1.020165	3.168449	1.354944
K	1.217884	2.303635	-3.839479
Cl	-0.675454	0.320106	-2.972525
C	5.998516	-1.825777	-0.487758
H	6.980972	-2.276564	-0.627306
O	6.641655	-4.019313	1.007287

O	4.712773	-4.043815	1.993533
N	5.525652	-3.579335	1.210212

87

m-NO2-TS-decomp+ SCF Done: -2021.13064861 A.U.

Au	-1.982196	-0.246901	-0.560568
N	0.281949	-0.565442	-2.774367
N	0.385332	0.130169	1.463033
N	1.622539	-0.291432	1.618706
N	2.009857	-0.705791	0.439842
C	0.344845	-1.032849	-4.023314
H	-0.351138	-0.600406	-4.748981
C	1.257656	-2.027292	-4.385582
H	1.313469	-2.407158	-5.406817
C	2.087228	-2.517448	-3.372611
H	2.820496	-3.306773	-3.568131
C	1.129193	-1.093760	-1.885846
C	1.026711	-0.581055	-0.505248
C	-0.055391	0.008067	0.163690
C	-0.329146	0.666923	2.599609
C	0.028522	1.954333	3.056081
C	-0.736175	2.473025	4.109430
H	-0.509419	3.464900	4.501513
C	-1.786717	1.741520	4.665171
H	-2.374098	2.173408	5.478904
C	-2.081027	0.455439	4.210193
H	-2.882947	-0.110331	4.685049
C	-1.345658	-0.127462	3.168522
C	1.213585	2.728434	2.486631
H	1.350368	2.413955	1.437509
C	2.499244	2.376674	3.264007
H	3.354669	2.936653	2.855758
H	2.730540	1.304303	3.216220
H	2.389202	2.655458	4.323916
C	1.010236	4.250417	2.479352
H	1.041666	4.669562	3.496879
H	0.055092	4.537845	2.020213
H	1.820592	4.730884	1.910172
C	-1.546996	-1.589084	2.780904
H	-1.261634	-1.715052	1.725559
C	-2.998608	-2.066259	2.898212
H	-3.686230	-1.411837	2.341857
H	-3.332283	-2.115011	3.946295
H	-3.092647	-3.079239	2.479549
C	-0.589496	-2.470075	3.608957
H	0.460805	-2.169416	3.472942
H	-0.685901	-3.525761	3.310102
H	-0.823119	-2.396329	4.682834
C	3.397976	-1.084124	0.259751

C	4.258515	-0.111571	-0.282394
C	5.597012	-0.492928	-0.445657
H	6.310563	0.218424	-0.865667
C	6.031955	-1.765455	-0.072111
H	7.082144	-2.036832	-0.202877
C	5.140905	-2.694184	0.468328
H	5.501540	-3.683750	0.755293
C	3.787683	-2.377416	0.639912
C	3.783582	1.276420	-0.702338
H	2.759748	1.423866	-0.322126
C	4.638620	2.393722	-0.083871
H	4.206455	3.378241	-0.322786
H	5.666365	2.384642	-0.478144
H	4.695445	2.295354	1.010432
C	3.723579	1.402641	-2.235877
H	3.116274	0.603952	-2.687695
H	4.732816	1.334628	-2.671776
H	3.282584	2.365770	-2.534706
C	2.804246	-3.388864	1.211020
H	1.787530	-2.978271	1.102203
C	2.829749	-4.712571	0.431308
H	2.649498	-4.538097	-0.640038
H	2.049693	-5.391910	0.809145
H	3.795708	-5.230301	0.538455
C	3.052100	-3.601587	2.714278
H	2.992324	-2.651515	3.267598
H	4.050728	-4.031464	2.892055
H	2.306317	-4.294131	3.134901
C	-1.087201	1.687110	-0.438051
C	-0.378769	2.223595	-1.512526
H	0.144670	1.591033	-2.226713
C	-0.375957	3.607579	-1.654469
C	-1.785227	3.883302	0.267572
H	-2.348977	4.520460	0.951906
C	-1.770254	2.499951	0.481000
H	-2.308025	2.075262	1.328290
I	-4.004859	-1.753941	-1.159524
N	2.020626	-2.055441	-2.121734
C	-1.080372	4.452135	-0.795521
H	-1.058921	5.528880	-0.963871
O	1.092145	3.419198	-3.432745
O	0.370687	5.395803	-2.911607
N	0.425337	4.191697	-2.761460

16

m-PhOMeI SCF Done: -357.343038087 A.U.

C	2.559699	1.130031	-0.000383
C	2.225310	-0.233025	-0.000607
C	-0.118183	0.352582	-0.000145

C	0.192204	1.717743	0.000109
C	1.538077	2.086140	0.000002
H	3.599516	1.455389	-0.000614
H	-0.591823	2.475772	0.000394
H	1.800273	3.147476	0.000153
I	-2.182977	-0.257161	0.000104
C	0.872025	-0.622586	-0.000446
H	0.643288	-1.688662	-0.000573
O	3.123150	-1.247064	-0.001225
C	4.502684	-0.949687	0.001303
H	5.029463	-1.913335	0.002884
H	4.802288	-0.379446	-0.897183
H	4.798693	-0.378328	0.900253

89

m-OMe-A-ad+ SCF Done: -1931.26095122 A.U.

Au	0.965621	1.393969	0.203146
N	-1.877578	2.789897	1.178361
N	-0.751927	-1.001604	-0.374264
N	-1.970131	-1.497363	-0.473982
N	-2.766404	-0.509376	-0.147764
C	-2.417677	3.961013	1.517581
H	-1.769208	4.669371	2.044320
C	-3.747780	4.277776	1.223668
H	-4.186665	5.234395	1.511491
C	-4.480606	3.308558	0.535002
H	-5.525166	3.476888	0.251593
C	-2.672473	1.926739	0.532774
C	-2.067041	0.633834	0.164150
C	-0.719632	0.311499	0.018462
C	0.380204	-1.852963	-0.667865
C	0.967399	-1.764107	-1.942549
C	2.054138	-2.613019	-2.195763
H	2.543774	-2.580834	-3.170944
C	2.510613	-3.502943	-1.225179
H	3.350024	-4.164423	-1.452589
C	1.908472	-3.553106	0.032205
H	2.284174	-4.248481	0.782437
C	0.832075	-2.715836	0.349479
C	0.471424	-0.808040	-3.019447
H	-0.364222	-0.223359	-2.606399
C	-0.071094	-1.573361	-4.238336
H	-0.478262	-0.870606	-4.982489
H	-0.873679	-2.268211	-3.949106
H	0.721906	-2.158409	-4.730533
C	1.567611	0.193826	-3.420234
H	2.423682	-0.313554	-3.892392
H	1.945132	0.742014	-2.542719
H	1.173929	0.926878	-4.141761

C	0.169128	-2.761515	1.720476
H	-0.315205	-1.784645	1.887071
C	1.177396	-2.957831	2.862656
H	2.025535	-2.261556	2.786890
H	1.588851	-3.979861	2.868895
H	0.680251	-2.806270	3.833141
C	-0.928452	-3.841540	1.759254
H	-1.691484	-3.671130	0.985943
H	-1.427147	-3.849867	2.740743
H	-0.492095	-4.839134	1.590597
C	-4.192693	-0.766403	-0.104097
C	-4.929638	-0.564827	-1.283618
C	-6.300926	-0.834983	-1.219053
H	-6.920424	-0.702186	-2.107162
C	-6.890553	-1.267040	-0.028764
H	-7.964281	-1.467304	0.000743
C	-6.123513	-1.446294	1.121590
H	-6.604592	-1.782093	2.041829
C	-4.741865	-1.204582	1.111707
C	-4.256019	-0.106931	-2.569771
H	-3.333196	0.426404	-2.288764
C	-3.849069	-1.321703	-3.424719
H	-3.309990	-0.997555	-4.329083
H	-4.739378	-1.887691	-3.742398
H	-3.198216	-2.007690	-2.861576
C	-5.106693	0.887564	-3.370165
H	-5.419767	1.735596	-2.742307
H	-6.009968	0.415080	-3.786886
H	-4.527724	1.281618	-4.219899
C	-3.905988	-1.363892	2.376407
H	-2.844240	-1.390307	2.084994
C	-4.100213	-0.152998	3.307727
H	-3.849297	0.792334	2.803194
H	-3.463225	-0.243835	4.201823
H	-5.147814	-0.083153	3.641611
C	-4.192641	-2.682225	3.110507
H	-4.075397	-3.547879	2.441664
H	-5.212589	-2.706786	3.524541
H	-3.496800	-2.803762	3.955385
N	-3.944450	2.138003	0.184706
I	3.409009	2.602770	0.297972
C	4.383624	0.681398	-0.003152
C	5.208527	0.514081	-1.114406
H	5.382103	1.313583	-1.835065
C	5.811194	-0.738504	-1.270629
C	4.749188	-1.571105	0.742755
H	6.468653	-0.906909	-2.127003
C	5.593505	-1.776417	-0.360689
H	6.082263	-2.736881	-0.519542

C	4.134184	-0.317331	0.925709
H	3.484120	-0.176690	1.788975
O	4.457246	-2.498179	1.676740
C	5.091635	-3.764420	1.632951
H	6.188400	-3.669335	1.710462
H	4.716488	-4.326752	2.497365
H	4.840191	-4.313074	0.708747

89

m-OMe-B+ SCF Done: -1931.26856483 A.U.

Au	-1.168030	1.373837	0.070730
N	0.733820	2.645735	0.342488
N	0.558344	-1.359343	-0.272107
N	1.846846	-1.676925	-0.273417
N	2.491157	-0.556478	-0.062411
C	0.838781	3.963759	0.537446
H	-0.098895	4.526215	0.581182
C	2.093202	4.562676	0.674412
H	2.188785	5.637331	0.834605
C	3.209022	3.726626	0.595974
H	4.224542	4.124106	0.692043
C	1.875756	1.914860	0.283163
C	1.624265	0.491052	0.066450
C	0.343521	-0.028804	-0.069289
C	-0.408449	-2.423029	-0.475328
C	-0.720302	-3.241219	0.625092
C	-1.634296	-4.278116	0.394654
H	-1.911549	-4.942133	1.213505
C	-2.206826	-4.459216	-0.862275
H	-2.921522	-5.270620	-1.019753
C	-1.883272	-3.613116	-1.924406
H	-2.353328	-3.771732	-2.894746
C	-0.963202	-2.570746	-1.761807
C	-0.087974	-3.037018	1.996860
H	0.209336	-1.975999	2.072286
C	1.184474	-3.894224	2.140970
H	1.652954	-3.726266	3.122861
H	1.923916	-3.665886	1.360130
H	0.933271	-4.963979	2.063810
C	-1.060387	-3.313480	3.154686
H	-1.254013	-4.392154	3.265681
H	-2.030358	-2.817050	3.009405
H	-0.621286	-2.968717	4.103549
C	-0.531756	-1.684790	-2.926156
H	-0.373836	-0.668432	-2.526922
C	-1.584673	-1.564671	-4.034233
H	-2.564485	-1.274845	-3.629718
H	-1.704233	-2.510251	-4.586054
H	-1.272984	-0.803198	-4.765533

C	0.807294	-2.180482	-3.509429
H	1.592727	-2.261524	-2.744771
H	1.163456	-1.497783	-4.296950
H	0.681726	-3.178995	-3.957109
C	3.939053	-0.559427	0.000274
C	4.540285	-0.792837	1.250492
C	5.940402	-0.762208	1.282801
H	6.459900	-0.932060	2.227366
C	6.681443	-0.518215	0.125530
H	7.772683	-0.501140	0.176052
C	6.042772	-0.301660	-1.095295
H	6.639303	-0.119096	-1.991305
C	4.644802	-0.315634	-1.190059
C	3.729336	-1.016645	2.521571
H	2.692337	-1.248867	2.232377
C	4.235425	-2.214895	3.338306
H	3.568787	-2.396307	4.195599
H	5.243438	-2.036645	3.743532
H	4.268881	-3.130842	2.729857
C	3.697078	0.266874	3.371625
H	3.309621	1.121691	2.796995
H	4.709910	0.532610	3.714125
H	3.063502	0.127582	4.261800
C	3.953918	-0.063752	-2.524200
H	2.866664	-0.160414	-2.378196
C	4.218885	1.367585	-3.022496
H	3.920109	2.113024	-2.269646
H	3.658613	1.562905	-3.950453
H	5.287638	1.522445	-3.239287
C	4.353919	-1.113812	-3.573407
H	4.142440	-2.134349	-3.219660
H	5.427178	-1.054643	-3.812684
H	3.795344	-0.952409	-4.508501
C	-2.726645	0.078750	-0.187032
C	-2.981353	-0.830569	0.827322
H	-2.407596	-0.856536	1.753301
C	-4.007858	-1.779430	0.655218
C	-4.492797	-0.830780	-1.516713
H	-5.096386	-0.821795	-2.427860
C	-3.469661	0.110392	-1.369266
H	-3.282716	0.852317	-2.145396
I	-2.886968	3.394330	0.323894
N	3.099140	2.406976	0.400402
C	-4.766904	-1.776815	-0.524961
H	-5.567589	-2.498940	-0.679407
O	-4.162703	-2.655294	1.673138
C	-5.140544	-3.674576	1.574969
H	-4.946787	-4.335977	0.711913
H	-5.072073	-4.260091	2.500775

H	-6.157709	-3.254999	1.488637

89
m-OMe-TS-AB+ SCF Done: -1931.23495734 A.U.

Au	-1.176686	-1.287348	0.086716
N	1.241704	-2.899358	-0.058102
N	0.685632	1.237604	0.018600
N	1.951793	1.615361	-0.055787
N	2.640514	0.501933	-0.075014
C	1.566346	-4.190605	-0.033374
H	0.740833	-4.908729	-0.084249
C	2.898583	-4.608261	0.052415
H	3.167473	-5.665481	0.069587
C	3.864788	-3.601313	0.117574
H	4.931182	-3.840135	0.189673
C	2.244486	-2.010137	0.006556
C	1.824255	-0.599853	-0.013523
C	0.517792	-0.118119	0.046164
C	-0.332180	2.263844	0.098907
C	-0.970206	2.663299	-1.091855
C	-1.920772	3.686035	-0.980034
H	-2.440406	4.037341	-1.871058
C	-2.213289	4.259973	0.257409
H	-2.954215	5.060889	0.320917
C	-1.566160	3.830410	1.415327
H	-1.812927	4.294114	2.371208
C	-0.596130	2.820037	1.364830
C	-0.598074	2.054309	-2.439434
H	-0.374694	0.987930	-2.264284
C	0.675206	2.718466	-3.001863
H	0.982581	2.230239	-3.939928
H	1.517246	2.669416	-2.297677
H	0.486280	3.781911	-3.218672
C	-1.730536	2.113930	-3.473694
H	-1.887982	3.141524	-3.838829
H	-2.687165	1.757789	-3.066592
H	-1.469568	1.500394	-4.349557
C	0.108518	2.331421	2.626702
H	1.068639	1.885905	2.326989
C	-0.713787	1.227228	3.314719
H	-0.890088	0.377801	2.638655
H	-1.696955	1.612206	3.628485
H	-0.189124	0.854977	4.208974
C	0.443714	3.465644	3.604844
H	1.003132	4.273842	3.109741
H	1.060772	3.078865	4.430315
H	-0.461075	3.901722	4.056425
C	4.084451	0.587491	-0.165869
C	4.666692	0.564425	-1.442816

C	6.064800	0.654898	-1.493695
H	6.568877	0.637734	-2.462008
C	6.819540	0.768329	-0.326583
H	7.907733	0.842424	-0.390668
C	6.197805	0.790661	0.923482
H	6.806157	0.884713	1.824548
C	4.805784	0.693353	1.035303
C	3.848006	0.415851	-2.719048
H	2.780445	0.476625	-2.455839
C	4.123140	1.554880	-3.713321
H	3.466185	1.461305	-4.592232
H	5.162876	1.534400	-4.075430
H	3.943862	2.539157	-3.254754
C	4.081981	-0.963859	-3.359613
H	3.860104	-1.776372	-2.651075
H	5.130140	-1.077874	-3.679058
H	3.443728	-1.091865	-4.248272
C	4.109361	0.741843	2.388981
H	3.090902	0.339357	2.261661
C	4.797497	-0.138397	3.441774
H	4.916959	-1.171082	3.079450
H	4.199107	-0.162605	4.365951
H	5.793592	0.246057	3.711005
C	3.969075	2.198829	2.866437
H	3.427225	2.810364	2.128814
H	4.960132	2.655132	3.018721
H	3.423126	2.244550	3.822000
N	3.541110	-2.305443	0.097088
I	-3.235678	-3.002018	0.025129
C	-3.294642	-0.482770	0.366982
C	-3.594057	-0.126472	1.682325
C	-4.423613	1.425305	-0.537849
C	-4.310436	1.060626	1.855905
H	-3.286454	-0.734799	2.531268
H	-4.554393	1.383322	2.871204
C	-4.725679	1.837184	0.770868
H	-5.282558	2.754944	0.952743
C	-3.713723	0.226720	-0.747760
H	-3.507203	-0.098173	-1.766272
O	-4.754173	2.101540	-1.655804
C	-5.525899	3.287443	-1.559162
H	-5.681277	3.638240	-2.587121
H	-6.505776	3.093175	-1.090387
H	-4.995968	4.067353	-0.985876

104

m-OMe-TS-CD+ SCF Done: -3324.80220803 A.U.

Au	1.541084	-0.179191	0.267370
N	0.740579	-1.848216	-0.858275

N	-1.513356	1.033270	0.820716
N	-2.744453	0.548737	0.677197
N	-2.587229	-0.633659	0.131683
C	1.436874	-2.715541	-1.598474
H	2.517665	-2.571762	-1.643505
C	0.784215	-3.750834	-2.265666
H	1.340872	-4.454484	-2.885149
C	-0.595321	-3.859871	-2.077355
H	-1.166548	-4.676334	-2.530912
C	-0.616314	-1.982794	-0.799849
C	-1.267661	-0.900193	-0.079020
C	-0.544003	0.185244	0.391357
C	-1.331470	2.369937	1.339393
C	-1.176797	2.522715	2.731023
C	-0.936462	3.822929	3.197285
H	-0.806320	4.001263	4.265270
C	-0.872296	4.899037	2.310077
H	-0.683889	5.904290	2.694785
C	-1.057311	4.709135	0.939702
H	-1.013294	5.568091	0.269800
C	-1.297067	3.431701	0.411229
C	-1.343430	1.342359	3.683579
H	-1.006398	0.433488	3.154767
C	-2.834617	1.145343	4.025233
H	-2.968614	0.265265	4.673055
H	-3.442540	1.002466	3.120664
H	-3.223661	2.026609	4.559311
C	-0.508978	1.458882	4.966375
H	-0.883395	2.258609	5.623851
H	0.552332	1.668732	4.758782
H	-0.565501	0.520323	5.538631
C	-1.571322	3.203562	-1.070195
H	-1.157861	2.221878	-1.354928
C	-0.924652	4.247226	-1.988955
H	0.159199	4.344272	-1.834718
H	-1.382192	5.242360	-1.868480
H	-1.092463	3.954551	-3.037906
C	-3.091827	3.151517	-1.325571
H	-3.593153	2.403608	-0.696743
H	-3.283171	2.890101	-2.377126
H	-3.554578	4.130518	-1.120238
C	-3.733675	-1.507141	-0.029129
C	-4.039956	-2.354834	1.054456
C	-5.136157	-3.209641	0.890603
H	-5.413906	-3.891149	1.696501
C	-5.882141	-3.200178	-0.289993
H	-6.736120	-3.873763	-0.394930
C	-5.550071	-2.336404	-1.331783
H	-6.146735	-2.337712	-2.246041

C	-4.457512	-1.461963	-1.230663
C	-3.201263	-2.388967	2.328343
H	-2.593353	-1.471606	2.366197
C	-4.057308	-2.392612	3.603343
H	-3.412153	-2.317942	4.493017
H	-4.640514	-3.320987	3.703482
H	-4.760165	-1.546095	3.616407
C	-2.230933	-3.584291	2.301887
H	-1.595969	-3.568630	1.402937
H	-2.786568	-4.535541	2.293777
H	-1.579470	-3.578937	3.190745
C	-4.098190	-0.531046	-2.377705
H	-3.191243	0.029429	-2.110963
C	-3.748752	-1.322192	-3.648081
H	-2.947661	-2.046780	-3.445937
H	-3.376436	-0.634776	-4.421438
H	-4.624446	-1.860946	-4.046087
C	-5.221191	0.486772	-2.635725
H	-5.451841	1.072742	-1.732811
H	-6.149739	-0.009161	-2.961891
H	-4.920896	1.187378	-3.430502
C	3.449763	-0.982228	0.111528
C	4.338742	-0.532870	-0.873763
H	4.081364	0.327428	-1.492836
C	5.558312	-1.204544	-1.018023
H	6.267086	-0.869595	-1.780415
C	5.003053	-2.721573	0.787746
C	3.768135	-2.055470	0.940418
H	3.090512	-2.427098	1.713042
N	-1.284196	-2.985821	-1.340850
O	2.420677	1.828996	-1.305011
O	2.474492	3.758168	-2.436499
C	2.562145	3.095633	-1.389403
C	2.896006	3.837305	-0.088136
H	2.526849	4.869538	-0.157927
H	3.996959	3.901674	-0.026917
C	2.335977	3.142807	1.146432
C	2.827840	1.765778	1.383678
H	3.844498	1.529449	1.050358
C	2.163009	0.824049	2.165614
H	2.737792	0.013908	2.624033
H	1.236989	1.118636	2.663509
H	2.659098	3.691048	2.057373
H	1.237393	3.166035	1.170464
K	1.455078	1.844110	-3.912178
Cl	-0.624919	0.081593	-3.001697
C	5.899896	-2.290250	-0.204356
H	6.860704	-2.784556	-0.345188
O	5.219408	-3.752425	1.630469

C	6.438396	-4.471730	1.560028
H	6.387601	-5.244959	2.337254
H	6.568030	-4.957523	0.576618
H	7.307146	-3.819113	1.757059

89

m-OMe-TS-decomp+ SCF Done: -1931.23201898 A.U.

Au	-1.989549	-0.273220	-0.521320
N	0.134470	-1.191854	-2.613587
N	0.428690	0.401462	1.355572
N	1.672991	0.016694	1.555267
N	2.032330	-0.608970	0.464249
C	0.133796	-1.933954	-3.722356
H	-0.640073	-1.712076	-4.464215
C	1.075496	-2.946875	-3.925318
H	1.077655	-3.553079	-4.832435
C	2.007191	-3.149466	-2.903051
H	2.773827	-3.928029	-2.976646
C	1.070301	-1.466013	-1.698730
C	1.020350	-0.670471	-0.457440
C	-0.043639	0.043950	0.111908
C	-0.268712	1.103816	2.411301
C	0.076276	2.450604	2.655548
C	-0.658477	3.109342	3.649973
H	-0.440200	4.152869	3.878462
C	-1.670296	2.453410	4.352186
H	-2.235278	2.993337	5.115735
C	-1.954993	1.110369	4.105062
H	-2.727535	0.610321	4.689583
C	-1.247695	0.389516	3.132685
C	1.216063	3.152090	1.923433
H	1.300131	2.697260	0.921592
C	2.548685	2.932458	2.669529
H	3.368272	3.441954	2.139527
H	2.802357	1.868240	2.760718
H	2.488783	3.356641	3.684350
C	0.981868	4.655623	1.718549
H	1.067828	5.212343	2.664594
H	-0.004779	4.864172	1.285089
H	1.746049	5.060505	1.037455
C	-1.437758	-1.116273	2.976310
H	-1.196643	-1.394685	1.939644
C	-2.874140	-1.588070	3.227287
H	-3.595656	-1.032522	2.609970
H	-3.162815	-1.483679	4.284699
H	-2.966444	-2.653466	2.968719
C	-0.431523	-1.853245	3.883263
H	0.606671	-1.562782	3.660493
H	-0.519971	-2.942856	3.748021

H	-0.622975	-1.624169	4.943569
C	3.429630	-0.960894	0.298549
C	4.233866	-0.037730	-0.396503
C	5.581148	-0.388258	-0.555630
H	6.253385	0.285477	-1.089820
C	6.078386	-1.582538	-0.030796
H	7.134330	-1.830820	-0.161407
C	5.243919	-2.459816	0.663762
H	5.654624	-3.385227	1.071632
C	3.884509	-2.172110	0.838904
C	3.680554	1.264077	-0.970764
H	2.705825	1.461276	-0.498177
C	4.569027	2.474290	-0.645253
H	4.069678	3.404067	-0.960455
H	5.533756	2.427273	-1.173620
H	4.775843	2.541391	0.433021
C	3.434949	1.149468	-2.486901
H	2.806333	0.281335	-2.734834
H	4.387151	1.029117	-3.027602
H	2.928861	2.050217	-2.869024
C	2.959929	-3.116807	1.591980
H	1.922827	-2.788457	1.416042
C	3.059631	-4.559658	1.075658
H	2.878054	-4.600797	-0.008751
H	2.312506	-5.194725	1.576751
H	4.049237	-4.998877	1.276796
C	3.218668	-3.030584	3.106467
H	3.106578	-1.998470	3.473051
H	4.240293	-3.364058	3.349302
H	2.512463	-3.671527	3.657151
C	-1.021697	1.617174	-0.805770
C	-0.312847	1.896591	-1.968773
H	0.168928	1.113288	-2.549311
C	-0.235736	3.228252	-2.415715
C	-1.622356	3.911761	-0.545841
H	-2.155609	4.698484	-0.007176
C	-1.664243	2.607226	-0.047959
H	-2.204646	2.380239	0.869496
I	-4.086159	-1.788040	-0.772888
N	2.002077	-2.415618	-1.788217
C	-0.910726	4.236839	-1.704902
H	-0.886274	5.272560	-2.041816
O	0.507813	3.424356	-3.522230
C	0.606738	4.726425	-4.080811
H	1.077401	5.433849	-3.375790
H	1.239771	4.632025	-4.971832
H	-0.382939	5.112602	-4.379425

15

o-PhCF3I SCF Done: -579.686376842 A.U.

C	2.709185	-1.971582	0.000003
C	2.560158	-0.585011	-0.000009
C	1.289951	0.010620	-0.000006
C	0.154610	-0.820784	-0.000014
C	0.302744	-2.211503	-0.000008
C	1.576639	-2.785509	0.000009
H	3.709099	-2.410795	0.000017
H	3.440243	0.058318	-0.000009
H	-0.579299	-2.853932	0.000008
H	1.674086	-3.873901	0.000014
I	-1.871646	-0.085667	0.000001
C	1.209718	1.527447	0.000000
F	0.568177	1.992701	-1.083981
F	0.568123	1.992692	1.083955
F	2.434265	2.085562	0.000035

88

o-CF3-A-ad+ SCF Done: -2153.60515506 A.U.

Au	-0.920373	-1.207579	0.174257
N	1.859227	-3.005921	0.499962
N	0.972234	1.115294	-0.045965
N	2.220233	1.528040	-0.142643
N	2.940258	0.433206	-0.137727
C	2.314020	-4.259582	0.511270
H	1.654414	-5.020063	0.942921
C	3.572703	-4.597277	0.003909
H	3.943110	-5.623409	0.024929
C	4.325749	-3.553362	-0.538262
H	5.316782	-3.728787	-0.970489
C	2.667972	-2.079508	-0.030445
C	2.159977	-0.695494	-0.035278
C	0.842840	-0.247427	0.029717
C	-0.097383	2.087911	-0.023051
C	-0.736503	2.395567	-1.236048
C	-1.786286	3.323417	-1.176034
H	-2.315639	3.593405	-2.091988
C	-2.158264	3.907293	0.034328
H	-2.975951	4.631700	0.059289
C	-1.499762	3.572318	1.218240
H	-1.817849	4.029834	2.155529
C	-0.452271	2.643283	1.221072
C	-0.339282	1.758110	-2.561166
H	0.526776	1.102415	-2.384726
C	0.100111	2.813757	-3.588920
H	0.445164	2.326285	-4.514410
H	0.924008	3.430838	-3.200212
H	-0.729615	3.486684	-3.857208
C	-1.473899	0.873939	-3.103911

H	-2.372928	1.469069	-3.328085
H	-1.761897	0.103828	-2.371555
H	-1.162939	0.368937	-4.032185
C	0.287328	2.273516	2.500652
H	0.716358	1.267849	2.357243
C	-0.634900	2.187468	3.724891
H	-1.503413	1.541801	3.528896
H	-1.006717	3.179016	4.027833
H	-0.081412	1.776345	4.583200
C	1.454997	3.248003	2.746583
H	2.152988	3.265219	1.896421
H	2.016740	2.957640	3.647925
H	1.077526	4.272305	2.895448
C	4.383554	0.565121	-0.168194
C	5.010885	0.601784	-1.425502
C	6.402059	0.749496	-1.426353
H	6.941143	0.790258	-2.373837
C	7.113595	0.838989	-0.227347
H	8.200387	0.949350	-0.251230
C	6.452186	0.789156	0.998817
H	7.027524	0.857542	1.923738
C	5.057055	0.655937	1.060352
C	4.203973	0.514438	-2.713661
H	3.271958	-0.026790	-2.481963
C	3.816396	1.922191	-3.204538
H	3.183707	1.859369	-4.104277
H	4.716043	2.504433	-3.460411
H	3.264321	2.479568	-2.432832
C	4.911862	-0.289259	-3.811731
H	5.215117	-1.281269	-3.443877
H	5.808451	0.228035	-4.187729
H	4.237454	-0.430525	-4.670663
C	4.333766	0.563875	2.399044
H	3.257949	0.717842	2.220726
C	4.504510	-0.840212	3.007750
H	4.144783	-1.625085	2.324964
H	3.944799	-0.925148	3.952668
H	5.565637	-1.046139	3.220886
C	4.775385	1.657091	3.383369
H	4.665553	2.660047	2.944476
H	5.825411	1.531985	3.690139
H	4.162582	1.613309	4.297298
N	3.873680	-2.298209	-0.561805
I	-3.433650	-2.283298	0.296778
C	-4.291226	-0.588581	-0.767281
C	-4.588000	-0.812765	-2.110340
H	-4.404579	-1.785699	-2.568664
C	-5.118704	0.229580	-2.878434
C	-5.037667	1.686392	-0.953639

H	-5.352816	0.052810	-3.930522
H	-5.214567	2.659864	-0.496702
C	-5.338400	1.478853	-2.300810
H	-5.750172	2.297043	-2.895191
C	-4.513483	0.657068	-0.159352
C	-4.252737	0.937929	1.310637
F	-2.956790	0.732842	1.637952
F	-4.538725	2.203731	1.632649
F	-4.988445	0.139082	2.097613

88

o-CF3-B+ SCF Done: -2153.61822353 A.U.

Au	1.378278	-1.034675	-0.135444
N	-0.224814	-2.630132	0.121182
N	-0.826550	1.318454	-0.488015
N	-2.149269	1.400426	-0.400254
N	-2.561969	0.198594	-0.084131
C	-0.067409	-3.947282	0.287742
H	0.953777	-4.331112	0.204078
C	-1.168536	-4.763652	0.554788
H	-1.050273	-5.839494	0.689692
C	-2.415945	-4.141897	0.641704
H	-3.323970	-4.717169	0.849238
C	-1.478527	-2.115116	0.227483
C	-1.512724	-0.670223	0.012820
C	-0.362640	0.064887	-0.232745
C	-0.089254	2.504214	-0.882462
C	0.093979	3.515439	0.077633
C	0.785524	4.660473	-0.344140
H	0.956636	5.474444	0.361372
C	1.253635	4.774709	-1.650957
H	1.788838	5.676629	-1.957125
C	1.045165	3.749659	-2.575654
H	1.419959	3.866526	-3.592367
C	0.364015	2.581274	-2.216560
C	-0.471948	3.413536	1.488539
H	-0.612647	2.346846	1.717608
C	-1.848738	4.101217	1.567671
H	-2.268692	3.999673	2.580565
H	-2.562074	3.665934	0.852977
H	-1.757788	5.176034	1.343936
C	0.479103	3.971034	2.557090
H	0.553217	5.068464	2.505529
H	1.491248	3.555878	2.460382
H	0.105021	3.717870	3.560695
C	0.075901	1.476740	-3.230086
H	0.181876	0.510860	-2.707055
C	1.052981	1.447565	-4.412237
H	2.102246	1.443147	-4.082477

H	0.911957	2.313313	-5.077851
H	0.881550	0.544575	-5.017663
C	-1.374514	1.579233	-3.744859
H	-2.110218	1.588562	-2.928984
H	-1.610915	0.734241	-4.410707
H	-1.510475	2.510708	-4.316558
C	-3.971051	-0.034304	0.160719
C	-4.449784	0.223560	1.459594
C	-5.810907	-0.022196	1.678661
H	-6.238206	0.157127	2.666337
C	-6.631327	-0.491062	0.650453
H	-7.690968	-0.671970	0.845622
C	-6.114378	-0.724371	-0.623551
H	-6.774104	-1.081918	-1.416660
C	-4.759053	-0.501735	-0.902821
C	-3.531190	0.688395	2.584467
H	-2.641479	1.150822	2.130251
C	-4.170052	1.763032	3.475166
H	-3.426835	2.147100	4.190901
H	-5.009476	1.362864	4.064700
H	-4.542577	2.609879	2.879552
C	-3.051713	-0.515564	3.416871
H	-2.564366	-1.275788	2.787764
H	-3.901924	-1.002132	3.920917
H	-2.334343	-0.193127	4.187756
C	-4.198889	-0.749208	-2.297098
H	-3.115756	-0.549563	-2.276906
C	-4.378606	-2.215014	-2.725867
H	-3.933764	-2.901378	-1.989324
H	-3.899877	-2.391166	-3.702028
H	-5.443475	-2.476554	-2.827977
C	-4.811946	0.222284	-3.319722
H	-4.655479	1.269989	-3.020530
H	-5.896437	0.059996	-3.422151
H	-4.355579	0.075975	-4.311223
C	2.660828	0.555572	-0.128034
C	2.954217	1.218742	1.074706
C	3.818220	2.322921	1.044077
H	4.057147	2.835422	1.976700
C	4.079085	2.084446	-1.344414
H	4.516517	2.411211	-2.290641
C	3.225744	0.975492	-1.328905
H	3.015570	0.439685	-2.254827
I	3.465413	-2.694024	-0.147970
N	-2.568235	-2.821980	0.478184
C	4.372882	2.759461	-0.159600
H	5.041953	3.622161	-0.166791
C	2.395507	0.758642	2.403713
F	1.029299	0.754236	2.402188

F	2.764584	-0.497570	2.703799
F	2.772033	1.547229	3.414223

88

o-CF3-TS-AB+ SCF Done: -2153.57810683 A.U.

Au	-1.192852	-1.061614	0.174541
N	1.008211	-2.889596	0.137209
N	0.914637	1.268792	-0.069265
N	2.213900	1.503452	-0.139543
N	2.779777	0.325112	-0.062189
C	1.178314	-4.205488	0.250499
H	0.276395	-4.825859	0.212810
C	2.449988	-4.766471	0.407485
H	2.593257	-5.844436	0.495509
C	3.524870	-3.874782	0.448153
H	4.553448	-4.229568	0.572665
C	2.106155	-2.118190	0.185021
C	1.849083	-0.675293	0.062785
C	0.601807	-0.054744	0.062033
C	-0.013203	2.378482	-0.089714
C	-0.565403	2.764096	-1.329076
C	-1.527041	3.782049	-1.289364
H	-1.999497	4.114477	-2.213369
C	-1.888525	4.381808	-0.081164
H	-2.644735	5.170717	-0.077033
C	-1.278425	4.006318	1.115127
H	-1.554369	4.511764	2.041770
C	-0.306878	2.995728	1.139755
C	-0.061682	2.171844	-2.642883
H	0.099116	1.092043	-2.477431
C	1.293582	2.806893	-3.024528
H	1.687587	2.341725	-3.941369
H	2.049346	2.699786	-2.234936
H	1.165360	3.883501	-3.219439
C	-1.038759	2.316635	-3.816451
H	-1.132707	3.367840	-4.132302
H	-2.039207	1.938781	-3.577791
H	-0.659172	1.754950	-4.683503
C	0.405395	2.604006	2.432261
H	1.345206	2.103187	2.158151
C	-0.418810	1.602635	3.255949
H	-0.646272	0.696501	2.675595
H	-1.376030	2.047800	3.567500
H	0.131450	1.304151	4.162471
C	0.800183	3.825315	3.275791
H	1.372353	4.555835	2.684038
H	1.425359	3.508660	4.124942
H	-0.079429	4.338789	3.694696
C	4.224899	0.247220	-0.131081

C	4.814481	0.082196	-1.394298
C	6.214320	0.010342	-1.424663
H	6.724577	-0.123056	-2.380525
C	6.963326	0.109467	-0.252462
H	8.053522	0.055808	-0.300936
C	6.334469	0.280760	0.982581
H	6.939861	0.362949	1.886756
C	4.939303	0.347911	1.074695
C	3.995873	-0.051429	-2.672531
H	2.946105	0.185329	-2.438144
C	4.441873	0.947895	-3.751201
H	3.782987	0.877256	-4.630816
H	5.468650	0.745484	-4.093595
H	4.405811	1.982478	-3.378178
C	4.034162	-1.499422	-3.193284
H	3.686932	-2.210228	-2.427988
H	5.059329	-1.788849	-3.474427
H	3.396284	-1.608084	-4.084712
C	4.235613	0.564943	2.408125
H	3.182037	0.262547	2.289222
C	4.814509	-0.303249	3.533905
H	4.829752	-1.366251	3.247997
H	4.205536	-0.198262	4.445442
H	5.841850	-0.005374	3.795248
C	4.242651	2.059391	2.779391
H	3.782706	2.670541	1.987799
H	5.273760	2.418958	2.924559
H	3.687747	2.230453	3.715614
N	3.355630	-2.553929	0.340329
I	-3.410409	-2.576905	0.134242
C	-3.220009	-0.148704	0.785819
C	-3.757450	0.815086	-0.081418
C	-3.312150	-0.023552	2.174030
C	-4.335577	1.956884	0.491044
C	-3.904016	1.121425	2.714579
H	-2.930553	-0.806583	2.829200
H	-4.732623	2.725898	-0.172143
H	-3.970432	1.221503	3.800358
C	-4.409354	2.114430	1.875206
H	-4.874733	3.008388	2.294554
C	-3.823812	0.655348	-1.586013
F	-2.687457	0.138800	-2.103184
F	-4.824347	-0.159667	-1.945478
F	-4.028998	1.835134	-2.193808

103
o-CF3-TS-CD+ SCF Done: -3547.15509686 A.U.

Au	1.494464	-0.300676	0.166734
N	0.519174	-1.813540	-1.062608

N	-1.384031	1.194883	0.801217
N	-2.662942	0.874548	0.619601
N	-2.646527	-0.278813	-0.004339
C	1.114734	-2.706600	-1.856117
H	2.204338	-2.688942	-1.895887
C	0.354300	-3.616091	-2.588522
H	0.832847	-4.339448	-3.249234
C	-1.030641	-3.574471	-2.413719
H	-1.688483	-4.287823	-2.920469
C	-0.843027	-1.793153	-1.019168
C	-1.368674	-0.690260	-0.232548
C	-0.525025	0.262830	0.313937
C	-1.053100	2.454006	1.429915
C	-1.046419	2.505746	2.837580
C	-0.660740	3.721979	3.419676
H	-0.633565	3.818382	4.505686
C	-0.318963	4.816361	2.624920
H	-0.019934	5.754576	3.098525
C	-0.368239	4.732894	1.231898
H	-0.111742	5.609667	0.637435
C	-0.745395	3.544859	0.589158
C	-1.499813	1.321161	3.686952
H	-1.301619	0.398792	3.113410
C	-3.021328	1.391510	3.926868
H	-3.359186	0.517028	4.504086
H	-3.578494	1.416174	2.979854
H	-3.277129	2.298368	4.497309
C	-0.748050	1.194762	5.019616
H	-1.007729	2.009537	5.712872
H	0.345247	1.205328	4.886256
H	-1.021365	0.251405	5.516484
C	-0.885080	3.443581	-0.925273
H	-0.523088	2.450903	-1.238432
C	-0.067985	4.492034	-1.688949
H	1.002760	4.468350	-1.441816
H	-0.441170	5.513256	-1.512449
H	-0.175550	4.317125	-2.772068
C	-2.367588	3.540379	-1.340856
H	-2.999402	2.818505	-0.807626
H	-2.463710	3.332034	-2.416995
H	-2.765562	4.547865	-1.138276
C	-3.889754	-0.990610	-0.231023
C	-4.318646	-1.859240	0.792883
C	-5.510958	-2.555185	0.562228
H	-5.887936	-3.245297	1.318818
C	-6.228549	-2.375178	-0.622606
H	-7.159096	-2.926208	-0.778992
C	-5.772015	-1.496397	-1.603073
H	-6.348267	-1.362519	-2.520604

C	-4.579072	-0.777649	-1.434465
C	-3.512418	-2.075632	2.069671
H	-2.823357	-1.224832	2.187336
C	-4.388514	-2.098988	3.330701
H	-3.754658	-2.138314	4.230661
H	-5.044080	-2.983048	3.358550
H	-5.023195	-1.202477	3.395127
C	-2.656608	-3.351278	1.961042
H	-1.999899	-3.325956	1.078579
H	-3.298617	-4.241556	1.866921
H	-2.027872	-3.477425	2.857067
C	-4.083244	0.177323	-2.507875
H	-3.095960	0.560436	-2.214313
C	-3.872200	-0.543233	-3.848158
H	-3.205101	-1.407848	-3.722903
H	-3.387462	0.139038	-4.561331
H	-4.824864	-0.888845	-4.282101
C	-5.029677	1.380574	-2.651545
H	-5.153828	1.913907	-1.696205
H	-6.029589	1.068547	-2.994409
H	-4.628352	2.091681	-3.390459
C	3.344026	-1.238798	-0.137068
C	4.170795	-0.700431	-1.131408
H	3.875572	0.225519	-1.623782
C	5.359952	-1.338385	-1.505319
H	5.986689	-0.895630	-2.283608
C	4.926659	-3.078038	0.104923
H	5.214247	-4.011213	0.591259
C	3.730749	-2.445506	0.484722
N	-1.618931	-2.672112	-1.626895
O	2.186003	1.670552	-1.154016
O	3.290692	3.354069	-2.089900
C	2.983413	2.673274	-1.099972
C	3.602630	3.036990	0.250364
H	3.730696	4.128150	0.275326
H	4.622239	2.611776	0.266688
C	2.805333	2.565752	1.467749
C	2.864608	1.106254	1.759737
H	3.840021	0.619552	1.643174
C	1.850540	0.391206	2.367108
H	2.046079	-0.577083	2.827794
H	0.937090	0.912281	2.650782
H	3.245914	3.029830	2.373567
H	1.762306	2.910185	1.422994
K	1.636603	2.088721	-3.796586
Cl	-0.574043	0.364757	-3.132895
C	5.740400	-2.529366	-0.886069
H	6.666702	-3.033119	-1.170287
C	2.930442	-3.095014	1.593450

F	3.081719	-2.429799	2.767098
F	1.598264	-3.109519	1.340105
F	3.294001	-4.359500	1.816392

88

o-CF3-TS-decomp+ SCF Done: -2153.57258391 A.U.

Au	-1.893076	-0.535487	-0.269250
N	0.033077	-2.584021	-0.870686
N	0.517312	1.212864	0.913561
N	1.765521	0.962705	1.242333
N	2.112661	-0.087365	0.542276
C	0.040359	-3.770347	-1.478237
H	-0.885914	-4.352535	-1.430485
C	1.183191	-4.239594	-2.133165
H	1.201385	-5.213690	-2.624518
C	2.300677	-3.399133	-2.131304
H	3.233003	-3.690818	-2.625777
C	1.149530	-1.856249	-0.936914
C	1.090307	-0.556153	-0.231780
C	0.013815	0.317383	-0.010183
C	-0.143235	2.317775	1.569891
C	0.120898	3.616985	1.091020
C	-0.563551	4.662733	1.721900
H	-0.402004	5.689738	1.392446
C	-1.454144	4.410005	2.767814
H	-1.980461	5.242155	3.241393
C	-1.667737	3.109722	3.226009
H	-2.348321	2.938529	4.061466
C	-1.006737	2.019883	2.640152
C	1.129106	3.875107	-0.022952
H	1.177582	2.967474	-0.649155
C	2.529554	4.115725	0.574798
H	3.267473	4.266694	-0.227558
H	2.861215	3.271078	1.194206
H	2.524102	5.017877	1.206829
C	0.736474	5.035660	-0.947501
H	0.807464	6.007368	-0.434609
H	-0.286436	4.923817	-1.331865
H	1.422771	5.075812	-1.807150
C	-1.165209	0.614043	3.207721
H	-0.771386	-0.106852	2.474992
C	-2.630351	0.231434	3.458097
H	-3.239755	0.345045	2.548799
H	-3.082257	0.838759	4.257354
H	-2.697152	-0.822378	3.767469
C	-0.308907	0.469217	4.479915
H	0.749802	0.694883	4.278824
H	-0.373698	-0.558181	4.870840
H	-0.656810	1.155905	5.267797

C	3.422566	-0.673494	0.747711
C	4.497714	-0.176160	-0.008282
C	5.740277	-0.786787	0.205373
H	6.609875	-0.442553	-0.357590
C	5.882202	-1.830474	1.120821
H	6.861845	-2.291293	1.267903
C	4.786778	-2.288420	1.853568
H	4.918757	-3.101181	2.570551
C	3.518404	-1.717018	1.685701
C	4.344708	0.943938	-1.029361
H	3.320687	1.342264	-0.956061
C	5.304205	2.109308	-0.738716
H	5.120173	2.937156	-1.441336
H	6.355708	1.805765	-0.859988
H	5.178291	2.492055	0.285045
C	4.520671	0.409701	-2.461652
H	3.812004	-0.404521	-2.664624
H	5.542197	0.026436	-2.615302
H	4.349254	1.215075	-3.193125
C	2.325450	-2.227106	2.486728
H	1.437885	-1.632464	2.218155
C	2.006224	-3.692042	2.141316
H	1.837575	-3.826194	1.062011
H	1.099704	-4.023311	2.671368
H	2.830737	-4.359667	2.436466
C	2.542449	-2.036484	3.997291
H	2.750324	-0.983640	4.241469
H	3.388267	-2.640650	4.360794
H	1.645766	-2.348470	4.555141
C	-1.315387	1.296767	-1.243885
C	-0.897326	1.421714	-2.587769
C	-1.345189	2.521714	-3.329506
H	-1.027282	2.617021	-4.368136
C	-2.585291	3.358604	-1.437773
H	-3.247309	4.095908	-0.979309
C	-2.130429	2.285397	-0.666950
H	-2.453415	2.211127	0.369217
I	-3.851399	-2.188396	0.218402
N	2.284832	-2.202235	-1.537685
C	-2.194364	3.478382	-2.769401
H	-2.540724	4.317143	-3.376398
C	0.035468	0.450502	-3.275690
F	1.298036	0.573750	-2.798436
F	-0.339867	-0.828256	-3.076698
F	0.096652	0.648359	-4.591479

15
o-PhMeI SCF Done: -282.193421759 A.U.

C	3.314158	-0.403732	0.000002

C	2.691693	0.845267	-0.000002
C	1.291651	0.983312	0.000002
C	0.541239	-0.206282	-0.000000
C	1.145159	-1.466339	-0.000004
C	2.538956	-1.564803	0.000002
H	4.404908	-0.468081	0.000003
H	3.302911	1.752062	-0.000002
H	0.534391	-2.370871	-0.000005
H	3.009802	-2.550903	0.000004
I	-1.620047	-0.146086	0.000000
C	0.665399	2.353480	-0.000001
H	0.023251	2.497692	-0.884620
H	0.023260	2.497703	0.884623
H	1.434431	3.139530	-0.000009

88

o-Me-A-ad+ SCF Done: -1856.10763494 A.U.

Au	-1.199456	-1.171226	-0.205946
N	1.490284	-2.983766	0.553971
N	0.726027	1.110663	-0.218176
N	1.974831	1.527902	-0.158634
N	2.688135	0.437638	-0.015544
C	1.924727	-4.235853	0.700487
H	1.185618	-4.981130	1.013615
C	3.258072	-4.590489	0.471605
H	3.609712	-5.615222	0.601215
C	4.112037	-3.565155	0.059586
H	5.169004	-3.754337	-0.156558
C	2.395248	-2.074534	0.168279
C	1.902210	-0.692496	0.021292
C	0.588466	-0.248828	-0.110484
C	-0.353769	2.060907	-0.366915
C	-0.859847	2.284171	-1.661055
C	-1.934552	3.176572	-1.767965
H	-2.363330	3.390973	-2.747527
C	-2.467419	3.794699	-0.636249
H	-3.308247	4.483733	-0.743041
C	-1.944908	3.536937	0.630873
H	-2.385186	4.024778	1.501021
C	-0.870084	2.653969	0.799574
C	-0.234942	1.623561	-2.883641
H	0.188389	0.659071	-2.560621
C	0.926446	2.483504	-3.417793
H	1.422726	1.983081	-4.264342
H	1.681483	2.669450	-2.639709
H	0.556754	3.460634	-3.767570
C	-1.251130	1.304721	-3.987404
H	-1.645710	2.216997	-4.461715
H	-2.102813	0.726701	-3.594996

H	-0.771015	0.711485	-4.780766
C	-0.264727	2.378080	2.170451
H	0.193629	1.375364	2.136065
C	-1.308630	2.347360	3.294615
H	-2.141976	1.672991	3.050391
H	-1.726765	3.346586	3.493546
H	-0.845023	2.000007	4.230763
C	0.852997	3.392251	2.480126
H	1.639466	3.372705	1.711612
H	1.316842	3.170394	3.453755
H	0.444236	4.414511	2.521225
C	4.121706	0.573925	0.145884
C	4.914756	0.640008	-1.010989
C	6.296642	0.763655	-0.817882
H	6.956507	0.819843	-1.686040
C	6.841419	0.810647	0.465647
H	7.922773	0.902211	0.592490
C	6.016194	0.749579	1.588962
H	6.459474	0.795480	2.585539
C	4.625717	0.636303	1.456765
C	4.329711	0.567646	-2.414999
H	3.235528	0.477210	-2.330324
C	4.613636	1.855473	-3.205441
H	4.130122	1.814096	-4.194058
H	5.693944	1.995368	-3.368573
H	4.235625	2.742831	-2.674951
C	4.828745	-0.681483	-3.160653
H	4.606487	-1.593362	-2.587073
H	5.917041	-0.640001	-3.326599
H	4.344847	-0.759259	-4.147288
C	3.729313	0.557395	2.687395
H	2.682495	0.657342	2.359860
C	3.867068	-0.811138	3.378550
H	3.639011	-1.638122	2.689302
H	3.183821	-0.882029	4.239706
H	4.894022	-0.960987	3.748234
C	3.993602	1.709317	3.669852
H	3.903788	2.687114	3.173617
H	4.999455	1.644244	4.113020
H	3.268723	1.676030	4.498530
N	3.682431	-2.311438	-0.096704
I	-3.759152	-2.085353	-0.388731
C	-4.527809	-0.133760	0.232310
C	-5.092960	0.656529	-0.766624
H	-5.146483	0.309667	-1.799676
C	-5.591265	1.915476	-0.418909
C	-4.932362	1.531124	1.877573
H	-6.040082	2.547275	-1.188445
H	-4.873395	1.880402	2.911395

C	-5.509352	2.348718	0.905161
H	-5.897880	3.330297	1.185786
C	-4.420337	0.254919	1.575446
C	-3.792328	-0.591207	2.651251
H	-3.881712	-0.107653	3.632946
H	-4.265868	-1.583461	2.716536
H	-2.719692	-0.754776	2.449645

88

o-Me-B+ SCF Done: -1856.11564285 A.U.

Au	1.482905	-0.989042	-0.089151
N	-0.170604	-2.607791	-0.059036
N	-0.700171	1.410453	-0.036984
N	-2.023205	1.496920	0.012533
N	-2.458139	0.262874	0.074569
C	-0.041671	-3.937571	-0.078944
H	0.975935	-4.327223	-0.181071
C	-1.165546	-4.760516	0.027129
H	-1.071265	-5.847014	0.008581
C	-2.404721	-4.130259	0.160096
H	-3.329179	-4.709167	0.253483
C	-1.416952	-2.087553	0.068933
C	-1.420259	-0.624996	0.064303
C	-0.252481	0.124288	-0.003314
C	0.065080	2.634656	-0.178301
C	0.575933	3.236240	0.987051
C	1.306575	4.417060	0.807808
H	1.736476	4.922994	1.672158
C	1.492709	4.960444	-0.464099
H	2.064776	5.884301	-0.576724
C	0.960761	4.337303	-1.591488
H	1.119365	4.781094	-2.575111
C	0.233720	3.145104	-1.477740
C	0.289263	2.663638	2.370610
H	0.288353	1.563982	2.276698
C	-1.109235	3.098151	2.855488
H	-1.342887	2.632830	3.825954
H	-1.902020	2.825787	2.144640
H	-1.144101	4.191250	2.986014
C	1.349498	3.025224	3.417791
H	1.301014	4.091236	3.689744
H	2.364785	2.810113	3.058667
H	1.178355	2.449366	4.339934
C	-0.384948	2.474155	-2.698661
H	-0.533991	1.406841	-2.460627
C	0.514223	2.526704	-3.942199
H	1.525689	2.147016	-3.733460
H	0.612742	3.551350	-4.332542
H	0.078902	1.916014	-4.748065

C	-1.767847	3.084883	-2.996771
H	-2.444346	2.997381	-2.134054
H	-2.236421	2.582718	-3.856942
H	-1.669550	4.154735	-3.239623
C	-3.881267	0.003012	0.157103
C	-4.450224	-0.096051	1.438682
C	-5.821488	-0.378215	1.492555
H	-6.313389	-0.466574	2.463171
C	-6.565635	-0.548455	0.324967
H	-7.633470	-0.769740	0.391240
C	-5.960744	-0.432997	-0.927194
H	-6.561611	-0.565570	-1.828686
C	-4.593842	-0.149173	-1.045551
C	-3.637192	0.063135	2.717570
H	-2.615939	0.373287	2.445672
C	-4.205543	1.167975	3.622285
H	-3.561253	1.307206	4.504357
H	-5.213256	0.914391	3.986442
H	-4.269476	2.128899	3.089690
C	-3.523429	-1.278893	3.462237
H	-3.102777	-2.061299	2.812102
H	-4.511390	-1.626395	3.803715
H	-2.879200	-1.175755	4.349703
C	-3.929568	-0.057964	-2.414246
H	-2.919144	0.359934	-2.281213
C	-3.772869	-1.458022	-3.035134
H	-3.214459	-2.133551	-2.369689
H	-3.241843	-1.398212	-3.998290
H	-4.757472	-1.916343	-3.219896
C	-4.678407	0.892666	-3.360846
H	-4.786495	1.895282	-2.920991
H	-5.684251	0.516537	-3.603355
H	-4.130438	0.991491	-4.310670
C	2.813069	0.571712	0.024532
C	3.404748	0.871656	1.259422
C	4.303427	1.954227	1.271333
H	4.791662	2.215197	2.214357
C	3.981987	2.353475	-1.091412
H	4.197857	2.918461	-2.000881
C	3.087573	1.278768	-1.142263
H	2.619272	1.004515	-2.088429
I	3.529087	-2.682260	-0.320977
N	-2.528808	-2.797472	0.181541
C	4.589096	2.689080	0.119241
H	5.292187	3.523511	0.168541
C	3.134695	0.080154	2.514523
H	3.504204	-0.952321	2.409737
H	3.627030	0.531848	3.386448
H	2.055469	0.020583	2.732584

88
o-Me-TS-AB+ SCF Done: -1856.08366695 A.U.

Au	-1.472577	-0.929686	0.002298
N	0.830022	-2.887381	-0.533417
N	0.719454	1.206925	0.351858
N	2.018828	1.437389	0.364268
N	2.567019	0.306366	-0.007019
C	1.028915	-4.165253	-0.854333
H	0.163320	-4.832572	-0.783523
C	2.282904	-4.635667	-1.257925
H	2.446910	-5.683696	-1.513174
C	3.311327	-3.692668	-1.321074
H	4.322481	-3.972221	-1.635455
C	1.885060	-2.064130	-0.622096
C	1.623443	-0.663241	-0.248508
C	0.386168	-0.068635	-0.009391
C	-0.186182	2.261005	0.745547
C	-0.545336	3.216598	-0.223503
C	-1.415010	4.232802	0.191038
H	-1.722301	5.002189	-0.518444
C	-1.893470	4.277263	1.502039
H	-2.566579	5.083352	1.804202
C	-1.520208	3.305555	2.429535
H	-1.900797	3.360084	3.451637
C	-0.648678	2.265919	2.072895
C	0.035712	3.180057	-1.631813
H	0.332815	2.139013	-1.842817
C	1.304188	4.051368	-1.707529
H	1.756632	3.989000	-2.709497
H	2.055775	3.734375	-0.970063
H	1.058497	5.106910	-1.509206
C	-0.976405	3.578520	-2.713728
H	-1.247041	4.644187	-2.649767
H	-1.899607	2.986575	-2.635905
H	-0.542021	3.416809	-3.712457
C	-0.209329	1.232564	3.103941
H	0.451573	0.506163	2.607857
C	-1.401378	0.437884	3.660904
H	-1.937924	-0.092620	2.858141
H	-2.120806	1.091989	4.178395
H	-1.056354	-0.315427	4.386145
C	0.612848	1.890641	4.225594
H	1.482260	2.428611	3.817588
H	0.981387	1.127738	4.929386
H	0.007994	2.610992	4.798678
C	4.013022	0.217223	-0.009090
C	4.702830	0.596358	-1.171609
C	6.099616	0.495819	-1.130620

H	6.683696	0.774683	-2.009915
C	6.754571	0.043486	0.015551
H	7.844911	-0.025979	0.024337
C	6.029611	-0.314709	1.152587
H	6.558743	-0.658293	2.043874
C	4.630884	-0.235489	1.169016
C	3.990430	1.064073	-2.433904
H	2.916213	1.158380	-2.208671
C	4.475363	2.451539	-2.883163
H	3.895534	2.795085	-3.754357
H	5.535486	2.432233	-3.180721
H	4.362180	3.195565	-2.080512
C	4.132370	0.021650	-3.556888
H	3.768207	-0.963214	-3.228775
H	5.186129	-0.092383	-3.857793
H	3.561411	0.331187	-4.446783
C	3.843741	-0.629314	2.413399
H	2.770810	-0.493538	2.206230
C	4.047698	-2.114804	2.755713
H	3.785959	-2.760010	1.902531
H	3.420114	-2.402959	3.613872
H	5.095204	-2.324295	3.023978
C	4.180746	0.284820	3.603032
H	3.998599	1.341899	3.355286
H	5.236336	0.184659	3.900892
H	3.560961	0.024558	4.475708
N	3.113953	-2.409244	-1.008606
I	-3.778722	-2.256303	0.243792
C	-3.416989	0.185799	-0.534584
C	-3.607163	0.374665	-1.908844
C	-3.786126	1.088476	0.459089
C	-4.179110	1.614621	-2.256698
C	-4.344982	2.304918	0.057908
H	-3.637727	0.864418	1.514434
H	-4.340626	1.823572	-3.317565
H	-4.623681	3.037530	0.817267
C	-4.546185	2.562165	-1.299573
H	-4.996974	3.504453	-1.618132
C	-3.248438	-0.636485	-2.964056
H	-2.197911	-0.957081	-2.872742
H	-3.872123	-1.539835	-2.868313
H	-3.395523	-0.223588	-3.971115

103

o-Me-TS-CD+ SCF Done: -3249.65013219 A.U.

Au	-1.602114	-0.557880	-0.465514
N	-0.476668	-2.207616	0.393013
N	1.124177	1.391211	-0.481356
N	2.425909	1.157287	-0.333167

N	2.515261	-0.117916	-0.043562
C	-0.980058	-3.333360	0.908656
H	-2.064681	-3.448827	0.880302
C	-0.132760	-4.303632	1.439537
H	-0.534972	-5.218556	1.875355
C	1.241098	-4.063482	1.355865
H	1.968443	-4.804302	1.703477
C	0.872345	-2.016885	0.444090
C	1.281102	-0.693364	0.003223
C	0.349735	0.288188	-0.305331
C	0.677037	2.738729	-0.749458
C	0.611448	3.158406	-2.092172
C	0.101261	4.443483	-2.324458
H	0.024087	4.820916	-3.344717
C	-0.304194	5.252856	-1.262075
H	-0.703116	6.249603	-1.465417
C	-0.192682	4.811589	0.057723
H	-0.502039	5.470811	0.868849
C	0.311884	3.537128	0.355288
C	1.139467	2.286861	-3.227552
H	1.009129	1.231292	-2.929936
C	2.652114	2.520623	-3.417473
H	3.047193	1.862322	-4.206643
H	3.211037	2.323254	-2.491994
H	2.842769	3.564090	-3.714102
C	0.394806	2.484238	-4.555393
H	0.607396	3.469850	-4.997213
H	-0.697736	2.402292	-4.440476
H	0.720522	1.728453	-5.286466
C	0.520826	3.056834	1.786602
H	0.302543	1.976684	1.827313
C	-0.390202	3.745392	2.809989
H	-1.456831	3.665087	2.557010
H	-0.140248	4.811737	2.930095
H	-0.236197	3.281405	3.797783
C	1.996660	3.235654	2.199472
H	2.688103	2.755222	1.494718
H	2.161010	2.784916	3.189713
H	2.259011	4.304721	2.253058
C	3.819858	-0.746252	0.032117
C	4.341985	-1.265739	-1.169694
C	5.593234	-1.887991	-1.091094
H	6.042723	-2.312373	-1.990437
C	6.277890	-1.969189	0.123715
H	7.255405	-2.456089	0.161115
C	5.728615	-1.430141	1.285407
H	6.279780	-1.496615	2.225469
C	4.473879	-0.802333	1.272128
C	3.574542	-1.202205	-2.486910

H	2.798981	-0.425869	-2.395175
C	4.459656	-0.790306	-3.672334
H	3.843586	-0.651149	-4.574829
H	5.211809	-1.558468	-3.909495
H	4.988790	0.152564	-3.468405
C	2.859520	-2.538066	-2.762150
H	2.197621	-2.821738	-1.929880
H	3.591662	-3.351254	-2.889708
H	2.256641	-2.476247	-3.682779
C	3.878185	-0.223071	2.545077
H	2.871722	0.159341	2.324775
C	3.691995	-1.306173	3.618972
H	3.091293	-2.139587	3.228658
H	3.145529	-0.886667	4.476055
H	4.658661	-1.699125	3.974763
C	4.724201	0.951283	3.063687
H	4.830949	1.739988	2.302954
H	5.735898	0.623682	3.353170
H	4.249751	1.395738	3.952513
C	-3.250571	-1.822986	-0.678522
C	-4.325028	-1.664565	0.205981
H	-4.304543	-0.847514	0.929246
C	-5.404853	-2.551997	0.157363
H	-6.241402	-2.426651	0.849395
C	-4.332418	-3.734753	-1.655848
H	-4.336026	-4.551038	-2.384690
C	-3.226123	-2.860923	-1.628710
N	1.734683	-2.930927	0.853798
O	-2.807082	0.744051	1.303651
O	-3.534900	2.389289	2.612862
C	-3.328280	1.900699	1.491243
C	-3.766417	2.707273	0.265078
H	-3.733894	3.773034	0.529428
H	-4.830959	2.465865	0.092941
C	-2.959862	2.455258	-1.007034
C	-3.079092	1.085221	-1.580649
H	-4.048747	0.587588	-1.464643
C	-2.158096	0.496715	-2.431503
H	-2.453938	-0.349837	-3.054739
H	-1.256956	1.039586	-2.720595
H	-3.346136	3.118579	-1.806993
H	-1.904740	2.730083	-0.879282
K	-2.143497	0.574587	3.967262
Cl	0.340490	-0.528482	3.015636
C	-5.407816	-3.588449	-0.781049
H	-6.248881	-4.284025	-0.831348
C	-2.086374	-3.128256	-2.590175
H	-1.267991	-2.401299	-2.504798
H	-1.655299	-4.126892	-2.410682

H	-2.440397	-3.119864	-3.633811

88

o-Me-TS-decomp+ SCF Done: -1856.07731944 A.U.

Au	-1.896252	-0.505465	-0.429496
N	0.091050	-2.554128	-1.190750
N	0.492447	1.196353	0.723463
N	1.746557	0.979663	1.058657
N	2.128738	-0.053719	0.355466
C	0.145251	-3.743131	-1.792984
H	-0.783932	-4.322010	-1.821073
C	1.334815	-4.220258	-2.351820
H	1.389532	-5.197567	-2.833915
C	2.447593	-3.377157	-2.273499
H	3.411942	-3.665558	-2.704539
C	1.215177	-1.834547	-1.153793
C	1.121209	-0.546954	-0.431734
C	0.018985	0.291787	-0.206557
C	-0.208577	2.286459	1.363855
C	0.039709	3.592703	0.895542
C	-0.698478	4.619367	1.497500
H	-0.552799	5.650170	1.172906
C	-1.625140	4.342197	2.504128
H	-2.196821	5.158905	2.951240
C	-1.817270	3.037159	2.959703
H	-2.525084	2.849903	3.767873
C	-1.099507	1.966942	2.406688
C	1.086141	3.883471	-0.174389
H	1.175521	2.983698	-0.807718
C	2.456521	4.145769	0.482167
H	3.221703	4.325946	-0.288128
H	2.783580	3.300685	1.103420
H	2.405269	5.038796	1.124984
C	0.710464	5.047053	-1.102067
H	0.742410	6.013830	-0.576038
H	-0.292479	4.919150	-1.530737
H	1.431294	5.109470	-1.931623
C	-1.196526	0.563002	2.993730
H	-0.909571	-0.161844	2.216451
C	-2.611463	0.180824	3.442880
H	-3.343119	0.318294	2.632824
H	-2.939032	0.767771	4.314838
H	-2.637164	-0.879642	3.734746
C	-0.174236	0.417737	4.138256
H	0.849187	0.635405	3.795110
H	-0.191509	-0.606960	4.541078
H	-0.408294	1.112409	4.960411
C	3.459684	-0.589614	0.560919
C	4.519211	-0.027585	-0.172119

C	5.784093	-0.593299	0.031737
H	6.642663	-0.199655	-0.515329
C	5.962833	-1.655396	0.919674
H	6.959293	-2.080906	1.059955
C	4.883587	-2.173598	1.635599
H	5.045020	-2.997193	2.333951
C	3.594022	-1.648579	1.475856
C	4.320053	1.106874	-1.170037
H	3.304402	1.510953	-1.034405
C	5.294923	2.268982	-0.925080
H	5.067949	3.104195	-1.605973
H	6.337890	1.970486	-1.113186
H	5.228442	2.640157	0.108434
C	4.416480	0.583914	-2.614646
H	3.705528	-0.237513	-2.785199
H	5.427713	0.199737	-2.824209
H	4.208973	1.393484	-3.332499
C	2.419506	-2.221806	2.261392
H	1.516355	-1.637761	2.023331
C	2.135434	-3.679441	1.858134
H	1.957817	-3.775164	0.776222
H	1.245033	-4.057493	2.384145
H	2.981979	-4.335021	2.115846
C	2.640732	-2.090402	3.777594
H	2.825761	-1.044612	4.065785
H	3.500880	-2.690845	4.112403
H	1.753930	-2.445861	4.325047
C	-1.296397	1.220540	-1.560443
C	-0.829392	1.141468	-2.888904
C	-1.180655	2.214684	-3.728578
H	-0.843039	2.186835	-4.767677
C	-2.396841	3.332024	-1.962582
H	-3.010771	4.159387	-1.601218
C	-2.049448	2.306105	-1.083470
H	-2.400440	2.342754	-0.053847
I	-3.843995	-2.132353	0.170749
N	2.388692	-2.181487	-1.680660
C	-1.954032	3.288265	-3.286028
H	-2.211832	4.091984	-3.979183
C	-0.015441	0.006573	-3.445388
H	1.019858	0.039691	-3.068027
H	-0.443388	-0.968318	-3.176109
H	0.034482	0.066679	-4.540533

14

o-PhNO2I SCF Done: -447.238389619 A.U.

C	3.062293	-1.301798	0.037048
C	2.687564	0.038646	0.010713
C	1.333062	0.388055	0.034871

C 0.333229 -0.594289 0.043897
C 0.723261 -1.939979 0.048045
C 2.075240 -2.290995 0.057803
H 4.120351 -1.571629 0.035492
H 3.427593 0.838974 -0.026219
H -0.037836 -2.721976 0.038476
H 2.352666 -3.347621 0.072166
I -1.768786 -0.190094 -0.057025
O 1.736617 2.542647 -0.664750
O 0.172719 2.232455 0.804966
N 1.045756 1.839796 0.055206

87
o-NO2-A-ad+ SCF Done: -2021.15855508 A.U.
Au -1.025632 1.224607 -0.174852
N 1.770582 3.019909 -0.407824
N 0.848719 -1.109737 0.008460
N 2.093698 -1.532177 0.111131
N 2.819280 -0.441974 0.148535
C 2.233854 4.270151 -0.376905
H 1.588194 5.046390 -0.801545
C 3.484336 4.585339 0.164032
H 3.862137 5.608904 0.177034
C 4.219051 3.522263 0.694069
H 5.202086 3.679289 1.150935
C 2.562487 2.074155 0.113623
C 2.046792 0.693784 0.069607
C 0.728828 0.255283 -0.027474
C -0.222493 -2.080062 -0.052567
C -0.857033 -2.439905 1.148595
C -1.882520 -3.392698 1.058629
H -2.402441 -3.707248 1.965741
C -2.244685 -3.941218 -0.170562
H -3.040649 -4.688334 -0.219856
C -1.606803 -3.539469 -1.345771
H -1.924101 -3.963224 -2.298315
C -0.576675 -2.590689 -1.317401
C -0.494237 -1.821158 2.492211
H 0.363981 -1.148349 2.344370
C -0.061134 -2.883866 3.514770
H 0.258393 -2.403758 4.453145
H 0.779037 -3.484020 3.134834
H -0.886022 -3.571849 3.758614
C -1.656879 -0.963114 3.021459
H -2.549432 -1.578396 3.218162
H -1.940769 -0.187292 2.293022
H -1.375483 -0.465401 3.962944
C 0.148463 -2.161901 -2.586665
H 0.525957 -1.138943 -2.420409

C	-0.772735	-2.102398	-3.813515
H	-1.686418	-1.528857	-3.602507
H	-1.067671	-3.109290	-4.149251
H	-0.244088	-1.626471	-4.653841
C	1.362005	-3.075689	-2.846473
H	2.058674	-3.083392	-1.995362
H	1.911854	-2.739611	-3.739127
H	1.031520	-4.112070	-3.021299
C	4.261567	-0.580574	0.193829
C	4.871152	-0.667170	1.457321
C	6.261892	-0.818394	1.472084
H	6.787291	-0.897442	2.424858
C	6.990170	-0.862662	0.280692
H	8.076313	-0.976488	0.315465
C	6.346173	-0.763157	-0.951708
H	6.934341	-0.796552	-1.870477
C	4.952307	-0.624456	-1.027793
C	4.047168	-0.627780	2.736944
H	3.114225	-0.085385	2.511734
C	3.663524	-2.053733	3.175091
H	3.019912	-2.025815	4.068789
H	4.564065	-2.638642	3.421589
H	3.124481	-2.587406	2.377776
C	4.735843	0.143726	3.869876
H	5.035492	1.149722	3.538935
H	5.632052	-0.379164	4.238944
H	4.050337	0.251050	4.724955
C	4.249052	-0.478668	-2.372394
H	3.169790	-0.631803	-2.215722
C	4.437679	0.945183	-2.927608
H	4.073131	1.707704	-2.222498
H	3.892004	1.067596	-3.876573
H	5.503035	1.151264	-3.118346
C	4.698991	-1.538321	-3.389159
H	4.575303	-2.555900	-2.989373
H	5.754681	-1.409649	-3.674289
H	4.101200	-1.456636	-4.310332
N	3.758302	2.270216	0.675481
I	-3.553670	2.290837	-0.266900
C	-4.396530	0.463768	0.533445
C	-4.947113	0.587487	1.811359
H	-4.922507	1.547382	2.329676
C	-5.528972	-0.518368	2.439248
C	-5.035655	-1.875765	0.509244
H	-5.948863	-0.403146	3.440879
H	-5.060869	-2.822032	-0.028797
C	-5.572505	-1.751581	1.787801
H	-6.027413	-2.618649	2.270622
C	-4.439932	-0.777825	-0.122965

O	-4.264479	-2.035067	-2.050666
O	-3.044701	-0.261243	-1.912146
N	-3.876653	-1.040383	-1.470556

87
o-NO2-B+ SCF Done: -2021.17673775 A.U.

Au	1.347687	-1.017592	-0.238307
N	-0.269119	-2.584435	-0.143176
N	-0.789900	1.416012	-0.143267
N	-2.107916	1.519055	-0.020708
N	-2.553814	0.290137	0.063590
C	-0.131059	-3.914737	-0.145212
H	0.886433	-4.300254	-0.257416
C	-1.247282	-4.742510	-0.008461
H	-1.143273	-5.828219	-0.009953
C	-2.489480	-4.120300	0.129838
H	-3.408587	-4.704410	0.241218
C	-1.520442	-2.069889	0.001760
C	-1.529492	-0.609743	-0.010905
C	-0.361639	0.125879	-0.140836
C	-0.003051	2.621820	-0.313764
C	0.522512	3.238609	0.837485
C	1.285408	4.394986	0.628016
H	1.723919	4.913525	1.480757
C	1.492450	4.896275	-0.657787
H	2.089174	5.801346	-0.793646
C	0.947540	4.256734	-1.770831
H	1.122307	4.669208	-2.765168
C	0.183410	3.091385	-1.626894
C	0.227844	2.706045	2.235425
H	0.193726	1.605452	2.171156
C	-1.149802	3.201619	2.721455
H	-1.383643	2.773406	3.708329
H	-1.959775	2.931617	2.029467
H	-1.148307	4.298836	2.818507
C	1.305568	3.059112	3.269364
H	1.279292	4.128218	3.532544
H	2.318460	2.826488	2.914249
H	1.136537	2.490611	4.195740
C	-0.457789	2.403367	-2.826838
H	-0.587870	1.336864	-2.574779
C	0.406608	2.454323	-4.094236
H	1.429080	2.092201	-3.908114
H	0.478646	3.475332	-4.499707
H	-0.040439	1.827166	-4.880642
C	-1.856086	2.994104	-3.094415
H	-2.506653	2.917252	-2.211088
H	-2.344948	2.469985	-3.930146
H	-1.776995	4.060027	-3.360406

C	-3.969966	0.045022	0.245651
C	-4.446931	-0.048351	1.565258
C	-5.814098	-0.315673	1.714696
H	-6.237551	-0.400742	2.717242
C	-6.640843	-0.475108	0.602077
H	-7.703877	-0.683682	0.743818
C	-6.124869	-0.366195	-0.689786
H	-6.788958	-0.491025	-1.547124
C	-4.766110	-0.099760	-0.903664
C	-3.540591	0.093760	2.782365
H	-2.549952	0.434588	2.442683
C	-4.057283	1.156900	3.764318
H	-3.344891	1.284740	4.594026
H	-5.024565	0.867898	4.203785
H	-4.186281	2.131432	3.269779
C	-3.342704	-1.266805	3.474559
H	-2.957183	-2.021346	2.772028
H	-4.295321	-1.645325	3.878098
H	-2.633070	-1.175183	4.311672
C	-4.198801	-0.014284	-2.315303
H	-3.158508	0.342282	-2.250090
C	-4.167678	-1.405954	-2.972371
H	-3.606639	-2.126448	-2.357792
H	-3.699320	-1.354640	-3.968010
H	-5.187239	-1.803128	-3.099971
C	-4.959295	1.001470	-3.181901
H	-4.968412	1.998675	-2.716825
H	-6.003502	0.693508	-3.345988
H	-4.484449	1.088391	-4.171527
C	2.692479	0.508213	-0.209729
C	3.305322	0.921782	0.980912
C	4.273116	1.934003	0.982160
H	4.741306	2.209845	1.927027
C	3.994959	2.165954	-1.403376
H	4.256201	2.651812	-2.346185
C	3.049734	1.135125	-1.403410
H	2.601679	0.814362	-2.344476
I	3.408365	-2.707143	-0.475937
N	-2.622647	-2.788180	0.134946
C	4.610346	2.563091	-0.212379
H	5.356147	3.360143	-0.214297
O	3.702898	0.527294	3.220470
O	1.917524	-0.331046	2.367918
N	2.959291	0.321112	2.283699

87

o-NO2-TS-AB+ SCF Done: -2021.13435728 A.U.

Au	-1.317262	-0.952433	0.267322
N	0.821713	-2.891991	-0.107986

N	0.887609	1.250757	0.344435
N	2.191273	1.450201	0.262891
N	2.703007	0.264814	0.037186
C	0.935296	-4.198834	-0.338920
H	0.028834	-4.802400	-0.224181
C	2.156342	-4.772834	-0.708313
H	2.251854	-5.843536	-0.894862
C	3.244653	-3.905020	-0.826062
H	4.236680	-4.272039	-1.109950
C	1.926132	-2.143441	-0.257670
C	1.733020	-0.704547	-0.019998
C	0.519482	-0.054517	0.186646
C	0.010581	2.373254	0.579746
C	-0.336273	3.181717	-0.522857
C	-1.225397	4.232977	-0.267682
H	-1.529920	4.891653	-1.081543
C	-1.718311	4.462924	1.019262
H	-2.406409	5.294120	1.192096
C	-1.326676	3.657412	2.086575
H	-1.702662	3.868602	3.089876
C	-0.439928	2.587409	1.893297
C	0.232568	2.920873	-1.916057
H	1.243771	2.508220	-1.782901
C	0.394234	4.197128	-2.751781
H	0.948353	3.969532	-3.675187
H	0.948590	4.974269	-2.204066
H	-0.578030	4.617161	-3.054792
C	-0.594937	1.870904	-2.679016
H	-1.612873	2.242590	-2.869895
H	-0.676494	0.925933	-2.123100
H	-0.131023	1.648080	-3.652427
C	0.032816	1.746657	3.074549
H	0.777141	1.023422	2.708900
C	-1.115010	0.933973	3.695072
H	-1.542559	0.230795	2.963598
H	-1.922877	1.589690	4.057742
H	-0.751781	0.346332	4.552510
C	0.741289	2.618600	4.125690
H	1.570492	3.187722	3.678825
H	1.153118	1.987705	4.928945
H	0.048644	3.337620	4.590605
C	4.138915	0.159685	-0.124838
C	4.662757	0.364493	-1.409777
C	6.057244	0.297472	-1.533822
H	6.519020	0.457968	-2.509986
C	6.861103	0.027513	-0.426663
H	7.946198	-0.020468	-0.545082
C	6.295078	-0.183204	0.833057
H	6.945114	-0.394336	1.683102

C	4.908426	-0.122741	1.017159
C	3.776644	0.661702	-2.612530
H	2.727747	0.499976	-2.316716
C	3.905273	2.135813	-3.034098
H	3.219390	2.362181	-3.866138
H	4.929717	2.362000	-3.370113
H	3.670501	2.812123	-2.197551
C	4.053782	-0.296546	-3.781184
H	3.961003	-1.345671	-3.460921
H	5.065032	-0.155043	-4.193543
H	3.337706	-0.118921	-4.598921
C	4.263377	-0.301477	2.386911
H	3.239621	-0.680638	2.227591
C	4.981488	-1.335539	3.263760
H	5.108013	-2.293342	2.735880
H	4.401217	-1.520956	4.180853
H	5.976986	-0.985296	3.578276
C	4.143339	1.055728	3.107504
H	3.586156	1.787509	2.503498
H	5.141955	1.476463	3.305707
H	3.625037	0.938435	4.072620
N	3.131304	-2.592077	-0.606038
I	-3.685704	-2.225034	0.554357
C	-3.349167	0.104660	-0.093781
C	-3.558934	0.489315	-1.429427
C	-3.722373	0.971120	0.941771
C	-4.142905	1.729309	-1.717045
C	-4.273349	2.216461	0.641803
H	-3.589715	0.669226	1.979925
H	-4.291448	1.992518	-2.764469
H	-4.540163	2.887438	1.460462
C	-4.497969	2.593196	-0.686663
H	-4.948309	3.559720	-0.919267
O	-3.508841	0.003896	-3.681502
O	-2.396951	-1.293014	-2.360603
N	-3.130184	-0.333697	-2.577006

102
o-NO2-TS-CD+ SCF Done: -3414.71207581 A.U.

Au	1.525171	-0.475392	0.178766
N	0.437947	-2.009896	-0.917384
N	-1.236800	1.256327	0.722375
N	-2.535675	1.008072	0.571680
N	-2.600546	-0.184008	0.029818
C	0.966506	-3.023662	-1.604514
H	2.054653	-3.088384	-1.639789
C	0.140414	-3.948330	-2.240896
H	0.562809	-4.772534	-2.816356
C	-1.237634	-3.782144	-2.086451

H	-1.947463	-4.489178	-2.527623
C	-0.916923	-1.892386	-0.869555
C	-1.355856	-0.693600	-0.176148
C	-0.444405	0.239755	0.292809
C	-0.824565	2.528622	1.271294
C	-0.769900	2.655133	2.673393
C	-0.301799	3.877969	3.175489
H	-0.233569	4.030557	4.253177
C	0.068552	4.909624	2.312097
H	0.432862	5.853593	2.724596
C	-0.037309	4.757602	0.928226
H	0.239923	5.587664	0.278495
C	-0.497752	3.558925	0.364095
C	-1.270391	1.550299	3.600006
H	-1.131465	0.584479	3.083042
C	-2.782843	1.717453	3.852127
H	-3.158196	0.899764	4.486347
H	-3.352504	1.714199	2.912390
H	-2.980925	2.670308	4.367878
C	-0.510771	1.468212	4.931809
H	-0.727835	2.333103	5.577320
H	0.580835	1.423831	4.791666
H	-0.819897	0.568572	5.485329
C	-0.708975	3.392252	-1.135913
H	-0.439050	2.359735	-1.412565
C	0.147843	4.336754	-1.987522
H	1.222624	4.256097	-1.770564
H	-0.151685	5.389266	-1.861241
H	-0.009407	4.104675	-3.053828
C	-2.198549	3.580793	-1.491105
H	-2.853712	2.924709	-0.903270
H	-2.358666	3.345632	-2.554076
H	-2.513922	4.621086	-1.310013
C	-3.889799	-0.822615	-0.151195
C	-4.377989	-1.585160	0.928794
C	-5.614942	-2.212619	0.739768
H	-6.039335	-2.820318	1.540617
C	-6.316213	-2.069130	-0.459672
H	-7.281833	-2.565529	-0.582904
C	-5.798726	-1.296090	-1.497427
H	-6.362308	-1.190404	-2.426481
C	-4.560037	-0.649770	-1.371952
C	-3.589049	-1.767496	2.221625
H	-2.840649	-0.962008	2.282388
C	-4.465339	-1.638907	3.476146
H	-3.836567	-1.663969	4.380100
H	-5.184640	-2.468119	3.560643
H	-5.031780	-0.695510	3.476014
C	-2.826379	-3.105245	2.208168

H	-2.166718	-3.188314	1.331859
H	-3.530355	-3.951920	2.172488
H	-2.208308	-3.211831	3.113756
C	-3.994996	0.184299	-2.510567
H	-2.994694	0.541938	-2.228610
C	-3.798131	-0.660715	-3.778849
H	-3.184440	-1.546936	-3.563869
H	-3.262977	-0.070546	-4.536807
H	-4.760733	-0.991071	-4.202642
C	-4.873303	1.416956	-2.780353
H	-4.987561	2.037570	-1.878110
H	-5.881277	1.128239	-3.119726
H	-4.420552	2.038954	-3.568348
C	3.301294	-1.555015	0.004853
C	4.228576	-1.200469	-0.983342
H	4.017639	-0.332576	-1.609686
C	5.412923	-1.928584	-1.154313
H	6.114403	-1.635971	-1.939612
C	4.814272	-3.368556	0.686401
H	5.015602	-4.198388	1.365070
C	3.619082	-2.651049	0.823269
N	-1.756670	-2.764584	-1.396860
O	2.512768	1.289860	-1.240350
O	3.402982	3.094225	-2.177106
C	3.160378	2.396090	-1.181357
C	3.692349	2.862870	0.174641
H	3.747225	3.959890	0.152453
H	4.735718	2.506366	0.248867
C	2.888272	2.399565	1.390041
C	2.976116	0.947346	1.708135
H	3.950852	0.471300	1.549889
C	2.006115	0.223219	2.371382
H	2.237952	-0.736524	2.836609
H	1.091007	0.716992	2.696552
H	3.304589	2.895138	2.290245
H	1.840186	2.721010	1.328093
K	1.847382	1.717229	-3.873885
Cl	-0.448637	0.161625	-3.094999
C	5.709144	-3.011422	-0.319490
H	6.636424	-3.573656	-0.447209
O	3.194845	-3.580502	2.893901
O	1.492603	-2.997762	1.694693
N	2.700981	-3.111803	1.886224

87
o-NO2-TS-decomp+ SCF Done: -2021.12675920 A.U.

Au	-1.927180	-0.464785	-0.387601
N	0.037686	-2.686818	-0.656276
N	0.489084	1.230505	0.814575

N	1.726841	0.981214	1.184368
N	2.081292	-0.097483	0.535314
C	0.029448	-3.898384	-1.212505
H	-0.869479	-4.504357	-1.060088
C	1.122081	-4.365802	-1.949443
H	1.120265	-5.356876	-2.405892
C	2.217505	-3.506522	-2.071879
H	3.114311	-3.801072	-2.626320
C	1.123765	-1.938706	-0.862157
C	1.074307	-0.592836	-0.243700
C	0.006284	0.302004	-0.089172
C	-0.200205	2.354932	1.405910
C	0.117861	3.648490	0.941371
C	-0.620883	4.704532	1.490178
H	-0.422500	5.726650	1.166250
C	-1.611916	4.469146	2.444596
H	-2.181560	5.308826	2.849786
C	-1.868530	3.176850	2.904517
H	-2.623005	3.022394	3.676491
C	-1.155067	2.077828	2.405296
C	1.236944	3.900471	-0.063040
H	1.331167	3.000181	-0.695507
C	2.574837	4.107517	0.676162
H	3.387450	4.272470	-0.047117
H	2.838132	3.243916	1.301387
H	2.514070	4.993460	1.327833
C	0.969455	5.087575	-0.999187
H	1.035635	6.048092	-0.465162
H	-0.019832	5.026359	-1.471819
H	1.727864	5.111144	-1.796423
C	-1.318015	0.690213	3.017114
H	-1.021331	-0.061513	2.270238
C	-2.759855	0.358749	3.418011
H	-3.455568	0.499322	2.577428
H	-3.103498	0.972638	4.264822
H	-2.827341	-0.694090	3.729913
C	-0.346245	0.544032	4.205080
H	0.695947	0.724701	3.899333
H	-0.408914	-0.470360	4.628833
H	-0.592969	1.263536	5.001723
C	3.395386	-0.668815	0.751765
C	4.453941	-0.201318	-0.046720
C	5.702923	-0.797180	0.170889
H	6.560375	-0.475003	-0.422879
C	5.866091	-1.798454	1.129176
H	6.850515	-2.248131	1.278762
C	4.786032	-2.229651	1.900040
H	4.934552	-3.010834	2.648158
C	3.511560	-1.672772	1.729389

C	4.279886	0.876423	-1.109459
H	3.252608	1.269506	-1.040785
C	5.231735	2.059932	-0.869244
H	5.023030	2.867465	-1.588520
H	6.283359	1.763747	-1.006716
H	5.125196	2.466090	0.147549
C	4.446122	0.293479	-2.523680
H	3.732762	-0.522832	-2.699284
H	5.466307	-0.096588	-2.669175
H	4.271838	1.074432	-3.280142
C	2.334093	-2.155038	2.568659
H	1.434871	-1.590954	2.273644
C	2.036854	-3.642267	2.310565
H	1.869416	-3.841131	1.241336
H	1.135497	-3.954618	2.860485
H	2.870733	-4.279566	2.643898
C	2.562907	-1.875197	4.063565
H	2.752538	-0.806617	4.246794
H	3.425075	-2.441462	4.449097
H	1.679107	-2.173187	4.648879
C	-1.146400	1.195136	-1.475076
C	-0.553400	1.133220	-2.748487
C	-0.655377	2.185283	-3.655560
H	-0.153520	2.098185	-4.620560
C	-2.029096	3.379280	-2.067682
H	-2.631323	4.248431	-1.795971
C	-1.863461	2.354572	-1.131110
H	-2.323041	2.451200	-0.148837
I	-3.992993	-1.944651	0.159945
N	2.220265	-2.286023	-1.528233
C	-1.419544	3.304941	-3.322169
H	-1.532260	4.118384	-4.041435
O	1.272716	0.113706	-3.699074
O	-0.364381	-1.139593	-3.021933
N	0.180627	-0.058576	-3.191274

16
o-PhOMeI SCF Done: -357.341883993 A.U.

C	3.021789	-1.227732	0.000004
C	2.645121	0.118225	0.000013
C	1.287059	0.483215	-0.000001
C	0.320727	-0.545250	0.000001
C	0.700946	-1.885456	-0.000004
C	2.056822	-2.234104	-0.000011
H	4.084234	-1.483653	0.000010
H	3.416055	0.888807	0.000028
H	-0.063847	-2.664502	-0.000008
H	2.346573	-3.287063	-0.000018
I	-1.763732	-0.039107	0.000000

O	0.849713	1.759561	0.000018
C	1.778635	2.821715	-0.000017
H	2.421004	2.805662	-0.899154
H	1.188388	3.747591	0.000011
H	2.421076	2.805649	0.899068

89

o-OMe-A-ad+ SCF Done: -1931.26111318 A.U.

Au	-1.076864	-1.152921	-0.224239
N	1.696567	-3.036041	0.078487
N	0.862026	1.113008	-0.263794
N	2.108185	1.530963	-0.171642
N	2.804551	0.452396	0.093991
C	2.123553	-4.270914	0.345203
H	1.475853	-5.095927	0.029655
C	3.339962	-4.512260	0.991801
H	3.688151	-5.525373	1.199093
C	4.080410	-3.388320	1.364971
H	5.038538	-3.483556	1.887175
C	2.490098	-2.030204	0.468490
C	2.010392	-0.669523	0.167860
C	0.709519	-0.235865	-0.074772
C	-0.199937	2.050949	-0.544904
C	-0.565653	2.241716	-1.891389
C	-1.631641	3.117050	-2.133170
H	-1.952266	3.307770	-3.158186
C	-2.294729	3.748077	-1.078468
H	-3.128375	4.421254	-1.290416
C	-1.908743	3.522958	0.241546
H	-2.447941	4.015949	1.052564
C	-0.843250	2.662953	0.543120
C	0.192740	1.559722	-3.023240
H	0.614809	0.623392	-2.623436
C	1.370594	2.439446	-3.484202
H	1.953017	1.928349	-4.266662
H	2.048486	2.673207	-2.649534
H	1.003883	3.392224	-3.898400
C	-0.706901	1.163546	-4.200924
H	-1.087393	2.042859	-4.744014
H	-1.570412	0.568154	-3.864352
H	-0.136464	0.560629	-4.924136
C	-0.447216	2.388798	1.988173
H	0.456642	1.761217	1.987779
C	-1.550958	1.595958	2.707903
H	-1.772281	0.650603	2.188645
H	-2.484832	2.178506	2.750769
H	-1.248151	1.360994	3.740869
C	-0.091938	3.681109	2.739983
H	0.708021	4.235003	2.225985

H	0.254928	3.447133	3.758756
H	-0.962378	4.349828	2.831763
C	4.245629	0.575485	0.183683
C	4.988356	0.405979	-0.995390
C	6.380319	0.537580	-0.881065
H	7.007268	0.408906	-1.765276
C	6.973184	0.833103	0.345510
H	8.058952	0.937033	0.410162
C	6.194147	0.997439	1.493715
H	6.680394	1.227129	2.442837
C	4.802519	0.864084	1.441487
C	4.340218	0.047328	-2.327704
H	3.254751	0.210889	-2.239589
C	4.825685	0.944272	-3.476300
H	4.272273	0.710941	-4.399398
H	5.895013	0.792366	-3.690337
H	4.672838	2.009202	-3.244866
C	4.556104	-1.443244	-2.647352
H	4.167940	-2.089459	-1.845020
H	5.628603	-1.666524	-2.764360
H	4.047567	-1.717556	-3.585207
C	3.922693	1.048953	2.670199
H	2.995122	0.478309	2.498951
C	4.551724	0.482723	3.949404
H	4.855954	-0.565891	3.810839
H	3.827225	0.525685	4.777551
H	5.435533	1.059507	4.264212
C	3.534744	2.530134	2.838825
H	3.036172	2.920736	1.938987
H	4.429399	3.146599	3.021579
H	2.852535	2.658071	3.694413
N	3.655500	-2.149312	1.110375
I	-3.616335	-2.071731	-0.524989
C	-4.449314	-0.125711	-0.084926
C	-4.815010	0.709874	-1.134684
C	-4.601769	0.240191	1.266042
H	-4.671444	0.395756	-2.169992
C	-5.370251	1.961130	-0.845786
C	-5.165572	1.500319	1.532125
H	-5.664376	2.623359	-1.661951
H	-5.308057	1.823166	2.563302
C	-5.544369	2.343654	0.484869
H	-5.982321	3.316461	0.720235
O	-4.187771	-0.628480	2.207180
C	-4.453538	-0.370579	3.579089
H	-4.083041	-1.244112	4.129568
H	-3.923025	0.529401	3.930264
H	-5.535488	-0.256428	3.760737

89
o-OMe-B+ SCF Done: -1931.27003280 A.U.

Au	1.381881	-0.973637	-0.262749
N	-0.246610	-2.590667	-0.185895
N	-0.786251	1.414140	0.003339
N	-2.105098	1.498038	0.127213
N	-2.539633	0.261978	0.144929
C	-0.108742	-3.918354	-0.250075
H	0.908362	-4.296128	-0.392972
C	-1.223308	-4.752855	-0.137556
H	-1.121128	-5.837462	-0.190975
C	-2.463091	-4.137319	0.047035
H	-3.380387	-4.726411	0.147150
C	-1.494012	-2.084334	-0.008383
C	-1.505667	-0.623551	0.036102
C	-0.342559	0.129246	-0.056111
C	-0.018834	2.639706	-0.102156
C	0.624921	3.122166	1.054033
C	1.356690	4.306361	0.906173
H	1.885147	4.725803	1.761832
C	1.423069	4.963166	-0.323943
H	1.999586	5.887059	-0.410555
C	0.769061	4.451371	-1.442480
H	0.838610	4.979626	-2.394263
C	0.032596	3.261855	-1.361706
C	0.489663	2.411465	2.397289
H	0.548197	1.327330	2.201321
C	-0.877326	2.717265	3.043371
H	-1.000124	2.143136	3.975254
H	-1.721460	2.478841	2.381984
H	-0.950079	3.787672	3.292761
C	1.617466	2.740165	3.381962
H	1.538392	3.771940	3.759273
H	2.606033	2.621256	2.919779
H	1.559548	2.070316	4.253103
C	-0.705333	2.704820	-2.573667
H	-0.871836	1.627231	-2.403932
C	0.097853	2.824337	-3.877127
H	1.106421	2.395478	-3.777098
H	0.207161	3.872365	-4.195946
H	-0.421149	2.292996	-4.689724
C	-2.085225	3.375134	-2.714141
H	-2.696109	3.235374	-1.810255
H	-2.634069	2.956731	-3.571662
H	-1.970386	4.458084	-2.879539
C	-3.958256	-0.001727	0.277576
C	-4.466948	-0.191663	1.573770
C	-5.837130	-0.467601	1.672675
H	-6.283561	-0.621350	2.657086

C -6.636964 -0.548162 0.532879
H -7.702758 -0.766217 0.633787
C -6.090243 -0.347832 -0.735485
H -6.734054 -0.412956 -1.614288
C -4.728071 -0.064037 -0.898091
C -3.595666 -0.123186 2.821598
H -2.572779 0.150362 2.519136
C -4.076934 0.970882 3.788322
H -3.395874 1.045569 4.650377
H -5.083057 0.750271 4.177809
H -4.113498 1.953858 3.294571
C -3.512452 -1.496251 3.510942
H -3.157492 -2.270491 2.813554
H -4.497986 -1.812918 3.887422
H -2.824066 -1.455731 4.369817
C -4.123338 0.112910 -2.285934
H -3.121404 0.555403 -2.171462
C -3.950526 -1.252770 -2.975817
H -3.347239 -1.940255 -2.363886
H -3.460156 -1.134255 -3.954979
H -4.928540 -1.731910 -3.142085
C -4.933823 1.081592 -3.160021
H -5.061534 2.056895 -2.667251
H -5.933256 0.682869 -3.392760
H -4.419153 1.246154 -4.119337
C 2.702028 0.583843 -0.235896
C 3.400938 0.804253 0.961715
C 4.370000 1.818093 0.991620
H 4.944779 1.999237 1.900368
C 3.881003 2.384992 -1.318549
H 4.059907 2.998015 -2.203968
C 2.929053 1.357073 -1.365979
H 2.383527 1.160343 -2.290130
I 3.429310 -2.649578 -0.617322
N -2.596591 -2.806839 0.111791
C 4.599808 2.602320 -0.143623
H 5.353885 3.391502 -0.101780
O 3.074595 0.019424 2.021861
C 3.981179 -0.093765 3.108442
H 3.592830 -0.893303 3.752220
H 4.989696 -0.367106 2.755630
H 4.036235 0.840141 3.692777

89
o-OMe-TS-AB+ SCF Done: -1931.23568645 A.U.
Au -1.377926 -0.941982 0.182253
N 0.847885 -2.905203 -0.211255
N 0.823076 1.244030 0.264111
N 2.122292 1.469321 0.191956

N	2.658434	0.295266	-0.036430
C	1.012844	-4.217298	-0.367908
H	0.113991	-4.839532	-0.299849
C	2.273772	-4.775065	-0.604770
H	2.410621	-5.850302	-0.729431
C	3.346502	-3.883170	-0.673618
H	4.367483	-4.233783	-0.858562
C	1.942515	-2.134248	-0.295629
C	1.706015	-0.693167	-0.107023
C	0.477347	-0.065945	0.090087
C	-0.073784	2.345569	0.526793
C	-0.556414	3.075401	-0.576509
C	-1.439878	4.124382	-0.295592
H	-1.844904	4.723428	-1.111946
C	-1.810263	4.417055	1.018553
H	-2.501435	5.240539	1.213448
C	-1.308119	3.671071	2.083165
H	-1.609146	3.916860	3.103423
C	-0.418366	2.609358	1.863046
C	-0.079285	2.777176	-1.993109
H	0.189001	1.708279	-2.036065
C	1.190086	3.591632	-2.310145
H	1.568127	3.343721	-3.314170
H	1.990863	3.393758	-1.583070
H	0.970082	4.670831	-2.285003
C	-1.157659	3.004448	-3.059511
H	-1.397434	4.072495	-3.180632
H	-2.086377	2.474795	-2.805107
H	-0.803216	2.641427	-4.036774
C	0.129948	1.795960	3.030041
H	0.916772	1.128726	2.646468
C	-0.961043	0.903015	3.645760
H	-1.363459	0.196324	2.903284
H	-1.797521	1.507179	4.032527
H	-0.554001	0.316384	4.484234
C	0.788830	2.692677	4.090602
H	1.572059	3.326475	3.648031
H	1.251529	2.075055	4.876043
H	0.055182	3.351552	4.580724
C	4.100941	0.211451	-0.151832
C	4.674560	0.411725	-1.416571
C	6.072105	0.325045	-1.489798
H	6.569443	0.466559	-2.451238
C	6.834593	0.058539	-0.353514
H	7.922571	-0.002041	-0.433627
C	6.222059	-0.129168	0.887556
H	6.838954	-0.331046	1.764284
C	4.830237	-0.060762	1.019354
C	3.841100	0.663514	-2.666776

H	2.800415	0.848413	-2.357039
C	4.304405	1.913975	-3.429723
H	3.637018	2.107697	-4.284161
H	5.322208	1.791263	-3.831584
H	4.299968	2.803746	-2.782363
C	3.836538	-0.581927	-3.571282
H	3.485331	-1.469669	-3.024263
H	4.850446	-0.799609	-3.943559
H	3.183258	-0.425240	-4.444476
C	4.141510	-0.222165	2.369304
H	3.107972	-0.558679	2.182961
C	4.794619	-1.290464	3.256207
H	4.893103	-2.248917	2.723470
H	4.184874	-1.457424	4.157685
H	5.796207	-0.985094	3.596974
C	4.057305	1.135600	3.092482
H	3.539405	1.887439	2.478171
H	5.066222	1.518977	3.313067
H	3.514464	1.035444	4.045626
N	3.183475	-2.566181	-0.522073
I	-3.642140	-2.335462	0.500268
C	-3.377967	0.117851	-0.063633
C	-3.675269	0.388402	-1.414532
C	-3.749472	0.963511	0.976746
C	-4.352345	1.587606	-1.691163
C	-4.404848	2.160268	0.670489
H	-3.531185	0.697264	2.010588
H	-4.603959	1.840235	-2.721326
H	-4.679474	2.842654	1.476391
C	-4.710288	2.458562	-0.658081
H	-5.240668	3.381497	-0.902526
O	-3.273567	-0.505318	-2.338239
C	-3.616760	-0.313819	-3.703212
H	-3.221868	-1.184374	-4.241255
H	-4.710529	-0.268201	-3.838817
H	-3.156805	0.603350	-4.108514

104
o-OMe-TS-CD+ SCF Done: -3324.80831114 A.U.

Au	1.551663	-0.551597	0.263909
N	0.456123	-2.003201	-0.903764
N	-1.191333	1.263369	0.769809
N	-2.492605	1.043191	0.597908
N	-2.570240	-0.141214	0.039732
C	0.975827	-2.992001	-1.635644
H	2.064040	-3.070756	-1.658162
C	0.139861	-3.870589	-2.323027
H	0.554253	-4.670821	-2.936815
C	-1.236510	-3.694490	-2.165322

H	-1.952950	-4.373688	-2.638655
C	-0.900083	-1.858954	-0.871687
C	-1.330029	-0.670901	-0.151664
C	-0.408473	0.238614	0.345219
C	-0.751488	2.527559	1.315047
C	-0.610428	2.630199	2.712739
C	-0.117799	3.846386	3.206519
H	0.014932	3.982350	4.280414
C	0.196186	4.894331	2.339109
H	0.579682	5.833548	2.745098
C	0.014686	4.762343	0.961470
H	0.256072	5.600411	0.307374
C	-0.471516	3.569220	0.405901
C	-1.049513	1.502121	3.641762
H	-0.900642	0.547935	3.106480
C	-2.556688	1.624727	3.945876
H	-2.892148	0.786699	4.576284
H	-3.157128	1.624760	3.025217
H	-2.762544	2.562936	4.484943
C	-0.242978	1.423638	4.945233
H	-0.455328	2.277410	5.607039
H	0.843606	1.404327	4.765465
H	-0.513298	0.512431	5.500487
C	-0.748780	3.420511	-1.084996
H	-0.538454	2.378417	-1.377563
C	0.120492	4.321365	-1.970541
H	1.197011	4.193143	-1.789409
H	-0.128045	5.387287	-1.843182
H	-0.076626	4.082334	-3.028003
C	-2.241079	3.683597	-1.374571
H	-2.900142	3.046300	-0.769870
H	-2.455279	3.479616	-2.434412
H	-2.499289	4.733927	-1.162756
C	-3.867280	-0.759352	-0.154513
C	-4.364979	-1.534285	0.912412
C	-5.608971	-2.144878	0.715525
H	-6.039369	-2.760713	1.507070
C	-6.309999	-1.973608	-0.480324
H	-7.281344	-2.456766	-0.610943
C	-5.784500	-1.188599	-1.504702
H	-6.347925	-1.060576	-2.431024
C	-4.538479	-0.557675	-1.370309
C	-3.578931	-1.747429	2.202562
H	-2.805537	-0.966425	2.267814
C	-4.448379	-1.600320	3.459784
H	-3.821199	-1.660615	4.363320
H	-5.201231	-2.400153	3.534436
H	-4.975555	-0.634579	3.471409
C	-2.858151	-3.108020	2.176879

H	-2.209140	-3.204640	1.293369
H	-3.587256	-3.933146	2.140816
H	-2.240198	-3.238952	3.080101
C	-3.968933	0.289305	-2.497069
H	-2.970876	0.645345	-2.205584
C	-3.764865	-0.543480	-3.772436
H	-3.140561	-1.423936	-3.565613
H	-3.237315	0.058398	-4.526540
H	-4.724893	-0.880019	-4.197385
C	-4.849238	1.521938	-2.760375
H	-4.971369	2.133352	-1.853130
H	-5.853772	1.234231	-3.110649
H	-4.392713	2.152910	-3.538915
C	3.257602	-1.730359	0.141100
C	4.273013	-1.464634	-0.776103
H	4.220145	-0.548026	-1.367907
C	5.344381	-2.360778	-0.917477
H	6.138672	-2.150706	-1.637310
C	4.379665	-3.779433	0.803605
H	4.438612	-4.682249	1.411939
C	3.307146	-2.885699	0.946261
N	-1.747487	-2.700797	-1.435526
O	2.906129	1.240512	-1.251020
O	3.351113	3.158431	-2.313046
C	3.289809	2.458362	-1.288495
C	3.733216	3.078949	0.043193
H	3.605148	4.168655	-0.008383
H	4.817824	2.893841	0.140817
C	2.989610	2.503176	1.242569
C	3.167535	1.049260	1.470583
H	4.122569	0.606585	1.167828
C	2.289825	0.262022	2.210729
H	2.637595	-0.673884	2.656993
H	1.426439	0.740809	2.676396
H	3.384662	2.961209	2.174425
H	1.920961	2.759215	1.225424
K	2.005912	1.507182	-3.854563
Cl	-0.424393	0.194158	-3.042134
C	5.389147	-3.510600	-0.129051
H	6.220158	-4.212869	-0.229525
O	2.273267	-3.063624	1.821621
C	2.244807	-4.216938	2.645192
H	2.208702	-5.143175	2.045597
H	3.118556	-4.258033	3.318580
H	1.329741	-4.144254	3.247421

89

o-OMe-TS-decomp+ SCF Done: -1931.23637056 A.U.

Au	-1.918133	-0.466929	-0.381100

N	0.061990	-2.610512	-0.955603
N	0.445295	1.187015	0.830250
N	1.696327	0.990620	1.191752
N	2.094844	-0.064817	0.527484
C	0.105543	-3.809883	-1.533927
H	-0.827797	-4.382632	-1.549381
C	1.291301	-4.312027	-2.082030
H	1.336123	-5.300644	-2.541603
C	2.413661	-3.482772	-2.012801
H	3.379027	-3.794863	-2.424989
C	1.191709	-1.897689	-0.941161
C	1.102582	-0.592683	-0.254612
C	-0.001747	0.246013	-0.070612
C	-0.285425	2.298204	1.397555
C	-0.006911	3.591587	0.908267
C	-0.781881	4.634163	1.432661
H	-0.615873	5.655187	1.087854
C	-1.769168	4.386154	2.387437
H	-2.367934	5.214925	2.772612
C	-1.987170	3.095643	2.871729
H	-2.741915	2.932686	3.641439
C	-1.236090	2.010881	2.397651
C	1.109952	3.858889	-0.095478
H	1.226035	2.952379	-0.714975
C	2.440203	4.112261	0.643650
H	3.253386	4.276765	-0.079705
H	2.719514	3.271188	1.292111
H	2.358519	5.013083	1.272257
C	0.815299	5.022708	-1.052010
H	0.841717	5.992743	-0.531709
H	-0.163641	4.916866	-1.537503
H	1.584025	5.060445	-1.839014
C	-1.352223	0.628461	3.032499
H	-1.088720	-0.126796	2.276363
C	-2.765954	0.284729	3.513281
H	-3.507709	0.413086	2.711056
H	-3.068721	0.900671	4.374277
H	-2.804439	-0.766526	3.835561
C	-0.317954	0.500788	4.169273
H	0.705852	0.688155	3.809773
H	-0.349316	-0.510283	4.604824
H	-0.530028	1.225343	4.971369
C	3.434090	-0.570293	0.746945
C	4.484341	0.003545	0.007920
C	5.759298	-0.534458	0.221839
H	6.612042	-0.132344	-0.327686
C	5.956812	-1.580011	1.126132
H	6.961388	-1.983119	1.274576
C	4.887174	-2.108498	1.848345

H	5.063569	-2.917231	2.560375
C	3.587574	-1.611923	1.677426
C	4.256038	1.117634	-1.007789
H	3.269370	1.563531	-0.809417
C	5.283340	2.251661	-0.876824
H	5.020417	3.079274	-1.554078
H	6.296685	1.919495	-1.150798
H	5.319556	2.646295	0.149606
C	4.223375	0.549823	-2.438659
H	3.470106	-0.245283	-2.531739
H	5.200051	0.115611	-2.706713
H	3.998041	1.348430	-3.164102
C	2.422268	-2.191638	2.471463
H	1.505749	-1.642200	2.204515
C	2.182682	-3.669402	2.116437
H	2.018835	-3.805441	1.036586
H	1.297816	-4.053759	2.647214
H	3.043599	-4.293027	2.404217
C	2.624728	-1.998896	3.983820
H	2.772420	-0.937577	4.235143
H	3.501528	-2.557581	4.346848
H	1.744811	-2.363384	4.536671
C	-1.200337	1.119690	-1.580768
C	-0.563929	0.874837	-2.819248
C	-0.665920	1.859096	-3.816136
H	-0.191010	1.704362	-4.784653
C	-2.014012	3.257271	-2.354150
H	-2.589250	4.167601	-2.177108
C	-1.895686	2.309592	-1.335230
H	-2.371078	2.485836	-0.370586
I	-3.983268	-1.952606	0.187971
N	2.364667	-2.270875	-1.450754
C	-1.393126	3.028923	-3.584380
H	-1.471215	3.771774	-4.381345
O	0.093927	-0.290058	-2.966648
C	0.765552	-0.581502	-4.185391
H	0.059246	-0.622707	-5.030649
H	1.556667	0.157846	-4.393034
H	1.225021	-1.567529	-4.047447
